# Genomic characterization of early-stage esophageal squamous cell carcinoma in a Japanese population

**DOI:** 10.18632/oncotarget.27014

**Published:** 2019-06-25

**Authors:** Yuji Urabe, Kenichi Kagemoto, C. Nelson Hayes, Koki Nakamura, Kazuhiko Masuda, Atsushi Ono, Shinji Tanaka, Koji Arihiro, Kazuaki Chayama

**Affiliations:** ^1^Division of Regeneration and Medicine Center for Translational and Clinical Research, Hiroshima University Hospital, Hiroshima, Japan; ^2^Department of Gastroenterology and Metabolism, Hiroshima University Hospital, Hiroshima, Japan; ^3^Department of Endoscopy, Hiroshima University Hospital, Hiroshima, Japan; ^4^Department of Pathology, Hiroshima University Hospital, Hiroshima, Japan

**Keywords:** early-stage esophageal squamous cell carcinoma, exome sequencing, target sequencing, genomic characterization, TP53

## Abstract

Major risk factors for esophageal squamous cell carcinoma (ESCC) are smoking, alcohol consumption, and single nucleotide polymorphisms in *ADH1B* and *ALDH2*. Several groups have reported large-scale genomic analyses of ESCCs. However, the specific genetic changes that promote the development of ESCC have not been characterized. We performed exome sequencing of 16 fresh esophageal squamous cell neoplasms and targeted sequencing of 128 genes in 52 archival specimens, of which 26 were cancerous, and 26 were adjacent normal tissue, from Japanese ESCC patients. We found significantly more somatic mutations in *TP53* and *NOTCH1*, *CDKN2A* deletions, and *CCND1* amplifications in cancerous areas than in non-cancerous areas, consistent with previous studies that have characterized them as tumor suppressors and oncogenes. These data suggest that mutations, deletions, and amplifications, which alter the function of *TP53*, *NOTCH1*, *CDKN2A*, and *CCND1*, are the key changes that promote the transformation of esophageal mucosa to ESCC.

## INTRODUCTION

Esophageal cancer is the sixth most common cause of cancer death worldwide, and its incidence has increased in recent years. There are two general types of esophageal cancer: squamous cell carcinoma (SCC) and adenocarcinoma. Esophageal SCC (ESCC) is the most common type of esophageal cancer in Asian countries, accounting for approximately 80% of esophageal cancer cases [1]. The most common cause of ESCC is damage to the esophageal mucosa by chronic inflammation due to exposure to acetaldehyde or other such carcinogens [2]. The genetic risk factors for ESCC are well known; common germline variants for ESCC are rs1229984 on *ADH1B* and rs671 on *ALDH2* [3]. In addition, somatic mutations and copy-number variants (CNVs) have been implicated in the development and proliferation of ESCC [4–6]. A recent study on ESCC in Japan has highlighted the roles of multiple recurrently altered genes in the pathogenesis of ESCC, including those that regulate the cell cycle (*TP53*, *CCND1*, *CDKN2A*, *FBXW7*); epigenetic processes (*MLL2*, *EP300*, *CREBBP*, *TET2*); and the NOTCH (*NOTCH1*, *NOTCH3*), WNT (*FAT1*, *YAP1*, *AJUBA*), and receptor-tyrosine kinase phosphoinositide 3-kinase signaling pathways (*PIK3CA*, *EGFR*, *ERBB2*) [7]. The evidence thus far on the genetic evolution of cancer from premalignant lesions suggests that the accumulation of mutations is stepwise [8–10]. The esophageal mucosa modified by inflammation develops into carcinoma through the development of intraepithelial neoplasia (IN). Therefore, identifying mutations associated with the development of SCC in the background mucosa and IN is essential. Hotspot genes and the allelic loss of tumor-suppressor genes in precancerous lesions of ESCC have been studied extensively [11–13]. Recently, Liu et al. reported mutations and gene copy-number changes in non-tumor, IN, and ESCC samples, collected from 70 advanced-ESCC patients [14]. This paper shows a panorama of the genetic architecture of the carcinogenesis process in ESCC development from IN and background mucosa. However, differences in ethnicity, lifestyle, and tumor stage have considerable influences on the mutation patterns of carcinoma. Furthermore, the effects of genetic mutations and alteration on early-stage ESCC have never been researched.

In light of the lack of the said research, we evaluated the somatic mutations and copy-number variants in 42 T1 or High-grade IN (HGIN) patients to study the genetic changes in the early-stage development of ESCC. To identify genomic changes underlying the transition from the non-dysplastic epithelium and IN to ESCC, we performed whole-exome sequencing (WES, screening-stage) and targeted sequencing (replication-stage) with matched samples (non-dysplastic epithelium/IN and ESCC) derived by microdissection from the same individuals. We aimed to determine the early-stage genomic alteration events that contribute to ESCC carcinogenesis (Supplementary Figure 1).

## RESULTS

### Patient characteristics in this study

Patient characteristics are shown in Supplementary Table 1. Genotyping indicated that 5 patients harbored the rs1229984 GG allele in *ADH1B,* and 29 patients harbored the rs671 GA allele in ALDH2. Therefore, 7 cases had no risk SNPs (rs671 AA or GG and rs1229984 AA or GA), 33 cases had 1 risk SNP (rs671 GA or rs1229984 GG), and 2 cases had 2 risk SNPs (rs671 GA and rs1229984 GG). Three non-smokers, 21 light smokers (less than Brickman index 1000), and 18 heavy smokers (more than Brickman index 1000) were included in this study. In addition, 1 non-drinker, 16 light drinkers (alcohol consumption was less than 60 g/day), and 25 heavy drinkers (alcohol consumption was more than 60 g/day) were included. All patients had a history of smoking and/or alcohol consumption.

### In the screening stage

**Figure 1:**
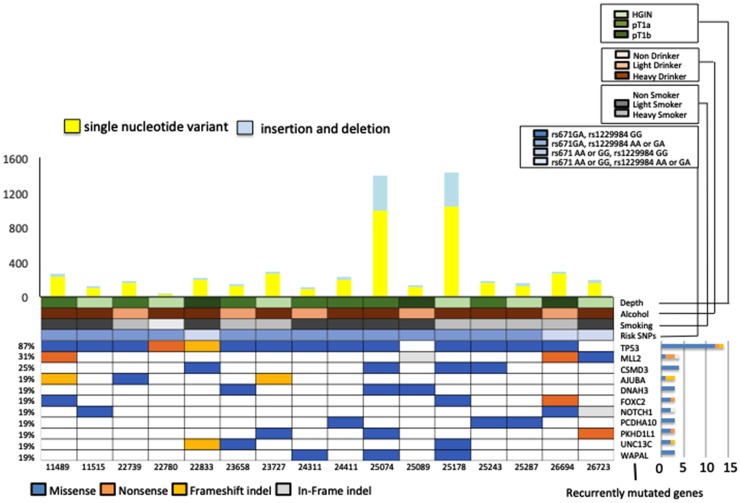
Representative exonic somatic mutations in 16 early-stage esophageal squamous cell carcinoma (ESCC) samples (High-grade intraepithelial neoplasm 6, T1a 7, T1b 3 samples). (Upper panel) The number of somatic mutations per sample. Yellow bars indicate the number of single nucleotide variants and blue bars indicate the number of indels. (Lower panel) Percentage of recurrently mutated genes (genes mutated in more than 3 patients with a mutation rate of > 10 mutation/Mb. Main panels show mutation type of these 11 genes, history of alcohol and smoking, risk SNPs, and tumor depth. Right, bar-graph shows non-silent mutations for each gene.

First, tumor and paired healthy DNAs from 16 Japanese esophageal squamous cell neoplasm (ESCN) patients were subjected to WES. The mean read depth was 125, and 98.7% of target bases were covered by > 10 independent readings (Supplementary Tables 2 and 3). A total of 5,280 somatic events, including 4,084 single-nucleotide substitutions and 1,196 short insertions and deletions (indels), were identified. The mean number of somatic mutations was 179 (range, 34–1428) per sample or 2.38 (range, 0.45–19.4) per megabase across the target exome sequences (Figure 1, Supplementary Tables 2 and 3). Similar to previous findings for many cancer types, the predominant substitution across all ESCN samples was C to T involving the CpG dinucleotide (Supplementary Figure 3A) [12]. The 5,280 somatic mutations identified by WES of the 16 ESCN samples contained 1,328 non-synonymous somatic mutations and indels in 1,182 genes (Supplementary Table 4). Among all 1,182 genes, we identified 89 that had non-synonymous somatic mutations and indels in at least two patients (Supplementary Tables 4 and 5). We found 11 genes that were mutated in more than 3 patients and had a mutation rate of > 10 mutation/Megabase (Mb) (Figure 1). There was no significant difference in the non-synonymous somatic mutation rates of IN and SCC samples (*p* = 0.55); however, there was a significant difference in the CNVs (amplifications and deletions) of these samples (*p* = 0.03). In addition, we searched for mutation signatures in the exome-sequencing data. We found 6 samples that were strongly associated with signature 4 (Supplementary Figure 3) and these were found to belong to heavy smokers; signature 4 is associated with smoking (https://cancer.sanger.ac.uk/cosmic/signatures).

### Detection of non-synonymous somatic mutations in the replication stage

**Figure 2:**
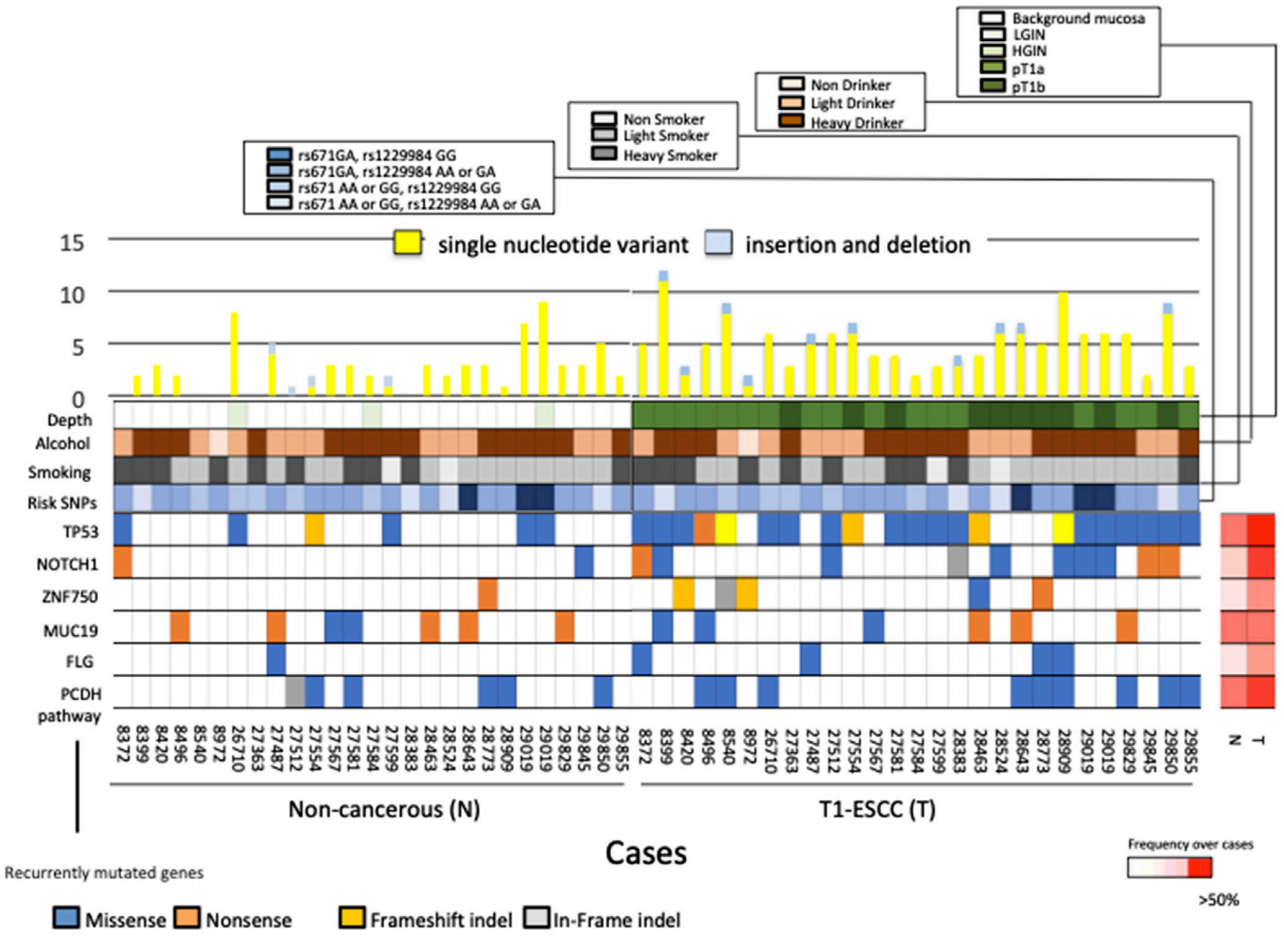
Mutational landscapes in carcinoma tissues and non-cancerous tissues of 26 ESCC patients. (Upper panel) The number of somatic mutations per sample. Yellow bars indicate the number of single nucleotide variants and blue bars indicate the number of indels. (Lower panel) Genes were mutated in > 3 patients with a mutation rate of > 10 mutation/Mb. The panel shows mutated type of these 6 genes, history of alcohol and smoking, risk SNPs, and tumor depth. The right panel shows non-cancerous tissues information, and left panel shows cancerous tissues information. The heat map on the right indicates the frequency of each mutated gene in non-cancerous tissue and ESCC over all cases.

We performed a targeted panel using 89 genes identified in the screening stage and 39 genes associated with the development of ESCC as found in previous studies [7]. Then, SCC tissues, paired non-cancerous tissues, and normal DNA from 26 Japanese ESCC patients were subjected to the targeted panel. We found 5 genes that were mutated in more than 3 patients and had a mutation rate of > 10 mutation/Mb. The most frequently mutated gene in T1 ESCCs was *TP53* (mutated in 76.9% of our cohort), followed by *NOTCH1* (34.6%), *MUC19* (23.1%), *ZNF750* (19.2%), and *FLG* (15.3%) (Figure 2, Supplementary Table 6). The mutation rates of each representative ESCC driver gene identified in our study during the replication stage were compared with the advanced-cancer data, which comprise Sawada’s advanced-ESCC somatic mutation data [6] (Supplementary Figure 4A) and Chen’s advanced-ESCC somatic mutation data [16]. (Supplementary Figure 4B). We found that the frequency of mutations in *MLL2* and *TP53*, observed in advanced-ESCC patients [6, 16], was significantly higher than that in T1 ESCC patients. However, one HGIN and three T1 samples, out of the 16 screening-stage samples, had somatic mutations in *MLL2*. The mutation rates in the non-cancerous samples were as follows: *MUC19* (mutated in 26.9% of our cohort), followed by *TP53* (19.2%), *EGFR* (7.7%), *FAT1* (7.7%), *MUC16* (7.7%), and *NOTCH1* (7.7%) (Supplementary Figure 4C, Supplementary Table 7). The frequency of mutations in *TP53* and *NOTCH1* was higher in the cancerous samples than in the non-cancerous samples (*p* < 0.01, *p* = 0.038, respectively).

### Analysis of copy-number variants at the replication stage

**Figure 3:**
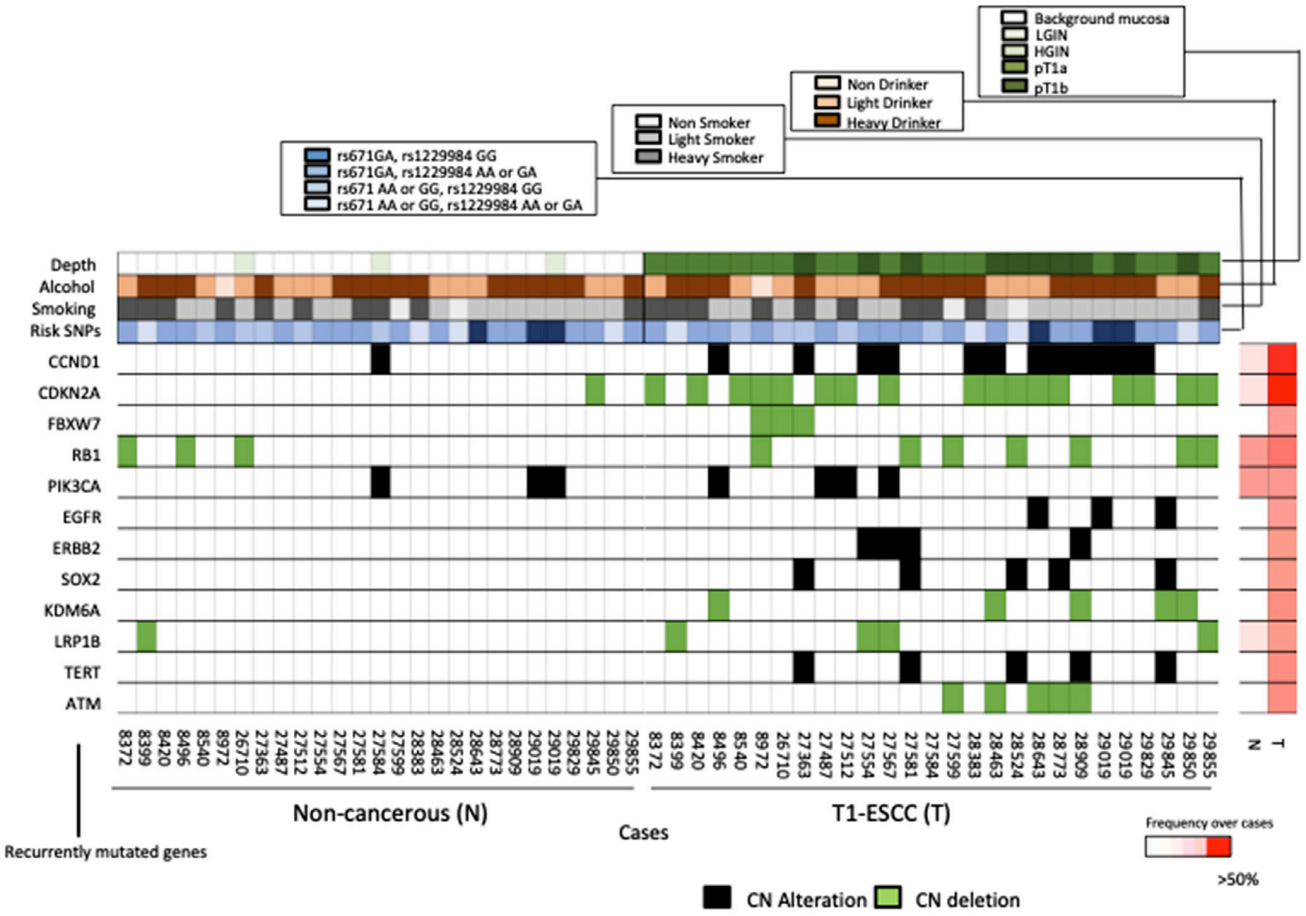
Comparison of the key alterations in non-cancerous tissues and ESCCs. Plots showing the amplification of oncogenes (red) and deletion of tumor suppressor genes (blue) in non-cancerous tissues (left), and ESCCs (right). The heat map on the right indicates the frequency of each mutated gene in non-cancerous tissue and ESCC over all cases.

We analyzed 26 paired ESCC and non-cancerous samples for the presence of recurrent focal copy-number variants (CNVs) via SureCall Copy Number Methods. We compared the CNVs in ESCC driver genes identified in our study with those identified in previous reports [7, 16]. The genes most frequently affected in cancerous areas were *CDKN2A*/*2B* (deleted in 61.5%) and *CCND1* (amplified in 42.3%), followed by *ATM* (deletion 19.3%), *TERT* (amplified in 19.3%), *SOX2* (amplified in 19.3%), *KDM6A* (deletion 19.3%), *LRP1B* (deleted in 15.3%), *ERBB2* (amplified in 15.3%), *PIK3CA* (amplified 15.3%), and *EGFR* (amplified in 11.5%) (Figure 3). We identified the following CNVs: *RB1* deletions and *PIK3CA* amplifications in 3 non-cancerous tissues; and *CCND1* amplification and *CDKN2A* and *LRP1B* deletion in 1 non-cancerous sample (Figure 3). The CNV rates of each representative ESCC driver gene identified in our study during the replication stage were compared with the advanced-cancer data, which comprise Sawada’s advanced-ESCC somatic mutation data [6] (Supplementary Figure 4A) and Chen’s advanced-ESCC somatic mutation data [16]. (Supplementary Figure 4B). However, the CNV rates of some genes (*PIK3CA* and *SOX2*) were significantly different between T1 ESCC and Chen’s advanced-ESCC data [16]. There was no difference between T1 ESCC and advanced-ESCC data (Supplementary Figure 4C). Furthermore, upon gathering the results of the comparison between T1 ESCC and non-cancerous tissues for CNVs, we determined that the number of CNVs in T1 ESCC areas was significantly higher than that in non-cancerous areas (*p* < 0.001) (Supplementary Figure 5). Moreover, the frequencies of *CCND1* amplification and *CDKN2A* deletion in T1 ESCC areas were significantly higher than those in non-cancerous areas (*p* < 0.001 for both) (Supplementary Figure 4C). These results suggested that CNVs occurred in the early stages of carcinogenesis and that *CCND1* amplification and *CDKN2A* deletion played important roles in the development of carcinogenesis in the early stage of ESCC.

### Differences in the mutation allele frequency of *MUC19* and *TP53* in cancerous and non-cancerous states

**Figure 4:**
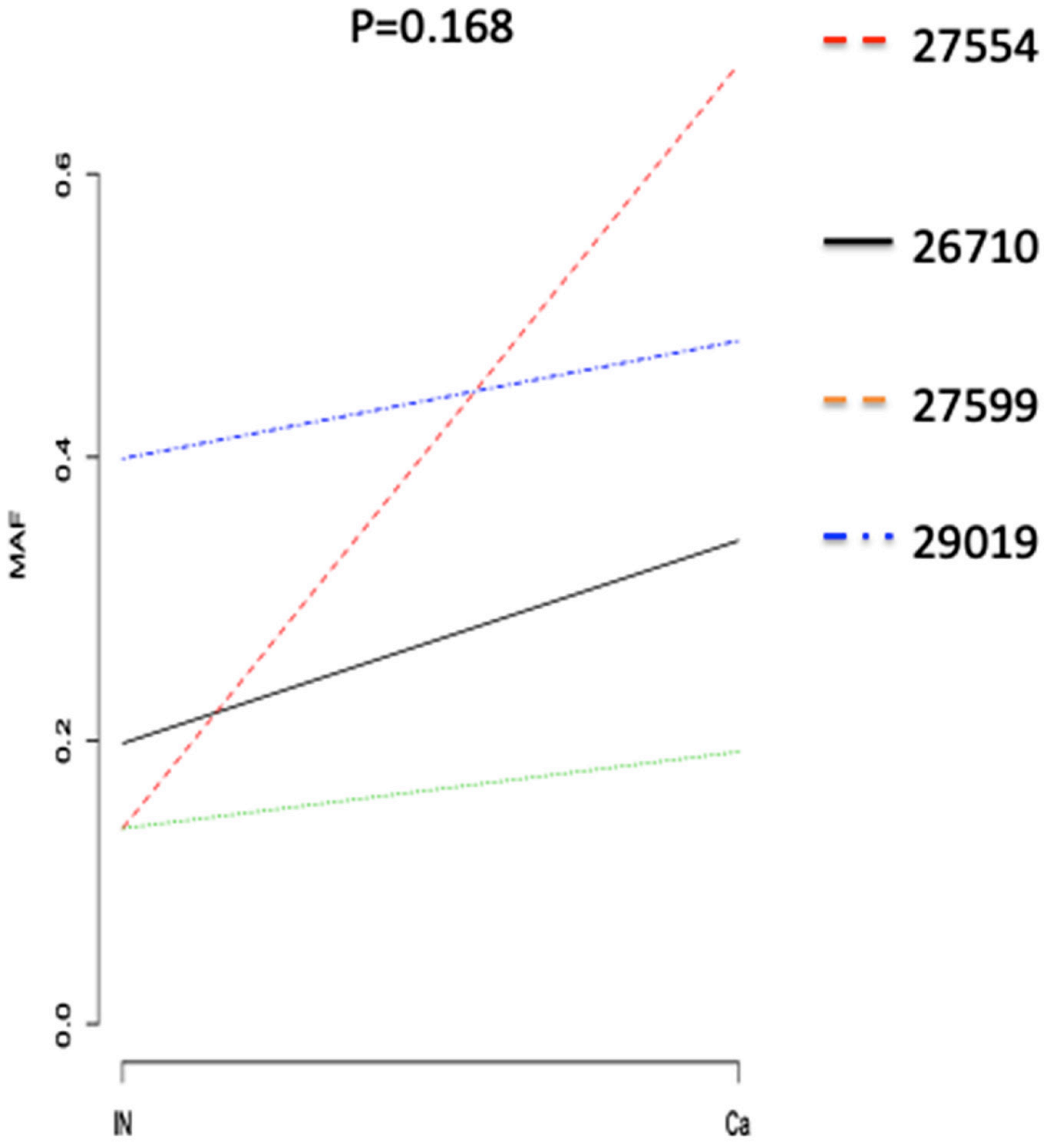
Comparison of *TP53* mutation frequencies in normal mucosa and T1 ESCC areas. Plots showing the amplification of oncogenes (red) and deletion of tumor suppressor genes (blue) in non-cancerous tissues (left), and ESCCs (right). The heat map on the right indicates the frequency of each mutated gene in non-cancerous tissue and ESCC over all cases.

There was no trend in the change in the mutation allele frequency (MAF) of *MUC19* during the transition from a non-cancerous to a cancerous state. Conversely, the MAFs of *TP53* in cancerous areas tended to be higher than those in non-cancerous areas (*p* = 0.17) (Figure 4). Moreover, in one patient, we observed a trend in the MAF of *TP53* in non-cancerous, HGIN, T1a, and T1b areas. This patient’s T1a and T1b areas showed a homozygous mutation, while non-cancerous and HGIN areas showed heterozygous mutations. Tumor growth progression and increases in MAF showed a positive correlation. These results seemed to support our two-hit hypothesis regarding the tumor-suppressor gene *TP53*.

## DISCUSSION

Our study performed the genetic profiling of early-stage ESCC arising from non-cancerous mucosa using early-stage cancer samples. Esophageal normal mucosa in the pre-developmental stages of esophageal neoplasm exhibited known somatic mutations. Katada et al. [15] reported that non-cancerous esophageal mucosa with multiple lugol-voiding lesions had low frequencies of *TP53* mutations. In addition, some papers reported that the pathological transitions from dysplasia to ESCC occur via the accumulation of genetic changes [14, 16]. These previous papers found that mutations and CNVs of dysplasia were almost the same as those of cancer; however, the samples evaluated in their analyses were advanced-stage ESCC cases. Therefore, it is critical to validate these genetic evolutions in early-stage ESCC samples.

In our study, we characterized alterations associated with the transformation to ESCC by comparing the genetic profiles of non-cancerous esophageal tissue and early stage ESCC. We found that somatic mutations in *TP53* and *NOTCH1*, *CDKN2A* deletions, and *CCND1* amplifications play critical roles in the development of ESCC.

The Notch pathway is associated with neoplastic progression [17], and NOTCH1 signaling is growth-repressive [18, 19]. For example, functional studies have shown that NOTCH1 family members suppress proliferation and promote the differentiation of keratinocytes, cells that populate the normal squamous epithelial lining [20, 21]. Moreover, loss of epidermal NOTCH1 promotes skin tumorigenesis by impacting the stromal microenvironment [22]. In ESCC, NOTCH1 plays a tumor-suppressive role during ESCC development [23]. Components of the NOTCH signaling pathway have been reported to interact with TP53 [24, 25]. However, mutations in *TP53* and *NOTCH1* were not mutually exclusive in the esophageal tumors that we evaluated.

Overexpression and somatic mutations of CCND1 often contribute to transformation; they can either directly or indirectly promote constitutive cyclin D1 nuclear localization, which is a critical oncogenic event [26]. In ESCC, CCND1 amplification or overexpression is also significantly correlated with lymph node metastasis [27]. In our data, the frequency of CNVs in early stage ESCC was similar to that in advanced-stage ESCC. Therefore, we hypothesize that *CCND1* alterations occur in the early stages of carcinogenesis.

We also compared the data on somatic mutations and CNVs in early stage ESCC samples with the previously reported advanced stage ESCC data [7, 16]. CNVs of some genes were different between our early stage ESCC analysis and the advanced stage ESCC data [16], and this inconsistency may be partly because the method for CNV analysis in this study was different from that of Chen et al.

Finally, we focused on the change in the frequency of *TP53* mutations as a result of cancer progression. The frequency of *TP53* mutations in each early stage ESCC was higher than that in inflamed esophageal mucosa, which correlated with the transformation of inflamed esophageal mucosa to ESCC. Our data illustrate that decreased TP53 activity was characteristic of dysplastic mucosal tissue, whereas full TP53 inactivation was associated with early-stage ESCC. Previous studies indicated that TP53 expression was altered in some patients with dysplasia [11, 28] and that mutations in *TP53* arose early during ESCC development [29, 30]. On the contrary, Chen et al. characterized the genomic alterations in ESCC precursor lesions, delineated clonal evolution in ESCC development, and suggested that the complete inactivation of TP53 is essential for the development of ESCC [16].

Our study had several limitations. The most significant limitation was that the sample size was less than 50, which limits the possibility to detect all possible changes in cancer driver genes. We expect that a few changes went undetected. Thus, further studies for clarifying the initial-stage mechanism for carcinogenesis using a large number of Japanese early stage ESCC samples are warranted. Secondly, not all samples were evaluated with whole genome/exome sequencing. We performed exome sequencing for only the early stage ESCC samples in which we had identified high mutation frequencies. In the future, more samples should be included in this analysis. Moreover, the functional effects of these mutations should be determined. Lastly, our study did not examine a sufficient number of distinct genomic regions in cancerous and non-cancerous tissues. However, Chen et al. applied multi-region whole-exome sequencing to characterize the genomic landscape in ESCC precursor lesions [16].

In conclusion, our study sheds light on the genetic changes that contribute to the development of ESCC. In recent years, the genome profiles of various cancer types have been studied in detail, and much effort has been put into elucidating and identifying the specific changes that accrue during the development of various cancers. We believe that our study will be of great help in unraveling the mechanisms underlying ESCC development.

## MATERIALS AND METHODS

### Sample collection

This research was approved by the Hiroshima University Human Genome Ethical Committee. Informed consent for whole-genome analysis was obtained from all patients. All samples were obtained with the approval of the ethics committee of Hiroshima University Hospital. A total of 117 distinct samples from 42 individuals with early-stage ESCC and precursor lesions were used in this study. Clinicopathological characteristics of individuals included in this study are shown in Supplementary Table 1. Samples were collected from patients undergoing endoscopic resection at the Hiroshima University Hospital from December 2012 to December 2015. We analyzed 16 fresh-frozen samples (6 high-grade INs, 10 T1 stage ESCC) in the screening stage as well as cancerous and non-cancerous tissues from 26 patients in the replication stage. Fresh-frozen samples were collected from the cancerous areas in ESCN patients by biopsy. Cancerous and adjacent normal areas of formalin-fixed paraffin embedded (FFPE) specimens isolated using the Leica LMD 6000 laser microdissection system were identified by a pathologist (Supplementary Figure 2). DNA was extracted from frozen samples, FFPE specimens, and blood leukocyte samples using the AllPrep DNA/RNA Micro Kit, GeneRead DNA FFPE Kit, and QIAamp DNA Blood Midi/Maxi kit, respectively (Qiagen, Hilden, Germany). DNA volumes were measured by Qubit HS (Qiagen). The quantity and quality of the FFPE-derived DNA was assessed by calculating normalized DNA integrity scores (ΔΔCq) using quantitative PCR with the Agilent NGS FFPE QC Kit (Agilent Technologies, San Diego, CA, USA).

### Whole exome sequencing and targeted sequencing

For WES, sequencing libraries were prepared from 200 ng of genomic DNA using the Agilent SureSelect Human All ExonV5 kit (Agilent Technologies) by following the manufacturer’s instructions; the library was sequenced using an Illumina Hiseq 2500 platform. A total of 128 genes were selected based on results obtained during the screening stage. A total of 50 ng of genomic DNA per sample was used as input. Sequencing libraries were generated using the Agilent Haloplex HS Custom Kit (Agilent Technologies) by following the manufacturer’s instructions. Quality control of the pooled libraries was checked using the 2200 Tape Station instrument of the High Sensitivity D1000 ScreenTape System (Agilent Technologies).

### Detection of somatic mutations in cancerous and non-cancerous tissues

The analysis of the screening stage used the CLC Genomics Workbench (CLC bio, Aarhus, Denmark). Sequences were aligned to the hg19/GRCh37 reference sequence and analyzed using Map Reads to Reference program with default parameters to generate a binary sequence alignment map (BAM) file. The aligned BAM file was sorted and merged using Merge Read Mappings program. PCR duplicates were removed using Removed Duplicate Mapped Reads program, and local realignment was performed using Local Realignment program to improve mapping quality prior to screening for variants. To identify variants, the low-frequency variant detection Program (http://resources.qiagenbioinformatics.com/manuals/clcgenomicsworkbench/current/User_Manual.pdf, p653-6) was used. We set the following criteria for identification of reliable somatic SNVs or INDELs: (1) reads covering the mutated sites should number more than 30, with at least 3 reads harboring the mutations; (2) allele frequency of mutant reads should be more than 10% in all reads; (3) reads covering the mutated sites in the corresponding normal control should number more than 30, with at most 1 read harboring the mutations; (4) variants for which the minimum of the fraction of ‘countable’ forward reads and ‘countable’ reverse reads carrying the variant to all ‘countable’ reads carrying the variant is less than 0.2 were excluded; and (5) mutations listed in dbSNP 137, the HapMap database, or the 1000 Genomes Project were removed.

In the replication stage, the paired-end clean reads were mapped to the reference genome (UCSC Human Genome Reference hg19), and the mutation detection process was performed using the SureCall System 4.0.1.46 Haloplex Default Analysis Method (Agilent Technologies). Variant calling was performed by SNPPET in the SureCall System 4.0.1.46, and the criteria for identification of reliable somatic SNVs or Indels were set as follows: 1) variant score threshold of 0.3, minimum quality for base 30; 2) variant call quality threshold of 100; 3) minimum allele frequency of 0.1; 4) minimum number of reads supporting variant allele of 10; 5) minimum number of read pairs per barcode of 2; and 6) removal of mutations listed in dbSNP 137, the HapMap database, or the 1000 Genomes Project.

### Detection of copy-number variants in cancerous and non-cancerous tissues

We performed copy-number analysis using the SureCall System 4.0.1.46 Haloplex Default Copy Number Method (Agilent Technologies) to analyze somatic CNVs and loss of heterozygosity in screening and replication stages. For somatic CNVs, coverage in the tumor genome was normalized to coverage for the same region in the matched normal genome. A hidden Markov model was used to calculate CNVs in the genome. CNV analysis predicts amplifications and deletions on the log ratio of the normalized sample/reference read depth. The parameters used to determine the criteria of the CNV identification were as follows: adaptive interval window size: 200; minimum read depth of reference: 10; log ratio threshold for amplification: 3; and log ratio threshold for deletion: -1 in all stages.

### Comparison between early-stage ESCC and advanced ESCC

The T1 data from 26 ESCC patients with mutations in each representative ESCC driver gene were collected only during the replication stage. The advanced-cancer data indicated the somatic mutations in all exons in Sawada’s advanced-ESCC data [6] and the Chinese advanced-ESCC data [16]. Besides, The T1 data from 26 ESCC patients with CNVs in each representative ESCC driver gene were collected only during the replication stage. The advanced-cancer data indicated the CNVs in all exons in Sawada’s [7] advanced-ESCC data and Chen et al.’s [16] advanced stage ESCC data.

### Analysis of mutation signature

We made a Python script to extract each Excel sheet into a separate file containing four columns (CHROM, POS, REF, ALT) and uploaded the files to the http://bioinfo.ciberehd.org:3838/MuSiCa/ web site as TSV files with exome sequencing against the hg19 reference sequence. Information on mutation signatures was obtained from the COSMIC website (https://cancer.sanger.ac.uk/cosmic/signatures).

### Statistical analysis

We compared our patients’ data with that of the results of somatic mutations in all exons in the Japanese advanced-ESCC data [7] via the chi-square and Fisher’s exact tests in order to observe the differences in the frequency of patients with somatic mutations and CNVs in each gene. Several mutations were compared between cancerous and non-cancerous areas using the Kruskal-Wallis test. Changes in MAFs in *TP53* were shown via paired *T*-test. All statistical analyses were performed using R version 3.3.1(https://cran.r-project.org/), and *p*-values less than 0.05 were considered significant.

## SUPPLEMENTARY MATERIALS







## Abbreviations

SCC: squamous cell carcinoma
ESCC: esophageal squamous cell carcinoma
ESCN: esophageal squamous cell neoplasm
IN: intraepithelial neoplasia
HGIN: high-grade IN
WES: whole-exome sequencing
MAF: mutation allele frequency
CNV: copy-number variants
