# Supplementary material for: Genomic characterization of early-stage esophageal squamous cell carcinoma in a Japanese population
Source: Oncotarget. 2019 Jun 25;10(41):4139–48. doi: 10.18632/oncotarget.27014 (PMC6609253; doi:10.18632/oncotarget.27014)
Supplement: Supplementary file 1 [file oncotarget-10-4139-s001.pdf]

# Genomic characterization of early-stage esophageal squamous cell carcinoma in a Japanese population

## SUPPLEMENTARY MATERIALS

### Supplementary Table 1: Sample characteristics of 42 superficial esophageal squamous cell neoplasm patients

| ESCN[a](HGIN/T1a/T1b) | 6/24/12 |
|---|---|
| Gender (Male/Female) | 39/3 |
| Age | 68.4 ± 7.0 (53–83) |
| Drinking History (none/light/heavy)[b] | 1/16/25 |
| smoking (none/light/heavy)[c] | 3/21/18 |
| rs671 at ALDH2 (GA/GG+AA) | 29/13 |
| rs1229984 at ADH1B (GG/GA+AA) | 5/37 |

[b]Heavy alcohol drinker was defined as alcohol consumption of more than 60 g/day based on the ethanol conversion data.
[c]Heavy smoker was defined as tobacco consumption more than the Brickman index 1000.
[a]ESCN; Esophageal squamous cell neoplasm.

### Supplementary Table 2: Average read depth for exome sequencing in 16 ESCC samples

| | Average Coverage | |
|---|---|---|
| ID | Tumor | Blood |
| 26723 | 186.6 | 105.4 |
| 11515 | 139.6 | 101.7 |
| 22780 | 127.5 | 107 |
| 23727 | 74.2 | 75.4 |
| 25178 | 142 | 128.8 |
| 25287 | 118.5 | 131.8 |
| 11489 | 166.4 | 122.4 |
| 22739 | 159.3 | 68.8 |
| 23658 | 132 | 110.2 |
| 24311 | 140.4 | 87.5 |
| 24411 | 150.1 | 84.7 |
| 25074 | 144.7 | 85.2 |
| 25243 | 141.4 | 58.9 |
| 22833 | 120.7 | 91.8 |
| 26694 | 187.6 | 123.4 |
| 25089 | 164.1 | 143 |

Average read Depth for exome sequencing in 16 esophageal squamous cell carcinoma and matched blood leukemia samples. Results were analyzed using exome sequencing.

**Supplementary Table 3: The frequencies covered by more than 10 sequences in screening stage samples**

| ID | The frequency of target bases covered were > 10 (%) | |
|---|---|---|
| | Tumor | Blood |
| 26723 | 98.39 | 99.1 |
| 11515 | 98.88 | 98.51 |
| 22780 | 98.78 | 98.52 |
| 23727 | 84.74 | 97.61 |
| 25178 | 98.84 | 98.74 |
| 25287 | 98.65 | 98.78 |
| 11489 | 98.99 | 98.77 |
| 22739 | 98.95 | 97.03 |
| 23658 | 98.7 | 98.58 |
| 24311 | 98.87 | 98.03 |
| 24411 | 98.85 | 97.92 |
| 25074 | 98.78 | 98.16 |
| 25243 | 98.82 | 81.75 |
| 22833 | 98.61 | 98.23 |
| 26694 | 99.04 | 98.52 |
| 25089 | 98.71 | 98.99 |

Target bases were covered by more than 10 sequences during exome sequencing of 16 esophageal squamous cell carcinoma and matched blood leukemia samples. Results were analyzed by exome sequencing.

**Supplementary Table 4: Somatic mutations in 16 early-stage Esophageal neoplasms using exome sequecncing.** See Supplementary_Table_4

**Supplementary Table 5: Taget panel mounted 128 genes used in the replication stage**

| | | | | | |
|---|---|---|---|---|---|
| *AJUBA* | *FAT2* | *KRAS* | *NOTHC3* | *SLC6A6* | *ZNF323* |
| *BAP1* | *FAT3* | *LAMB1* | *NUTM2G* | *SLITRK3* | *ZNF496* |
| *BMPR1B* | *FAT4* | *LIMCH1* | *OBSCN* | *SOX1* | *ZNF750* |
| *C17orf28* | *FBLN2* | *LRP1B* | *OR2T3* | *SOX2* | |
| *CABS1* | *FBRS* | *LRP5* | *PALB2* | *STAG2* | |
| *CCND1* | *FLG* | *LRPAP1* | *PCDH8* | *SYBU* | |
| *CDH23* | *FOXA1* | *LYST* | *PCDHA1* | *TDRD1* | |
| *CDKN2B* | *FOXC2* | *MAGEE1* | *PCDHA13* | *TERT* | |
| *CES1* | *FRYL* | *MET* | *PCDHA3* | *TET1* | |
| *COL6A6* | *FSCB* | *MGA* | *PCLO* | *TET2* | |
| *CREBBP* | *GPR98* | *MIA3* | *PIK3CA* | *TGFBR2* | |
| *CSMD1* | *GTPBP8* | *MLL1* | *PKHD1L1* | *THSD7B* | |
| *CSMD3* | *HBG2* | *MLL2* | *PRDM9* | *TLR1* | |
| *CUL9* | *HERC6* | *MSTO1* | *PRSS21* | *TP53* | |
| *DCSTAMP* | *HKR1* | *MTERFD1* | *PTEN* | *TTN* | |
| *DHX9* | *HPSE* | *MUC16* | *PTPN13* | *UBR2* | |
| *DNAH14* | *HUWE1* | *MUC19* | *PTPRD* | *ULK4* | |
| *DNAH3* | *HYDIN* | *MYO18A* | *RAG1* | *UNC13C* | |
| *DST* | *INTU* | *NBEA* | *RB* | *VPS35* | |
| *EGFR* | *IQCB1* | *NCKAP5* | *RELN* | *WAPAL* | |
| *EP300* | *IQSEC1* | *NCOR1* | *RFX6* | *WNK2* | |
| *ERBB2* | *ITGA10* | *NFE2L2* | *SACS* | *YAP1* | |
| *F5* | *KDM6A* | *NLRP14* | *SDAD1* | *ZAN* | |
| *FAM135B* | *KIR3DL1* | *NOTCH1* | *SIDT1* | *ZCCHC2* | |
| *FAT1* | *KLF5* | *NOTCH2* | *SLC35E1* | *ZFHX4* | |

Target panel-mounted 128 genes used in the replication stage. These 128 genes included 89 genes that had non-synonymous somatic mutations and copy-number alterations in at least two patients, and 39 genes were associated with the development of Japanese esophageal squamous cell carcinoma.

**Supplementary Table 6: Somatic mutations in 26 cancerous tissues in early-stage Esophageal squamous cell carcinoma using target sequecncing.** See Supplementary_Table_6

## Supplementary Table 7: Somatic mutations in 26 non-cancerous tissues in early-stage esophageal squamous cell carcinoma using target sequencing

| Sample ID | Gene | Type | Zygosity | Chr. | Position | Ref | Alt | MAF | Count | Coverage | Codon | AA | AA |
|---|---|---|---|---|---|---|---|---|---|---|---|---|---|
| 8372 | NOTCH2 | SNP | HET | 1 | 120478094 | C | A | 0.308 | 20 | 65 | MISSENSE | gGt/gTt | G1219V |
| 8372 | TTN | SNP | HOM | 2 | 179449478 | G | T | 0.205 | 9 | 44 | NONSENSE | taC/taA | Y12565* |
| 8372 | FAM135B | SNP | HOM | 8 | 139164848 | G | C | 0.131 | 49 | 374 | MISSENSE | Caa/Gaa | Q624E |
| 8372 | NOTCH1 | SNP | HOM | 9 | 139399229 | C | T | 0.219 | 33 | 151 | NONSENSE | tgG/tgA | W1638* |
| 8372 | TP53 | SNP | HET | 17 | 7578478 | G | C | 0.511 | 95 | 186 | MISSENSE | cCc/cGc | P112R |
| 8399 | ULK4 | SNP | HOM | 3 | 41996187 | C | A | 0.133 | 4 | 30 | MISSENSE | cGa/cTa | R22L |
| 8399 | EGFR | SNP | HOM | 7 | 55233129 | G | T | 0.105 | 4 | 38 | NONSENSE | Gga/Tga | G627* |
| 8420 | CUL9 | SNP | HOM | 6 | 43170869 | G | T | 0.121 | 4 | 33 | MISSENSE | aGc/aTc | S1259I |
| 8420 | EGFR | SNP | HOM | 7 | 55260487 | C | A | 0.111 | 4 | 36 | NONSENSE | tCa/tAa | S885* |
| 8420 | PCLO | SNP | HOM | 7 | 82545627 | G | T | 0.125 | 4 | 32 | MISSENSE | tCc/tAc | S3892Y |
| 8496 | MUC19 | SNP | HOM | 12 | 40879080 | C | T | 0.216 | 66 | 306 | MISSENSE | gCa/gTa | A4109V |
| 8496 | MUC19 | SNP | HOM | 12 | 40879082 | A | T | 0.216 | 66 | 306 | NONSENSE | Aga/Tga | R4110* |
| 26710 | MIA3 | SNP | HOM | 1 | 222838725 | C | T | 0.185 | 22 | 119 | MISSENSE | Ctc/Ttc | L1830F |
| 26710 | MIA3 | SNP | HOM | 1 | 222838734 | C | T | 0.212 | 21 | 99 | MISSENSE | Cgg/Tgg | R1833W |
| 26710 | TTN | SNP | HOM | 2 | 179632555 | G | T | 0.121 | 4 | 33 | MISSENSE | aaC/aaA | N3088K |
| 26710 | ZAN | SNP | HOM | 7 | 100374121 | C | T | 0.207 | 12 | 58 | MISSENSE | gCg/gTg | A2139V |
| 26710 | CLUH | SNP | HET | 17 | 2594021 | A | T | 0.264 | 42 | 159 | MISSENSE | cTc/cAc | L1266H |
| 26710 | TP53 | SNP | HOM | 17 | 7577586 | A | C | 0.198 | 118 | 595 | MISSENSE | aTc/aGc | I100S |
| 27487 | AKR7L | SNP | HOM | 1 | 19600376 | T | G | 0.119 | 5 | 42 | MISSENSE | Atg/Ctg | M65L |
| 27487 | FLG | SNP | HOM | 1 | 152280857 | T | C | 0.107 | 6 | 56 | MISSENSE | Agc/Ggc | S2169G |
| 27487 | MUC19 | SNP | HOM | 12 | 40879080 | C | T | 0.201 | 40 | 199 | MISSENSE | gCa/gTa | A4109V |
| 27487 | MUC19 | SNP | HOM | 12 | 40879082 | A | T | 0.201 | 40 | 199 | NONSENSE | Aga/Tga | R4110* |
| 27487 | NBEA | Deletion | HET | 13 | 35516972 | GCCG GGC | G | 0.662 | 45 | 68 | | ccgggc/- | PG6- |
| 27512 | PCDH17 | Deletion | HOM | 13 | 58299434 | GA | G | 0.169 | 11 | 65 | | | |
| 27554 | PCDHB6 | SNP | HOM | 5 | 140532121 | G | T | 0.118 | 4 | 34 | MISSENSE | gaG/gaT | E625D |
| 27554 | TP53 | Insertion | HOM | 17 | 7578394 | T | TGGTG GGGGCA | 0.138 | 34 | 247 | | | |
| 27567 | F5 | SNP | HOM | 1 | 169515742 | C | A | 0.133 | 4 | 30 | MISSENSE | tGt/tTt | C567F |
| 27567 | IQCB1 | SNP | HOM | 3 | 121500705 | G | A | 0.125 | 4 | 32 | MISSENSE | gCg/gTg | A299V |
| 27567 | MUC19 | SNP | HET | 12 | 40879854 | G | T | 0.467 | 28 | 60 | MISSENSE | aGc/aTc | S4367I |
| 27581 | F5 | SNP | HOM | 1 | 169529927 | C | A | 0.105 | 4 | 38 | NONSENSE | Gaa/Taa | E151* |
| 27581 | PCDH18 | SNP | HOM | 4 | 138451277 | C | A | 0.108 | 4 | 37 | MISSENSE | Gtt/Ttt | V656F |
| 27581 | MUC19 | SNP | HOM | 12 | 40879854 | G | T | 1 | 34 | 34 | MISSENSE | aGc/aTc | S4367I |
| 27584 | TTN | SNP | HET | 2 | 179430997 | G | A | 0.357 | 5 | 14 | MISSENSE | aCg/aTg | T17556M |
| 27584 | FAM135B | SNP | HOM | 8 | 139149456 | G | T | 0.105 | 4 | 38 | MISSENSE | Cgt/Agt | R1317S |
| 27596 | CSPG4 | SNP | HOM | 15 | 75982072 | G | A | 0.103 | 6 | 58 | MISSENSE | gCc/gTc | A445V |
| 27596 | CSPG4 | SNP | HOM | 15 | 75982085 | C | T | 0.105 | 6 | 57 | MISSENSE | Gag/Aag | E441K |
| 27599 | TP53 | SNP | HOM | 17 | 7577081 | T | C | 0.138 | 30 | 218 | MISSENSE | gAa/gGa | E127G |
| 28463 | TTN | SNP | HOM | 2 | 179463986 | G | A | 0.129 | 4 | 31 | MISSENSE | aCg/aTg | T16277M |
| 28463 | MUC19 | SNP | HOM | 12 | 40879080 | C | T | 0.153 | 24 | 157 | MISSENSE | gCa/gTa | A4109V |
| 28463 | MUC19 | SNP | HOM | 12 | 40879082 | A | T | 0.152 | 24 | 158 | NONSENSE | Aga/Tga | R4110* |
| 28524 | TTN | SNP | HOM | 2 | 179474027 | C | A | 0.105 | 4 | 38 | MISSENSE | cGa/cTa | R14769L |
| 28524 | CSPG4 | SNP | HOM | 15 | 75982085 | C | T | 0.196 | 10 | 51 | MISSENSE | Gag/Aag | E441K |
| 28643 | MUC19 | SNP | HOM | 12 | 40879080 | C | T | 0.212 | 50 | 236 | MISSENSE | gCa/gTa | A4109V |
| 28643 | MUC19 | SNP | HOM | 12 | 40879082 | A | T | 0.211 | 50 | 237 | NONSENSE | Aga/Tga | R4110* |
| 28643 | HUWE1 | SNP | HOM | X | 53610712 | G | A | 0.121 | 4 | 33 | MISSENSE | Cat/Tat | H1776Y |
| 28773 | PRG4 | SNP | HOM | 1 | 186277277 | G | A | 0.144 | 115 | 801 | MISSENSE | gGg/gAg | G675E |
| 28773 | PCDHB8 | SNP | HET | 5 | 140558212 | A | C | 0.273 | 12 | 44 | MISSENSE | aaA/aaC | K199N |
| 28773 | ZNF750 | SNP | HOM | 17 | 80790256 | A | T | 0.113 | 59 | 520 | NONSENSE | taT/taA | Y25* |
| 28909 | PCDHB2 | SNP | HOM | 5 | 140474669 | G | A | 0.118 | 4 | 34 | MISSENSE | Ggc/Agc | G99S |

| | | | | | | | | | | | | | |
|---|---|---|---|---|---|---|---|---|---|---|---|---|---|
| 29019 | ULK4 | SNP | HOM | 3 | 41942307 | G | T | 0.108 | 4 | 37 | MISSENSE | caC/caA | H399Q |
| 29019 | FAT1 | SNP | HET | 4 | 187532585 | T | C | 0.25 | 11 | 44 | MISSENSE | Ata/Gta | I3270V |
| 29019 | ADGRV1 | SNP | HOM | 5 | 90449044 | C | A | 0.105 | 4 | 38 | MISSENSE | Cct/Act | P6211T |
| 29019 | CSMD3 | SNP | HOM | 8 | 113314108 | C | A | 0.111 | 4 | 36 | MISSENSE | tGc/tTc | C2616F |
| 29019 | SACS | SNP | HOM | 13 | 23912502 | C | A | 0.125 | 4 | 32 | MISSENSE | gGa/gTa | G1691V |
| 29019 | TP53 | SNP | HET | 17 | 7578235 | T | C | 0.399 | 144 | 361 | MISSENSE | tAt/tGt | Y166C |
| 29019 | MUC16 | SNP | HOM | 19 | 9059971 | C | T | 0.174 | 33 | 190 | MISSENSE | Gtt/Att | V9159I |
| 29019_IN | DHX9 | SNP | HET | 1 | 182850535 | G | T | 0.447 | 59 | 132 | MISSENSE | Gta/Tta | V1025L |
| 29019_IN | CAMSAP2 | SNP | HOM | 1 | 200776609 | C | G | 0.179 | 7 | 39 | MISSENSE | Caa/Gaa | Q169E |
| 29019_IN | LRP1B | SNP | HOM | 2 | 141660668 | A | G | 0.183 | 17 | 93 | MISSENSE | gTc/gCc | V1196A |
| 29019_IN | TTN | SNP | HET | 2 | 179659753 | C | T | 0.357 | 96 | 269 | MISSENSE | Ggt/Agt | G381S |
| 29019_IN | BAP1 | SNP | HET | 3 | 52443861 | G | C | 0.618 | 55 | 89 | MISSENSE | Cca/Gca | P12A |
| 29019_IN | PKHD1L1 | SNP | HET | 8 | 110410707 | G | C | 0.253 | 56 | 221 | MISSENSE | ttG/ttC | L314F |
| 29019_IN | NOTCH1 | SNP | HET | 9 | 139399276 | C | A | 0.572 | 115 | 201 | NONSENSE | Gag/Tag | E1623* |
| 29019_IN | TP53 | SNP | HOM | 17 | 7578272 | G | A | 0.762 | 80 | 105 | MISSENSE | Cat/Tat | H154Y |
| 29829 | MUC19 | SNP | HOM | 12 | 40879080 | C | T | 0.125 | 32 | 256 | MISSENSE | gCa/gTa | A4109V |
| 29829 | MUC19 | SNP | HOM | 12 | 40879082 | A | T | 0.125 | 32 | 256 | NONSENSE | Aga/Tga | R4110* |
| 29829 | MUC19 | SNP | HET | 12 | 40879854 | G | T | 0.531 | 34 | 64 | MISSENSE | aGc/aTc | S4367I |
| 29845 | TTN | SNP | HET | 2 | 179441732 | G | T | 0.283 | 15 | 53 | NONSENSE | taC/taA | Y14045* |
| 29845 | FAT1 | SNP | HOM | 4 | 187549851 | C | G | 0.235 | 8 | 34 | MISSENSE | Gtt/Ctt | V1464L |
| 29845 | NOTCH1 | SNP | HET | 9 | 139395021 | C | G | 0.45 | 54 | 120 | MISSENSE | Gca/Cca | A1973P |
| 29850 | FLG-AS1 | SNP | HOM | 1 | 152286367 | C | A | 0.167 | 6 | 36 | MISSENSE | Cca/Aca | P145T |
| 29850 | PRG4 | SNP | HOM | 1 | 186277277 | G | A | 0.143 | 101 | 708 | MISSENSE | gGg/gAg | G675E |
| 29850 | PCDHA8 | SNP | HOM | 5 | 140221012 | C | T | 0.2 | 17 | 85 | MISSENSE | Ccc/Tcc | P36S |
| 29850 | CUL9 | SNP | HOM | 6 | 43184132 | A | C | 0.108 | 8 | 74 | MISSENSE | cAc/cCc | H2058P |
| 29850 | MUC16 | SNP | HOM | 19 | 9018508 | T | G | 0.127 | 8 | 63 | MISSENSE | Aag/Cag | K12556Q |
| 29855 | PCDHA8 | SNP | HOM | 5 | 140221071 | G | T | 0.231 | 15 | 65 | MISSENSE | gaG/gaT | E55D |
| 29855 | APOBEC3B | SNP | HOM | 22 | 39381835 | T | C | 0.138 | 18 | 130 | MISSENSE | Tac/Cac | Y65H |

Non-synonymous somatic mutations in 26 normal mucosae from early-stage esophageal squamous cell carcinoma (ESCC).
Patients identified using target panel mounted with candidate ESCC driver genes.
Chr, Chromosome; Ref, Reference allele; Alt, mutant allele; MAF, mutant allele freguency; Coverage is filter read depth using molecular barcode.

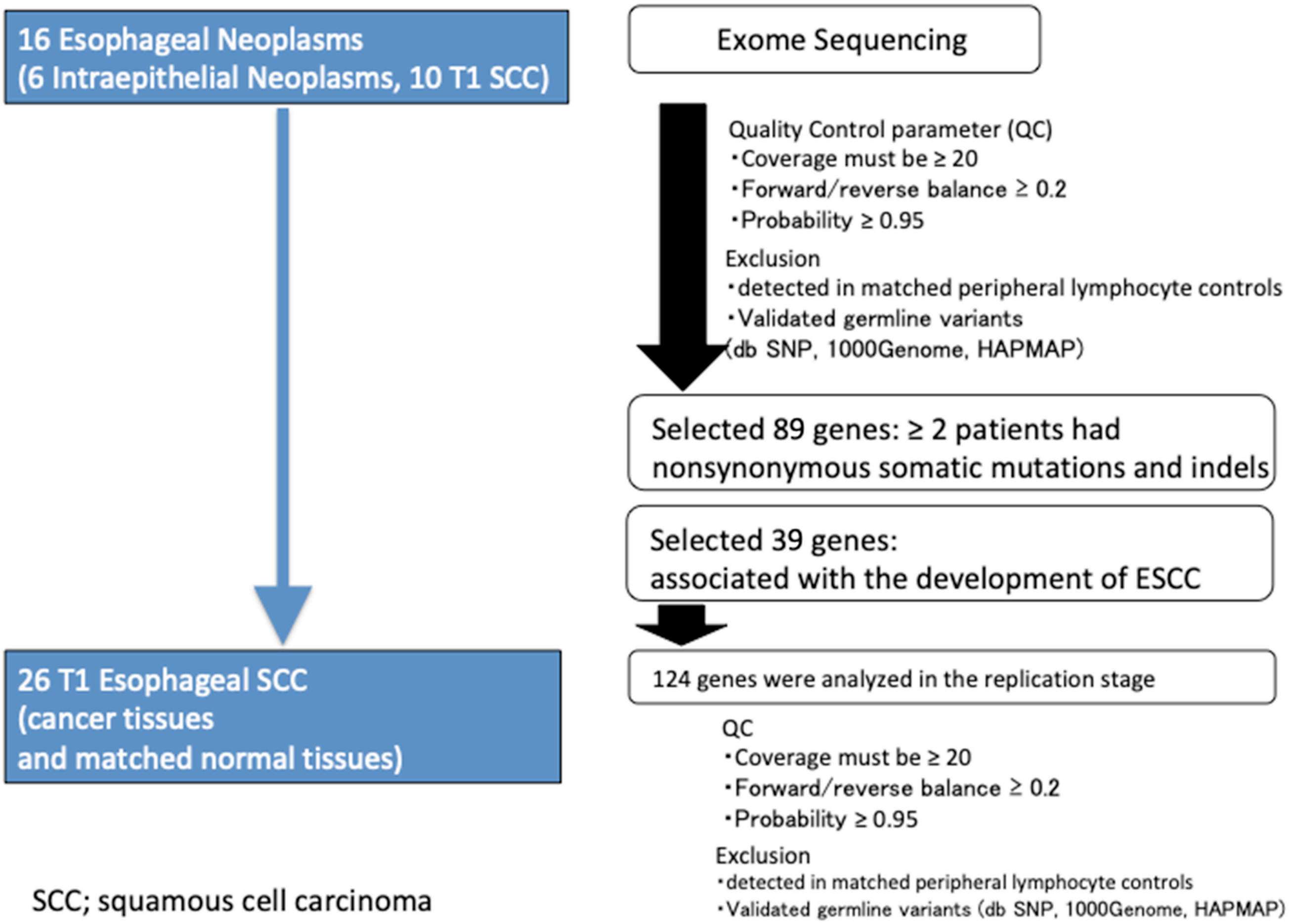


**Supplementary Figure 1: Study design.**

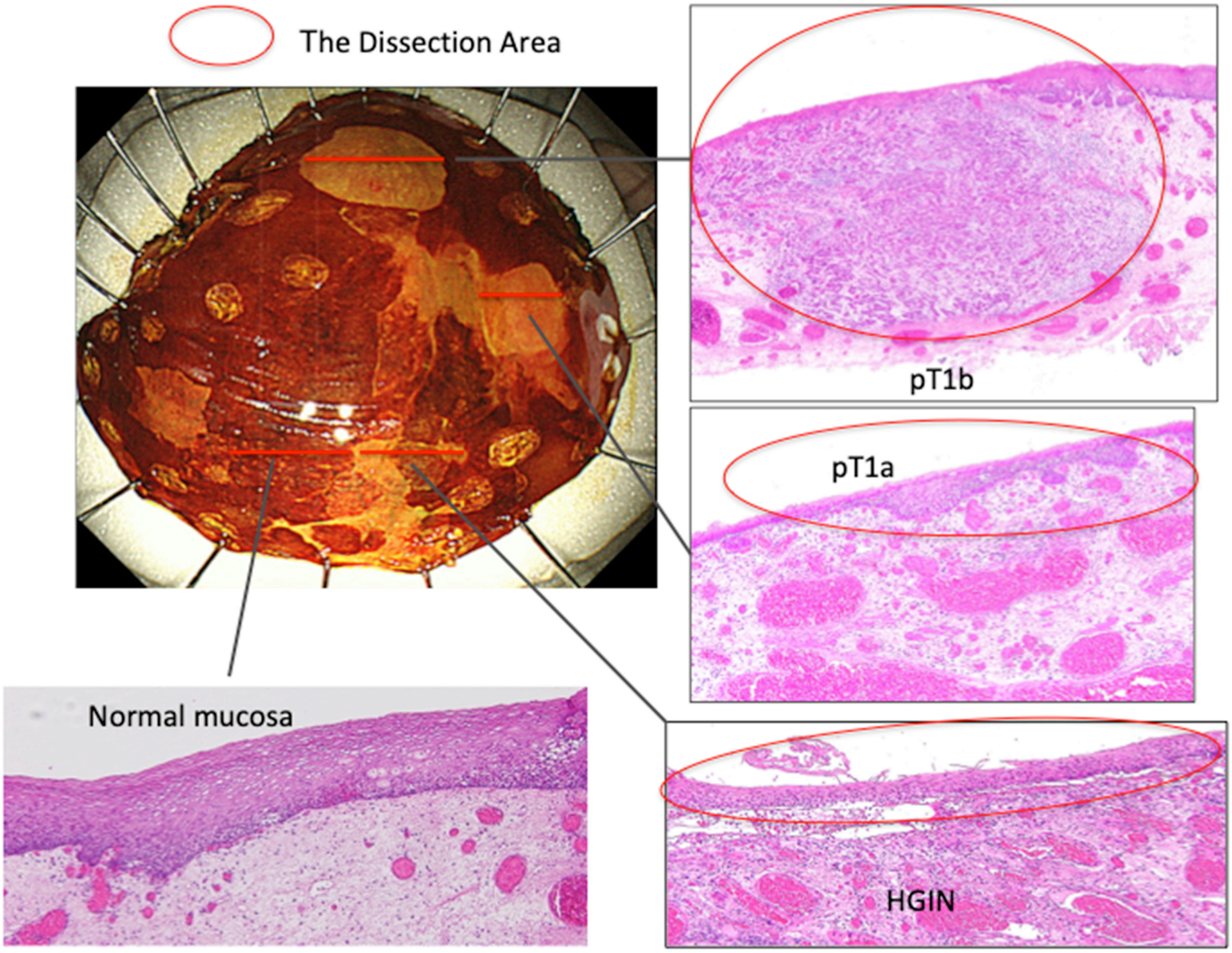


**Supplementary Figure 2: Diagram showing the hematoxylin- and eosin-stained sections of morphologically normal epithelial tissue, low-grade dysplasia (LD), high-grade dysplasia (HD), and invasive esophageal squamous cell carcinoma (ESCC) from the same case.**

A

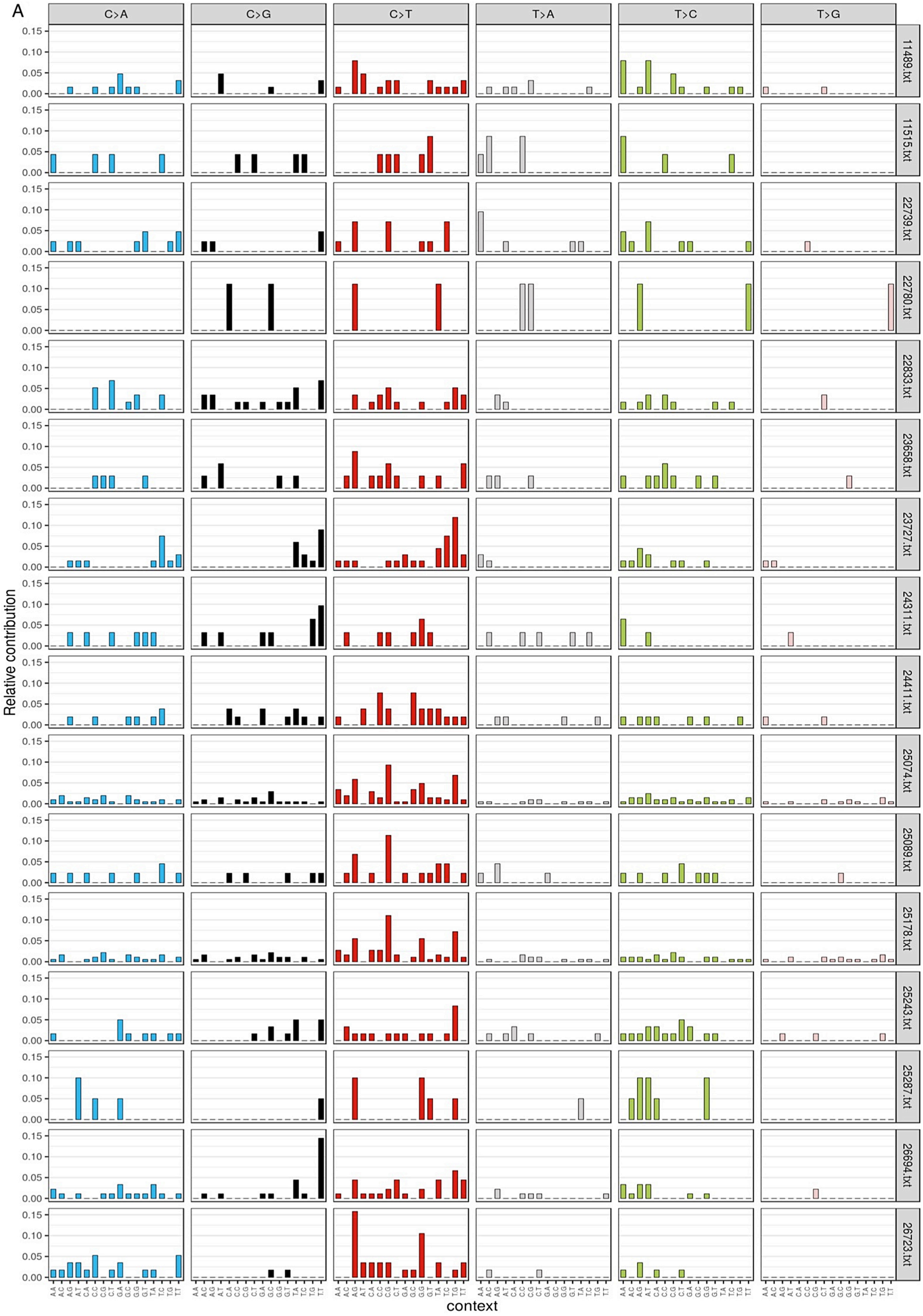
C>A
C>G
C>T
T>A
T>C
T>G
0.15
0.10
0.05
0.00
11489.txt
11515.txt
22739.txt
22780.txt
22833.txt
23658.txt
23727.txt
24311.txt
24411.txt
25074.txt
25089.txt
25178.txt
25243.txt
25287.txt
26694.txt
26723.txt
Relative contribution
A.A A.C A.G A.T C.A C.C C.G C.T G.A G.C G.G G.T T.A T.C T.G T.T
context

B

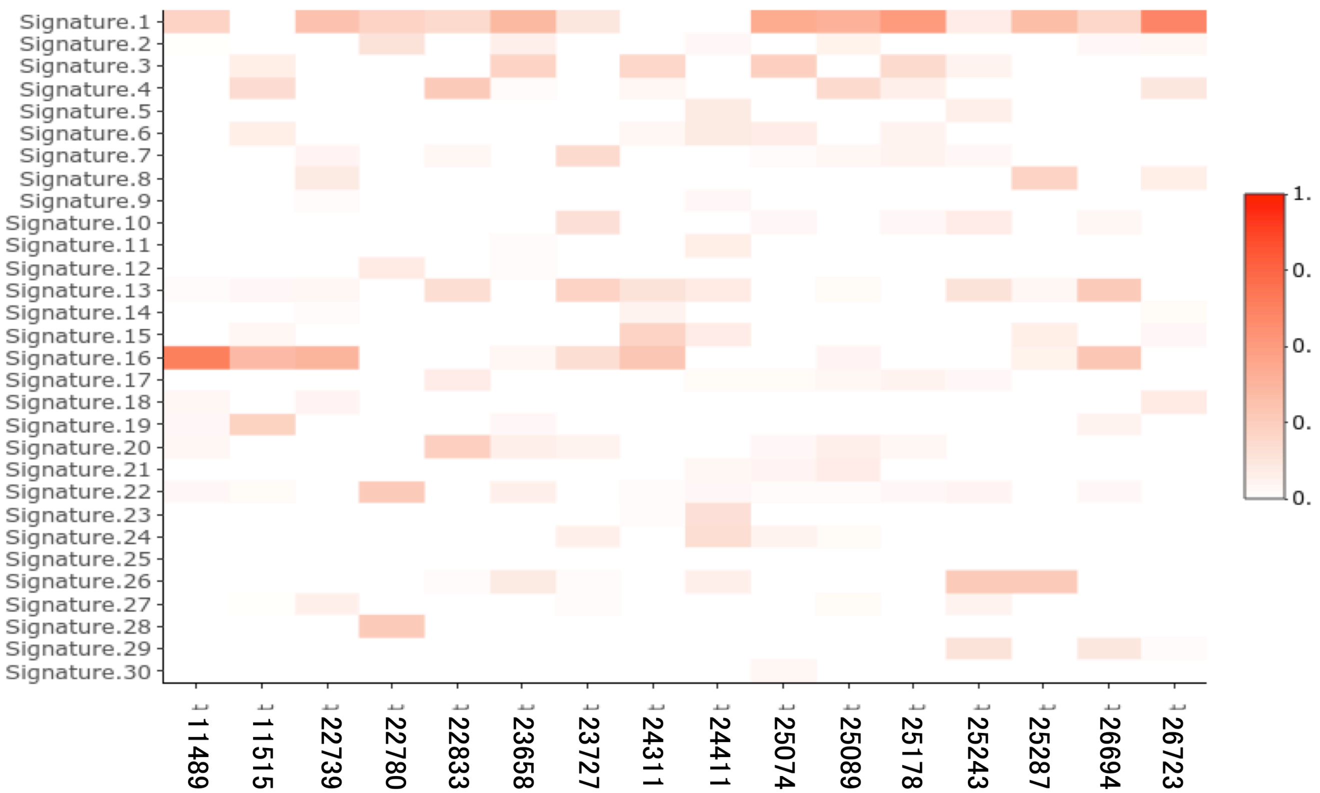


**Supplementary Figure 3: Mutation signatures of ESCC samples used during the screening stage.** (**A**) Number of trinucleotide substitution patterns in esophageal squamous cell neoplasms. Six possible substitutions from pyrimidine bases were further subdivided into 96 patterns based on the neighboring nucleotides. (**B**) PCA plot of mutational signatures.

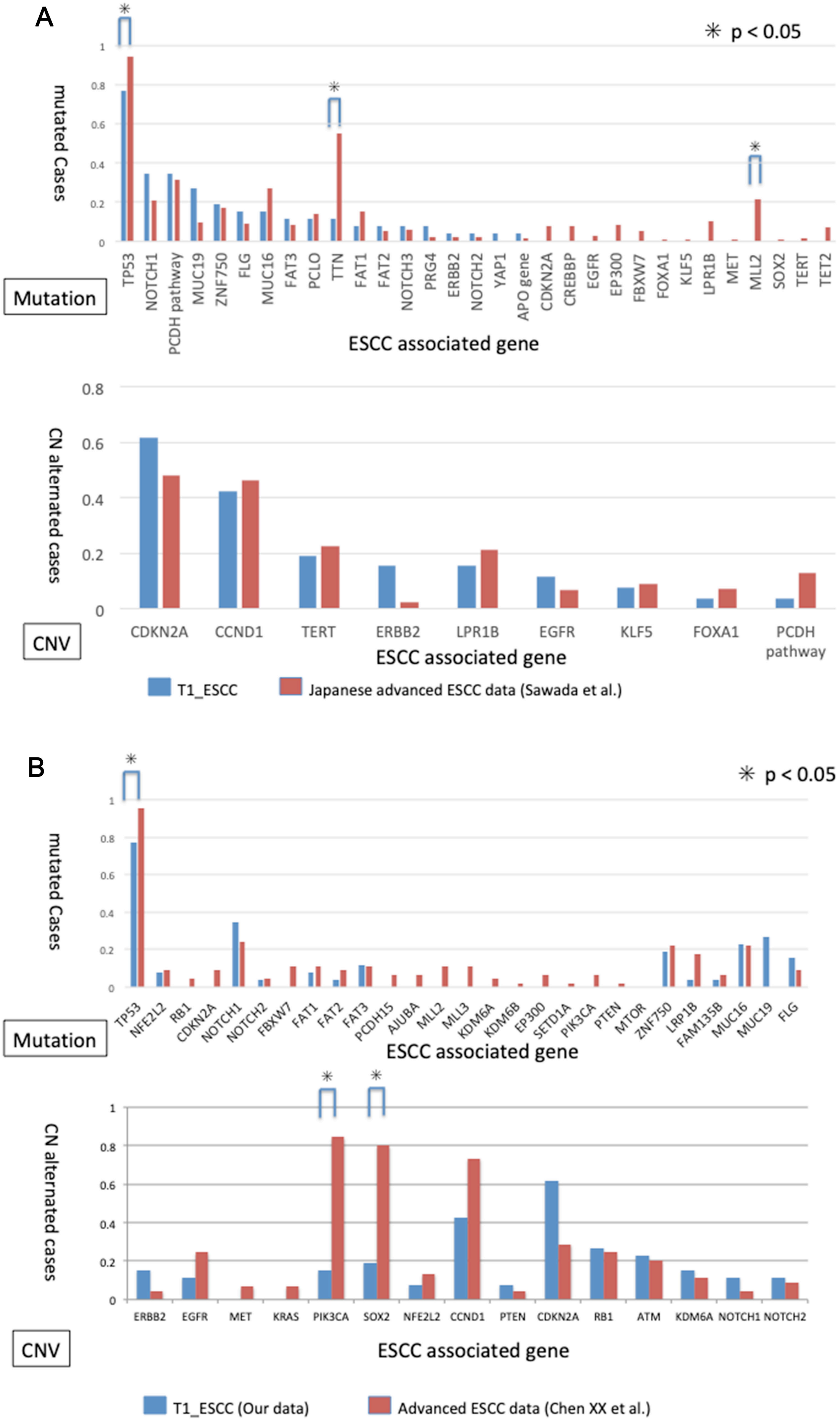

A
✳ p < 0.05
mutated Cases
1
0.8
0.6
0.4
0.2
0
TP53
NOTCH1
PCDH pathway
MUC19
ZNF750
FLG
MUC16
FAT3
PCLO
TTN
FAT1
FAT2
NOTCH3
PRG4
ERBB2
NOTCH2
YAP1
APO gene
CDKN2A
CREBBP
EGFR
EP300
FBXW7
FOXA1
KLF5
LPR1B
MET
MLL2
SOX2
TERT
TET2
Mutation
ESCC associated gene
CN alternated cases
0.8
0.6
0.4
0.2
0
CDKN2A
CCND1
TERT
ERBB2
LPR1B
EGFR
KLF5
FOXA1
PCDH pathway
CNV
ESCC associated gene
T1_ESCC
Japanese advanced ESCC data (Sawada et al.)
B
✳ p < 0.05
mutated Cases
1
0.8
0.6
0.4
0.2
0
TP53
NFE2L2
RB1
CDKN2A
NOTCH1
NOTCH2
FBXW7
FAT1
FAT2
FAT3
PCDH15
AJUBA
MLL2
MLL3
KDM6A
KDM6B
EP300
SETD1A
PIK3CA
PTEN
MTOR
ZNF750
LRP1B
FAM135B
MUC16
MUC19
FLG
Mutation
ESCC associated gene
CN alternated cases
1
0.8
0.6
0.4
0.2
0
ERBB2
EGFR
MET
KRAS
PIK3CA
SOX2
NFE2L2
CCND1
PTEN
CDKN2A
RB1
ATM
KDM6A
NOTCH1
NOTCH2
CNV
ESCC associated gene
T1_ESCC (Our data)
Advanced ESCC data (Chen XX et al.)

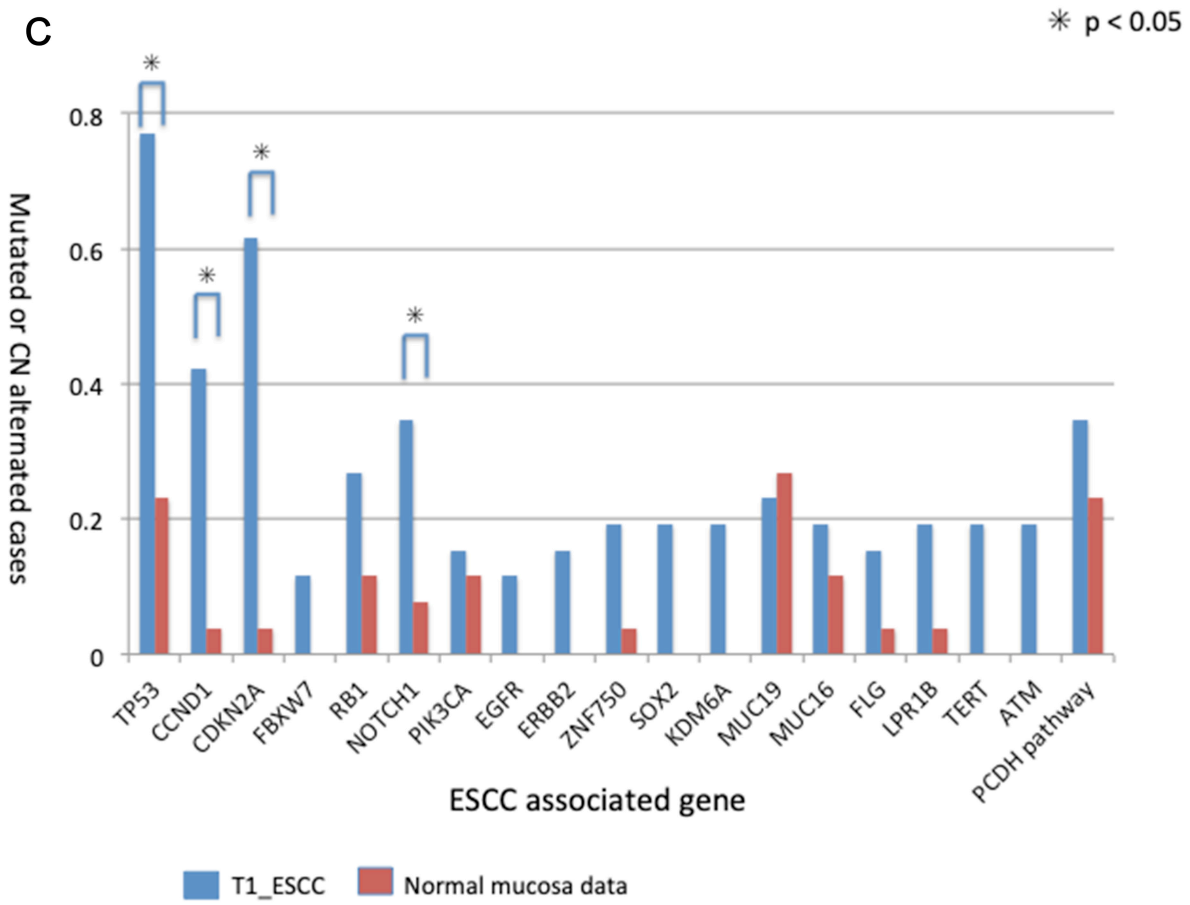


**Supplementary Figure 4:** (**A**) Comparative analysis of the frequency of non-synonymous somatic mutations and copy-number alterations in early stage and advanced-stage cancer [7] in representative esophageal squamous cell carcinoma (ESCC) driver genes. (**B**) Comparative analysis of the frequency of non-synonymous somatic mutations and copy-number alterations in early stage ESCC and advanced-stage cancer [16] in representative esophageal squamous cell carcinoma (ESCC) driver genes. (**C**) Comparative analysis of the frequency of non-synonymous somatic mutations between early-stage esophageal squamous cell carcinoma (ESCC) and non-cancerous tissue in each representative gene. The early stage ESCC and non-cancerous data from 26 ESCC patients with mutations in each representative ESCC driver gene were collected only during the replication stage.

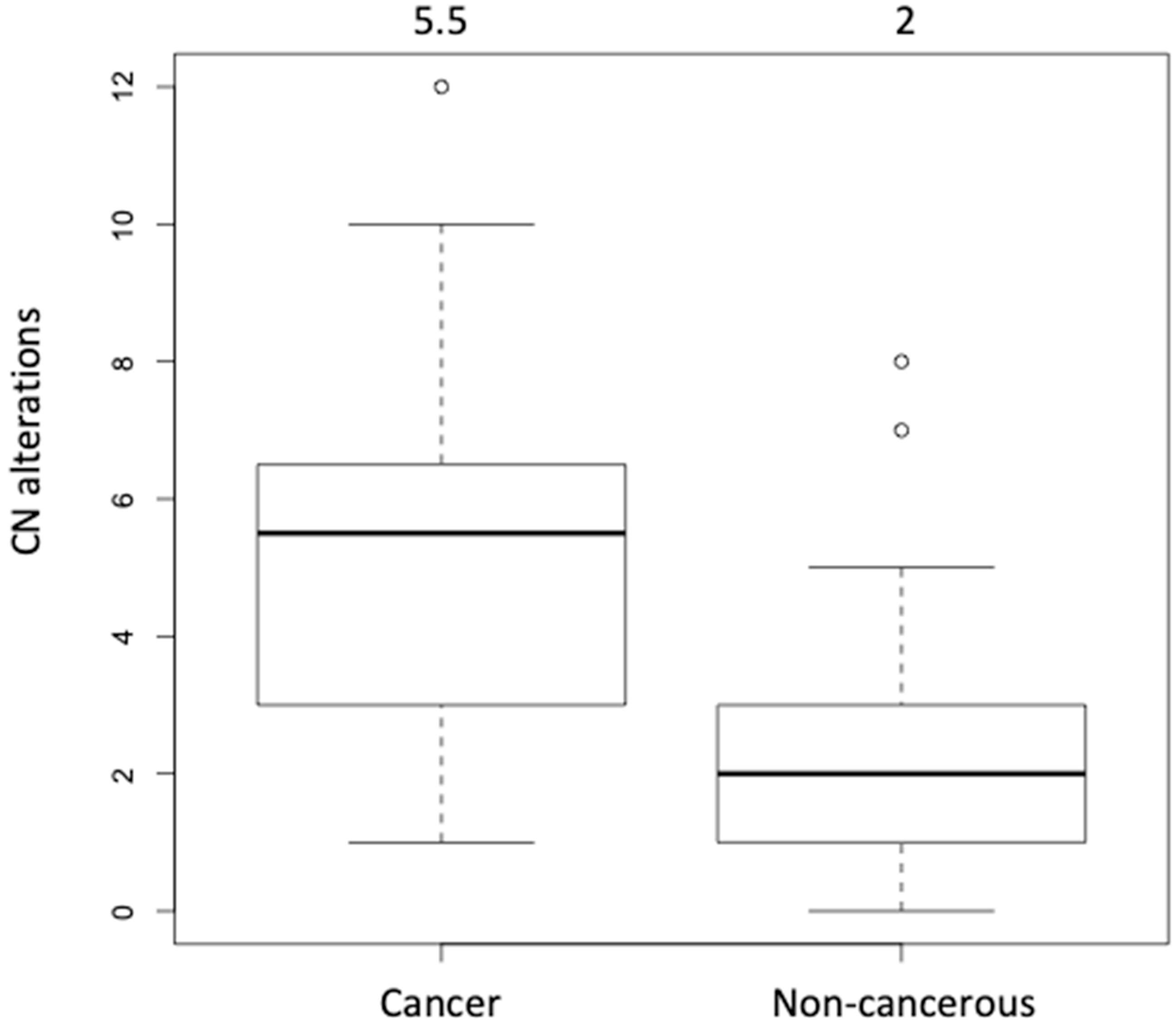


**Supplementary Figure 5: Comparative analysis of copy-number alterations in early-stage esophageal squamous cell carcinoma tissues and matched non-cancerous tissues in 26 replication-stage patients.**
