# Supplementary material for: Genomic characterization of early-stage esophageal squamous cell carcinoma in a Japanese population
Source: Oncotarget. 2019 Jun 25;10(41):4139–48. doi: 10.18632/oncotarget.27014 (PMC6609253; doi:10.18632/oncotarget.27014)
Supplement: Supplementary file 2 [file oncotarget-10-4139-s002.docx]

| **Supplementary Table 4: Somatic mutations in 16 early-stage Esophageal neoplasms using exome sequecncing** | | | | | | | | | | | | | | | |
| --- | --- | --- | --- | --- | --- | --- | --- | --- | --- | --- | --- | --- | --- | --- | --- |
| Chr. | Region | Type | Ref. | Allele | Length | ID | Zygosity | Count | Coverage | Freq. | F/R | Control count | Control coverage | Non-synonymous | hg19_Gene |
| 1 | 6159048 | SNV | A | T | 1 | 11489 | Hetero | 69 | 206 | 33.5 | 0.44 | 0 | 205 | - | KCNAB2 |
| 1 | 13839765 | SNV | G | A | 1 | 11489 | Hetero | 90 | 295 | 30.5 | 0.46 | 0 | 246 | No | LRRC38 |
| 1 | 36322804 | SNV | A | G | 1 | 11489 | Hetero | 27 | 73 | 37.0 | 0.48 | 0 | 47 | - | EIF2C4 |
| 1 | 43317474 | SNV | A | T | 1 | 11489 | Hetero | 88 | 298 | 29.5 | 0.41 | 0 | 297 | Yes | ZNF691 |
| 1 | 62916496 | SNV | A | G | 1 | 11489 | Hetero | 67 | 210 | 31.9 | 0.45 | 0 | 174 | No | USP1 |
| 1 | 68610217 | SNV | G | T | 1 | 11489 | Hetero | 29 | 92 | 31.5 | 0.29 | 0 | 98 | - | GNG12-AS1 |
| 1 | 85418092 | SNV | T | C | 1 | 11489 | Hetero | 44 | 181 | 24.3 | 0.23 | 0 | 152 | - | MCOLN2 |
| 1 | 90050238 | SNV | C | T | 1 | 11489 | Hetero | 66 | 189 | 34.9 | 0.43 | 0 | 181 | No | LRRC8B |
| 1 | 92647976 | SNV | C | A | 1 | 11489 | Hetero | 53 | 155 | 34.2 | 0.46 | 0 | 132 | Yes | KIAA1107 |
| 1 | 110224391 | SNV | C | A | 1 | 11489 | Hetero | 46 | 154 | 29.9 | 0.16 | 0 | 132 | - | GSTM2 |
| 1 | 149881032 | SNV | G | T | 1 | 11489 | Hetero | 55 | 168 | 32.7 | 0.49 | 0 | 151 | Yes | SV2A |
| 1 | 155106211 | SNV | A | G | 1 | 11489 | Hetero | 57 | 203 | 28.1 | 0.45 | 0 | 173 | Yes | EFNA1 |
| 1 | 156911741 | SNV | T | A | 1 | 11489 | Hetero | 60 | 176 | 34.1 | 0.43 | 0 | 146 | Yes | ARHGEF11 |
| 1 | 158654931 | SNV | G | A | 1 | 11489 | Hetero | 55 | 184 | 29.9 | 0.35 | 0 | 159 | No | SPTA1 |
| 1 | 158815446 | SNV | G | C | 1 | 11489 | Hetero | 42 | 191 | 22.0 | 0.33 | 0 | 159 | Yes | MNDA |
| 1 | 160112595 | Deletion | A | - | 1 | 11489 | Hetero | 9 | 51 | 17.6 | 0.11 | 0 | 32 | - | ATP1A2 |
| 1 | 168154099 | SNV | G | T | 1 | 11489 | Hetero | 15 | 66 | 22.7 | 0.50 | 0 | 74 | Yes | TIPRL |
| 1 | 182827726 | SNV | A | T | 1 | 11489 | Hetero | 28 | 92 | 30.4 | 0.21 | 0 | 93 | Yes | DHX9 |
| 1 | 186281996 | SNV | G | A | 1 | 11489 | Hetero | 56 | 170 | 32.9 | 0.35 | 0 | 158 | Yes | PRG4 |
| 1 | 197447049 | SNV | G | C | 1 | 11489 | Hetero | 118 | 390 | 30.3 | 0.26 | 0 | 387 | - | CRB1 |
| 1 | 200817696 | SNV | G | A | 1 | 11489 | Hetero | 41 | 151 | 27.2 | 0.35 | 0 | 120 | Yes | CAMSAP2 |
| 1 | 205890823 | SNV | G | A | 1 | 11489 | Hetero | 9 | 62 | 14.5 | 0.11 | 0 | 62 | No | SLC26A9 |
| 1 | 206944846 | Deletion | T | - | 1 | 11489 | Hetero | 5 | 47 | 10.6 | 0.17 | 0 | 32 | - | IL10 |
| 1 | 207082814 | SNV | G | T | 1 | 11489 | Hetero | 14 | 47 | 29.8 | 0.25 | 0 | 40 | Yes | FAIM3 |
| 1 | 215972290 | SNV | C | A | 1 | 11489 | Hetero | 45 | 177 | 25.4 | 0.35 | 0 | 163 | Yes | USH2A |
| 1 | 215972391 | SNV | C | A | 1 | 11489 | Hetero | 46 | 141 | 32.6 | 0.34 | 0 | 114 | No | USH2A |
| 1 | 220322066 | SNV | T | C | 1 | 11489 | Hetero | 19 | 58 | 32.8 | 0.39 | 0 | 78 | - | RAB3GAP2 |
| 1 | 228369265 | SNV | C | G | 1 | 11489 | Hetero | 47 | 185 | 25.4 | 0.45 | 0 | 152 | - | IBA57 |
| 1 | 231398467 | SNV | A | T | 1 | 11489 | Hetero | 25 | 92 | 27.2 | 0.26 | 0 | 77 | - | GNPAT |
| 1 | 237780692 | SNV | G | A | 1 | 11489 | Hetero | 73 | 224 | 32.6 | 0.46 | 0 | 205 | Yes | RYR2 |
| 1 | 248636873 | SNV | G | A | 1 | 11489 | Hetero | 9 | 87 | 10.3 | 0.11 | 0 | 252 | Yes | OR2T3 |
| 1 | 24077466 | Insertion | - | T | 1 | 11489 | Hetero | 44 | 153 | 28.8 | 0.48 | 0 | 155 | Insertion | TCEB3 |
| 1 | 241793453 | Insertion | - | A | 1 | 11489 | Hetero | 44 | 139 | 31.7 | 0.42 | 0 | 88 | - | OPN3 |
| 1 | 43630389 | Insertion | - | T | 1 | 11489 | Hetero | 44 | 140 | 31.4 | 0.35 | 0 | 133 | Insertion | EBNA1BP2 |
| 1 | 52740272 | Insertion | - | T | 1 | 11489 | Hetero | 26 | 46 | 56.5 | 0.47 | 0 | 39 | - | ZFYVE9 |
| 1 | 59042498 | Insertion | - | G | 1 | 11489 | Hetero | 91 | 336 | 27.1 | 0.47 | 0 | 253 | Insertion | TACSTD2 |
| 2 | 44461672 | Deletion | G | - | 1 | 11489 | Hetero | 9 | 39 | 23.1 | 0.22 | 0 | 30 | - | PPM1B |
| 2 | 48686998 | SNV | C | G | 1 | 11489 | Hetero | 16 | 62 | 25.8 | 0.38 | 0 | 69 | Yes | PPP1R21 |
| 2 | 64683477 | SNV | A | G | 1 | 11489 | Hetero | 33 | 156 | 21.2 | 0.46 | 0 | 101 | Yes | LGALSL |
| 2 | 86365409 | SNV | A | G | 1 | 11489 | Hetero | 28 | 123 | 22.8 | 0.40 | 0 | 99 | - | PTCD3 |
| 2 | 99689549 | SNV | T | A | 1 | 11489 | Hetero | 31 | 81 | 38.3 | 0.09 | 0 | 92 | - | TSGA10 |
| 2 | 99935814 | SNV | A | T | 1 | 11489 | Hetero | 12 | 71 | 16.9 | 0.36 | 0 | 89 | - | TXNDC9 |
| 2 | 138434838 | SNV | A | C | 1 | 11489 | Hetero | 25 | 103 | 24.3 | 0.48 | 0 | 89 | - | THSD7B |
| 2 | 170550849 | SNV | A | G | 1 | 11489 | Hetero | 66 | 202 | 32.7 | 0.44 | 0 | 141 | - | CCDC173 |
| 2 | 175213292 | SNV | T | C | 1 | 11489 | Hetero | 75 | 224 | 33.5 | 0.40 | 0 | 224 | Yes | CIR1 |
| 2 | 175664966 | SNV | T | C | 1 | 11489 | Hetero | 90 | 314 | 28.7 | 0.27 | 0 | 283 | Yes | CHN1 |
| 2 | 176042628 | SNV | T | C | 1 | 11489 | Hetero | 33 | 130 | 25.4 | 0.40 | 0 | 102 | - | ATP5G3 |
| 2 | 182322198 | SNV | C | A | 1 | 11489 | Hetero | 44 | 133 | 33.1 | 0.50 | 0 | 123 | - | ITGA4 |
| 2 | 215440500 | SNV | G | T | 1 | 11489 | Hetero | 49 | 424 | 11.6 | 0.48 | 0 | 487 | Yes | AC107218.3 |
| 2 | 215591778 | SNV | C | G | 1 | 11489 | Hetero | 33 | 145 | 22.8 | 0.32 | 0 | 145 | - | BARD1 |
| 2 | 220096959 | SNV | C | G | 1 | 11489 | Hetero | 39 | 164 | 23.8 | 0.14 | 0 | 145 | - | ANKZF1 |
| 2 | 225362576 | SNV | A | C | 1 | 11489 | Hetero | 17 | 164 | 10.4 | 0.35 | 0 | 124 | - | CUL3 |
| 2 | 225717020 | SNV | C | T | 1 | 11489 | Hetero | 52 | 232 | 22.4 | 0.32 | 0 | 199 | - | DOCK10 |
| 2 | 228137617 | SNV | G | C | 1 | 11489 | Hetero | 21 | 51 | 41.2 | 0.31 | 0 | 54 | - | COL4A3 |
| 2 | 238234196 | SNV | C | A | 1 | 11489 | Hetero | 58 | 272 | 21.3 | 0.29 | 0 | 276 | - | COL6A3 |
| 2 | 227967600 | Insertion | - | TG | 2 | 11489 | Hetero | 20 | 187 | 10.7 | 0.15 | 0 | 160 | - | COL4A4 |
| 2 | 65301406 | Insertion | - | TC | 2 | 11489 | Hetero | 31 | 78 | 39.7 | 0.41 | 0 | 70 | - | CEP68 |
| 3 | 12942375 | SNV | G | C | 1 | 11489 | Hetero | 53 | 97 | 54.6 | 0.25 | 0 | 110 | Yes | IQSEC1 |
| 3 | 15090733 | SNV | C | A | 1 | 11489 | Hetero | 27 | 50 | 54.0 | 0.38 | 0 | 83 | - | NR2C2 |
| 3 | 32746289 | Deletion | T | - | 1 | 11489 | Hetero | 8 | 54 | 14.8 | 0.22 | 0 | 58 | - | CNOT10 |
| 3 | 52558461 | SNV | C | T | 1 | 11489 | Hetero | 40 | 192 | 20.8 | 0.48 | 0 | 211 | - | STAB1 |
| 3 | 56785446 | SNV | T | A | 1 | 11489 | Hetero | 63 | 148 | 42.6 | 0.35 | 0 | 211 | - | ARHGEF3 |
| 3 | 57304576 | SNV | A | T | 1 | 11489 | Hetero | 49 | 107 | 45.8 | 0.39 | 0 | 128 | - | APPL1 |
| 3 | 122129883 | SNV | A | T | 1 | 11489 | Hetero | 41 | 113 | 36.3 | 0.46 | 0 | 90 | - | FAM162A |
| 3 | 130721707 | SNV | A | T | 1 | 11489 | Hetero | 20 | 74 | 27.0 | 0.43 | 0 | 59 | - | ATP2C1 |
| 3 | 150690280 | SNV | C | T | 1 | 11489 | Hetero | 176 | 390 | 45.1 | 0.41 | 0 | 287 | No | CLRN1-AS1 |
| 3 | 178956869 | SNV | A | C | 1 | 11489 | Hetero | 127 | 552 | 23.0 | 0.45 | 0 | 378 | - | PIK3CA |
| 3 | 179504091 | Deletion | A | - | 1 | 11489 | Hetero | 44 | 171 | 25.7 | 0.49 | 0 | 116 | - | USP13 |
| 3 | 150917109 | Insertion | - | A | 1 | 11489 | Hetero | 92 | 408 | 22.5 | 0.31 | 0 | 261 | Insertion | MED12L |
| 3 | 56807854 | Insertion | - | A | 1 | 11489 | Hetero | 5 | 48 | 10.4 | 0.20 | 0 | 71 | - | ARHGEF3 |
| 3 | 98517450 | Insertion | - | A | 1 | 11489 | Hetero | 28 | 101 | 27.7 | 0.47 | 0 | 84 | - | ST3GAL6 |
| 4 | 8407739 | SNV | G | A | 1 | 11489 | Hetero | 50 | 155 | 32.3 | 0.38 | 0 | 135 | Yes | ACOX3 |
| 4 | 26321496 | SNV | G | C | 1 | 11489 | Hetero | 67 | 243 | 27.6 | 0.48 | 0 | 226 | - | RBPJ |
| 4 | 37847376 | SNV | A | G | 1 | 11489 | Hetero | 56 | 167 | 33.5 | 0.22 | 0 | 112 | - | PGM2 |
| 4 | 42153938 | SNV | G | T | 1 | 11489 | Hetero | 29 | 108 | 26.9 | 0.31 | 0 | 83 | Yes | BEND4 |
| 4 | 69189957 | SNV | T | C | 1 | 11489 | Hetero | 15 | 71 | 21.1 | 0.06 | 0 | 47 | - | YTHDC1 |
| 4 | 87610804 | SNV | T | G | 1 | 11489 | Hetero | 30 | 124 | 24.2 | 0.47 | 0 | 91 | Yes | PTPN13 |
| 4 | 99993626 | SNV | G | A | 1 | 11489 | Hetero | 35 | 130 | 26.9 | 0.43 | 0 | 136 | - | ADH5 |
| 4 | 100544617 | Deletion | A | - | 1 | 11489 | Hetero | 28 | 89 | 31.5 | 0.33 | 0 | 70 | - | MTTP |
| 4 | 121719451 | SNV | G | T | 1 | 11489 | Hetero | 7 | 68 | 10.3 | 0.38 | 0 | 38 | Yes | PRDM5 |
| 4 | 144361326 | SNV | A | G | 1 | 11489 | Hetero | 98 | 353 | 27.8 | 0.48 | 0 | 339 | Yes | GAB1 |
| 4 | 148575342 | SNV | G | A | 1 | 11489 | Hetero | 73 | 207 | 35.3 | 0.43 | 0 | 176 | Yes | TMEM184C |
| 4 | 170908423 | SNV | G | C | 1 | 11489 | Hetero | 31 | 114 | 27.2 | 0.33 | 0 | 108 | - | MFAP3L |
| 5 | 10679658 | SNV | C | T | 1 | 11489 | Hetero | 24 | 150 | 16.0 | 0.31 | 0 | 101 | - | DAP |
| 5 | 13690760 | SNV | G | A | 1 | 11489 | Hetero | 33 | 76 | 43.4 | 0.44 | 0 | 34 | - | DNAH5 |
| 5 | 34918626 | SNV | C | A | 1 | 11489 | Hetero | 10 | 55 | 18.2 | 0.18 | 0 | 34 | - | RAD1 |
| 5 | 37065314 | SNV | A | G | 1 | 11489 | Hetero | 47 | 98 | 48.0 | 0.43 | 0 | 78 | - | NIPBL |
| 5 | 86668013 | SNV | G | T | 1 | 11489 | Hetero | 11 | 83 | 13.3 | 0.43 | 0 | 130 | - | RASA1 |
| 5 | 89791610 | Deletion | T | - | 1 | 11489 | Hetero | 3 | 30 | 10.0 | 0.33 | 0 | 30 | - | POLR3G |
| 5 | 150704695 | SNV | G | C | 1 | 11489 | Hetero | 5 | 44 | 11.4 | 0.40 | 0 | 51 | - | SLC36A2 |
| 5 | 175740644 | SNV | C | T | 1 | 11489 | Hetero | 39 | 83 | 47.0 | 0.49 | 0 | 99 | - | C5orf25 |
| 5 | 122344251 | Insertion | - | AA | 2 | 11489 | Hetero | 6 | 54 | 11.1 | 0.50 | 0 | 55 | - | SNX24 |
| 6 | 7602925 | SNV | G | A | 1 | 11489 | Hetero | 14 | 65 | 21.5 | 0.35 | 0 | 36 | Yes | SNRNP48 |
| 6 | 8015546 | SNV | A | G | 1 | 11489 | Hetero | 18 | 78 | 23.1 | 0.45 | 0 | 80 | - | TXNDC5 |
| 6 | 17601364 | SNV | G | A | 1 | 11489 | Hetero | 37 | 146 | 25.3 | 0.50 | 0 | 116 | - | FAM8A1 |
| 6 | 27419540 | Deletion | G | - | 1 | 11489 | Hetero | 64 | 255 | 25.1 | 0.46 | 0 | 213 | Deletion | ZNF184 |
| 6 | 28297329 | SNV | C | G | 1 | 11489 | Hetero | 17 | 167 | 10.2 | 0.33 | 0 | 120 | Yes | ZNF323 |
| 6 | 28874978 | SNV | C | T | 1 | 11489 | Hetero | 7 | 70 | 10.0 | 0.14 | 0 | 51 | - | TRIM27 |
| 6 | 30698396 | SNV | C | A | 1 | 11489 | Hetero | 39 | 175 | 22.3 | 0.40 | 0 | 161 | - | FLOT1 |
| 6 | 37138100 | SNV | C | A | 1 | 11489 | Hetero | 20 | 43 | 46.5 | 0.05 | 0 | 47 | - | PIM1 |
| 6 | 38830163 | SNV | T | C | 1 | 11489 | Hetero | 85 | 290 | 29.3 | 0.50 | 0 | 257 | Yes | DNAH8 |
| 6 | 41033255 | SNV | T | C | 1 | 11489 | Hetero | 20 | 71 | 28.2 | 0.32 | 0 | 96 | - | C6orf130 |
| 6 | 41564829 | SNV | A | T | 1 | 11489 | Hetero | 16 | 70 | 22.9 | 0.33 | 0 | 42 | - | FOXP4 |
| 6 | 46107974 | SNV | C | T | 1 | 11489 | Hetero | 81 | 270 | 30.0 | 0.47 | 0 | 230 | No | ENPP4 |
| 6 | 47684531 | SNV | T | G | 1 | 11489 | Hetero | 21 | 66 | 31.8 | 0.09 | 0 | 63 | - | GPR115 |
| 6 | 109662432 | SNV | T | C | 1 | 11489 | Hetero | 11 | 46 | 23.9 | 0.36 | 0 | 63 | - | CCDC162P |
| 6 | 111214043 | Insertion | - | T | 1 | 11489 | Hetero | 5 | 40 | 12.5 | 0.43 | 0 | 32 | - | AMD1 |
| 6 | 37447018 | Insertion | - | T | 1 | 11489 | Hetero | 44 | 127 | 34.6 | 0.44 | 0 | 108 | Insertion | FTSJD2 |
| 7 | 6656949 | SNV | C | G | 1 | 11489 | Hetero | 17 | 65 | 26.2 | 0.11 | 0 | 65 | - | ZNF853 |
| 7 | 43982522 | SNV | A | T | 1 | 11489 | Hetero | 46 | 150 | 30.7 | 0.49 | 0 | 105 | - | UBE2D4 |
| 7 | 45140001 | SNV | G | C | 1 | 11489 | Hetero | 45 | 156 | 28.8 | 0.45 | 0 | 121 | Yes | TBRG4 |
| 7 | 51152982 | SNV | A | G | 1 | 11489 | Hetero | 58 | 229 | 25.3 | 0.43 | 0 | 184 | Yes | COBL |
| 7 | 55269460 | SNV | C | T | 1 | 11489 | Hetero | 85 | 268 | 31.7 | 0.37 | 0 | 254 | No | EGFR |
| 7 | 73085560 | SNV | G | T | 1 | 11489 | Hetero | 24 | 80 | 30.0 | 0.38 | 0 | 67 | Yes | VPS37D |
| 7 | 87903459 | SNV | G | C | 1 | 11489 | Hetero | 5 | 44 | 11.4 | 0.20 | 0 | 49 | - | AC003991.3 |
| 7 | 103368608 | SNV | T | C | 1 | 11489 | Hetero | 54 | 213 | 25.4 | 0.45 | 0 | 169 | Yes | RELN |
| 7 | 113091304 | SNV | T | A | 1 | 11489 | Hetero | 87 | 298 | 29.2 | 0.32 | 0 | 315 | Yes | AC073348.1 |
| 7 | 119914400 | SNV | G | T | 1 | 11489 | Hetero | 36 | 226 | 15.9 | 0.46 | 0 | 183 | - | KCND2 |
| 7 | 122091467 | SNV | C | G | 1 | 11489 | Hetero | 16 | 51 | 31.4 | 0.25 | 0 | 55 | Yes | CADPS2 |
| 7 | 148481004 | SNV | C | T | 1 | 11489 | Hetero | 38 | 141 | 27.0 | 0.47 | 0 | 136 | - | CUL1 |
| 7 | 148494970 | SNV | A | G | 1 | 11489 | Hetero | 9 | 73 | 12.3 | 0.50 | 0 | 42 | - | CUL1 |
| 7 | 155532573 | SNV | G | C | 1 | 11489 | Hetero | 16 | 70 | 22.9 | 0.06 | 0 | 53 | Yes | RBM33 |
| 7 | 104752530 | Insertion | - | CA | 2 | 11489 | Hetero | 122 | 365 | 33.4 | 0.47 | 0 | 326 | Insertion | MLL5 |
| 7 | 2567892 | Insertion | - | CG | 2 | 11489 | Hetero | 15 | 83 | 18.1 | 0.35 | 0 | 52 | - | LFNG |
| 7 | 6656947 | Insertion | - | T | 1 | 11489 | Hetero | 17 | 68 | 25.0 | 0.11 | 0 | 65 | - | ZNF853 |
| 8 | 6272440 | SNV | A | G | 1 | 11489 | Hetero | 20 | 65 | 30.8 | 0.17 | 0 | 43 | - | MCPH1 |
| 8 | 38034181 | SNV | G | T | 1 | 11489 | Hetero | 64 | 165 | 38.8 | 0.43 | 0 | 117 | - | LSM1 |
| 8 | 42183435 | SNV | G | A | 1 | 11489 | Hetero | 47 | 167 | 28.1 | 0.30 | 0 | 129 | - | IKBKB |
| 8 | 48749881 | SNV | T | C | 1 | 11489 | Hetero | 53 | 163 | 32.5 | 0.39 | 0 | 157 | No | PRKDC |
| 8 | 53049973 | SNV | T | C | 1 | 11489 | Hetero | 68 | 224 | 30.4 | 0.43 | 0 | 186 | - | ST18 |
| 8 | 56705197 | SNV | A | T | 1 | 11489 | Hetero | 39 | 132 | 29.5 | 0.22 | 0 | 117 | - | TGS1 |
| 8 | 59852141 | SNV | A | T | 1 | 11489 | Hetero | 11 | 47 | 23.4 | 0.09 | 0 | 45 | - | TOX |
| 8 | 139144006 | SNV | C | G | 1 | 11489 | Hetero | 40 | 130 | 30.8 | 0.40 | 0 | 111 | - | FAM135B |
| 8 | 140743444 | SNV | G | A | 1 | 11489 | Hetero | 30 | 90 | 33.3 | 0.43 | 0 | 86 | Yes | TRAPPC9 |
| 8 | 144681488 | SNV | G | A | 1 | 11489 | Hetero | 77 | 203 | 37.9 | 0.38 | 0 | 163 | Yes | EEF1D |
| 9 | 6257009 | SNV | G | C | 1 | 11489 | Hetero | 38 | 83 | 45.8 | 0.42 | 0 | 150 | - | IL33 |
| 9 | 35740115 | SNV | T | C | 1 | 11489 | Hetero | 107 | 348 | 30.7 | 0.50 | 0 | 316 | Yes | GBA2 |
| 9 | 35750953 | SNV | C | T | 1 | 11489 | Hetero | 47 | 135 | 34.8 | 0.20 | 0 | 125 | Yes | RGP1 |
| 9 | 97912275 | SNV | A | G | 1 | 11489 | Hetero | 43 | 107 | 40.2 | 0.38 | 0 | 72 | Yes | FANCC |
| 9 | 137017106 | SNV | C | T | 1 | 11489 | Hetero | 20 | 168 | 11.9 | 0.48 | 0 | 129 | Possible splice site disruption | WDR5 |
| 9 | 138713532 | SNV | G | C | 1 | 11489 | Hetero | 74 | 264 | 28.0 | 0.35 | 0 | 206 | Yes | CAMSAP1 |
| 10 | 5030465 | SNV | A | C | 1 | 11489 | Hetero | 25 | 55 | 45.5 | 0.40 | 0 | 75 | - | AKR1C2 |
| 10 | 11374595 | Deletion | T | - | 1 | 11489 | Hetero | 11 | 66 | 16.7 | 0.45 | 0 | 124 | - | CELF2 |
| 10 | 72638374 | SNV | C | T | 1 | 11489 | Hetero | 20 | 94 | 21.3 | 0.36 | 0 | 86 | - | SGPL1 |
| 10 | 87487748 | SNV | A | G | 1 | 11489 | Hetero | 28 | 95 | 29.5 | 0.41 | 0 | 95 | Yes | GRID1 |
| 10 | 93851586 | SNV | C | G | 1 | 11489 | Hetero | 50 | 179 | 27.9 | 0.39 | 0 | 158 | - | CPEB3 |
| 10 | 101715841 | SNV | T | A | 1 | 11489 | Hetero | 48 | 135 | 35.6 | 0.49 | 0 | 128 | Yes | DNMBP |
| 10 | 103545810 | SNV | G | A | 1 | 11489 | Hetero | 36 | 112 | 32.1 | 0.37 | 0 | 102 | - | MGEA5 |
| 10 | 104390336 | SNV | C | A | 1 | 11489 | Hetero | 46 | 124 | 37.1 | 0.42 | 0 | 94 | - | SUFU |
| 10 | 106074781 | SNV | G | T | 1 | 11489 | Hetero | 77 | 262 | 29.4 | 0.39 | 0 | 225 | Yes | ITPRIP |
| 10 | 114205160 | SNV | T | C | 1 | 11489 | Hetero | 38 | 109 | 34.9 | 0.23 | 0 | 87 | Yes | ZDHHC6 |
| 10 | 134915930 | SNV | G | A | 1 | 11489 | Hetero | 40 | 110 | 36.4 | 0.23 | 0 | 87 | - | GPR123 |
| 10 | 76854246 | Deletion | AA | - | 2 | 11489 | Hetero | 22 | 78 | 28.2 | 0.28 | 0 | 54 | - | DUSP13 |
| 11 | 373419 | SNV | C | A | 1 | 11489 | Hetero | 8 | 63 | 12.7 | 0.25 | 0 | 52 | - | B4GALNT4 |
| 11 | 7063867 | SNV | A | G | 1 | 11489 | Hetero | 56 | 167 | 33.5 | 0.38 | 0 | 145 | Yes | NLRP14 |
| 11 | 7490243 | SNV | T | C | 1 | 11489 | Hetero | 21 | 92 | 22.8 | 0.34 | 0 | 60 | - | SYT9 |
| 11 | 7614449 | SNV | A | G | 1 | 11489 | Hetero | 70 | 222 | 31.5 | 0.40 | 0 | 165 | Yes | PPFIBP2 |
| 11 | 14539407 | SNV | T | A | 1 | 11489 | Hetero | 47 | 156 | 30.1 | 0.25 | 0 | 136 | - | PSMA1 |
| 11 | 36596949 | SNV | C | T | 1 | 11489 | Hetero | 45 | 138 | 32.6 | 0.28 | 0 | 90 | Yes | RAG1 |
| 11 | 43602946 | SNV | C | T | 1 | 11489 | Hetero | 19 | 72 | 26.4 | 0.45 | 0 | 59 | - | HSD17B12 |
| 11 | 45928147 | SNV | G | A | 1 | 11489 | Hetero | 110 | 325 | 33.8 | 0.48 | 0 | 237 | No | C11orf94 |
| 11 | 61115803 | SNV | C | T | 1 | 11489 | Hetero | 28 | 128 | 21.9 | 0.39 | 0 | 72 | - | DAK |
| 11 | 66130677 | SNV | G | T | 1 | 11489 | Hetero | 67 | 255 | 26.3 | 0.23 | 0 | 171 | - | RP11-867G23.8 |
| 11 | 66252664 | SNV | G | A | 1 | 11489 | Hetero | 128 | 266 | 48.1 | 0.39 | 0 | 153 | No | DPP3 |
| 11 | 123503578 | SNV | G | T | 1 | 11489 | Hetero | 14 | 31 | 45.2 | 0.29 | 0 | 52 | - | SCN3B |
| 11 | 124294078 | SNV | T | C | 1 | 11489 | Hetero | 37 | 91 | 40.7 | 0.30 | 0 | 145 | No | OR8B4 |
| 11 | 35251769 | Insertion | - | T | 1 | 11489 | Hetero | 15 | 39 | 38.5 | 0.40 | 0 | 35 | - | CD44 |
| 12 | 7528090 | SNV | C | G | 1 | 11489 | Hetero | 26 | 137 | 19.0 | 0.47 | 0 | 138 | Yes | CD163L1 |
| 12 | 29492414 | SNV | T | C | 1 | 11489 | Hetero | 42 | 131 | 32.1 | 0.35 | 0 | 134 | - | FAR2 |
| 12 | 46354774 | SNV | T | C | 1 | 11489 | Hetero | 9 | 41 | 22.0 | 0.36 | 0 | 53 | - | SCAF11 |
| 12 | 49426526 | SNV | G | A | 1 | 11489 | Hetero | 33 | 114 | 28.9 | 0.12 | 0 | 83 | Yes | MLL2 |
| 12 | 56669919 | SNV | A | T | 1 | 11489 | Hetero | 47 | 170 | 27.6 | 0.37 | 0 | 157 | Yes | CS |
| 12 | 56991681 | SNV | T | A | 1 | 11489 | Hetero | 12 | 97 | 12.4 | 0.43 | 0 | 76 | - | BAZ2A |
| 12 | 57926658 | SNV | A | G | 1 | 11489 | Hetero | 88 | 277 | 31.8 | 0.34 | 0 | 195 | - | DCTN2 |
| 12 | 58214351 | SNV | T | A | 1 | 11489 | Hetero | 166 | 476 | 34.9 | 0.48 | 0 | 411 | - | CTDSP2 |
| 12 | 60173226 | SNV | A | T | 1 | 11489 | Hetero | 51 | 200 | 25.5 | 0.34 | 0 | 183 | No | SLC16A7 |
| 12 | 80211256 | SNV | G | C | 1 | 11489 | Hetero | 23 | 82 | 28.0 | 0.45 | 0 | 68 | Yes | PPP1R12A |
| 12 | 94702043 | SNV | A | G | 1 | 11489 | Hetero | 28 | 78 | 35.9 | 0.28 | 0 | 59 | - | CCDC41 |
| 12 | 105509525 | SNV | C | G | 1 | 11489 | Hetero | 81 | 258 | 31.4 | 0.19 | 0 | 179 | - | KIAA1033 |
| 12 | 109494588 | SNV | G | A | 1 | 11489 | Hetero | 84 | 345 | 24.3 | 0.39 | 0 | 318 | Yes | USP30 |
| 12 | 110841481 | SNV | C | T | 1 | 11489 | Hetero | 51 | 184 | 27.7 | 0.47 | 0 | 151 | No | ANAPC7 |
| 12 | 131285663 | SNV | T | A | 1 | 11489 | Hetero | 25 | 78 | 32.1 | 0.26 | 0 | 66 | - | STX2 |
| 12 | 66346864 | Insertion | - | T | 1 | 11489 | Hetero | 9 | 44 | 20.5 | 0.30 | 0 | 44 | - | HMGA2 |
| 13 | 20245389 | SNV | A | G | 1 | 11489 | Hetero | 108 | 342 | 31.6 | 0.34 | 0 | 333 | Yes | MPHOSPH8 |
| 13 | 23905245 | SNV | C | T | 1 | 11489 | Hetero | 46 | 171 | 26.9 | 0.25 | 0 | 165 | Yes | SACS |
| 13 | 25670978 | SNV | C | T | 1 | 11489 | Hetero | 27 | 234 | 11.5 | 0.45 | 0 | 231 | No | PABPC3 |
| 13 | 25670981 | SNV | G | C | 1 | 11489 | Hetero | 26 | 236 | 11.0 | 0.43 | 0 | 239 | No | PABPC3 |
| 13 | 40362503 | SNV | A | T | 1 | 11489 | Hetero | 92 | 280 | 32.9 | 0.44 | 0 | 257 | - | COG6 |
| 13 | 46356680 | SNV | T | G | 1 | 11489 | Hetero | 11 | 95 | 11.6 | 0.42 | 0 | 79 | - | SIAH3 |
| 13 | 52726755 | SNV | T | C | 1 | 11489 | Hetero | 14 | 47 | 29.8 | 0.35 | 0 | 46 | Yes | NEK3 |
| 13 | 58301978 | SNV | C | T | 1 | 11489 | Hetero | 24 | 78 | 30.8 | 0.45 | 0 | 55 | - | PCDH17 |
| 13 | 73330275 | SNV | T | G | 1 | 11489 | Hetero | 7 | 52 | 13.5 | 0.14 | 0 | 50 | - | BORA |
| 13 | 77580453 | SNV | T | C | 1 | 11489 | Hetero | 27 | 85 | 31.8 | 0.24 | 0 | 65 | - | FBXL3 |
| 13 | 96395104 | SNV | A | T | 1 | 11489 | Hetero | 15 | 60 | 25.0 | 0.29 | 0 | 56 | - | DNAJC3 |
| 13 | 113563484 | SNV | G | T | 1 | 11489 | Hetero | 61 | 200 | 30.5 | 0.27 | 0 | 130 | - | MCF2L |
| 13 | 73330273 | Insertion | - | C | 1 | 11489 | Hetero | 6 | 53 | 11.3 | 0.17 | 0 | 52 | - | BORA |
| 14 | 24887950 | SNV | A | C | 1 | 11489 | Hetero | 96 | 141 | 68.1 | 0.49 | 0 | 113 | - | NYNRIN |
| 14 | 64907213 | SNV | T | C | 1 | 11489 | Hetero | 108 | 191 | 56.5 | 0.45 | 0 | 170 | - | MTHFD1 |
| 14 | 23451323 | Insertion | - | CC | 2 | 11489 | Hetero | 275 | 445 | 61.8 | 0.48 | 0 | 362 | Insertion | AJUBA |
| 15 | 55613680 | SNV | A | C | 1 | 11489 | Hetero | 4 | 33 | 12.1 | 0.25 | 0 | 30 | - | PIGB |
| 15 | 65447422 | SNV | G | A | 1 | 11489 | Hetero | 52 | 152 | 34.2 | 0.17 | 0 | 173 | - | CLPX |
| 15 | 70341433 | SNV | T | A | 1 | 11489 | Hetero | 10 | 87 | 11.5 | 0.18 | 0 | 81 | - | TLE3 |
| 15 | 88409399 | SNV | G | A | 1 | 11489 | Hetero | 43 | 113 | 38.1 | 0.35 | 0 | 98 | - | NTRK3 |
| 15 | 77363218 | Insertion | - | C | 1 | 11489 | Hetero | 26 | 91 | 28.6 | 0.19 | 0 | 80 | - | TSPAN3 |
| 15 | 83808031 | Insertion | - | A | 1 | 11489 | Hetero | 6 | 48 | 12.5 | 0.43 | 0 | 36 | - | TM6SF1 |
| 16 | 14307403 | SNV | C | G | 1 | 11489 | Hetero | 13 | 54 | 24.1 | 0.13 | 0 | 47 | Possible splice site disruption | MKL2 |
| 16 | 30680720 | SNV | G | T | 1 | 11489 | Hetero | 84 | 313 | 26.8 | 0.44 | 0 | 264 | Yes | FBRS |
| 16 | 46964536 | SNV | A | G | 1 | 11489 | Hetero | 52 | 156 | 33.3 | 0.46 | 0 | 159 | - | GPT2 |
| 16 | 76513376 | SNV | A | C | 1 | 11489 | Hetero | 57 | 208 | 27.4 | 0.45 | 0 | 172 | Yes | CNTNAP4 |
| 16 | 86601075 | SNV | C | T | 1 | 11489 | Hetero | 57 | 176 | 32.4 | 0.46 | 0 | 155 | Yes | FOXC2 |
| 16 | 66544113 | Insertion | - | AA | 2 | 11489 | Hetero | 11 | 77 | 14.3 | 0.25 | 0 | 72 | - | RP11-403P17.5 |
| 17 | 650796 | SNV | C | T | 1 | 11489 | Hetero | 124 | 404 | 30.7 | 0.47 | 0 | 441 | Yes | GEMIN4 |
| 17 | 3729440 | Deletion | T | - | 1 | 11489 | Hetero | 12 | 68 | 17.6 | 0.14 | 0 | 66 | Deletion | C17orf85 |
| 17 | 5393374 | SNV | G | A | 1 | 11489 | Hetero | 21 | 62 | 33.9 | 0.20 | 0 | 62 | - | MIS12 |
| 17 | 7577536 | SNV | T | A | 1 | 11489 | Hetero | 49 | 89 | 55.1 | 0.46 | 0 | 98 | Yes | TP53 |
| 17 | 15964747 | SNV | C | A | 1 | 11489 | Hetero | 34 | 125 | 27.2 | 0.40 | 0 | 90 | Yes | NCOR1 |
| 17 | 18873790 | SNV | T | C | 1 | 11489 | Hetero | 48 | 74 | 64.9 | 0.21 | 0 | 82 | - | SLC5A10 |
| 17 | 27077546 | SNV | T | G | 1 | 11489 | Hetero | 37 | 119 | 31.1 | 0.40 | 0 | 87 | - | TRAF4 |
| 17 | 35981297 | SNV | C | A | 1 | 11489 | Hetero | 56 | 179 | 31.3 | 0.33 | 0 | 147 | Yes | DDX52 |
| 17 | 37075285 | SNV | G | A | 1 | 11489 | Hetero | 275 | 541 | 50.8 | 0.26 | 0 | 441 | - | LASP1 |
| 17 | 43481718 | SNV | T | C | 1 | 11489 | Hetero | 59 | 181 | 32.6 | 0.44 | 0 | 159 | - | ARHGAP27 |
| 17 | 73231745 | SNV | A | G | 1 | 11489 | Hetero | 67 | 239 | 28.0 | 0.49 | 0 | 162 | Yes | NUP85 |
| 17 | 73824263 | SNV | C | T | 1 | 11489 | Hetero | 17 | 48 | 35.4 | 0.26 | 0 | 43 | - | UNC13D |
| 17 | 79875615 | SNV | G | C | 1 | 11489 | Hetero | 23 | 65 | 35.4 | 0.13 | 0 | 63 | - | SIRT7 |
| 17 | 79914768 | SNV | C | T | 1 | 11489 | Hetero | 53 | 190 | 27.9 | 0.47 | 0 | 150 | Yes | NOTUM |
| 17 | 37819722 | Insertion | - | A | 1 | 11489 | Hetero | 8 | 58 | 13.8 | 0.13 | 0 | 36 | - | STARD3 |
| 17 | 7080112 | Deletion | CA | - | 2 | 11489 | Hetero | 6 | 57 | 10.5 | 0.25 | 0 | 38 | - | ASGR1 |
| 17 | 80521231 | Insertion | - | GC | 2 | 11489 | Hetero | 78 | 277 | 28.2 | 0.37 | 0 | 267 | Insertion | FOXK2 |
| 19 | 501786 | SNV | C | A | 1 | 11489 | Hetero | 8 | 78 | 10.3 | 0.50 | 0 | 60 | Yes | MADCAM1 |
| 19 | 9046086 | SNV | G | T | 1 | 11489 | Hetero | 20 | 186 | 10.8 | 0.45 | 0 | 198 | Yes | MUC16 |
| 19 | 10084976 | SNV | C | T | 1 | 11489 | Hetero | 107 | 386 | 27.7 | 0.37 | 0 | 348 | - | COL5A3 |
| 19 | 16466655 | SNV | T | C | 1 | 11489 | Hetero | 52 | 178 | 29.2 | 0.42 | 0 | 166 | Yes | EPS15L1 |
| 19 | 17303901 | SNV | A | G | 1 | 11489 | Hetero | 34 | 130 | 26.2 | 0.40 | 0 | 111 | - | MYO9B |
| 19 | 38198898 | SNV | G | C | 1 | 11489 | Hetero | 84 | 275 | 30.5 | 0.26 | 0 | 281 | Yes | ZNF607 |
| 19 | 41248222 | SNV | G | A | 1 | 11489 | Hetero | 93 | 313 | 29.7 | 0.46 | 0 | 254 | - | C19orf54 |
| 19 | 44079169 | SNV | G | A | 1 | 11489 | Hetero | 51 | 144 | 35.4 | 0.38 | 0 | 131 | - | XRCC1 |
| 19 | 45562573 | SNV | C | G | 1 | 11489 | Hetero | 90 | 284 | 31.7 | 0.31 | 0 | 213 | Yes | CLASRP |
| 19 | 45593512 | SNV | C | T | 1 | 11489 | Hetero | 49 | 199 | 24.6 | 0.47 | 0 | 164 | Yes | GEMIN7 |
| 19 | 50926139 | SNV | G | A | 1 | 11489 | Hetero | 59 | 198 | 29.8 | 0.37 | 0 | 157 | Yes | SPIB |
| 20 | 1902237 | SNV | C | A | 1 | 11489 | Hetero | 55 | 185 | 29.7 | 0.33 | 0 | 160 | Yes | SIRPA |
| 20 | 5526820 | SNV | G | A | 1 | 11489 | Hetero | 47 | 126 | 37.3 | 0.48 | 0 | 115 | - | GPCPD1 |
| 20 | 10394416 | SNV | C | A | 1 | 11489 | Hetero | 29 | 97 | 29.9 | 0.39 | 0 | 74 | - | MKKS |
| 20 | 36146510 | SNV | T | C | 1 | 11489 | Hetero | 58 | 221 | 26.2 | 0.34 | 0 | 196 | - | BLCAP |
| 20 | 45314129 | SNV | C | A | 1 | 11489 | Hetero | 23 | 70 | 32.9 | 0.38 | 0 | 63 | - | TP53RK |
| 20 | 48252481 | SNV | G | T | 1 | 11489 | Hetero | 72 | 169 | 42.6 | 0.27 | 0 | 138 | - | B4GALT5 |
| 20 | 48256158 | SNV | T | C | 1 | 11489 | Hetero | 50 | 142 | 35.2 | 0.22 | 0 | 107 | - | B4GALT5 |
| 20 | 60705651 | SNV | A | G | 1 | 11489 | Hetero | 117 | 358 | 32.7 | 0.47 | 0 | 324 | Yes | LSM14B |
| 20 | 61341049 | SNV | G | A | 1 | 11489 | Hetero | 132 | 406 | 32.5 | 0.41 | 0 | 373 | Yes | NTSR1 |
| 20 | 62566209 | SNV | G | A | 1 | 11489 | Hetero | 87 | 191 | 45.5 | 0.42 | 0 | 148 | - | DNAJC5 |
| 22 | 21352467 | SNV | G | T | 1 | 11489 | Hetero | 28 | 280 | 10.0 | 0.50 | 0 | 206 | - | LZTR1 |
| 22 | 24199260 | SNV | T | G | 1 | 11489 | Hetero | 8 | 70 | 11.4 | 0.11 | 0 | 53 | - | SLC2A11 |
| 22 | 26181401 | SNV | G | T | 1 | 11489 | Hetero | 12 | 100 | 12.0 | 0.23 | 0 | 65 | Yes | MYO18B |
| 22 | 26875278 | SNV | C | T | 1 | 11489 | Hetero | 85 | 385 | 22.1 | 0.41 | 0 | 251 | Yes | HPS4 |
| 22 | 29445945 | SNV | C | T | 1 | 11489 | Hetero | 365 | 786 | 46.4 | 0.44 | 0 | 525 | No | ZNRF3 |
| 22 | 44333092 | SNV | C | T | 1 | 11489 | Hetero | 73 | 174 | 42.0 | 0.33 | 0 | 102 | Yes | PNPLA3 |
| 22 | 51150036 | SNV | T | A | 1 | 11489 | Hetero | 56 | 112 | 50.0 | 0.49 | 0 | 73 | - | SHANK3 |
| X | 53402158 | SNV | T | G | 1 | 11489 | Hetero | 44 | 71 | 62.0 | 0.48 | 0 | 62 | - | SMC1A |
| 1 | 19549190 | SNV | T | C | 1 | 11515 | Hetero | 16 | 112 | 14.3 | 0.21 | 0 | 72 | Yes | EMC1 |
| 1 | 22418276 | Deletion | G | - | 1 | 11515 | Hetero | 34 | 38 | 89.5 | 0.29 | 0 | 30 | - | CDC42 |
| 1 | 65311175 | SNV | T | C | 1 | 11515 | Hetero | 10 | 81 | 12.3 | 0.10 | 0 | 86 | - | JAK1 |
| 1 | 100548549 | SNV | G | C | 1 | 11515 | Hetero | 8 | 73 | 11.0 | 0.44 | 0 | 47 | - | HIAT1 |
| 1 | 111660135 | SNV | C | T | 1 | 11515 | Hetero | 9 | 54 | 16.7 | 0.33 | 0 | 37 | - | DRAM2 |
| 1 | 145535827 | SNV | A | T | 1 | 11515 | Hetero | 30 | 143 | 21.0 | 0.28 | 0 | 120 | Yes | ITGA10 |
| 1 | 160062365 | SNV | C | T | 1 | 11515 | Hetero | 19 | 118 | 16.1 | 0.46 | 0 | 96 | Yes | IGSF8 |
| 1 | 162330753 | SNV | G | T | 1 | 11515 | Hetero | 16 | 55 | 29.1 | 0.37 | 0 | 58 | - | NOS1AP |
| 1 | 170697464 | SNV | T | A | 1 | 11515 | Hetero | 6 | 48 | 12.5 | 0.50 | 0 | 46 | - | PRRX1 |
| 1 | 183195832 | Deletion | G | - | 1 | 11515 | Hetero | 26 | 145 | 17.9 | 0.32 | 0 | 115 | - | LAMC2 |
| 1 | 209961217 | Deletion | T | - | 1 | 11515 | Hetero | 23 | 129 | 17.8 | 0.48 | 0 | 119 | - | IRF6 |
| 1 | 222833135 | SNV | G | C | 1 | 11515 | Hetero | 29 | 150 | 19.3 | 0.36 | 0 | 139 | Yes | MIA3 |
| 1 | 150783108 | Insertion | - | A | 1 | 11515 | Hetero | 7 | 57 | 12.3 | 0.43 | 0 | 50 | - | ARNT |
| 1 | 236713364 | Insertion | - | T | 1 | 11515 | Hetero | 18 | 138 | 13.0 | 0.36 | 0 | 114 | - | LGALS8 |
| 1 | 26112414 | Insertion | - | A | 1 | 11515 | Hetero | 7 | 69 | 10.1 | 0.13 | 0 | 72 | - | MAN1C1 |
| 2 | 7001259 | SNV | C | A | 1 | 11515 | Hetero | 12 | 64 | 18.8 | 0.08 | 0 | 61 | - | CMPK2 |
| 2 | 24223389 | SNV | C | G | 1 | 11515 | Hetero | 5 | 40 | 12.5 | 0.29 | 0 | 35 | - | UBXN2A |
| 2 | 103378598 | SNV | G | T | 1 | 11515 | Hetero | 9 | 70 | 12.9 | 0.11 | 0 | 52 | - | TMEM182 |
| 2 | 172180414 | SNV | T | C | 1 | 11515 | Hetero | 6 | 42 | 14.3 | 0.33 | 0 | 32 | - | METTL8 |
| 2 | 179634392 | SNV | A | T | 1 | 11515 | Hetero | 28 | 62 | 45.2 | 0.19 | 0 | 43 | - | TTN |
| 2 | 240504907 | Insertion | - | AG | 2 | 11515 | Hetero | 35 | 235 | 14.9 | 0.16 | 0 | 230 | - | AC079612.1 |
| 3 | 3192062 | Deletion | T | - | 1 | 11515 | Hetero | 16 | 77 | 20.8 | 0.28 | 0 | 95 | - | TRNT1 |
| 3 | 9975617 | SNV | C | A | 1 | 11515 | Hetero | 75 | 428 | 17.5 | 0.44 | 0 | 311 | - | CRELD1 |
| 3 | 25638975 | SNV | T | G | 1 | 11515 | Hetero | 9 | 70 | 12.9 | 0.30 | 0 | 64 | - | RARB |
| 3 | 50365654 | SNV | T | A | 1 | 11515 | Hetero | 26 | 157 | 16.6 | 0.48 | 0 | 105 | - | TUSC2 |
| 3 | 52435736 | SNV | C | T | 1 | 11515 | Hetero | 15 | 142 | 10.6 | 0.47 | 0 | 85 | - | BAP1 |
| 3 | 65456198 | Deletion | G | - | 1 | 11515 | Hetero | 10 | 50 | 20.0 | 0.09 | 0 | 36 | - | MAGI1 |
| 3 | 113187028 | SNV | C | T | 1 | 11515 | Hetero | 18 | 170 | 10.6 | 0.43 | 0 | 142 | No | SPICE1 |
| 3 | 141332669 | SNV | A | G | 1 | 11515 | Hetero | 11 | 107 | 10.3 | 0.46 | 0 | 85 | - | RASA2 |
| 3 | 156876624 | SNV | G | C | 1 | 11515 | Hetero | 15 | 145 | 10.3 | 0.42 | 0 | 81 | - | CCNL1 |
| 3 | 178952911 | Deletion | A | - | 1 | 11515 | Hetero | 8 | 69 | 11.6 | 0.25 | 0 | 38 | - | PIK3CA |
| 3 | 179517617 | SNV | C | T | 1 | 11515 | Hetero | 15 | 106 | 14.2 | 0.37 | 0 | 90 | - | PEX5L |
| 3 | 108179220 | Deletion | AA | - | 2 | 11515 | Hetero | 6 | 53 | 11.3 | 0.25 | 0 | 45 | - | MYH15 |
| 3 | 153974694 | Insertion | - | A | 1 | 11515 | Hetero | 56 | 375 | 14.9 | 0.38 | 0 | 312 | Insertion | ARHGEF26 |
| 3 | 48518513 | Insertion | - | AA | 2 | 11515 | Hetero | 12 | 77 | 15.6 | 0.29 | 0 | 64 | - | SHISA5 |
| 3 | 93754286 | Insertion | - | T | 1 | 11515 | Hetero | 5 | 42 | 11.9 | 0.20 | 0 | 39 | - | ARL13B |
| 4 | 17631196 | SNV | C | T | 1 | 11515 | Hetero | 15 | 94 | 16.0 | 0.35 | 0 | 90 | - | FAM184B |
| 4 | 20525728 | SNV | C | T | 1 | 11515 | Hetero | 20 | 106 | 18.9 | 0.48 | 0 | 115 | Yes | SLIT2 |
| 4 | 36068507 | SNV | T | C | 1 | 11515 | Hetero | 25 | 107 | 23.4 | 0.49 | 0 | 111 | - | ARAP2 |
| 4 | 56298918 | SNV | T | C | 1 | 11515 | Hetero | 7 | 68 | 10.3 | 0.40 | 0 | 51 | - | CLOCK |
| 4 | 106197273 | SNV | G | T | 1 | 11515 | Hetero | 26 | 133 | 19.5 | 0.31 | 0 | 104 | Yes | TET2 |
| 4 | 154623944 | SNV | G | A | 1 | 11515 | Hetero | 10 | 63 | 15.9 | 0.42 | 0 | 63 | - | TLR2 |
| 5 | 61699253 | SNV | G | T | 1 | 11515 | Hetero | 22 | 191 | 11.5 | 0.23 | 0 | 169 | - | KIF2A |
| 5 | 68426053 | SNV | A | G | 1 | 11515 | Hetero | 18 | 95 | 18.9 | 0.26 | 0 | 89 | - | SLC30A5 |
| 5 | 86659192 | SNV | A | G | 1 | 11515 | Hetero | 10 | 55 | 18.2 | 0.31 | 0 | 41 | Yes | RASA1 |
| 5 | 95414841 | SNV | C | A | 1 | 11515 | Hetero | 8 | 73 | 11.0 | 0.13 | 0 | 67 | - | CTD-2337A12.1 |
| 6 | 10509399 | SNV | G | T | 1 | 11515 | Hetero | 25 | 238 | 10.5 | 0.37 | 0 | 211 | - | GCNT2 |
| 6 | 89321515 | SNV | C | A | 1 | 11515 | Hetero | 9 | 58 | 15.5 | 0.44 | 0 | 67 | - | RNGTT |
| 6 | 155099246 | SNV | A | G | 1 | 11515 | Hetero | 8 | 46 | 17.4 | 0.25 | 0 | 32 | - | SCAF8 |
| 6 | 152422169 | Insertion | - | T | 1 | 11515 | Hetero | 6 | 55 | 10.9 | 0.17 | 0 | 52 | - | ESR1 |
| 7 | 22160342 | SNV | T | C | 1 | 11515 | Hetero | 14 | 86 | 16.3 | 0.39 | 0 | 80 | - | RAPGEF5 |
| 7 | 23545969 | SNV | G | C | 1 | 11515 | Hetero | 8 | 52 | 15.4 | 0.22 | 0 | 49 | - | TRA2A |
| 7 | 75958175 | Deletion | A | - | 1 | 11515 | Hetero | 8 | 73 | 11.0 | 0.38 | 0 | 53 | - | YWHAG |
| 7 | 77423081 | SNV | T | A | 1 | 11515 | Hetero | 7 | 63 | 11.1 | 0.14 | 0 | 60 | - | TMEM60 |
| 7 | 100159834 | SNV | C | T | 1 | 11515 | Hetero | 17 | 127 | 13.4 | 0.41 | 0 | 111 | - | AGFG2 |
| 7 | 103557581 | SNV | T | C | 1 | 11515 | Hetero | 24 | 230 | 10.4 | 0.34 | 0 | 217 | Yes | RELN |
| 7 | 139762909 | SNV | G | C | 1 | 11515 | Hetero | 16 | 134 | 11.9 | 0.35 | 0 | 77 | - | PARP12 |
| 8 | 9567550 | SNV | A | T | 1 | 11515 | Hetero | 6 | 52 | 11.5 | 0.33 | 0 | 55 | Yes | TNKS |
| 8 | 23186037 | SNV | C | T | 1 | 11515 | Hetero | 15 | 139 | 10.8 | 0.43 | 0 | 105 | No | LOXL2 |
| 8 | 104442894 | SNV | A | T | 1 | 11515 | Hetero | 7 | 57 | 12.3 | 0.33 | 0 | 37 | Yes | DCAF13 |
| 8 | 12882413 | Insertion | - | T | 1 | 11515 | Hetero | 11 | 99 | 11.1 | 0.23 | 0 | 89 | - | KIAA1456 |
| 9 | 21854675 | SNV | A | T | 1 | 11515 | Hetero | 9 | 77 | 11.7 | 0.11 | 0 | 60 | Yes | MTAP |
| 9 | 86276110 | SNV | C | A | 1 | 11515 | Hetero | 12 | 61 | 19.7 | 0.46 | 0 | 38 | - | UBQLN1 |
| 9 | 97419562 | SNV | C | T | 1 | 11515 | Hetero | 5 | 33 | 15.2 | 0.40 | 0 | 42 | - | C9orf118 |
| 9 | 130533705 | SNV | C | A | 1 | 11515 | Hetero | 12 | 120 | 10.0 | 0.36 | 0 | 88 | - | SH2D3C |
| 9 | 139412326 | SNV | C | A | 1 | 11515 | Hetero | 31 | 183 | 16.9 | 0.35 | 0 | 130 | Yes | NOTCH1 |
| 9 | 33921843 | Insertion | - | T | 1 | 11515 | Hetero | 4 | 40 | 10.0 | 0.25 | 0 | 39 | - | UBAP2 |
| 10 | 35456615 | SNV | C | T | 1 | 11515 | Hetero | 12 | 111 | 10.8 | 0.17 | 0 | 81 | - | CREM |
| 10 | 94688190 | SNV | G | A | 1 | 11515 | Hetero | 7 | 64 | 10.9 | 0.50 | 0 | 68 | - | EXOC6 |
| 10 | 95288504 | SNV | G | A | 1 | 11515 | Hetero | 11 | 110 | 10.0 | 0.33 | 0 | 103 | - | CEP55 |
| 10 | 120791155 | SNV | C | A | 1 | 11515 | Hetero | 9 | 77 | 11.7 | 0.40 | 0 | 58 | - | NANOS1 |
| 11 | 34682564 | SNV | T | G | 1 | 11515 | Hetero | 7 | 45 | 15.6 | 0.22 | 0 | 42 | - | EHF |
| 11 | 35327842 | Deletion | A | - | 1 | 11515 | Hetero | 5 | 50 | 10.0 | 0.40 | 0 | 54 | - | SLC1A2 |
| 11 | 128851443 | SNV | C | A | 1 | 11515 | Hetero | 7 | 53 | 13.2 | 0.14 | 0 | 51 | Yes | ARHGAP32 |
| 11 | 57505520 | Insertion | - | T | 1 | 11515 | Hetero | 5 | 45 | 11.1 | 0.43 | 0 | 53 | - | TMX2 |
| 11 | 63395791 | Insertion | - | A | 1 | 11515 | Hetero | 4 | 32 | 12.5 | 0.25 | 0 | 38 | - | ATL3 |
| 12 | 41423002 | SNV | G | T | 1 | 11515 | Hetero | 15 | 130 | 11.5 | 0.41 | 0 | 100 | No | CNTN1 |
| 12 | 51511393 | SNV | T | C | 1 | 11515 | Hetero | 12 | 119 | 10.1 | 0.31 | 0 | 111 | - | TFCP2 |
| 12 | 95471696 | SNV | T | A | 1 | 11515 | Hetero | 17 | 105 | 16.2 | 0.40 | 0 | 113 | - | FGD6 |
| 12 | 110656517 | SNV | A | G | 1 | 11515 | Hetero | 5 | 32 | 15.6 | 0.29 | 0 | 38 | - | IFT81 |
| 13 | 42873805 | SNV | A | G | 1 | 11515 | Hetero | 11 | 79 | 13.9 | 0.27 | 0 | 76 | Yes | AKAP11 |
| 13 | 84453837 | SNV | G | T | 1 | 11515 | Hetero | 14 | 131 | 10.7 | 0.47 | 0 | 135 | No | SLITRK1 |
| 13 | 111145595 | SNV | C | A | 1 | 11515 | Hetero | 29 | 185 | 15.7 | 0.41 | 0 | 173 | No | COL4A2 |
| 13 | 111940725 | SNV | C | T | 1 | 11515 | Hetero | 15 | 108 | 13.9 | 0.37 | 0 | 126 | Yes | ARHGEF7 |
| 14 | 24701528 | SNV | T | G | 1 | 11515 | Hetero | 19 | 164 | 11.6 | 0.36 | 0 | 139 | - | NEDD8-MDP1 |
| 14 | 24798574 | Deletion | G | - | 1 | 11515 | Hetero | 22 | 214 | 10.3 | 0.32 | 0 | 162 | - | ADCY4 |
| 15 | 44092493 | SNV | T | G | 1 | 11515 | Hetero | 5 | 49 | 10.2 | 0.43 | 0 | 53 | - | SERF2 |
| 15 | 63944621 | SNV | G | A | 1 | 11515 | Hetero | 14 | 102 | 13.7 | 0.42 | 0 | 83 | No | HERC1 |
| 15 | 88411794 | SNV | C | A | 1 | 11515 | Hetero | 8 | 55 | 14.5 | 0.50 | 0 | 54 | - | NTRK3 |
| 16 | 21185410 | SNV | G | T | 1 | 11515 | Hetero | 13 | 109 | 11.9 | 0.50 | 0 | 74 | No | TMEM159 |
| 16 | 23646873 | SNV | G | C | 1 | 11515 | Hetero | 10 | 84 | 11.9 | 0.45 | 0 | 100 | Yes | PALB2 |
| 16 | 30041484 | SNV | G | A | 1 | 11515 | Hetero | 14 | 112 | 12.5 | 0.25 | 0 | 68 | - | FAM57B |
| 16 | 89167068 | Insertion | - | G | 1 | 11515 | Hetero | 12 | 86 | 14.0 | 0.08 | 0 | 85 | - | ACSF3 |
| 17 | 7577530 | SNV | T | A | 1 | 11515 | Hetero | 10 | 73 | 13.7 | 0.38 | 0 | 65 | Yes | TP53 |
| 17 | 7606678 | SNV | C | T | 1 | 11515 | Hetero | 31 | 231 | 13.4 | 0.42 | 0 | 184 | No | WRAP53 |
| 17 | 18770725 | SNV | A | G | 1 | 11515 | Hetero | 20 | 145 | 13.8 | 0.26 | 0 | 147 | - | PRPSAP2 |
| 17 | 19564470 | SNV | C | T | 1 | 11515 | Hetero | 12 | 112 | 10.7 | 0.14 | 0 | 98 | Yes | ALDH3A2 |
| 17 | 42225693 | SNV | G | T | 1 | 11515 | Hetero | 12 | 113 | 10.6 | 0.36 | 0 | 92 | No | C17orf53 |
| 17 | 29647361 | Insertion | - | TA | 2 | 11515 | Hetero | 17 | 164 | 10.4 | 0.11 | 0 | 150 | - | NF1 |
| 18 | 6896551 | SNV | G | A | 1 | 11515 | Hetero | 7 | 57 | 12.3 | 0.43 | 0 | 60 | No | ARHGAP28 |
| 18 | 6985448 | SNV | C | A | 1 | 11515 | Hetero | 15 | 50 | 30.0 | 0.38 | 0 | 55 | - | LAMA1 |
| 18 | 56203550 | SNV | G | C | 1 | 11515 | Hetero | 16 | 125 | 12.8 | 0.13 | 0 | 121 | Yes | ALPK2 |
| 18 | 59477506 | Deletion | GT | - | 2 | 11515 | Hetero | 5 | 43 | 11.6 | 0.20 | 0 | 47 | - | RNF152 |
| 19 | 3623840 | SNV | G | A | 1 | 11515 | Hetero | 14 | 136 | 10.3 | 0.44 | 0 | 122 | Yes | CACTIN |
| 19 | 13397420 | SNV | G | A | 1 | 11515 | Hetero | 19 | 149 | 12.8 | 0.33 | 0 | 153 | No | CACNA1A |
| 19 | 55512107 | SNV | T | C | 1 | 11515 | Hetero | 25 | 232 | 10.8 | 0.41 | 0 | 175 | - | NLRP2 |
| 20 | 23584291 | SNV | G | A | 1 | 11515 | Hetero | 24 | 221 | 10.9 | 0.48 | 0 | 193 | No | CST9 |
| 20 | 40113069 | SNV | T | C | 1 | 11515 | Hetero | 15 | 106 | 14.2 | 0.29 | 0 | 87 | Yes | CHD6 |
| 20 | 48494444 | SNV | C | T | 1 | 11515 | Hetero | 18 | 95 | 18.9 | 0.14 | 0 | 52 | - | SLC9A8 |
| 21 | 22370878 | SNV | G | T | 1 | 11515 | Hetero | 4 | 38 | 10.5 | 0.33 | 0 | 42 | - | NCAM2 |
| 22 | 24199260 | SNV | T | G | 1 | 11515 | Hetero | 10 | 43 | 23.3 | 0.10 | 0 | 45 | - | SLC2A11 |
| 22 | 26346311 | Insertion | - | T | 1 | 11515 | Hetero | 8 | 48 | 16.7 | 0.13 | 0 | 46 | - | MYO18B |
| X | 38128632 | SNV | G | C | 1 | 11515 | Hetero | 11 | 56 | 19.6 | 0.21 | 0 | 40 | - | RP5-972B16.2 |
| X | 71492068 | SNV | G | A | 1 | 11515 | Hetero | 9 | 75 | 12.0 | 0.27 | 0 | 49 | - | PIN4 |
| X | 125954919 | SNV | G | A | 1 | 11515 | Hetero | 15 | 64 | 23.4 | 0.44 | 0 | 52 | Yes | CXorf64 |
| X | 152846545 | SNV | T | G | 1 | 11515 | Hetero | 7 | 65 | 10.8 | 0.25 | 0 | 34 | - | ATP2B3 |
| X | 2732356 | Insertion | - | T | 1 | 11515 | Hetero | 6 | 56 | 10.7 | 0.33 | 0 | 33 | - | XG |
| X | 30269191 | Insertion | - | T | 1 | 11515 | Hetero | 12 | 119 | 10.1 | 0.33 | 0 | 114 | Insertion | MAGEB1 |
| 1 | 3354992 | SNV | G | T | 1 | 22739 | Hetero | 34 | 73 | 46.6 | 0.49 | 0 | 36 | - | PRDM16 |
| 1 | 17086177 | SNV | G | A | 1 | 22739 | Hetero | 8 | 40 | 20.0 | 0.20 | 0 | 50 | No | MST1P9 |
| 1 | 38077855 | SNV | A | C | 1 | 22739 | Hetero | 24 | 60 | 40.0 | 0.38 | 0 | 34 | - | RSPO1 |
| 1 | 50659425 | SNV | G | T | 1 | 22739 | Hetero | 36 | 107 | 33.6 | 0.20 | 0 | 65 | - | ELAVL4 |
| 1 | 64015343 | SNV | C | A | 1 | 22739 | Hetero | 75 | 181 | 41.4 | 0.30 | 0 | 92 | - | ITGB3BP |
| 1 | 67466210 | SNV | T | G | 1 | 22739 | Hetero | 30 | 85 | 35.3 | 0.47 | 0 | 37 | - | SLC35D1 |
| 1 | 100214122 | SNV | A | C | 1 | 22739 | Hetero | 64 | 159 | 40.3 | 0.25 | 0 | 80 | - | FRRS1 |
| 1 | 103385976 | SNV | C | T | 1 | 22739 | Hetero | 47 | 130 | 36.2 | 0.08 | 0 | 67 | - | COL11A1 |
| 1 | 111147553 | SNV | G | C | 1 | 22739 | Hetero | 30 | 92 | 32.6 | 0.40 | 0 | 44 | - | KCNA2 |
| 1 | 151682185 | SNV | G | A | 1 | 22739 | Hetero | 70 | 244 | 28.7 | 0.45 | 0 | 116 | - | CELF3 |
| 1 | 152085039 | SNV | C | T | 1 | 22739 | Hetero | 63 | 547 | 11.5 | 0.47 | 0 | 281 | No | TCHH |
| 1 | 201438258 | SNV | T | C | 1 | 22739 | Hetero | 37 | 153 | 24.2 | 0.38 | 0 | 93 | - | PHLDA3 |
| 2 | 15567870 | SNV | G | C | 1 | 22739 | Hetero | 13 | 79 | 16.5 | 0.27 | 0 | 49 | Yes | NBAS |
| 2 | 71740372 | SNV | G | A | 1 | 22739 | Hetero | 43 | 129 | 33.3 | 0.26 | 0 | 87 | Yes | DYSF |
| 2 | 73453410 | SNV | T | C | 1 | 22739 | Hetero | 45 | 121 | 37.2 | 0.19 | 0 | 57 | - | SMYD5 |
| 2 | 99012938 | SNV | G | A | 1 | 22739 | Hetero | 55 | 142 | 38.7 | 0.48 | 0 | 71 | No | CNGA3 |
| 2 | 103324619 | SNV | G | A | 1 | 22739 | Hetero | 33 | 69 | 47.8 | 0.23 | 0 | 35 | Yes | SLC9A2 |
| 2 | 103379051 | SNV | G | C | 1 | 22739 | Hetero | 76 | 237 | 32.1 | 0.42 | 0 | 109 | Yes | TMEM182 |
| 2 | 109930171 | SNV | G | A | 1 | 22739 | Hetero | 43 | 112 | 38.4 | 0.37 | 0 | 58 | - | SH3RF3 |
| 2 | 116539961 | SNV | C | T | 1 | 22739 | Hetero | 27 | 130 | 20.8 | 0.43 | 0 | 62 | Yes | DPP10 |
| 2 | 152658172 | SNV | T | C | 1 | 22739 | Hetero | 80 | 236 | 33.9 | 0.41 | 0 | 100 | - | ARL5A |
| 2 | 172947143 | SNV | A | G | 1 | 22739 | Hetero | 20 | 71 | 28.2 | 0.33 | 0 | 35 | - | METAP1D |
| 2 | 186654338 | SNV | T | C | 1 | 22739 | Hetero | 76 | 174 | 43.7 | 0.40 | 0 | 102 | No | FSIP2 |
| 2 | 212243953 | Deletion | T | - | 1 | 22739 | Hetero | 10 | 85 | 11.8 | 0.50 | 0 | 48 | - | ERBB4 |
| 2 | 223423323 | SNV | G | A | 1 | 22739 | Hetero | 109 | 264 | 41.3 | 0.50 | 0 | 123 | No | SGPP2 |
| 2 | 238681587 | SNV | A | G | 1 | 22739 | Hetero | 64 | 188 | 34.0 | 0.44 | 0 | 82 | Possible splice site disruption | LRRFIP1 |
| 2 | 241976125 | SNV | G | A | 1 | 22739 | Hetero | 53 | 161 | 32.9 | 0.13 | 0 | 71 | - | SNED1 |
| 2 | 242170161 | SNV | C | A | 1 | 22739 | Hetero | 83 | 261 | 31.8 | 0.34 | 0 | 128 | - | HDLBP |
| 2 | 242650874 | SNV | T | C | 1 | 22739 | Hetero | 45 | 107 | 42.1 | 0.29 | 0 | 48 | Yes | ING5 |
| 2 | 20836787 | Insertion | - | G | 1 | 22739 | Hetero | 21 | 72 | 29.2 | 0.43 | 0 | 48 | - | HS1BP3 |
| 2 | 38971040 | Insertion | - | TT | 2 | 22739 | Hetero | 11 | 86 | 12.8 | 0.50 | 0 | 36 | - | SRSF7 |
| 3 | 38024978 | SNV | C | T | 1 | 22739 | Hetero | 158 | 216 | 73.1 | 0.47 | 0 | 99 | - | CTDSPL |
| 3 | 38141911 | SNV | C | G | 1 | 22739 | Hetero | 69 | 95 | 72.6 | 0.36 | 0 | 44 | No | DLEC1 |
| 3 | 47098933 | SNV | T | C | 1 | 22739 | Hetero | 135 | 183 | 73.8 | 0.35 | 0 | 87 | Yes | SETD2 |
| 3 | 112545961 | SNV | G | A | 1 | 22739 | Hetero | 24 | 106 | 22.6 | 0.40 | 0 | 43 | No | CD200R1L |
| 3 | 140698382 | SNV | C | T | 1 | 22739 | Hetero | 60 | 227 | 26.4 | 0.44 | 0 | 68 | - | SLC25A36 |
| 3 | 142840554 | SNV | G | A | 1 | 22739 | Hetero | 47 | 212 | 22.2 | 0.46 | 0 | 63 | Yes | CHST2 |
| 3 | 183542984 | SNV | G | T | 1 | 22739 | Hetero | 230 | 359 | 64.1 | 0.46 | 0 | 89 | Yes | MAP6D1 |
| 3 | 196555291 | SNV | C | T | 1 | 22739 | Hetero | 71 | 115 | 61.7 | 0.32 | 0 | 33 | - | PAK2 |
| 3 | 12571420 | Insertion | - | T | 1 | 22739 | Hetero | 9 | 86 | 10.5 | 0.30 | 0 | 49 | - | TSEN2 |
| 3 | 183963360 | Insertion | - | A | 1 | 22739 | Hetero | 139 | 247 | 56.3 | 0.48 | 0 | 92 | Insertion | EIF2B5 |
| 3 | 52734580 | Insertion | - | T | 1 | 22739 | Hetero | 11 | 99 | 11.1 | 0.50 | 0 | 48 | - | GLT8D1 |
| 3 | 57546766 | Insertion | - | T | 1 | 22739 | Hetero | 11 | 83 | 13.3 | 0.38 | 0 | 46 | - | PDE12 |
| 4 | 6050673 | SNV | G | T | 1 | 22739 | Hetero | 65 | 213 | 30.5 | 0.49 | 0 | 124 | - | JAKMIP1 |
| 4 | 48592719 | SNV | T | C | 1 | 22739 | Hetero | 31 | 114 | 27.2 | 0.09 | 0 | 43 | Yes | FRYL |
| 4 | 70715184 | SNV | T | G | 1 | 22739 | Hetero | 93 | 267 | 34.8 | 0.45 | 0 | 126 | Yes | SULT1E1 |
| 4 | 79465219 | SNV | A | G | 1 | 22739 | Hetero | 45 | 112 | 40.2 | 0.33 | 0 | 51 | - | FRAS1 |
| 4 | 119177793 | SNV | C | T | 1 | 22739 | Hetero | 40 | 106 | 37.7 | 0.48 | 0 | 49 | - | NDST3 |
| 4 | 153897650 | SNV | G | A | 1 | 22739 | Hetero | 25 | 215 | 11.6 | 0.22 | 0 | 150 | No | FHDC1 |
| 4 | 147563009 | Deletion | AC | - | 2 | 22739 | Hetero | 4 | 39 | 10.3 | 0.50 | 0 | 30 | - | POU4F2 |
| 5 | 10265972 | SNV | A | G | 1 | 22739 | Hetero | 29 | 75 | 38.7 | 0.47 | 0 | 39 | - | CCT5 |
| 5 | 33683155 | SNV | C | A | 1 | 22739 | Hetero | 99 | 215 | 46.0 | 0.39 | 0 | 106 | Yes | ADAMTS12 |
| 5 | 35654760 | SNV | C | T | 1 | 22739 | Hetero | 216 | 632 | 34.2 | 0.48 | 0 | 298 | Yes | SPEF2 |
| 5 | 138700307 | SNV | C | T | 1 | 22739 | Hetero | 55 | 110 | 50.0 | 0.32 | 0 | 115 | No | PAIP2 |
| 5 | 151188982 | SNV | A | G | 1 | 22739 | Hetero | 23 | 129 | 17.8 | 0.44 | 0 | 68 | - | G3BP1 |
| 5 | 177574801 | SNV | G | A | 1 | 22739 | Hetero | 35 | 255 | 13.7 | 0.46 | 0 | 233 | No | RMND5B |
| 5 | 177673260 | SNV | C | T | 1 | 22739 | Hetero | 36 | 69 | 52.2 | 0.13 | 0 | 48 | Yes | COL23A1 |
| 5 | 179156481 | SNV | T | C | 1 | 22739 | Hetero | 47 | 88 | 53.4 | 0.41 | 0 | 59 | - | CANX |
| 5 | 179706988 | SNV | G | T | 1 | 22739 | Hetero | 43 | 91 | 47.3 | 0.37 | 0 | 55 | No | MAPK9 |
| 5 | 106715248 | Insertion | - | A | 1 | 22739 | Hetero | 7 | 52 | 13.5 | 0.25 | 0 | 37 | - | EFNA5 |
| 5 | 180046417 | Insertion | - | AG | 2 | 22739 | Hetero | 8 | 62 | 12.9 | 0.22 | 0 | 62 | - | FLT4 |
| 6 | 11138942 | SNV | A | G | 1 | 22739 | Hetero | 32 | 85 | 37.6 | 0.38 | 0 | 54 | - | C6orf228 |
| 6 | 17609273 | SNV | G | T | 1 | 22739 | Hetero | 36 | 92 | 39.1 | 0.38 | 0 | 46 | - | FAM8A1 |
| 6 | 27108152 | SNV | C | T | 1 | 22739 | Hetero | 39 | 108 | 36.1 | 0.40 | 0 | 53 | - | HIST1H2BK |
| 6 | 27277471 | SNV | C | T | 1 | 22739 | Hetero | 38 | 97 | 39.2 | 0.41 | 0 | 54 | Yes | POM121L2 |
| 6 | 32062886 | SNV | G | A | 1 | 22739 | Hetero | 25 | 197 | 12.7 | 0.23 | 0 | 95 | No | TNXB |
| 6 | 33179186 | SNV | G | A | 1 | 22739 | Hetero | 44 | 105 | 41.9 | 0.32 | 0 | 62 | Yes | RING1 |
| 6 | 37186327 | SNV | G | A | 1 | 22739 | Hetero | 90 | 225 | 40.0 | 0.30 | 0 | 115 | No | TMEM217 |
| 6 | 70048837 | SNV | C | T | 1 | 22739 | Hetero | 88 | 209 | 42.1 | 0.33 | 0 | 109 | Yes | BAI3 |
| 6 | 82935256 | SNV | G | A | 1 | 22739 | Hetero | 61 | 171 | 35.7 | 0.18 | 0 | 84 | No | IBTK |
| 6 | 83076816 | SNV | T | C | 1 | 22739 | Hetero | 63 | 136 | 46.3 | 0.42 | 0 | 41 | - | TPBG |
| 6 | 100833989 | SNV | T | G | 1 | 22739 | Hetero | 28 | 87 | 32.2 | 0.50 | 0 | 53 | - | SIM1 |
| 6 | 112486351 | SNV | T | A | 1 | 22739 | Hetero | 32 | 100 | 32.0 | 0.16 | 0 | 39 | - | LAMA4 |
| 6 | 127608544 | SNV | T | C | 1 | 22739 | Hetero | 78 | 219 | 35.6 | 0.41 | 0 | 125 | No | RNF146 |
| 6 | 131179228 | SNV | G | T | 1 | 22739 | Hetero | 63 | 462 | 13.6 | 0.32 | 0 | 267 | - | EPB41L2 |
| 6 | 146875972 | SNV | A | G | 1 | 22739 | Hetero | 30 | 74 | 40.5 | 0.32 | 0 | 38 | - | RAB32 |
| 6 | 158097180 | SNV | G | A | 1 | 22739 | Hetero | 14 | 116 | 12.1 | 0.50 | 0 | 96 | - | ZDHHC14 |
| 7 | 51096273 | SNV | G | A | 1 | 22739 | Hetero | 128 | 305 | 42.0 | 0.48 | 0 | 173 | No | COBL |
| 7 | 101895359 | SNV | A | G | 1 | 22739 | Hetero | 22 | 65 | 33.8 | 0.40 | 0 | 31 | - | CUX1 |
| 7 | 135269671 | SNV | A | G | 1 | 22739 | Hetero | 63 | 160 | 39.4 | 0.34 | 0 | 87 | No | NUP205 |
| 7 | 138305706 | SNV | G | A | 1 | 22739 | Hetero | 38 | 79 | 48.1 | 0.36 | 0 | 42 | - | SVOPL |
| 7 | 26252324 | Insertion | - | T | 1 | 22739 | Hetero | 76 | 332 | 22.9 | 0.40 | 0 | 180 | - | CBX3 |
| 8 | 10966289 | SNV | G | A | 1 | 22739 | Hetero | 60 | 169 | 35.5 | 0.46 | 0 | 103 | - | XKR6 |
| 8 | 17739639 | SNV | C | T | 1 | 22739 | Hetero | 60 | 151 | 39.7 | 0.48 | 0 | 78 | Yes | FGL1 |
| 8 | 49641711 | SNV | G | A | 1 | 22739 | Hetero | 40 | 94 | 42.6 | 0.26 | 0 | 59 | - | AC026904.1 |
| 8 | 76456139 | SNV | A | G | 1 | 22739 | Hetero | 29 | 96 | 30.2 | 0.39 | 0 | 70 | Yes | HNF4G |
| 8 | 76478276 | Deletion | A | - | 1 | 22739 | Hetero | 15 | 65 | 23.1 | 0.47 | 0 | 37 | - | HNF4G |
| 8 | 97270680 | SNV | T | C | 1 | 22739 | Hetero | 83 | 199 | 41.7 | 0.27 | 0 | 123 | Yes | MTERFD1 |
| 8 | 103324407 | SNV | T | A | 1 | 22739 | Hetero | 21 | 104 | 20.2 | 0.24 | 0 | 70 | Yes | UBR5 |
| 8 | 105361447 | SNV | G | C | 1 | 22739 | Hetero | 70 | 179 | 39.1 | 0.42 | 0 | 75 | Yes | DCSTAMP |
| 8 | 125565700 | SNV | G | A | 1 | 22739 | Hetero | 37 | 282 | 13.1 | 0.49 | 0 | 126 | Yes | NDUFB9 |
| 8 | 144734746 | SNV | C | T | 1 | 22739 | Hetero | 48 | 115 | 41.7 | 0.47 | 0 | 63 | - | ZNF623 |
| 8 | 95718131 | Insertion | - | TT | 2 | 22739 | Hetero | 13 | 115 | 11.3 | 0.20 | 0 | 63 | - | ESRP1 |
| 9 | 32543709 | SNV | T | A | 1 | 22739 | Hetero | 104 | 183 | 56.8 | 0.38 | 0 | 163 | Yes | TOPORS |
| 9 | 80412544 | SNV | C | A | 1 | 22739 | Hetero | 23 | 85 | 27.1 | 0.48 | 0 | 37 | Yes | GNAQ |
| 9 | 107380207 | SNV | G | C | 1 | 22739 | Hetero | 34 | 230 | 14.8 | 0.42 | 0 | 140 | No | OR13C9 |
| 9 | 111849513 | SNV | T | A | 1 | 22739 | Hetero | 56 | 153 | 36.6 | 0.34 | 0 | 74 | No | TMEM245 |
| 9 | 130127596 | SNV | T | C | 1 | 22739 | Hetero | 63 | 280 | 22.5 | 0.47 | 0 | 160 | No | GARNL3 |
| 9 | 130550529 | SNV | A | T | 1 | 22739 | Hetero | 66 | 202 | 32.7 | 0.49 | 0 | 123 | Yes | CDK9 |
| 9 | 139906909 | SNV | C | T | 1 | 22739 | Hetero | 57 | 186 | 30.6 | 0.42 | 0 | 110 | - | ABCA2 |
| 10 | 6060008 | SNV | A | T | 1 | 22739 | Hetero | 31 | 75 | 41.3 | 0.44 | 0 | 43 | - | IL2RA |
| 10 | 7797851 | SNV | T | C | 1 | 22739 | Hetero | 25 | 82 | 30.5 | 0.34 | 0 | 48 | - | KIN |
| 10 | 11374627 | SNV | G | A | 1 | 22739 | Hetero | 13 | 74 | 17.6 | 0.31 | 0 | 41 | - | CELF2 |
| 10 | 50665515 | SNV | T | A | 1 | 22739 | Hetero | 24 | 61 | 39.3 | 0.33 | 0 | 44 | - | ERCC6 |
| 10 | 50665517 | SNV | C | A | 1 | 22739 | Hetero | 23 | 61 | 37.7 | 0.32 | 0 | 42 | - | ERCC6 |
| 10 | 120450798 | SNV | A | C | 1 | 22739 | Hetero | 22 | 72 | 30.6 | 0.32 | 0 | 53 | Yes | CACUL1 |
| 10 | 133770472 | SNV | C | T | 1 | 22739 | Hetero | 50 | 131 | 38.2 | 0.42 | 0 | 56 | - | PPP2R2D |
| 10 | 135027475 | SNV | G | A | 1 | 22739 | Hetero | 58 | 159 | 36.5 | 0.34 | 0 | 78 | - | KNDC1 |
| 10 | 135044755 | SNV | C | G | 1 | 22739 | Hetero | 30 | 81 | 37.0 | 0.26 | 0 | 55 | Yes | UTF1 |
| 11 | 8124341 | SNV | C | T | 1 | 22739 | Hetero | 34 | 94 | 36.2 | 0.35 | 0 | 63 | - | TUB |
| 11 | 62290149 | SNV | C | A | 1 | 22739 | Hetero | 50 | 321 | 15.6 | 0.36 | 0 | 145 | Yes | AHNAK |
| 11 | 64949921 | SNV | G | A | 1 | 22739 | Hetero | 50 | 326 | 15.3 | 0.28 | 0 | 82 | - | CAPN1 |
| 11 | 76237603 | SNV | G | A | 1 | 22739 | Hetero | 374 | 552 | 67.8 | 0.39 | 0 | 151 | Yes | C11orf30 |
| 11 | 86198530 | Deletion | T | - | 1 | 22739 | Hetero | 14 | 132 | 10.6 | 0.38 | 0 | 45 | - | RP11-317J19.1 |
| 11 | 131781225 | SNV | T | G | 1 | 22739 | Hetero | 165 | 257 | 64.2 | 0.50 | 0 | 245 | - | NTM |
| 11 | 111745484 | Insertion | - | AA | 2 | 22739 | Hetero | 9 | 84 | 10.7 | 0.11 | 0 | 67 | - | RP11-108O10.8 |
| 12 | 32793270 | SNV | A | T | 1 | 22739 | Hetero | 43 | 109 | 39.4 | 0.46 | 0 | 52 | Yes | FGD4 |
| 12 | 41316240 | SNV | C | A | 1 | 22739 | Hetero | 93 | 235 | 39.6 | 0.32 | 0 | 133 | - | CNTN1 |
| 12 | 56619229 | SNV | A | T | 1 | 22739 | Hetero | 73 | 186 | 39.2 | 0.47 | 0 | 71 | Yes | NABP2 |
| 12 | 105443764 | SNV | A | T | 1 | 22739 | Hetero | 48 | 130 | 36.9 | 0.50 | 0 | 76 | Yes | ALDH1L2 |
| 12 | 113818667 | SNV | C | T | 1 | 22739 | Hetero | 67 | 172 | 39.0 | 0.49 | 0 | 103 | - | PLBD2 |
| 12 | 119600266 | SNV | A | G | 1 | 22739 | Hetero | 6 | 55 | 10.9 | 0.17 | 0 | 43 | - | SRRM4 |
| 12 | 119617470 | SNV | A | G | 1 | 22739 | Hetero | 43 | 115 | 37.4 | 0.29 | 0 | 85 | Yes | HSPB8 |
| 12 | 125473391 | SNV | G | A | 1 | 22739 | Hetero | 131 | 341 | 38.4 | 0.36 | 0 | 186 | - | DHX37 |
| 12 | 93792934 | Insertion | - | T | 1 | 22739 | Hetero | 37 | 108 | 34.3 | 0.31 | 0 | 60 | - | NUDT4 |
| 13 | 28222487 | SNV | C | T | 1 | 22739 | Hetero | 71 | 217 | 32.7 | 0.44 | 0 | 125 | - | POLR1D |
| 13 | 30096563 | SNV | C | T | 1 | 22739 | Hetero | 61 | 191 | 31.9 | 0.30 | 0 | 106 | No | SLC7A1 |
| 13 | 46727083 | SNV | G | A | 1 | 22739 | Hetero | 31 | 79 | 39.2 | 0.39 | 0 | 34 | - | LCP1 |
| 13 | 108884659 | SNV | C | G | 1 | 22739 | Hetero | 34 | 93 | 36.6 | 0.39 | 0 | 42 | - | ABHD13 |
| 13 | 43491778 | Insertion | - | A | 1 | 22739 | Hetero | 10 | 63 | 15.9 | 0.10 | 0 | 31 | - | EPSTI1 |
| 13 | 52988307 | Deletion | GA | - | 2 | 22739 | Hetero | 7 | 62 | 11.3 | 0.13 | 0 | 32 | - | VPS36 |
| 14 | 23450556 | SNV | G | T | 1 | 22739 | Hetero | 232 | 300 | 77.3 | 0.49 | 0 | 158 | Yes | AJUBA |
| 14 | 23451684 | SNV | G | C | 1 | 22739 | Hetero | 97 | 124 | 78.2 | 0.45 | 0 | 61 | - | AJUBA |
| 14 | 23456445 | SNV | G | A | 1 | 22739 | Hetero | 220 | 292 | 75.3 | 0.48 | 0 | 152 | No | C14orf93 |
| 14 | 90865337 | SNV | A | T | 1 | 22739 | Hetero | 107 | 265 | 40.4 | 0.30 | 0 | 153 | - | CALM1 |
| 14 | 29261307 | Insertion | - | A | 1 | 22739 | Hetero | 26 | 231 | 11.3 | 0.43 | 0 | 136 | Insertion | C14orf23 |
| 14 | 53241309 | Insertion | - | T | 1 | 22739 | Hetero | 6 | 45 | 13.3 | 0.43 | 0 | 34 | - | STYX |
| 15 | 22383400 | SNV | G | T | 1 | 22739 | Hetero | 14 | 135 | 10.4 | 0.47 | 0 | 56 | Yes | RP11-69H14.6 |
| 15 | 41060229 | SNV | C | A | 1 | 22739 | Hetero | 47 | 120 | 39.2 | 0.48 | 0 | 65 | Yes | DNAJC17 |
| 15 | 42191705 | Deletion | A | - | 1 | 22739 | Hetero | 7 | 68 | 10.3 | 0.50 | 0 | 33 | - | EHD4 |
| 15 | 43769914 | SNV | T | A | 1 | 22739 | Hetero | 58 | 140 | 41.4 | 0.36 | 0 | 72 | Yes | TP53BP1 |
| 15 | 71144406 | SNV | C | A | 1 | 22739 | Hetero | 42 | 129 | 32.6 | 0.37 | 0 | 79 | - | LARP6 |
| 15 | 88416083 | Deletion | A | - | 1 | 22739 | Hetero | 21 | 73 | 28.8 | 0.46 | 0 | 36 | - | NTRK3 |
| 15 | 60786826 | Insertion | - | A | 1 | 22739 | Hetero | 22 | 68 | 32.4 | 0.48 | 0 | 40 | - | RORA |
| 16 | 2246432 | SNV | A | G | 1 | 22739 | Hetero | 25 | 93 | 26.9 | 0.48 | 0 | 41 | Yes | CASKIN1 |
| 16 | 20871378 | SNV | A | G | 1 | 22739 | Hetero | 53 | 145 | 36.6 | 0.47 | 0 | 65 | Yes | ERI2 |
| 16 | 24228934 | SNV | C | A | 1 | 22739 | Hetero | 27 | 85 | 31.8 | 0.44 | 0 | 51 | - | PRKCB |
| 16 | 28855602 | SNV | C | A | 1 | 22739 | Hetero | 122 | 352 | 34.7 | 0.46 | 0 | 209 | Yes | TUFM |
| 17 | 7577085 | SNV | C | T | 1 | 22739 | Hetero | 75 | 204 | 36.8 | 0.17 | 0 | 113 | Yes | TP53 |
| 17 | 8052532 | Deletion | A | - | 1 | 22739 | Hetero | 26 | 121 | 21.5 | 0.21 | 0 | 43 | - | PER1 |
| 17 | 16665011 | SNV | C | T | 1 | 22739 | Hetero | 32 | 110 | 29.1 | 0.28 | 0 | 82 | No | CCDC144A |
| 17 | 29051355 | SNV | T | C | 1 | 22739 | Hetero | 33 | 81 | 40.7 | 0.45 | 0 | 44 | - | SUZ12P |
| 17 | 57057810 | SNV | C | A | 1 | 22739 | Hetero | 88 | 200 | 44.0 | 0.43 | 0 | 104 | Yes | PPM1E |
| 17 | 58024469 | SNV | A | G | 1 | 22739 | Hetero | 121 | 332 | 36.4 | 0.44 | 0 | 188 | - | RPS6KB1 |
| 17 | 62496718 | SNV | T | A | 1 | 22739 | Hetero | 51 | 130 | 39.2 | 0.44 | 0 | 65 | Yes | DDX5 |
| 17 | 76671609 | SNV | C | A | 1 | 22739 | Hetero | 103 | 294 | 35.0 | 0.19 | 0 | 125 | - | CYTH1 |
| 17 | 80560667 | SNV | T | C | 1 | 22739 | Hetero | 46 | 111 | 41.4 | 0.35 | 0 | 59 | - | FOXK2 |
| 17 | 29706036 | Insertion | - | T | 1 | 22739 | Hetero | 81 | 206 | 39.3 | 0.29 | 0 | 97 | - | NF1 |
| 18 | 9258894 | SNV | G | A | 1 | 22739 | Hetero | 78 | 201 | 38.8 | 0.33 | 0 | 96 | Yes | ANKRD12 |
| 18 | 47017658 | SNV | T | A | 1 | 22739 | Hetero | 43 | 142 | 30.3 | 0.45 | 0 | 57 | - | RPL17 |
| 18 | 60223485 | SNV | C | T | 1 | 22739 | Hetero | 105 | 282 | 37.2 | 0.43 | 0 | 119 | Yes | ZCCHC2 |
| 19 | 7605055 | SNV | A | G | 1 | 22739 | Hetero | 34 | 99 | 34.3 | 0.47 | 0 | 42 | Yes | PNPLA6 |
| 19 | 33444604 | SNV | C | G | 1 | 22739 | Hetero | 54 | 230 | 23.5 | 0.05 | 0 | 136 | Yes | CEP89 |
| 19 | 36939498 | SNV | T | C | 1 | 22739 | Hetero | 72 | 156 | 46.2 | 0.44 | 0 | 64 | - | ZNF566 |
| 19 | 46878953 | SNV | C | T | 1 | 22739 | Hetero | 53 | 171 | 31.0 | 0.33 | 0 | 73 | No | PPP5C |
| 20 | 4850568 | Insertion | - | G | 1 | 22739 | Hetero | 12 | 76 | 15.8 | 0.17 | 0 | 33 | Insertion | SLC23A2 |
| 21 | 31768806 | SNV | C | T | 1 | 22739 | Hetero | 52 | 141 | 36.9 | 0.37 | 0 | 94 | No | KRTAP13-1 |
| 21 | 44452259 | SNV | C | T | 1 | 22739 | Hetero | 51 | 185 | 27.6 | 0.41 | 0 | 124 | Yes | PKNOX1 |
| 21 | 45177160 | SNV | C | T | 1 | 22739 | Hetero | 32 | 100 | 32.0 | 0.33 | 0 | 56 | - | PDXK |
| 21 | 46644510 | Insertion | - | C | 1 | 22739 | Hetero | 6 | 34 | 17.6 | 0.17 | 0 | 34 | - | ADARB1 |
| 22 | 45592119 | SNV | G | A | 1 | 22739 | Hetero | 44 | 148 | 29.7 | 0.36 | 0 | 82 | - | KIAA0930 |
| X | 10474363 | SNV | C | A | 1 | 22739 | Hetero | 10 | 58 | 17.2 | 0.09 | 0 | 45 | - | MID1 |
| X | 55185682 | SNV | G | T | 1 | 22739 | Hetero | 12 | 43 | 27.9 | 0.08 | 0 | 66 | - | FAM104B |
| X | 73744340 | SNV | T | A | 1 | 22739 | Hetero | 67 | 92 | 72.8 | 0.44 | 0 | 51 | Yes | SLC16A2 |
| X | 103359019 | SNV | C | A | 1 | 22739 | Hetero | 91 | 114 | 79.8 | 0.44 | 0 | 76 | - | SLC25A53 |
| X | 153176590 | SNV | G | A | 1 | 22739 | Hetero | 74 | 102 | 72.5 | 0.50 | 0 | 56 | No | ARHGAP4 |
| 1 | 39470995 | SNV | A | C | 1 | 22780 | Hetero | 7 | 46 | 15.2 | 0.14 | 0 | 35 | - | AKIRIN1 |
| 1 | 223537285 | Insertion | - | AA | 2 | 22780 | Hetero | 7 | 68 | 10.3 | 0.14 | 0 | 62 | - | SUSD4 |
| 1 | 39470979 | Insertion | - | A | 1 | 22780 | Hetero | 6 | 39 | 15.4 | 0.14 | 0 | 30 | - | AKIRIN1 |
| 2 | 220466344 | Insertion | - | TT | 2 | 22780 | Hetero | 9 | 89 | 10.1 | 0.33 | 0 | 85 | - | STK11IP |
| 2 | 27694905 | Insertion | - | A | 1 | 22780 | Hetero | 7 | 66 | 10.6 | 0.43 | 0 | 43 | - | IFT172 |
| 3 | 12624614 | SNV | C | T | 1 | 22780 | Hetero | 7 | 65 | 10.8 | 0.43 | 0 | 64 | - | MKRN2 |
| 3 | 58351676 | SNV | G | T | 1 | 22780 | Hetero | 4 | 34 | 11.8 | 0.25 | 0 | 34 | - | PXK |
| 3 | 58351660 | Insertion | - | T | 1 | 22780 | Hetero | 7 | 40 | 17.5 | 0.25 | 0 | 30 | - | PXK |
| 4 | 77356331 | Deletion | A | - | 1 | 22780 | Hetero | 5 | 40 | 12.5 | 0.33 | 0 | 31 | - | SHROOM3 |
| 5 | 54710072 | Insertion | - | T | 1 | 22780 | Hetero | 5 | 46 | 10.9 | 0.43 | 0 | 46 | - | SKIV2L2 |
| 7 | 100852161 | SNV | G | A | 1 | 22780 | Hetero | 17 | 169 | 10.1 | 0.37 | 0 | 181 | No | PLOD3 |
| 7 | 116963261 | SNV | C | T | 1 | 22780 | Hetero | 18 | 180 | 10.0 | 0.42 | 0 | 178 | - | WNT2 |
| 7 | 149186361 | SNV | C | T | 1 | 22780 | Hetero | 9 | 70 | 12.9 | 0.33 | 0 | 41 | - | ZNF746 |
| 7 | 27221325 | Deletion | AT | - | 2 | 22780 | Hetero | 5 | 50 | 10.0 | 0.20 | 0 | 33 | - | HOXA11 |
| 8 | 24346664 | SNV | A | C | 1 | 22780 | Hetero | 7 | 52 | 13.5 | 0.25 | 0 | 63 | - | ADAM7 |
| 10 | 126715144 | SNV | T | G | 1 | 22780 | Hetero | 9 | 43 | 20.9 | 0.11 | 0 | 59 | No | CTBP2 |
| 11 | 104896762 | SNV | T | A | 1 | 22780 | Hetero | 9 | 79 | 11.4 | 0.44 | 0 | 90 | - | CASP1 |
| 11 | 117075476 | Insertion | - | A | 1 | 22780 | Hetero | 6 | 35 | 17.1 | 0.14 | 0 | 46 | - | TAGLN |
| 12 | 112640757 | Deletion | T | - | 1 | 22780 | Hetero | 5 | 40 | 12.5 | 0.40 | 0 | 43 | - | HECTD4 |
| 13 | 25670984 | Insertion | - | T | 1 | 22780 | Hetero | 24 | 184 | 13.0 | 0.37 | 0 | 215 | Insertion | PABPC3 |
| 14 | 105768483 | Insertion | - | A | 1 | 22780 | Hetero | 3 | 30 | 10.0 | 0.50 | 0 | 40 | - | BRF1 |
| 15 | 49660063 | Insertion | - | A | 1 | 22780 | Hetero | 4 | 34 | 11.8 | 0.50 | 0 | 49 | - | GALK2 |
| 17 | 7255683 | SNV | C | G | 1 | 22780 | Hetero | 30 | 212 | 14.2 | 0.46 | 0 | 217 | - | KCTD11 |
| 17 | 7578275 | SNV | G | A | 1 | 22780 | Hetero | 31 | 183 | 16.9 | 0.46 | 0 | 159 | Yes | TP53 |
| 17 | 7578496 | SNV | A | T | 1 | 22780 | Hetero | 27 | 165 | 16.4 | 0.28 | 0 | 150 | Yes | TP53 |
| 17 | 39262701 | SNV | T | C | 1 | 22780 | Hetero | 6 | 46 | 13.0 | 0.14 | 0 | 68 | - | KRTAP4-9 |
| 17 | 41465920 | SNV | A | C | 1 | 22780 | Hetero | 38 | 148 | 25.7 | 0.26 | 0 | 170 | - | CTD-3014M21.4 |
| 17 | 48438103 | SNV | C | T | 1 | 22780 | Hetero | 9 | 87 | 10.3 | 0.44 | 0 | 72 | - | XYLT2 |
| 19 | 14091586 | SNV | A | T | 1 | 22780 | Hetero | 12 | 93 | 12.9 | 0.08 | 0 | 99 | - | RFX1 |
| 19 | 14091588 | SNV | A | G | 1 | 22780 | Hetero | 11 | 90 | 12.2 | 0.09 | 0 | 93 | - | RFX1 |
| 19 | 44280870 | Deletion | T | - | 1 | 22780 | Hetero | 5 | 48 | 10.4 | 0.17 | 0 | 36 | - | KCNN4 |
| 19 | 633542 | Deletion | GC | - | 2 | 22780 | Hetero | 7 | 42 | 16.7 | 0.14 | 0 | 43 | Deletion | POLRMT |
| X | 56590507 | SNV | C | A | 1 | 22780 | Hetero | 9 | 51 | 17.6 | 0.33 | 0 | 64 | No | UBQLN2 |
| X | 106844590 | SNV | G | A | 1 | 22780 | Hetero | 14 | 120 | 11.7 | 0.40 | 0 | 126 | No | FRMPD3 |
| 1 | 6150391 | SNV | A | T | 1 | 22833 | Hetero | 25 | 66 | 37.9 | 0.38 | 0 | 84 | - | KCNAB2 |
| 1 | 6475332 | Deletion | T | - | 1 | 22833 | Hetero | 8 | 55 | 14.5 | 0.22 | 0 | 32 | - | HES2 |
| 1 | 49051648 | SNV | A | G | 1 | 22833 | Hetero | 19 | 80 | 23.8 | 0.32 | 0 | 65 | - | AGBL4 |
| 1 | 67185033 | SNV | G | T | 1 | 22833 | Hetero | 12 | 111 | 10.8 | 0.38 | 0 | 90 | Yes | SGIP1 |
| 1 | 70694131 | SNV | G | C | 1 | 22833 | Hetero | 32 | 164 | 19.5 | 0.30 | 0 | 127 | Yes | SRSF11 |
| 1 | 156434063 | SNV | T | C | 1 | 22833 | Hetero | 14 | 59 | 23.7 | 0.42 | 0 | 48 | - | MEF2D |
| 1 | 157740495 | SNV | C | T | 1 | 22833 | Hetero | 20 | 143 | 14.0 | 0.48 | 0 | 119 | - | FCRL2 |
| 1 | 157804224 | SNV | C | G | 1 | 22833 | Hetero | 37 | 195 | 19.0 | 0.41 | 0 | 185 | Yes | CD5L |
| 1 | 176811477 | SNV | C | A | 1 | 22833 | Hetero | 24 | 225 | 10.7 | 0.25 | 0 | 136 | - | PAPPA2 |
| 1 | 200826961 | SNV | A | G | 1 | 22833 | Hetero | 22 | 173 | 12.7 | 0.41 | 0 | 163 | Yes | CAMSAP2 |
| 1 | 216678794 | SNV | G | T | 1 | 22833 | Hetero | 9 | 63 | 14.3 | 0.30 | 0 | 63 | - | ESRRG |
| 1 | 237067105 | SNV | A | C | 1 | 22833 | Hetero | 13 | 74 | 17.6 | 0.33 | 0 | 83 | - | MTR |
| 1 | 248264069 | SNV | G | C | 1 | 22833 | Hetero | 7 | 51 | 13.7 | 0.44 | 0 | 69 | - | OR2L13 |
| 2 | 40241 | SNV | T | A | 1 | 22833 | Hetero | 12 | 90 | 13.3 | 0.47 | 0 | 93 | - | FAM110C |
| 2 | 54864956 | SNV | A | G | 1 | 22833 | Hetero | 41 | 236 | 17.4 | 0.28 | 0 | 210 | - | SPTBN1 |
| 2 | 69475561 | SNV | C | T | 1 | 22833 | Hetero | 10 | 81 | 12.3 | 0.42 | 0 | 87 | - | ANTXR1 |
| 2 | 89544984 | SNV | C | A | 1 | 22833 | Hetero | 6 | 49 | 12.2 | 0.33 | 0 | 91 | - | IGKV2-30 |
| 2 | 103013029 | SNV | C | T | 1 | 22833 | Hetero | 9 | 40 | 22.5 | 0.42 | 0 | 32 | Yes | IL18R1 |
| 2 | 105882459 | SNV | C | A | 1 | 22833 | Hetero | 11 | 76 | 14.5 | 0.36 | 0 | 67 | - | TGFBRAP1 |
| 2 | 130988852 | SNV | G | A | 1 | 22833 | Hetero | 26 | 131 | 19.8 | 0.38 | 0 | 119 | - | ENSG00000213225 |
| 2 | 130988964 | SNV | C | T | 1 | 22833 | Hetero | 30 | 133 | 22.6 | 0.39 | 0 | 157 | - | ENSG00000213225 |
| 2 | 133539900 | SNV | G | C | 1 | 22833 | Hetero | 15 | 108 | 13.9 | 0.38 | 0 | 105 | Yes | NCKAP5 |
| 2 | 138435075 | SNV | T | A | 1 | 22833 | Hetero | 7 | 45 | 15.6 | 0.38 | 0 | 47 | - | THSD7B |
| 2 | 149274449 | SNV | A | T | 1 | 22833 | Hetero | 5 | 32 | 15.6 | 0.20 | 0 | 31 | - | MBD5 |
| 2 | 160637490 | SNV | T | C | 1 | 22833 | Hetero | 29 | 87 | 33.3 | 0.07 | 0 | 61 | Yes | CD302 |
| 2 | 162762422 | SNV | C | T | 1 | 22833 | Hetero | 16 | 149 | 10.7 | 0.32 | 0 | 116 | - | SLC4A10 |
| 2 | 171510682 | SNV | G | T | 1 | 22833 | Hetero | 8 | 54 | 14.8 | 0.33 | 0 | 70 | - | MYO3B |
| 2 | 172339345 | SNV | G | A | 1 | 22833 | Hetero | 12 | 89 | 13.5 | 0.23 | 0 | 57 | - | DCAF17 |
| 2 | 179655503 | SNV | C | G | 1 | 22833 | Hetero | 27 | 102 | 26.5 | 0.36 | 0 | 86 | Yes | TTN |
| 2 | 189877157 | SNV | G | T | 1 | 22833 | Hetero | 24 | 84 | 28.6 | 0.42 | 0 | 95 | - | COL3A1 |
| 2 | 211018403 | SNV | C | T | 1 | 22833 | Hetero | 42 | 288 | 14.6 | 0.46 | 0 | 261 | Yes | KANSL1L |
| 2 | 219561737 | SNV | C | T | 1 | 22833 | Hetero | 21 | 139 | 15.1 | 0.38 | 0 | 111 | - | STK36 |
| 2 | 228846229 | SNV | C | G | 1 | 22833 | Hetero | 13 | 93 | 14.0 | 0.47 | 0 | 94 | - | SPHKAP |
| 2 | 242033863 | SNV | G | A | 1 | 22833 | Hetero | 14 | 59 | 23.7 | 0.35 | 0 | 38 | - | SNED1 |
| 2 | 64687147 | Insertion | - | T | 1 | 22833 | Hetero | 6 | 57 | 10.5 | 0.33 | 0 | 31 | - | LGALSL |
| 3 | 1269599 | SNV | G | C | 1 | 22833 | Hetero | 44 | 204 | 21.6 | 0.47 | 0 | 186 | Yes | CNTN6 |
| 3 | 27204020 | SNV | G | C | 1 | 22833 | Hetero | 9 | 84 | 10.7 | 0.45 | 0 | 94 | Yes | NEK10 |
| 3 | 38519342 | SNV | G | C | 1 | 22833 | Hetero | 18 | 75 | 24.0 | 0.29 | 0 | 51 | - | ACVR2B |
| 3 | 46571418 | SNV | G | C | 1 | 22833 | Hetero | 20 | 165 | 12.1 | 0.24 | 0 | 133 | No | LRRC2 |
| 3 | 57322150 | SNV | C | G | 1 | 22833 | Hetero | 14 | 105 | 13.3 | 0.31 | 0 | 104 | - | ASB14 |
| 3 | 121383727 | SNV | C | A | 1 | 22833 | Hetero | 10 | 79 | 12.7 | 0.10 | 0 | 53 | - | GOLGB1 |
| 3 | 127788412 | SNV | G | T | 1 | 22833 | Hetero | 50 | 218 | 22.9 | 0.36 | 0 | 195 | No | SEC61A1 |
| 3 | 129281806 | SNV | G | A | 1 | 22833 | Hetero | 12 | 73 | 16.4 | 0.23 | 0 | 67 | - | PLXND1 |
| 3 | 164908322 | SNV | A | T | 1 | 22833 | Hetero | 11 | 109 | 10.1 | 0.29 | 0 | 103 | Yes | SLITRK3 |
| 3 | 193361838 | SNV | A | T | 1 | 22833 | Hetero | 24 | 234 | 10.3 | 0.50 | 0 | 173 | Yes | OPA1 |
| 3 | 49836020 | Insertion | - | A | 1 | 22833 | Hetero | 9 | 62 | 14.5 | 0.30 | 0 | 53 | - | CDHR4 |
| 3 | 50405014 | Insertion | - | G | 1 | 22833 | Hetero | 18 | 151 | 11.9 | 0.37 | 0 | 140 | - | CACNA2D2 |
| 3 | 75785169 | Insertion | - | T | 1 | 22833 | Hetero | 21 | 102 | 20.6 | 0.26 | 0 | 115 | - | ZNF717 |
| 4 | 5851128 | SNV | C | T | 1 | 22833 | Hetero | 17 | 81 | 21.0 | 0.38 | 0 | 77 | No | CRMP1 |
| 4 | 16198422 | SNV | G | C | 1 | 22833 | Hetero | 17 | 66 | 25.8 | 0.29 | 0 | 51 | - | TAPT1 |
| 4 | 17578828 | SNV | A | C | 1 | 22833 | Hetero | 23 | 206 | 11.2 | 0.35 | 0 | 221 | - | LAP3 |
| 4 | 48530328 | SNV | C | G | 1 | 22833 | Hetero | 14 | 82 | 17.1 | 0.44 | 0 | 60 | Yes | FRYL |
| 4 | 83551713 | SNV | A | G | 1 | 22833 | Hetero | 9 | 74 | 12.2 | 0.50 | 0 | 69 | - | SCD5 |
| 4 | 90169365 | SNV | G | C | 1 | 22833 | Hetero | 15 | 132 | 11.4 | 0.44 | 0 | 118 | Yes | GPRIN3 |
| 4 | 96762163 | SNV | C | G | 1 | 22833 | Hetero | 22 | 198 | 11.1 | 0.50 | 0 | 178 | Yes | PDHA2 |
| 4 | 126328095 | SNV | G | T | 1 | 22833 | Hetero | 13 | 95 | 13.7 | 0.33 | 0 | 95 | Yes | FAT4 |
| 4 | 140811125 | SNV | G | T | 1 | 22833 | Hetero | 4 | 40 | 10.0 | 0.50 | 0 | 61 | Yes | MAML3 |
| 4 | 144542177 | SNV | C | T | 1 | 22833 | Hetero | 20 | 140 | 14.3 | 0.39 | 0 | 132 | - | GUSBP5 |
| 4 | 151207210 | SNV | G | C | 1 | 22833 | Hetero | 9 | 49 | 18.4 | 0.10 | 0 | 48 | - | LRBA |
| 4 | 154623944 | SNV | G | A | 1 | 22833 | Hetero | 7 | 55 | 12.7 | 0.43 | 0 | 51 | - | TLR2 |
| 5 | 9629552 | SNV | A | G | 1 | 22833 | Hetero | 19 | 150 | 12.7 | 0.38 | 0 | 141 | Yes | TAS2R1 |
| 5 | 11397335 | SNV | G | C | 1 | 22833 | Hetero | 8 | 67 | 11.9 | 0.13 | 0 | 46 | - | CTNND2 |
| 5 | 26903836 | SNV | C | T | 1 | 22833 | Hetero | 46 | 260 | 17.7 | 0.49 | 0 | 240 | Yes | CDH9 |
| 5 | 38477915 | SNV | C | A | 1 | 22833 | Hetero | 16 | 86 | 18.6 | 0.45 | 0 | 74 | - | LIFR |
| 5 | 45303836 | SNV | C | G | 1 | 22833 | Hetero | 21 | 199 | 10.6 | 0.48 | 0 | 198 | Yes | HCN1 |
| 5 | 60454094 | SNV | C | G | 1 | 22833 | Hetero | 11 | 85 | 12.9 | 0.25 | 0 | 92 | - | C5orf43 |
| 5 | 76028431 | SNV | C | T | 1 | 22833 | Hetero | 19 | 143 | 13.3 | 0.45 | 0 | 143 | No | F2R |
| 5 | 115785298 | SNV | C | T | 1 | 22833 | Hetero | 8 | 62 | 12.9 | 0.33 | 0 | 50 | - | SEMA6A |
| 5 | 132334545 | SNV | A | G | 1 | 22833 | Hetero | 37 | 244 | 15.2 | 0.40 | 0 | 251 | - | ZCCHC10 |
| 5 | 175812233 | SNV | C | A | 1 | 22833 | Hetero | 8 | 44 | 18.2 | 0.40 | 0 | 57 | Yes | ARL10 |
| 5 | 54274657 | Deletion | AA | - | 2 | 22833 | Hetero | 4 | 37 | 10.8 | 0.50 | 0 | 43 | - | ESM1 |
| 5 | 54710073 | Deletion | TT | - | 2 | 22833 | Hetero | 6 | 51 | 11.8 | 0.17 | 0 | 30 | - | SKIV2L2 |
| 6 | 26507012 | SNV | C | G | 1 | 22833 | Hetero | 14 | 63 | 22.2 | 0.39 | 0 | 52 | Yes | BTN1A1 |
| 6 | 31795590 | SNV | C | G | 1 | 22833 | Hetero | 46 | 315 | 14.6 | 0.46 | 0 | 246 | - | HSPA1B |
| 6 | 39041436 | SNV | G | T | 1 | 22833 | Hetero | 16 | 121 | 13.2 | 0.22 | 0 | 79 | No | GLP1R |
| 6 | 46976833 | SNV | G | A | 1 | 22833 | Hetero | 28 | 257 | 10.9 | 0.33 | 0 | 212 | Yes | GPR110 |
| 6 | 64291048 | Deletion | T | - | 1 | 22833 | Hetero | 15 | 109 | 13.8 | 0.48 | 0 | 84 | - | PTP4A1 |
| 6 | 65622502 | SNV | G | A | 1 | 22833 | Hetero | 46 | 220 | 20.9 | 0.36 | 0 | 165 | Yes | EYS |
| 6 | 73017047 | SNV | G | A | 1 | 22833 | Hetero | 25 | 147 | 17.0 | 0.32 | 0 | 130 | Yes | RIMS1 |
| 6 | 147128817 | SNV | A | C | 1 | 22833 | Hetero | 35 | 185 | 18.9 | 0.35 | 0 | 196 | - | ADGB |
| 6 | 149862597 | SNV | A | G | 1 | 22833 | Hetero | 6 | 58 | 10.3 | 0.33 | 0 | 52 | - | PPIL4 |
| 7 | 44252830 | SNV | T | C | 1 | 22833 | Hetero | 13 | 126 | 10.3 | 0.35 | 0 | 97 | - | YKT6 |
| 7 | 70597954 | SNV | G | A | 1 | 22833 | Hetero | 21 | 133 | 15.8 | 0.50 | 0 | 122 | Yes | WBSCR17 |
| 7 | 87133452 | SNV | T | A | 1 | 22833 | Hetero | 19 | 95 | 20.0 | 0.48 | 0 | 48 | - | ABCB1 |
| 7 | 93125131 | SNV | C | T | 1 | 22833 | Hetero | 29 | 138 | 21.0 | 0.33 | 0 | 106 | - | CALCR |
| 7 | 94050242 | SNV | G | A | 1 | 22833 | Hetero | 18 | 164 | 11.0 | 0.32 | 0 | 124 | - | COL1A2 |
| 7 | 101892500 | SNV | G | A | 1 | 22833 | Hetero | 15 | 112 | 13.4 | 0.13 | 0 | 55 | - | CUX1 |
| 7 | 101892544 | SNV | G | A | 1 | 22833 | Hetero | 16 | 139 | 11.5 | 0.33 | 0 | 65 | - | CUX1 |
| 7 | 102079461 | SNV | G | A | 1 | 22833 | Hetero | 328 | 532 | 61.7 | 0.43 | 0 | 250 | Yes | ORAI2 |
| 7 | 103112410 | SNV | C | T | 1 | 22833 | Hetero | 18 | 133 | 13.5 | 0.44 | 0 | 66 | - | RELN |
| 7 | 117361166 | SNV | A | G | 1 | 22833 | Hetero | 12 | 49 | 24.5 | 0.46 | 0 | 49 | Yes | CTTNBP2 |
| 7 | 131808883 | SNV | C | A | 1 | 22833 | Hetero | 11 | 57 | 19.3 | 0.50 | 0 | 67 | - | PLXNA4 |
| 7 | 137074332 | SNV | C | A | 1 | 22833 | Hetero | 9 | 73 | 12.3 | 0.44 | 0 | 64 | - | DGKI |
| 7 | 75958134 | Insertion | - | CT | 2 | 22833 | Hetero | 4 | 40 | 10.0 | 0.50 | 0 | 41 | - | YWHAG |
| 7 | 98649755 | Insertion | - | A | 1 | 22833 | Hetero | 7 | 69 | 10.1 | 0.13 | 0 | 39 | - | SMURF1 |
| 8 | 30569610 | Deletion | A | - | 1 | 22833 | Hetero | 5 | 41 | 12.2 | 0.40 | 0 | 36 | - | GSR |
| 8 | 66515949 | SNV | T | C | 1 | 22833 | Hetero | 7 | 48 | 14.6 | 0.25 | 0 | 31 | - | ARMC1 |
| 8 | 97269222 | SNV | T | A | 1 | 22833 | Hetero | 12 | 117 | 10.3 | 0.29 | 0 | 79 | Yes | MTERFD1 |
| 8 | 100515033 | SNV | T | C | 1 | 22833 | Hetero | 9 | 60 | 15.0 | 0.25 | 0 | 50 | - | VPS13B |
| 8 | 110588198 | SNV | C | T | 1 | 22833 | Hetero | 12 | 104 | 11.5 | 0.14 | 0 | 102 | Yes | SYBU |
| 8 | 113246680 | SNV | G | T | 1 | 22833 | Hetero | 13 | 100 | 13.0 | 0.31 | 0 | 70 | Yes | CSMD3 |
| 8 | 131797656 | SNV | G | T | 1 | 22833 | Hetero | 9 | 64 | 14.1 | 0.30 | 0 | 42 | No | ADCY8 |
| 8 | 133883697 | SNV | G | C | 1 | 22833 | Hetero | 36 | 210 | 17.1 | 0.30 | 0 | 143 | Yes | TG |
| 8 | 124511709 | Insertion | - | A | 1 | 22833 | Hetero | 8 | 61 | 13.1 | 0.33 | 0 | 50 | - | FBXO32 |
| 8 | 98817552 | Insertion | - | T | 1 | 22833 | Hetero | 6 | 53 | 11.3 | 0.17 | 0 | 33 | - | LAPTM4B |
| 9 | 35105305 | SNV | C | A | 1 | 22833 | Hetero | 14 | 100 | 14.0 | 0.33 | 0 | 94 | No | FAM214B |
| 9 | 37733708 | Deletion | T | - | 1 | 22833 | Hetero | 15 | 136 | 11.0 | 0.40 | 0 | 140 | - | FRMPD1 |
| 9 | 90321402 | SNV | C | A | 1 | 22833 | Hetero | 25 | 211 | 11.8 | 0.43 | 0 | 284 | Yes | DAPK1 |
| 9 | 113704494 | SNV | C | G | 1 | 22833 | Hetero | 18 | 113 | 15.9 | 0.19 | 0 | 108 | - | LPAR1 |
| 9 | 120475176 | SNV | G | A | 1 | 22833 | Hetero | 27 | 168 | 16.1 | 0.39 | 0 | 208 | Yes | TLR4 |
| 9 | 130524639 | SNV | C | A | 1 | 22833 | Hetero | 22 | 126 | 17.5 | 0.30 | 0 | 116 | - | SH2D3C |
| 9 | 130635666 | SNV | C | T | 1 | 22833 | Hetero | 24 | 228 | 10.5 | 0.48 | 0 | 187 | - | AK1 |
| 9 | 77596937 | Insertion | - | A | 1 | 22833 | Hetero | 7 | 69 | 10.1 | 0.38 | 0 | 61 | - | C9orf41 |
| 10 | 28908504 | SNV | A | C | 1 | 22833 | Hetero | 23 | 132 | 17.4 | 0.19 | 0 | 104 | Yes | WAC |
| 10 | 42924579 | SNV | T | A | 1 | 22833 | Hetero | 5 | 49 | 10.2 | 0.20 | 0 | 41 | Yes | CCNYL2 |
| 10 | 73562829 | SNV | G | A | 1 | 22833 | Hetero | 38 | 188 | 20.2 | 0.43 | 0 | 185 | Yes | CDH23 |
| 10 | 93582036 | SNV | C | A | 1 | 22833 | Hetero | 30 | 159 | 18.9 | 0.36 | 0 | 147 | - | TNKS2 |
| 10 | 98719760 | SNV | A | G | 1 | 22833 | Hetero | 13 | 73 | 17.8 | 0.50 | 0 | 76 | - | LCOR |
| 10 | 38117884 | Deletion | AG | - | 2 | 22833 | Hetero | 27 | 118 | 22.9 | 0.50 | 0 | 85 | - | ZNF248 |
| 11 | 17409056 | SNV | G | C | 1 | 22833 | Hetero | 13 | 112 | 11.6 | 0.47 | 0 | 132 | Yes | KCNJ11 |
| 11 | 20057425 | SNV | G | C | 1 | 22833 | Hetero | 11 | 102 | 10.8 | 0.50 | 0 | 114 | - | NAV2 |
| 11 | 31826746 | SNV | C | G | 1 | 22833 | Hetero | 15 | 78 | 19.2 | 0.40 | 0 | 106 | - | PAX6 |
| 11 | 47776034 | SNV | C | A | 1 | 22833 | Hetero | 3 | 30 | 10.0 | 0.25 | 0 | 34 | - | FNBP4 |
| 11 | 57886330 | SNV | T | C | 1 | 22833 | Hetero | 34 | 195 | 17.4 | 0.46 | 0 | 180 | Yes | OR9Q1 |
| 11 | 64497185 | Deletion | C | - | 1 | 22833 | Hetero | 8 | 55 | 14.5 | 0.13 | 0 | 36 | - | RASGRP2 |
| 11 | 64948270 | SNV | C | A | 1 | 22833 | Hetero | 22 | 194 | 11.3 | 0.46 | 0 | 154 | Yes | AP003068.23 |
| 11 | 72549312 | SNV | C | A | 1 | 22833 | Hetero | 5 | 45 | 11.1 | 0.40 | 0 | 32 | - | ATG16L2 |
| 11 | 118344459 | SNV | G | A | 1 | 22833 | Hetero | 12 | 97 | 12.4 | 0.29 | 0 | 100 | Yes | MLL |
| 11 | 134254062 | SNV | G | A | 1 | 22833 | Hetero | 10 | 52 | 19.2 | 0.50 | 0 | 42 | Yes | B3GAT1 |
| 12 | 2101777 | SNV | G | A | 1 | 22833 | Hetero | 9 | 74 | 12.2 | 0.45 | 0 | 59 | - | DCP1B |
| 12 | 6132781 | SNV | G | A | 1 | 22833 | Hetero | 15 | 60 | 25.0 | 0.24 | 0 | 42 | - | VWF |
| 12 | 8630395 | SNV | T | C | 1 | 22833 | Hetero | 11 | 83 | 13.3 | 0.18 | 0 | 60 | - | CLEC6A |
| 12 | 12941247 | SNV | G | T | 1 | 22833 | Hetero | 15 | 118 | 12.7 | 0.45 | 0 | 95 | - | APOLD1 |
| 12 | 19282935 | SNV | C | T | 1 | 22833 | Hetero | 19 | 107 | 17.8 | 0.40 | 0 | 78 | - | PLEKHA5 |
| 12 | 22012501 | SNV | G | A | 1 | 22833 | Hetero | 33 | 143 | 23.1 | 0.38 | 0 | 124 | - | ABCC9 |
| 12 | 26809457 | SNV | C | A | 1 | 22833 | Hetero | 17 | 159 | 10.7 | 0.21 | 0 | 126 | Yes | ITPR2 |
| 12 | 31253938 | SNV | G | T | 1 | 22833 | Hetero | 26 | 219 | 11.9 | 0.34 | 0 | 179 | - | DDX11 |
| 12 | 49580429 | SNV | C | T | 1 | 22833 | Hetero | 27 | 99 | 27.3 | 0.47 | 0 | 113 | Yes | TUBA1A |
| 12 | 53875847 | SNV | G | A | 1 | 22833 | Hetero | 18 | 140 | 12.9 | 0.35 | 0 | 122 | Yes | MAP3K12 |
| 12 | 57993271 | SNV | C | A | 1 | 22833 | Hetero | 14 | 85 | 16.5 | 0.29 | 0 | 56 | - | PIP4K2C |
| 12 | 64437225 | SNV | A | T | 1 | 22833 | Hetero | 3 | 30 | 10.0 | 0.33 | 0 | 38 | - | SRGAP1 |
| 12 | 69962755 | SNV | A | T | 1 | 22833 | Hetero | 26 | 108 | 24.1 | 0.07 | 0 | 122 | - | FRS2 |
| 12 | 100957430 | SNV | G | C | 1 | 22833 | Hetero | 4 | 36 | 11.1 | 0.40 | 0 | 38 | - | NR1H4 |
| 12 | 104344426 | SNV | T | C | 1 | 22833 | Hetero | 10 | 61 | 16.4 | 0.27 | 0 | 68 | - | HSP90B1 |
| 12 | 106723666 | SNV | A | T | 1 | 22833 | Hetero | 16 | 58 | 27.6 | 0.41 | 0 | 51 | - | TCP11L2 |
| 12 | 93167827 | Insertion | - | A | 1 | 22833 | Hetero | 6 | 44 | 13.6 | 0.17 | 0 | 34 | - | EEA1 |
| 12 | 9572812 | Insertion | - | G | 1 | 22833 | Hetero | 4 | 34 | 11.8 | 0.25 | 0 | 36 | - | DDX12P |
| 13 | 19751703 | SNV | A | G | 1 | 22833 | Hetero | 19 | 48 | 39.6 | 0.40 | 0 | 47 | No | TUBA3C |
| 13 | 28130466 | SNV | C | A | 1 | 22833 | Hetero | 9 | 63 | 14.3 | 0.36 | 0 | 63 | Yes | LNX2 |
| 13 | 40326069 | SNV | A | G | 1 | 22833 | Hetero | 8 | 55 | 14.5 | 0.20 | 0 | 73 | - | COG6 |
| 13 | 47325163 | SNV | T | G | 1 | 22833 | Hetero | 8 | 71 | 11.3 | 0.13 | 0 | 77 | - | LRCH1 |
| 13 | 103053897 | SNV | C | T | 1 | 22833 | Hetero | 17 | 128 | 13.3 | 0.43 | 0 | 123 | Yes | FGF14 |
| 14 | 33202698 | SNV | A | T | 1 | 22833 | Hetero | 6 | 58 | 10.3 | 0.29 | 0 | 51 | - | AKAP6 |
| 14 | 104573573 | SNV | G | T | 1 | 22833 | Hetero | 15 | 130 | 11.5 | 0.35 | 0 | 127 | Yes | ASPG |
| 14 | 105612087 | SNV | T | C | 1 | 22833 | Hetero | 21 | 116 | 18.1 | 0.35 | 0 | 88 | Yes | JAG2 |
| 15 | 23002920 | SNV | C | T | 1 | 22833 | Hetero | 12 | 119 | 10.1 | 0.29 | 0 | 120 | No | CYFIP1 |
| 15 | 26793167 | SNV | C | T | 1 | 22833 | Hetero | 17 | 152 | 11.2 | 0.50 | 0 | 182 | Yes | GABRB3 |
| 15 | 45390187 | SNV | C | A | 1 | 22833 | Hetero | 18 | 138 | 13.0 | 0.43 | 0 | 96 | - | DUOX2 |
| 15 | 62994288 | SNV | C | T | 1 | 22833 | Hetero | 9 | 50 | 18.0 | 0.27 | 0 | 58 | No | TLN2 |
| 15 | 98982170 | SNV | C | A | 1 | 22833 | Hetero | 28 | 168 | 16.7 | 0.50 | 0 | 169 | - | FAM169B |
| 15 | 54527275 | Insertion | - | A | 1 | 22833 | Hetero | 34 | 159 | 21.4 | 0.48 | 0 | 148 | Insertion | UNC13C |
| 16 | 3788593 | SNV | A | C | 1 | 22833 | Hetero | 16 | 116 | 13.8 | 0.12 | 0 | 87 | Yes | CREBBP |
| 16 | 30722423 | SNV | G | A | 1 | 22833 | Hetero | 5 | 46 | 10.9 | 0.33 | 0 | 43 | - | SRCAP |
| 16 | 49670204 | SNV | C | A | 1 | 22833 | Hetero | 59 | 262 | 22.5 | 0.36 | 0 | 255 | No | ZNF423 |
| 16 | 71061594 | SNV | G | T | 1 | 22833 | Hetero | 15 | 143 | 10.5 | 0.50 | 0 | 137 | Yes | HYDIN |
| 16 | 79632063 | Deletion | T | - | 1 | 22833 | Hetero | 5 | 41 | 12.2 | 0.50 | 0 | 41 | - | MAF |
| 16 | 86612341 | SNV | C | G | 1 | 22833 | Hetero | 40 | 197 | 20.3 | 0.45 | 0 | 184 | No | FOXL1 |
| 17 | 7579511 | Deletion | C | - | 1 | 22833 | Hetero | 11 | 54 | 20.4 | 0.36 | 0 | 38 | Deletion | TP53 |
| 17 | 9531939 | SNV | C | T | 1 | 22833 | Hetero | 7 | 66 | 10.6 | 0.38 | 0 | 86 | - | WDR16 |
| 17 | 10348071 | SNV | T | C | 1 | 22833 | Hetero | 28 | 124 | 22.6 | 0.41 | 0 | 149 | - | MYH4 |
| 17 | 17164994 | SNV | C | T | 1 | 22833 | Hetero | 11 | 41 | 26.8 | 0.08 | 0 | 35 | Yes | COPS3 |
| 17 | 62498557 | SNV | C | G | 1 | 22833 | Hetero | 23 | 163 | 14.1 | 0.45 | 0 | 124 | Possible splice site disruption | DDX5 |
| 17 | 38990606 | Insertion | - | T | 1 | 22833 | Hetero | 5 | 44 | 11.4 | 0.33 | 0 | 51 | - | TMEM99 |
| 17 | 59001879 | Insertion | - | T | 1 | 22833 | Hetero | 6 | 54 | 11.1 | 0.38 | 0 | 62 | - | BCAS3 |
| 18 | 9958573 | SNV | G | T | 1 | 22833 | Hetero | 7 | 60 | 11.7 | 0.29 | 0 | 44 | - | VAPA |
| 18 | 10670575 | SNV | A | T | 1 | 22833 | Hetero | 16 | 129 | 12.4 | 0.19 | 0 | 65 | - | PIEZO2 |
| 18 | 44584677 | SNV | G | A | 1 | 22833 | Hetero | 11 | 87 | 12.6 | 0.46 | 0 | 67 | Yes | KATNAL2 |
| 18 | 56963372 | Deletion | A | - | 1 | 22833 | Hetero | 24 | 48 | 50.0 | 0.46 | 0 | 95 | - | CPLX4 |
| 18 | 64171051 | SNV | T | A | 1 | 22833 | Hetero | 12 | 75 | 16.0 | 0.40 | 0 | 77 | - | CDH19 |
| 18 | 72264658 | SNV | T | A | 1 | 22833 | Hetero | 10 | 89 | 11.2 | 0.45 | 0 | 108 | - | RP11-231E4.4 |
| 19 | 1487668 | SNV | C | A | 1 | 22833 | Hetero | 24 | 180 | 13.3 | 0.44 | 0 | 171 | Yes | PCSK4 |
| 19 | 1880972 | SNV | C | G | 1 | 22833 | Hetero | 10 | 66 | 15.2 | 0.45 | 0 | 64 | No | FAM108A1 |
| 19 | 10103536 | SNV | G | T | 1 | 22833 | Hetero | 19 | 88 | 21.6 | 0.22 | 0 | 82 | No | COL5A3 |
| 19 | 12878319 | SNV | C | A | 1 | 22833 | Hetero | 11 | 101 | 10.9 | 0.43 | 0 | 80 | - | HOOK2 |
| 19 | 14091572 | Deletion | A | - | 1 | 22833 | Hetero | 10 | 100 | 10.0 | 0.27 | 0 | 90 | - | RFX1 |
| 19 | 14091574 | SNV | A | T | 1 | 22833 | Hetero | 11 | 99 | 11.1 | 0.25 | 0 | 87 | - | RFX1 |
| 19 | 22270973 | SNV | C | A | 1 | 22833 | Hetero | 15 | 71 | 21.1 | 0.33 | 0 | 72 | Yes | ZNF257 |
| 19 | 37210895 | SNV | T | C | 1 | 22833 | Hetero | 38 | 174 | 21.8 | 0.41 | 0 | 142 | No | ZNF567 |
| 19 | 37854659 | SNV | G | C | 1 | 22833 | Hetero | 16 | 153 | 10.5 | 0.42 | 0 | 115 | Yes | HKR1 |
| 19 | 41850850 | SNV | C | A | 1 | 22833 | Hetero | 13 | 71 | 18.3 | 0.07 | 0 | 51 | - | TGFB1 |
| 19 | 50963356 | SNV | G | T | 1 | 22833 | Hetero | 9 | 51 | 17.6 | 0.40 | 0 | 34 | Yes | MYBPC2 |
| 19 | 55812841 | SNV | A | G | 1 | 22833 | Hetero | 16 | 111 | 14.4 | 0.45 | 0 | 90 | - | BRSK1 |
| 20 | 2732401 | SNV | C | T | 1 | 22833 | Hetero | 11 | 107 | 10.3 | 0.46 | 0 | 90 | No | EBF4 |
| 20 | 3726190 | SNV | C | T | 1 | 22833 | Hetero | 19 | 165 | 11.5 | 0.46 | 0 | 157 | No | HSPA12B |
| 20 | 29896263 | SNV | C | A | 1 | 22833 | Hetero | 10 | 85 | 11.8 | 0.42 | 0 | 73 | - | DEFB116 |
| 20 | 44856330 | SNV | C | T | 1 | 22833 | Hetero | 21 | 106 | 19.8 | 0.44 | 0 | 111 | - | CDH22 |
| 20 | 47567965 | SNV | G | T | 1 | 22833 | Hetero | 16 | 125 | 12.8 | 0.43 | 0 | 104 | Yes | ARFGEF2 |
| 20 | 55214255 | SNV | G | T | 1 | 22833 | Hetero | 10 | 73 | 13.7 | 0.36 | 0 | 60 | - | TFAP2C |
| 20 | 35220571 | Insertion | - | A | 1 | 22833 | Hetero | 7 | 57 | 12.3 | 0.43 | 0 | 53 | - | **RP5-977B1.11** |
| 20 | 39314997 | Insertion | - | G | 1 | 22833 | Hetero | 18 | 68 | 26.5 | 0.48 | 0 | 51 | - | MAFB |
| 21 | 47609640 | SNV | A | G | 1 | 22833 | Hetero | 10 | 91 | 11.0 | 0.30 | 0 | 78 | Yes | LSS |
| 22 | 17596167 | SNV | T | G | 1 | 22833 | Hetero | 12 | 51 | 23.5 | 0.15 | 0 | 57 | - | IL17RA |
| 22 | 39826235 | SNV | G | T | 1 | 22833 | Hetero | 49 | 167 | 29.3 | 0.39 | 0 | 101 | - | TAB1 |
| 22 | 39887114 | SNV | C | A | 1 | 22833 | Hetero | 13 | 130 | 10.0 | 0.21 | 0 | 85 | - | TAB1 |
| 22 | 40367768 | SNV | C | A | 1 | 22833 | Hetero | 12 | 78 | 15.4 | 0.33 | 0 | 48 | - | GRAP2 |
| 22 | 33256900 | Insertion | - | T | 1 | 22833 | Hetero | 6 | 46 | 13.0 | 0.33 | 0 | 32 | - | SYN3 |
| X | 18942188 | SNV | A | G | 1 | 22833 | Hetero | 11 | 51 | 21.6 | 0.23 | 0 | 43 | No | PHKA2 |
| X | 77302353 | SNV | C | T | 1 | 22833 | Hetero | 7 | 35 | 20.0 | 0.33 | 0 | 34 | - | ATP7A |
| X | 108867134 | SNV | C | T | 1 | 22833 | Hetero | 13 | 49 | 26.5 | 0.27 | 0 | 47 | - | KCNE1L |
| X | 139796602 | SNV | C | T | 1 | 22833 | Hetero | 18 | 70 | 25.7 | 0.23 | 0 | 83 | - | RP1-177G6.2 |
| X | 152089376 | SNV | G | T | 1 | 22833 | Hetero | 12 | 50 | 24.0 | 0.15 | 0 | 55 | - | ZNF185 |
| 1 | 1117759 | SNV | G | C | 1 | 23658 | Hetero | 37 | 218 | 17.0 | 0.41 | 0 | 281 | Yes | TTLL10 |
| 1 | 1661196 | SNV | G | A | 1 | 23658 | Hetero | 16 | 132 | 12.1 | 0.22 | 0 | 138 | - | RP1-283E3.4 |
| 1 | 10189509 | SNV | A | C | 1 | 23658 | Hetero | 23 | 198 | 11.6 | 0.46 | 0 | 190 | - | UBE4B |
| 1 | 85278679 | SNV | G | A | 1 | 23658 | Hetero | 10 | 55 | 18.2 | 0.33 | 0 | 59 | - | LPAR3 |
| 1 | 100316556 | SNV | C | T | 1 | 23658 | Hetero | 17 | 159 | 10.7 | 0.43 | 0 | 166 | - | AGL |
| 1 | 145698990 | SNV | G | A | 1 | 23658 | Hetero | 23 | 120 | 19.2 | 0.32 | 0 | 87 | No | CD160 |
| 1 | 155348097 | SNV | C | T | 1 | 23658 | Hetero | 7 | 56 | 12.5 | 0.33 | 0 | 63 | Yes | ASH1L |
| 1 | 169698637 | SNV | G | A | 1 | 23658 | Hetero | 39 | 156 | 25.0 | 0.42 | 0 | 146 | Yes | C1orf112 |
| 1 | 171672785 | SNV | G | A | 1 | 23658 | Hetero | 13 | 78 | 16.7 | 0.25 | 0 | 89 | - | VAMP4 |
| 1 | 175348794 | SNV | G | A | 1 | 23658 | Hetero | 8 | 74 | 10.8 | 0.44 | 0 | 58 | No | TNR |
| 1 | 180904711 | SNV | G | A | 1 | 23658 | Hetero | 8 | 77 | 10.4 | 0.33 | 0 | 75 | Yes | KIAA1614 |
| 1 | 181027295 | SNV | A | T | 1 | 23658 | Hetero | 19 | 57 | 33.3 | 0.43 | 0 | 59 | - | MR1 |
| 1 | 205385427 | SNV | G | A | 1 | 23658 | Hetero | 15 | 137 | 10.9 | 0.39 | 0 | 125 | - | LEMD1 |
| 1 | 215256547 | Deletion | G | - | 1 | 23658 | Hetero | 4 | 40 | 10.0 | 0.20 | 0 | 42 | - | KCNK2 |
| 1 | 222744019 | SNV | T | C | 1 | 23658 | Hetero | 19 | 85 | 22.4 | 0.27 | 0 | 76 | - | TAF1A |
| 1 | 228509207 | SNV | C | T | 1 | 23658 | Hetero | 16 | 100 | 16.0 | 0.44 | 0 | 105 | Yes | OBSCN |
| 1 | 243663103 | SNV | G | C | 1 | 23658 | Hetero | 49 | 275 | 17.8 | 0.42 | 0 | 297 | - | SDCCAG8 |
| 1 | 94984052 | Insertion | - | A | 1 | 23658 | Hetero | 6 | 51 | 11.8 | 0.50 | 0 | 46 | - | ABCD3 |
| 2 | 27595486 | SNV | A | G | 1 | 23658 | Hetero | 22 | 88 | 25.0 | 0.50 | 0 | 99 | - | SNX17 |
| 2 | 32412166 | SNV | G | C | 1 | 23658 | Hetero | 39 | 115 | 33.9 | 0.18 | 0 | 115 | - | SLC30A6 |
| 2 | 64778636 | SNV | T | C | 1 | 23658 | Hetero | 100 | 341 | 29.3 | 0.49 | 0 | 260 | Yes | AFTPH |
| 2 | 99977924 | SNV | G | C | 1 | 23658 | Hetero | 13 | 91 | 14.3 | 0.36 | 0 | 63 | Yes | EIF5B |
| 2 | 103334263 | SNV | C | A | 1 | 23658 | Hetero | 10 | 38 | 26.3 | 0.42 | 0 | 41 | - | MFSD9 |
| 2 | 111922916 | SNV | C | T | 1 | 23658 | Hetero | 24 | 157 | 15.3 | 0.37 | 0 | 164 | - | BCL2L11 |
| 2 | 131523873 | SNV | C | A | 1 | 23658 | Hetero | 12 | 63 | 19.0 | 0.46 | 0 | 69 | - | FAM123C |
| 2 | 141747181 | SNV | C | T | 1 | 23658 | Hetero | 21 | 204 | 10.3 | 0.42 | 0 | 168 | Yes | LRP1B |
| 2 | 152531046 | SNV | A | G | 1 | 23658 | Hetero | 22 | 193 | 11.4 | 0.38 | 0 | 125 | Yes | NEB |
| 2 | 158287388 | SNV | A | C | 1 | 23658 | Hetero | 56 | 231 | 24.2 | 0.41 | 0 | 205 | Yes | CYTIP |
| 2 | 189387456 | SNV | C | G | 1 | 23658 | Hetero | 32 | 146 | 21.9 | 0.12 | 0 | 108 | - | GULP1 |
| 2 | 202525124 | SNV | G | A | 1 | 23658 | Hetero | 23 | 157 | 14.6 | 0.43 | 0 | 139 | - | MPP4 |
| 2 | 219135396 | SNV | A | G | 1 | 23658 | Hetero | 26 | 129 | 20.2 | 0.42 | 0 | 110 | - | PNKD |
| 2 | 241406998 | SNV | G | A | 1 | 23658 | Hetero | 6 | 43 | 14.0 | 0.43 | 0 | 51 | - | GPC1 |
| 2 | 128605207 | Insertion | - | A | 1 | 23658 | Hetero | 7 | 64 | 10.9 | 0.50 | 0 | 59 | - | POLR2D |
| 2 | 157370018 | Insertion | - | T | 1 | 23658 | Hetero | 6 | 37 | 16.2 | 0.33 | 0 | 36 | - | GPD2 |
| 3 | 58551781 | SNV | G | T | 1 | 23658 | Hetero | 22 | 72 | 30.6 | 0.48 | 0 | 97 | - | FAM107A |
| 3 | 108634977 | SNV | C | T | 1 | 23658 | Hetero | 45 | 226 | 19.9 | 0.42 | 0 | 157 | Yes | GUCA1C |
| 3 | 109027942 | SNV | G | A | 1 | 23658 | Hetero | 34 | 83 | 41.0 | 0.43 | 0 | 60 | - | DPPA2 |
| 3 | 130311492 | SNV | A | T | 1 | 23658 | Hetero | 22 | 97 | 22.7 | 0.30 | 0 | 81 | - | COL6A6 |
| 3 | 131187043 | SNV | G | C | 1 | 23658 | Hetero | 9 | 66 | 13.6 | 0.30 | 0 | 43 | - | MRPL3 |
| 3 | 179504257 | SNV | A | T | 1 | 23658 | Hetero | 14 | 87 | 16.1 | 0.40 | 0 | 54 | - | USP13 |
| 4 | 39046756 | SNV | C | T | 1 | 23658 | Hetero | 4 | 32 | 12.5 | 0.50 | 0 | 31 | - | KLHL5 |
| 4 | 57798733 | SNV | A | T | 1 | 23658 | Hetero | 13 | 106 | 12.3 | 0.23 | 0 | 100 | - | REST |
| 4 | 96077806 | Deletion | A | - | 1 | 23658 | Hetero | 5 | 45 | 11.1 | 0.20 | 0 | 47 | - | BMPR1B |
| 4 | 113196843 | SNV | A | T | 1 | 23658 | Hetero | 10 | 69 | 14.5 | 0.47 | 0 | 65 | - | TIFA |
| 4 | 144338550 | SNV | T | A | 1 | 23658 | Hetero | 17 | 67 | 25.4 | 0.16 | 0 | 73 | - | GAB1 |
| 4 | 190394554 | SNV | G | T | 1 | 23658 | Hetero | 42 | 202 | 20.8 | 0.43 | 0 | 191 | - | HSP90AA4P |
| 4 | 40425475 | Insertion | - | A | 1 | 23658 | Hetero | 5 | 44 | 11.4 | 0.40 | 0 | 64 | - | RBM47 |
| 5 | 33946404 | SNV | T | G | 1 | 23658 | Hetero | 12 | 61 | 19.7 | 0.08 | 0 | 61 | - | SLC45A2 |
| 5 | 63257562 | SNV | G | A | 1 | 23658 | Hetero | 20 | 94 | 21.3 | 0.39 | 0 | 139 | - | HTR1A |
| 5 | 111066236 | Deletion | T | - | 1 | 23658 | Hetero | 17 | 110 | 15.5 | 0.06 | 0 | 152 | - | NREP |
| 5 | 118576176 | SNV | C | T | 1 | 23658 | Hetero | 4 | 30 | 13.3 | 0.25 | 0 | 35 | - | DMXL1 |
| 5 | 134870706 | SNV | G | C | 1 | 23658 | Hetero | 42 | 171 | 24.6 | 0.46 | 0 | 207 | Yes | NEUROG1 |
| 5 | 138658552 | SNV | G | T | 1 | 23658 | Hetero | 13 | 87 | 14.9 | 0.27 | 0 | 116 | Yes | MATR3 |
| 5 | 126113298 | Insertion | - | G | 1 | 23658 | Hetero | 10 | 48 | 20.8 | 0.42 | 0 | 73 | Insertion | LMNB1 |
| 5 | 154196577 | Insertion | - | T | 1 | 23658 | Hetero | 4 | 38 | 10.5 | 0.25 | 0 | 58 | - | LARP1 |
| 6 | 2783666 | SNV | G | A | 1 | 23658 | Hetero | 13 | 111 | 11.7 | 0.35 | 0 | 92 | Yes | WRNIP1 |
| 6 | 30139889 | SNV | G | T | 1 | 23658 | Hetero | 21 | 108 | 19.4 | 0.46 | 0 | 96 | Yes | TRIM15 |
| 6 | 30568235 | Deletion | A | - | 1 | 23658 | Hetero | 7 | 69 | 10.1 | 0.25 | 0 | 65 | - | PPP1R10 |
| 6 | 33421401 | SNV | C | A | 1 | 23658 | Hetero | 22 | 82 | 26.8 | 0.40 | 0 | 67 | - | SYNGAP1 |
| 6 | 39053982 | SNV | T | C | 1 | 23658 | Hetero | 17 | 95 | 17.9 | 0.29 | 0 | 74 | - | GLP1R |
| 6 | 43184032 | SNV | C | G | 1 | 23658 | Hetero | 53 | 206 | 25.7 | 0.46 | 0 | 172 | Yes | CUL9 |
| 6 | 62390900 | SNV | C | T | 1 | 23658 | Hetero | 41 | 166 | 24.7 | 0.22 | 0 | 173 | Yes | KHDRBS2 |
| 6 | 158049352 | Deletion | T | - | 1 | 23658 | Hetero | 7 | 65 | 10.8 | 0.22 | 0 | 54 | - | ZDHHC14 |
| 6 | 160872014 | SNV | C | G | 1 | 23658 | Hetero | 36 | 260 | 13.8 | 0.17 | 0 | 283 | - | SLC22A3 |
| 6 | 167752230 | SNV | A | T | 1 | 23658 | Hetero | 12 | 87 | 13.8 | 0.25 | 0 | 76 | Yes | TTLL2 |
| 6 | 114182251 | Insertion | - | T | 1 | 23658 | Hetero | 7 | 53 | 13.2 | 0.25 | 0 | 47 | - | MARCKS |
| 7 | 89869595 | Deletion | AA | - | 2 | 23658 | Hetero | 6 | 54 | 11.1 | 0.43 | 0 | 34 | - | STEAP2 |
| 8 | 30405003 | SNV | T | C | 1 | 23658 | Hetero | 34 | 257 | 13.2 | 0.39 | 0 | 292 | - | RBPMS |
| 8 | 38138964 | Deletion | A | - | 1 | 23658 | Hetero | 5 | 43 | 11.6 | 0.17 | 0 | 31 | - | WHSC1L1 |
| 8 | 43057460 | SNV | C | A | 1 | 23658 | Hetero | 25 | 110 | 22.7 | 0.45 | 0 | 125 | - | HGSNAT |
| 8 | 69243666 | SNV | G | T | 1 | 23658 | Hetero | 64 | 369 | 17.3 | 0.38 | 0 | 293 | No | C8orf34 |
| 8 | 73848197 | SNV | A | G | 1 | 23658 | Hetero | 31 | 140 | 22.1 | 0.20 | 0 | 94 | Yes | KCNB2 |
| 8 | 75614667 | SNV | A | T | 1 | 23658 | Hetero | 51 | 192 | 26.6 | 0.39 | 0 | 124 | Yes | RP11-758M4.1 |
| 8 | 144873997 | Deletion | A | - | 1 | 23658 | Hetero | 8 | 80 | 10.0 | 0.44 | 0 | 102 | - | SCRIB |
| 9 | 72459549 | SNV | G | A | 1 | 23658 | Hetero | 11 | 96 | 11.5 | 0.40 | 0 | 118 | - | C9orf135 |
| 9 | 140243814 | SNV | C | T | 1 | 23658 | Hetero | 12 | 57 | 21.1 | 0.43 | 0 | 79 | - | EXD3 |
| 10 | 52569774 | SNV | C | G | 1 | 23658 | Hetero | 24 | 147 | 16.3 | 0.44 | 0 | 109 | Yes | ASAH2B |
| 10 | 81075617 | Deletion | A | - | 1 | 23658 | Hetero | 9 | 80 | 11.3 | 0.22 | 0 | 81 | - | ZMIZ1 |
| 10 | 85976676 | SNV | G | A | 1 | 23658 | Hetero | 13 | 92 | 14.1 | 0.43 | 0 | 100 | - | CDHR1 |
| 10 | 90582605 | SNV | C | A | 1 | 23658 | Hetero | 44 | 164 | 26.8 | 0.38 | 0 | 145 | - | ANKRD22 |
| 10 | 116054775 | Insertion | - | AA | 2 | 23658 | Hetero | 7 | 38 | 18.4 | 0.43 | 0 | 66 | - | AFAP1L2 |
| 10 | 125780762 | Insertion | - | GC | 2 | 23658 | Hetero | 7 | 30 | 23.3 | 0.43 | 0 | 39 | Insertion | CHST15 |
| 11 | 17523464 | SNV | G | C | 1 | 23658 | Hetero | 17 | 128 | 13.3 | 0.32 | 0 | 140 | - | USH1C |
| 11 | 33037559 | SNV | G | T | 1 | 23658 | Hetero | 10 | 77 | 13.0 | 0.50 | 0 | 93 | Yes | DEPDC7 |
| 11 | 55606341 | SNV | C | A | 1 | 23658 | Hetero | 50 | 231 | 21.6 | 0.27 | 0 | 209 | No | OR5D16 |
| 11 | 64109574 | SNV | A | C | 1 | 23658 | Hetero | 11 | 55 | 20.0 | 0.42 | 0 | 60 | Yes | CCDC88B |
| 11 | 86520328 | SNV | C | T | 1 | 23658 | Hetero | 11 | 92 | 12.0 | 0.46 | 0 | 92 | - | PRSS23 |
| 11 | 93822094 | SNV | T | A | 1 | 23658 | Hetero | 18 | 128 | 14.1 | 0.43 | 0 | 110 | Yes | HEPHL1 |
| 11 | 107975795 | Insertion | - | T | 1 | 23658 | Hetero | 5 | 47 | 10.6 | 0.40 | 0 | 44 | - | CUL5 |
| 11 | 6619119 | Insertion | - | A | 1 | 23658 | Hetero | 6 | 59 | 10.2 | 0.38 | 0 | 62 | - | RRP8 |
| 12 | 9747514 | SNV | T | G | 1 | 23658 | Hetero | 31 | 80 | 38.8 | 0.42 | 0 | 51 | - | KLRB1 |
| 12 | 51138513 | SNV | A | G | 1 | 23658 | Hetero | 64 | 348 | 18.4 | 0.42 | 0 | 258 | Yes | DIP2B |
| 12 | 79980384 | SNV | T | C | 1 | 23658 | Hetero | 13 | 91 | 14.3 | 0.20 | 0 | 93 | - | PAWR |
| 12 | 81205511 | SNV | T | C | 1 | 23658 | Hetero | 6 | 47 | 12.8 | 0.29 | 0 | 43 | - | LIN7A |
| 12 | 99019180 | SNV | C | T | 1 | 23658 | Hetero | 6 | 59 | 10.2 | 0.33 | 0 | 74 | - | IKBIP |
| 12 | 109994544 | SNV | C | A | 1 | 23658 | Hetero | 16 | 82 | 19.5 | 0.25 | 0 | 77 | - | MMAB |
| 12 | 21327652 | Insertion | - | AA | 2 | 23658 | Hetero | 6 | 53 | 11.3 | 0.17 | 0 | 33 | - | SLCO1B1 |
| 12 | 52388986 | Insertion | - | A | 1 | 23658 | Hetero | 22 | 100 | 22.0 | 0.38 | 0 | 104 | - | ACVR1B |
| 13 | 37269030 | Deletion | T | - | 1 | 23658 | Hetero | 8 | 75 | 10.7 | 0.33 | 0 | 68 | - | SERTM1 |
| 13 | 39263035 | SNV | C | A | 1 | 23658 | Hetero | 9 | 85 | 10.6 | 0.40 | 0 | 89 | No | FREM2 |
| 13 | 42293537 | SNV | A | C | 1 | 23658 | Hetero | 9 | 52 | 17.3 | 0.30 | 0 | 51 | - | VWA8 |
| 13 | 50594359 | SNV | T | C | 1 | 23658 | Hetero | 23 | 183 | 12.6 | 0.12 | 0 | 174 | Yes | TRIM13 |
| 13 | 70314647 | SNV | G | T | 1 | 23658 | Hetero | 13 | 102 | 12.7 | 0.27 | 0 | 55 | Yes | KLHL1 |
| 13 | 30340720 | Insertion | - | A | 1 | 23658 | Hetero | 12 | 43 | 27.9 | 0.42 | 0 | 60 | - | UBL3 |
| 14 | 24778434 | Deletion | T | - | 1 | 23658 | Hetero | 39 | 121 | 32.2 | 0.41 | 0 | 134 | - | CIDEB |
| 14 | 52182165 | SNV | T | A | 1 | 23658 | Hetero | 104 | 225 | 46.2 | 0.35 | 0 | 168 | Yes | FRMD6 |
| 14 | 69341165 | SNV | G | A | 1 | 23658 | Hetero | 32 | 58 | 55.2 | 0.49 | 0 | 47 | - | ACTN1 |
| 14 | 91699293 | SNV | G | C | 1 | 23658 | Hetero | 104 | 237 | 43.9 | 0.39 | 0 | 180 | - | GPR68 |
| 15 | 24921264 | SNV | G | A | 1 | 23658 | Hetero | 21 | 143 | 14.7 | 0.48 | 0 | 147 | Yes | NPAP1 |
| 15 | 42104001 | SNV | A | C | 1 | 23658 | Hetero | 11 | 55 | 20.0 | 0.17 | 0 | 72 | - | MAPKBP1 |
| 15 | 51018573 | SNV | T | C | 1 | 23658 | Hetero | 17 | 115 | 14.8 | 0.39 | 0 | 94 | Possible splice site disruption | SPPL2A |
| 15 | 54557586 | SNV | A | T | 1 | 23658 | Hetero | 10 | 67 | 14.9 | 0.33 | 0 | 66 | Yes | UNC13C |
| 15 | 67500862 | SNV | C | A | 1 | 23658 | Hetero | 34 | 146 | 23.3 | 0.40 | 0 | 155 | - | AAGAB |
| 16 | 9858383 | SNV | C | T | 1 | 23658 | Hetero | 28 | 167 | 16.8 | 0.43 | 0 | 134 | No | GRIN2A |
| 16 | 20975808 | SNV | T | C | 1 | 23658 | Hetero | 24 | 191 | 12.6 | 0.41 | 0 | 200 | Yes | DNAH3 |
| 16 | 21689926 | SNV | G | T | 1 | 23658 | Hetero | 29 | 232 | 12.5 | 0.49 | 0 | 169 | Possible splice site disruption | OTOA |
| 16 | 30591132 | SNV | T | A | 1 | 23658 | Hetero | 18 | 146 | 12.3 | 0.43 | 0 | 177 | - | AC002310.7 |
| 16 | 48580151 | SNV | G | A | 1 | 23658 | Hetero | 40 | 155 | 25.8 | 0.46 | 0 | 123 | Yes | N4BP1 |
| 17 | 4859412 | SNV | C | T | 1 | 23658 | Hetero | 54 | 205 | 26.3 | 0.47 | 0 | 193 | No | ENO3 |
| 17 | 5338179 | SNV | T | C | 1 | 23658 | Hetero | 16 | 103 | 15.5 | 0.22 | 0 | 104 | - | C1QBP |
| 17 | 7577539 | SNV | G | A | 1 | 23658 | Hetero | 14 | 80 | 17.5 | 0.47 | 0 | 88 | Yes | TP53 |
| 17 | 7579485 | SNV | C | A | 1 | 23658 | Hetero | 10 | 40 | 25.0 | 0.31 | 0 | 34 | Yes | TP53 |
| 17 | 33334078 | SNV | A | G | 1 | 23658 | Hetero | 25 | 111 | 22.5 | 0.39 | 0 | 89 | - | RFFL |
| 17 | 42326118 | SNV | C | A | 1 | 23658 | Hetero | 18 | 119 | 15.1 | 0.29 | 0 | 122 | - | SLC4A1 |
| 17 | 46940693 | SNV | G | A | 1 | 23658 | Hetero | 15 | 89 | 16.9 | 0.50 | 0 | 70 | - | CALCOCO2 |
| 17 | 62009562 | SNV | C | A | 1 | 23658 | Hetero | 36 | 177 | 20.3 | 0.38 | 0 | 167 | No | CD79B |
| 17 | 66042003 | SNV | C | T | 1 | 23658 | Hetero | 4 | 40 | 10.0 | 0.33 | 0 | 42 | Yes | KPNA2 |
| 17 | 72956134 | SNV | A | T | 1 | 23658 | Hetero | 45 | 219 | 20.5 | 0.47 | 0 | 236 | Yes | C17orf28 |
| 17 | 27941253 | Insertion | - | T | 1 | 23658 | Hetero | 13 | 112 | 11.6 | 0.21 | 0 | 93 | - | ANKRD13B |
| 18 | 24442316 | SNV | C | A | 1 | 23658 | Hetero | 45 | 255 | 17.6 | 0.49 | 0 | 176 | Yes | AQP4 |
| 18 | 45359987 | SNV | T | C | 1 | 23658 | Hetero | 20 | 61 | 32.8 | 0.43 | 0 | 68 | - | SMAD2 |
| 18 | 59814210 | SNV | C | A | 1 | 23658 | Hetero | 20 | 77 | 26.0 | 0.32 | 0 | 85 | Yes | PIGN |
| 20 | 9389360 | SNV | G | T | 1 | 23658 | Hetero | 17 | 113 | 15.0 | 0.41 | 0 | 97 | Yes | PLCB4 |
| 20 | 13769224 | Deletion | T | - | 1 | 23658 | Hetero | 5 | 49 | 10.2 | 0.50 | 0 | 40 | - | NDUFAF5 |
| 20 | 48569062 | SNV | A | T | 1 | 23658 | Hetero | 15 | 127 | 11.8 | 0.39 | 0 | 134 | - | RNF114 |
| 21 | 33829984 | SNV | C | T | 1 | 23658 | Hetero | 21 | 97 | 21.6 | 0.44 | 0 | 87 | Yes | FAM176C |
| 21 | 38470407 | SNV | A | T | 1 | 23658 | Hetero | 51 | 247 | 20.6 | 0.27 | 0 | 204 | - | TTC3 |
| 22 | 24812423 | SNV | C | T | 1 | 23658 | Hetero | 59 | 169 | 34.9 | 0.38 | 0 | 116 | - | SPECC1L |
| 22 | 35719714 | SNV | C | G | 1 | 23658 | Hetero | 91 | 320 | 28.4 | 0.47 | 0 | 267 | - | TOM1 |
| 22 | 38522389 | SNV | G | A | 1 | 23658 | Hetero | 16 | 82 | 19.5 | 0.50 | 0 | 69 | Yes | PLA2G6 |
| X | 2184819 | SNV | A | T | 1 | 23658 | Hetero | 42 | 179 | 23.5 | 0.31 | 0 | 187 | Yes | DHRSX |
| X | 44920633 | Deletion | A | - | 1 | 23658 | Hetero | 14 | 69 | 20.3 | 0.50 | 0 | 51 | Deletion | KDM6A |
| X | 107310302 | SNV | A | G | 1 | 23658 | Hetero | 17 | 86 | 19.8 | 0.29 | 0 | 102 | Yes | VSIG1 |
| X | 142716949 | SNV | G | T | 1 | 23658 | Hetero | 23 | 76 | 30.3 | 0.46 | 0 | 89 | Yes | SLITRK4 |
| 1 | 2238330 | SNV | C | T | 1 | 23727 | Hetero | 17 | 82 | 20.7 | 0.33 | 0 | 62 | - | SKI |
| 1 | 6530971 | SNV | C | G | 1 | 23727 | Hetero | 17 | 104 | 16.3 | 0.41 | 0 | 52 | - | PLEKHG5 |
| 1 | 16272708 | SNV | A | T | 1 | 23727 | Hetero | 39 | 160 | 24.4 | 0.43 | 0 | 101 | Yes | ZBTB17 |
| 1 | 27239910 | SNV | G | C | 1 | 23727 | Hetero | 44 | 164 | 26.8 | 0.31 | 0 | 99 | No | NUDC |
| 1 | 28268833 | SNV | C | G | 1 | 23727 | Hetero | 48 | 123 | 39.0 | 0.42 | 0 | 69 | Yes | SMPDL3B |
| 1 | 38042049 | SNV | G | C | 1 | 23727 | Hetero | 36 | 164 | 22.0 | 0.43 | 0 | 119 | Yes | GNL2 |
| 1 | 39876032 | SNV | A | G | 1 | 23727 | Hetero | 38 | 112 | 33.9 | 0.27 | 0 | 85 | - | MACF1 |
| 1 | 39878900 | SNV | A | G | 1 | 23727 | Hetero | 26 | 91 | 28.6 | 0.20 | 0 | 75 | Yes | MACF1 |
| 1 | 77095499 | SNV | C | T | 1 | 23727 | Hetero | 8 | 77 | 10.4 | 0.33 | 0 | 50 | - | ST6GALNAC3 |
| 1 | 84815654 | SNV | C | T | 1 | 23727 | Hetero | 13 | 70 | 18.6 | 0.50 | 0 | 71 | - | SAMD13 |
| 1 | 95448665 | SNV | A | T | 1 | 23727 | Hetero | 97 | 366 | 26.5 | 0.49 | 0 | 290 | Yes | ALG14 |
| 1 | 99356040 | SNV | C | G | 1 | 23727 | Hetero | 26 | 109 | 23.9 | 0.43 | 0 | 59 | - | LPPR5 |
| 1 | 110233096 | SNV | C | T | 1 | 23727 | Hetero | 74 | 201 | 36.8 | 0.20 | 0 | 125 | No | GSTM2 |
| 1 | 117057259 | SNV | T | C | 1 | 23727 | Hetero | 12 | 70 | 17.1 | 0.46 | 0 | 35 | - | CD58 |
| 1 | 118168917 | SNV | C | T | 1 | 23727 | Hetero | 30 | 154 | 19.5 | 0.30 | 0 | 93 | - | FAM46C |
| 1 | 151732702 | SNV | T | C | 1 | 23727 | Hetero | 25 | 102 | 24.5 | 0.28 | 0 | 76 | - | MRPL9 |
| 1 | 151752475 | SNV | C | A | 1 | 23727 | Hetero | 35 | 129 | 27.1 | 0.33 | 0 | 91 | Yes | TDRKH |
| 1 | 152975827 | SNV | G | A | 1 | 23727 | Hetero | 19 | 58 | 32.8 | 0.19 | 0 | 69 | Yes | SPRR3 |
| 1 | 155631119 | SNV | G | C | 1 | 23727 | Hetero | 28 | 103 | 27.2 | 0.27 | 0 | 123 | Yes | MSTO1 |
| 1 | 158548798 | SNV | G | A | 1 | 23727 | Hetero | 55 | 207 | 26.6 | 0.34 | 0 | 103 | Yes | OR10X1 |
| 1 | 159172107 | SNV | G | A | 1 | 23727 | Hetero | 61 | 238 | 25.6 | 0.40 | 0 | 164 | - | CADM3 |
| 1 | 179380300 | SNV | G | C | 1 | 23727 | Hetero | 8 | 41 | 19.5 | 0.20 | 0 | 32 | Yes | AXDND1 |
| 1 | 196621129 | SNV | T | A | 1 | 23727 | Hetero | 86 | 333 | 25.8 | 0.35 | 0 | 221 | - | CFH |
| 1 | 202563821 | Deletion | T | - | 1 | 23727 | Hetero | 5 | 30 | 16.7 | 0.40 | 0 | 35 | - | SYT2 |
| 1 | 206629067 | SNV | C | A | 1 | 23727 | Hetero | 50 | 219 | 22.8 | 0.36 | 0 | 147 | - | SRGAP2 |
| 1 | 226034908 | SNV | C | A | 1 | 23727 | Hetero | 19 | 103 | 18.4 | 0.36 | 0 | 64 | Yes | TMEM63A |
| 1 | 236590676 | Deletion | T | - | 1 | 23727 | Hetero | 5 | 45 | 11.1 | 0.40 | 0 | 31 | - | EDARADD |
| 1 | 32697092 | Insertion | - | A | 1 | 23727 | Hetero | 7 | 42 | 16.7 | 0.38 | 0 | 36 | - | EIF3I |
| 2 | 74442713 | SNV | G | A | 1 | 23727 | Hetero | 37 | 129 | 28.7 | 0.38 | 0 | 64 | - | MTHFD2 |
| 2 | 86992215 | SNV | G | C | 1 | 23727 | Hetero | 48 | 173 | 27.7 | 0.47 | 0 | 106 | Yes | RMND5A |
| 2 | 95843360 | SNV | G | A | 1 | 23727 | Hetero | 45 | 155 | 29.0 | 0.18 | 0 | 125 | - | ZNF2 |
| 2 | 99697732 | SNV | G | C | 1 | 23727 | Hetero | 6 | 39 | 15.4 | 0.17 | 0 | 33 | - | TSGA10 |
| 2 | 107418377 | SNV | C | G | 1 | 23727 | Hetero | 18 | 91 | 19.8 | 0.35 | 0 | 69 | - | ST6GAL2 |
| 2 | 120849178 | SNV | C | G | 1 | 23727 | Hetero | 27 | 131 | 20.6 | 0.43 | 0 | 90 | Yes | EPB41L5 |
| 2 | 139045931 | SNV | C | A | 1 | 23727 | Hetero | 32 | 146 | 21.9 | 0.45 | 0 | 99 | - | ENSG00000234645 |
| 2 | 141232804 | SNV | A | C | 1 | 23727 | Hetero | 37 | 112 | 33.0 | 0.40 | 0 | 51 | No | LRP1B |
| 2 | 152659119 | Deletion | T | - | 1 | 23727 | Hetero | 13 | 75 | 17.3 | 0.20 | 0 | 34 | - | ARL5A |
| 2 | 160980401 | SNV | T | C | 1 | 23727 | Hetero | 43 | 145 | 29.7 | 0.33 | 0 | 100 | - | ITGB6 |
| 2 | 180815366 | SNV | G | A | 1 | 23727 | Hetero | 26 | 94 | 27.7 | 0.47 | 0 | 42 | No | CWC22 |
| 2 | 189460242 | SNV | T | C | 1 | 23727 | Hetero | 7 | 38 | 18.4 | 0.44 | 0 | 37 | - | GULP1 |
| 2 | 204083540 | SNV | G | A | 1 | 23727 | Hetero | 30 | 102 | 29.4 | 0.46 | 0 | 62 | - | NBEAL1 |
| 2 | 210858191 | SNV | G | C | 1 | 23727 | Hetero | 19 | 89 | 21.3 | 0.33 | 0 | 47 | - | UNC80 |
| 2 | 219826856 | SNV | T | C | 1 | 23727 | Hetero | 34 | 116 | 29.3 | 0.25 | 0 | 45 | - | CDK5R2 |
| 2 | 234386217 | SNV | G | T | 1 | 23727 | Hetero | 7 | 55 | 12.7 | 0.33 | 0 | 31 | - | USP40 |
| 2 | 238280544 | SNV | G | A | 1 | 23727 | Hetero | 61 | 219 | 27.9 | 0.35 | 0 | 125 | No | COL6A3 |
| 2 | 239358248 | SNV | A | T | 1 | 23727 | Hetero | 21 | 97 | 21.6 | 0.26 | 0 | 49 | - | ASB1 |
| 2 | 242179381 | SNV | T | C | 1 | 23727 | Hetero | 41 | 190 | 21.6 | 0.43 | 0 | 134 | Yes | HDLBP |
| 2 | 181927715 | Insertion | - | T | 1 | 23727 | Hetero | 52 | 196 | 26.5 | 0.43 | 0 | 117 | - | UBE2E3 |
| 3 | 12983139 | SNV | C | G | 1 | 23727 | Hetero | 11 | 64 | 17.2 | 0.46 | 0 | 50 | Yes | IQSEC1 |
| 3 | 37367445 | Deletion | A | - | 1 | 23727 | Hetero | 25 | 88 | 28.4 | 0.31 | 0 | 68 | Deletion | GOLGA4 |
| 3 | 57344960 | SNV | C | G | 1 | 23727 | Hetero | 14 | 92 | 15.2 | 0.27 | 0 | 106 | Yes | DNAH12 |
| 3 | 93733423 | SNV | C | T | 1 | 23727 | Hetero | 16 | 101 | 15.8 | 0.39 | 0 | 60 | Yes | ARL13B |
| 3 | 107525639 | SNV | G | C | 1 | 23727 | Hetero | 32 | 146 | 21.9 | 0.46 | 0 | 63 | - | BBX |
| 3 | 107762289 | Deletion | A | - | 1 | 23727 | Hetero | 15 | 88 | 17.0 | 0.35 | 0 | 38 | - | CD47 |
| 3 | 112968650 | SNV | A | G | 1 | 23727 | Hetero | 86 | 460 | 18.7 | 0.40 | 0 | 225 | Yes | BOC |
| 3 | 119150289 | SNV | G | A | 1 | 23727 | Hetero | 14 | 96 | 14.6 | 0.25 | 0 | 49 | - | TMEM39A |
| 3 | 119361309 | SNV | T | C | 1 | 23727 | Hetero | 57 | 334 | 17.1 | 0.34 | 0 | 154 | - | POPDC2 |
| 3 | 130313182 | SNV | G | T | 1 | 23727 | Hetero | 12 | 77 | 15.6 | 0.33 | 0 | 45 | Yes | COL6A6 |
| 3 | 132441016 | SNV | G | A | 1 | 23727 | Hetero | 24 | 146 | 16.4 | 0.28 | 0 | 60 | Yes | NPHP3 |
| 3 | 141670937 | SNV | C | G | 1 | 23727 | Hetero | 22 | 108 | 20.4 | 0.41 | 0 | 74 | - | TFDP2 |
| 3 | 149087295 | SNV | T | A | 1 | 23727 | Hetero | 15 | 91 | 16.5 | 0.32 | 0 | 50 | - | TM4SF1 |
| 3 | 150264600 | SNV | C | T | 1 | 23727 | Hetero | 19 | 90 | 21.1 | 0.35 | 0 | 57 | Yes | SERP1 |
| 3 | 152880184 | SNV | C | T | 1 | 23727 | Hetero | 39 | 153 | 25.5 | 0.41 | 0 | 43 | - | RAP2B |
| 3 | 152883396 | Deletion | T | - | 1 | 23727 | Hetero | 15 | 104 | 14.4 | 0.19 | 0 | 52 | - | RAP2B |
| 3 | 178956006 | SNV | G | T | 1 | 23727 | Hetero | 49 | 271 | 18.1 | 0.46 | 0 | 141 | - | PIK3CA |
| 3 | 181431412 | SNV | G | C | 1 | 23727 | Hetero | 12 | 69 | 17.4 | 0.50 | 0 | 30 | - | SOX2 |
| 3 | 184971836 | SNV | C | G | 1 | 23727 | Hetero | 39 | 190 | 20.5 | 0.40 | 0 | 115 | - | EHHADH |
| 3 | 185324237 | SNV | C | G | 1 | 23727 | Hetero | 38 | 201 | 18.9 | 0.41 | 0 | 97 | Yes | SENP2 |
| 3 | 185866139 | SNV | G | C | 1 | 23727 | Hetero | 29 | 136 | 21.3 | 0.38 | 0 | 61 | - | DGKG |
| 3 | 188427942 | SNV | G | A | 1 | 23727 | Hetero | 18 | 128 | 14.1 | 0.48 | 0 | 72 | - | LPP |
| 3 | 190982239 | SNV | C | G | 1 | 23727 | Hetero | 9 | 57 | 15.8 | 0.20 | 0 | 41 | - | OSTN |
| 3 | 195936343 | SNV | C | T | 1 | 23727 | Hetero | 37 | 145 | 25.5 | 0.30 | 0 | 52 | No | ZDHHC19 |
| 3 | 62283125 | Insertion | - | T | 1 | 23727 | Hetero | 6 | 38 | 15.8 | 0.29 | 0 | 31 | - | PTPRG |
| 3 | 9026778 | Deletion | AT | - | 2 | 23727 | Hetero | 5 | 30 | 16.7 | 0.40 | 0 | 31 | - | SRGAP3 |
| 3 | 9951039 | Deletion | AC | - | 2 | 23727 | Hetero | 8 | 38 | 21.1 | 0.25 | 0 | 40 | - | IL17RE |
| 4 | 8034391 | SNV | G | A | 1 | 23727 | Hetero | 38 | 129 | 29.5 | 0.45 | 0 | 84 | Yes | ABLIM2 |
| 4 | 17503417 | SNV | G | C | 1 | 23727 | Hetero | 9 | 84 | 10.7 | 0.45 | 0 | 51 | Yes | QDPR |
| 4 | 41622525 | SNV | G | A | 1 | 23727 | Hetero | 20 | 80 | 25.0 | 0.41 | 0 | 52 | Yes | LIMCH1 |
| 4 | 42627666 | SNV | C | T | 1 | 23727 | Hetero | 41 | 166 | 24.7 | 0.26 | 0 | 138 | Yes | ATP8A1 |
| 4 | 66201726 | SNV | C | G | 1 | 23727 | Hetero | 24 | 97 | 24.7 | 0.37 | 0 | 89 | Yes | EPHA5 |
| 4 | 76811235 | SNV | C | T | 1 | 23727 | Hetero | 16 | 76 | 21.1 | 0.42 | 0 | 44 | Yes | PPEF2 |
| 4 | 83847476 | SNV | C | A | 1 | 23727 | Hetero | 16 | 68 | 23.5 | 0.19 | 0 | 38 | - | LIN54 |
| 4 | 113191135 | SNV | G | T | 1 | 23727 | Hetero | 28 | 86 | 32.6 | 0.38 | 0 | 53 | - | AP1AR |
| 4 | 122255800 | SNV | C | A | 1 | 23727 | Hetero | 12 | 111 | 10.8 | 0.08 | 0 | 70 | - | QRFPR |
| 4 | 154623944 | SNV | G | A | 1 | 23727 | Hetero | 6 | 57 | 10.5 | 0.50 | 0 | 34 | - | TLR2 |
| 4 | 156271352 | SNV | G | C | 1 | 23727 | Hetero | 35 | 117 | 29.9 | 0.29 | 0 | 83 | - | MAP9 |
| 4 | 167655843 | SNV | A | T | 1 | 23727 | Hetero | 15 | 98 | 15.3 | 0.37 | 0 | 41 | - | SPOCK3 |
| 4 | 79800782 | Deletion | TG | - | 2 | 23727 | Hetero | 9 | 55 | 16.4 | 0.44 | 0 | 40 | - | BMP2K |
| 5 | 1443105 | SNV | G | C | 1 | 23727 | Hetero | 48 | 182 | 26.4 | 0.49 | 0 | 141 | Yes | SLC6A3 |
| 5 | 43557211 | SNV | C | T | 1 | 23727 | Hetero | 19 | 72 | 26.4 | 0.32 | 0 | 49 | - | PAIP1 |
| 5 | 49706790 | SNV | G | A | 1 | 23727 | Hetero | 23 | 97 | 23.7 | 0.19 | 0 | 61 | No | EMB |
| 5 | 111091499 | SNV | C | G | 1 | 23727 | Hetero | 51 | 220 | 23.2 | 0.50 | 0 | 167 | - | NREP |
| 5 | 138665226 | SNV | C | G | 1 | 23727 | Hetero | 13 | 79 | 16.5 | 0.25 | 0 | 71 | - | MATR3 |
| 5 | 140730419 | SNV | G | C | 1 | 23727 | Hetero | 39 | 154 | 25.3 | 0.48 | 0 | 103 | Yes | PCDHGA1 |
| 5 | 145968551 | SNV | C | G | 1 | 23727 | Hetero | 11 | 90 | 12.2 | 0.18 | 0 | 48 | - | PPP2R2B |
| 5 | 149362585 | Deletion | A | - | 1 | 23727 | Hetero | 17 | 84 | 20.2 | 0.33 | 0 | 40 | - | SLC26A2 |
| 5 | 156186326 | SNV | C | T | 1 | 23727 | Hetero | 28 | 93 | 30.1 | 0.17 | 0 | 74 | No | SGCD |
| 5 | 156679822 | SNV | G | C | 1 | 23727 | Hetero | 20 | 91 | 22.0 | 0.30 | 0 | 93 | - | ITK |
| 5 | 156905103 | Deletion | A | - | 1 | 23727 | Hetero | 29 | 86 | 33.7 | 0.45 | 0 | 47 | - | ADAM19 |
| 5 | 175308389 | SNV | G | C | 1 | 23727 | Hetero | 24 | 115 | 20.9 | 0.50 | 0 | 79 | - | CPLX2 |
| 5 | 176965083 | SNV | T | C | 1 | 23727 | Hetero | 7 | 34 | 20.6 | 0.50 | 0 | 30 | Yes | FAM193B |
| 6 | 7288158 | Deletion | A | - | 1 | 23727 | Hetero | 6 | 45 | 13.3 | 0.17 | 0 | 33 | - | SSR1 |
| 6 | 25983436 | SNV | C | G | 1 | 23727 | Hetero | 49 | 188 | 26.1 | 0.39 | 0 | 146 | Yes | TRIM38 |
| 6 | 33744434 | SNV | G | A | 1 | 23727 | Hetero | 16 | 67 | 23.9 | 0.28 | 0 | 36 | - | LEMD2 |
| 6 | 74348177 | SNV | G | T | 1 | 23727 | Hetero | 54 | 191 | 28.3 | 0.38 | 0 | 103 | Yes | SLC17A5 |
| 6 | 82950542 | SNV | C | T | 1 | 23727 | Hetero | 9 | 41 | 22.0 | 0.18 | 0 | 32 | - | IBTK |
| 6 | 110714306 | Deletion | A | - | 1 | 23727 | Hetero | 55 | 215 | 25.6 | 0.35 | 0 | 122 | Deletion | DDO |
| 6 | 117203588 | SNV | C | G | 1 | 23727 | Hetero | 50 | 220 | 22.7 | 0.49 | 0 | 172 | Yes | RFX6 |
| 6 | 127764998 | SNV | G | C | 1 | 23727 | Hetero | 14 | 68 | 20.6 | 0.47 | 0 | 65 | - | RP11-73O6.4 |
| 6 | 165742323 | SNV | C | A | 1 | 23727 | Hetero | 19 | 54 | 35.2 | 0.40 | 0 | 33 | - | PDE10A |
| 6 | 168947784 | SNV | C | T | 1 | 23727 | Hetero | 9 | 89 | 10.1 | 0.33 | 0 | 68 | Yes | SMOC2 |
| 6 | 32557582 | Insertion | - | G | 1 | 23727 | Hetero | 11 | 99 | 11.1 | 0.45 | 0 | 121 | - | HLA-DRB1 |
| 7 | 967226 | SNV | G | C | 1 | 23727 | Hetero | 14 | 70 | 20.0 | 0.14 | 0 | 48 | - | ADAP1 |
| 7 | 17920857 | SNV | T | C | 1 | 23727 | Hetero | 4 | 35 | 11.4 | 0.25 | 0 | 36 | - | SNX13 |
| 7 | 23293896 | SNV | A | G | 1 | 23727 | Hetero | 20 | 116 | 17.2 | 0.28 | 0 | 79 | Yes | GPNMB |
| 7 | 36570042 | SNV | C | A | 1 | 23727 | Hetero | 47 | 147 | 32.0 | 0.45 | 0 | 90 | Yes | AOAH |
| 7 | 42971769 | SNV | G | C | 1 | 23727 | Hetero | 85 | 338 | 25.1 | 0.24 | 0 | 248 | - | PSMA2 |
| 7 | 82545883 | SNV | T | C | 1 | 23727 | Hetero | 76 | 288 | 26.4 | 0.46 | 0 | 214 | Yes | PCLO |
| 7 | 88964905 | SNV | G | A | 1 | 23727 | Hetero | 39 | 190 | 20.5 | 0.39 | 0 | 143 | Yes | ZNF804B |
| 7 | 100345841 | SNV | G | A | 1 | 23727 | Hetero | 16 | 107 | 15.0 | 0.35 | 0 | 77 | - | ZAN |
| 7 | 101063289 | SNV | G | A | 1 | 23727 | Hetero | 25 | 123 | 20.3 | 0.40 | 0 | 69 | Yes | EMID2 |
| 7 | 104704479 | SNV | C | T | 1 | 23727 | Hetero | 45 | 196 | 23.0 | 0.47 | 0 | 132 | No | MLL5 |
| 7 | 104757383 | SNV | T | A | 1 | 23727 | Hetero | 27 | 110 | 24.5 | 0.22 | 0 | 54 | - | SRPK2 |
| 7 | 105516908 | SNV | C | T | 1 | 23727 | Hetero | 64 | 216 | 29.6 | 0.42 | 0 | 148 | Yes | ATXN7L1 |
| 7 | 132720695 | SNV | G | T | 1 | 23727 | Hetero | 13 | 122 | 10.7 | 0.47 | 0 | 79 | - | CHCHD3 |
| 7 | 139256640 | SNV | C | T | 1 | 23727 | Hetero | 16 | 70 | 22.9 | 0.39 | 0 | 58 | - | HIPK2 |
| 7 | 150758708 | SNV | C | G | 1 | 23727 | Hetero | 49 | 163 | 30.1 | 0.48 | 0 | 117 | - | SLC4A2 |
| 7 | 34194351 | Insertion | - | T | 1 | 23727 | Hetero | 5 | 49 | 10.2 | 0.40 | 0 | 30 | - | BMPER |
| 8 | 24384201 | SNV | G | A | 1 | 23727 | Hetero | 15 | 69 | 21.7 | 0.06 | 0 | 50 | - | ADAM7 |
| 8 | 48809740 | SNV | T | C | 1 | 23727 | Hetero | 30 | 146 | 20.5 | 0.10 | 0 | 106 | No | PRKDC |
| 8 | 55543296 | SNV | G | A | 1 | 23727 | Hetero | 13 | 66 | 19.7 | 0.20 | 0 | 37 | - | RP1 |
| 8 | 57080637 | SNV | G | T | 1 | 23727 | Hetero | 15 | 64 | 23.4 | 0.19 | 0 | 40 | Yes | PLAG1 |
| 8 | 57388256 | SNV | C | G | 1 | 23727 | Hetero | 16 | 96 | 16.7 | 0.47 | 0 | 55 | - | RP11-17A4.2 |
| 8 | 70617332 | SNV | G | A | 1 | 23727 | Hetero | 39 | 165 | 23.6 | 0.25 | 0 | 72 | Yes | SLCO5A1 |
| 8 | 75227885 | SNV | G | T | 1 | 23727 | Hetero | 23 | 123 | 18.7 | 0.40 | 0 | 55 | - | JPH1 |
| 8 | 77775653 | SNV | G | A | 1 | 23727 | Hetero | 56 | 349 | 16.0 | 0.48 | 0 | 187 | Yes | ZFHX4 |
| 8 | 89047792 | SNV | A | C | 1 | 23727 | Hetero | 17 | 110 | 15.5 | 0.42 | 0 | 72 | - | MMP16 |
| 8 | 91969691 | SNV | C | A | 1 | 23727 | Hetero | 14 | 62 | 22.6 | 0.31 | 0 | 42 | - | NECAB1 |
| 8 | 110412491 | SNV | A | G | 1 | 23727 | Hetero | 23 | 196 | 11.7 | 0.33 | 0 | 98 | Yes | PKHD1L1 |
| 8 | 110588142 | SNV | C | A | 1 | 23727 | Hetero | 42 | 195 | 21.5 | 0.35 | 0 | 105 | Yes | SYBU |
| 8 | 110984659 | SNV | C | T | 1 | 23727 | Hetero | 45 | 230 | 19.6 | 0.39 | 0 | 125 | No | KCNV1 |
| 8 | 125564323 | SNV | C | T | 1 | 23727 | Hetero | 13 | 77 | 16.9 | 0.40 | 0 | 38 | - | NDUFB9 |
| 8 | 141799549 | SNV | G | A | 1 | 23727 | Hetero | 59 | 329 | 17.9 | 0.39 | 0 | 187 | - | PTK2 |
| 8 | 142205723 | SNV | G | A | 1 | 23727 | Hetero | 31 | 160 | 19.4 | 0.25 | 0 | 80 | - | DENND3 |
| 8 | 145540518 | SNV | G | A | 1 | 23727 | Hetero | 19 | 109 | 17.4 | 0.50 | 0 | 63 | Yes | DGAT1 |
| 9 | 35056457 | SNV | T | A | 1 | 23727 | Hetero | 54 | 105 | 51.4 | 0.44 | 0 | 96 | - | VCP |
| 9 | 35703943 | SNV | G | A | 1 | 23727 | Hetero | 43 | 172 | 25.0 | 0.38 | 0 | 129 | - | TLN1 |
| 9 | 107298872 | SNV | G | C | 1 | 23727 | Hetero | 17 | 89 | 19.1 | 0.33 | 0 | 63 | Yes | OR13C3 |
| 9 | 139408959 | SNV | T | A | 1 | 23727 | Hetero | 37 | 77 | 48.1 | 0.38 | 0 | 72 | - | NOTCH1 |
| 9 | 139752832 | SNV | C | T | 1 | 23727 | Hetero | 44 | 377 | 11.7 | 0.46 | 0 | 227 | - | MAMDC4 |
| 10 | 1087455 | Deletion | A | - | 1 | 23727 | Hetero | 6 | 58 | 10.3 | 0.29 | 0 | 35 | - | IDI1 |
| 10 | 5008051 | SNV | G | A | 1 | 23727 | Hetero | 22 | 125 | 17.6 | 0.32 | 0 | 108 | - | AKR1C1 |
| 10 | 18964503 | SNV | A | T | 1 | 23727 | Hetero | 14 | 52 | 26.9 | 0.44 | 0 | 44 | - | ARL5B |
| 10 | 45471661 | SNV | G | A | 1 | 23727 | Hetero | 14 | 62 | 22.6 | 0.50 | 0 | 55 | - | RASSF4 |
| 10 | 91099436 | SNV | G | T | 1 | 23727 | Hetero | 45 | 184 | 24.5 | 0.48 | 0 | 142 | Yes | LIPA |
| 10 | 95326744 | SNV | C | A | 1 | 23727 | Hetero | 59 | 238 | 24.8 | 0.49 | 0 | 145 | No | O3FAR1 |
| 10 | 98380180 | SNV | C | T | 1 | 23727 | Hetero | 19 | 69 | 27.5 | 0.41 | 0 | 52 | Yes | PIK3AP1 |
| 11 | 994005 | SNV | C | T | 1 | 23727 | Hetero | 53 | 204 | 26.0 | 0.43 | 0 | 131 | - | AP2A2 |
| 11 | 1151627 | SNV | A | G | 1 | 23727 | Hetero | 20 | 90 | 22.2 | 0.50 | 0 | 57 | Yes | MUC5AC |
| 11 | 6079186 | SNV | G | C | 1 | 23727 | Hetero | 41 | 161 | 25.5 | 0.45 | 0 | 132 | - | ENSG00000262980 |
| 11 | 27519526 | SNV | T | C | 1 | 23727 | Hetero | 13 | 42 | 31.0 | 0.33 | 0 | 44 | - | LIN7C |
| 11 | 35829011 | SNV | C | A | 1 | 23727 | Hetero | 43 | 172 | 25.0 | 0.43 | 0 | 136 | - | TRIM44 |
| 11 | 44074181 | SNV | G | A | 1 | 23727 | Hetero | 16 | 60 | 26.7 | 0.30 | 0 | 40 | - | ACCSL |
| 11 | 57585404 | SNV | C | T | 1 | 23727 | Hetero | 60 | 220 | 27.3 | 0.37 | 0 | 182 | - | CTNND1 |
| 11 | 63706544 | SNV | G | A | 1 | 23727 | Hetero | 18 | 84 | 21.4 | 0.24 | 0 | 56 | - | NAA40 |
| 11 | 64072007 | SNV | C | T | 1 | 23727 | Hetero | 24 | 57 | 42.1 | 0.12 | 0 | 35 | - | C11orf20 |
| 11 | 66102652 | SNV | C | T | 1 | 23727 | Hetero | 22 | 93 | 23.7 | 0.33 | 0 | 58 | No | RIN1 |
| 11 | 66322021 | SNV | G | A | 1 | 23727 | Hetero | 26 | 103 | 25.2 | 0.46 | 0 | 87 | Yes | ACTN3 |
| 11 | 73724429 | SNV | G | A | 1 | 23727 | Hetero | 12 | 45 | 26.7 | 0.31 | 0 | 36 | - | C2CD3 |
| 11 | 76836929 | SNV | G | C | 1 | 23727 | Hetero | 34 | 111 | 30.6 | 0.37 | 0 | 68 | - | CAPN5 |
| 11 | 93545263 | SNV | G | A | 1 | 23727 | Hetero | 42 | 122 | 34.4 | 0.19 | 0 | 105 | - | MED17 |
| 11 | 113195394 | SNV | C | T | 1 | 23727 | Hetero | 36 | 157 | 22.9 | 0.38 | 0 | 88 | No | TTC12 |
| 11 | 119991358 | Deletion | G | - | 1 | 23727 | Hetero | 20 | 75 | 26.7 | 0.25 | 0 | 61 | Deletion | TRIM29 |
| 11 | 120858975 | SNV | C | T | 1 | 23727 | Hetero | 19 | 88 | 21.6 | 0.36 | 0 | 51 | - | GRIK4 |
| 11 | 74553050 | Insertion | - | T | 1 | 23727 | Hetero | 10 | 57 | 17.5 | 0.40 | 0 | 36 | - | RNF169 |
| 11 | 77372483 | Deletion | TG | - | 2 | 23727 | Hetero | 6 | 36 | 16.7 | 0.17 | 0 | 34 | - | RSF1 |
| 12 | 6673023 | SNV | C | T | 1 | 23727 | Hetero | 12 | 117 | 10.3 | 0.15 | 0 | 77 | - | NOP2 |
| 12 | 9010629 | SNV | G | A | 1 | 23727 | Hetero | 18 | 87 | 20.7 | 0.46 | 0 | 78 | Yes | A2ML1 |
| 12 | 9747351 | SNV | G | A | 1 | 23727 | Hetero | 20 | 65 | 30.8 | 0.50 | 0 | 36 | - | KLRB1 |
| 12 | 14929157 | SNV | A | C | 1 | 23727 | Hetero | 11 | 88 | 12.5 | 0.40 | 0 | 53 | - | H2AFJ |
| 12 | 39760261 | SNV | G | A | 1 | 23727 | Hetero | 34 | 131 | 26.0 | 0.29 | 0 | 94 | Yes | KIF21A |
| 12 | 54764269 | SNV | C | T | 1 | 23727 | Hetero | 48 | 168 | 28.6 | 0.47 | 0 | 93 | Possible splice site disruption | RP11-753H16.3 |
| 12 | 56113356 | SNV | A | G | 1 | 23727 | Hetero | 69 | 240 | 28.8 | 0.22 | 0 | 195 | Yes | BLOC1S1 |
| 12 | 72017923 | SNV | T | C | 1 | 23727 | Hetero | 23 | 78 | 29.5 | 0.17 | 0 | 45 | Yes | ZFC3H1 |
| 12 | 91444896 | SNV | C | T | 1 | 23727 | Hetero | 10 | 51 | 19.6 | 0.42 | 0 | 43 | - | KERA |
| 12 | 91558420 | SNV | T | C | 1 | 23727 | Hetero | 27 | 76 | 35.5 | 0.48 | 0 | 85 | Yes | DCN |
| 12 | 104326068 | SNV | A | G | 1 | 23727 | Hetero | 44 | 152 | 28.9 | 0.42 | 0 | 100 | Yes | HSP90B1 |
| 12 | 124809100 | SNV | G | C | 1 | 23727 | Hetero | 66 | 192 | 34.4 | 0.44 | 0 | 110 | - | NCOR2 |
| 12 | 133161054 | SNV | C | G | 1 | 23727 | Hetero | 26 | 101 | 25.7 | 0.30 | 0 | 62 | - | FBRSL1 |
| 13 | 32877837 | SNV | C | G | 1 | 23727 | Hetero | 10 | 67 | 14.9 | 0.38 | 0 | 43 | - | ZAR1L |
| 13 | 78188080 | SNV | A | G | 1 | 23727 | Hetero | 24 | 64 | 37.5 | 0.26 | 0 | 34 | Yes | SCEL |
| 14 | 21967278 | SNV | G | A | 1 | 23727 | Hetero | 82 | 225 | 36.4 | 0.26 | 0 | 169 | Yes | TOX4 |
| 14 | 31091393 | SNV | G | A | 1 | 23727 | Hetero | 17 | 57 | 29.8 | 0.42 | 0 | 50 | - | SCFD1 |
| 14 | 57116731 | SNV | C | T | 1 | 23727 | Hetero | 7 | 69 | 10.1 | 0.13 | 0 | 48 | - | C14orf101 |
| 14 | 74951322 | SNV | G | C | 1 | 23727 | Hetero | 10 | 58 | 17.2 | 0.30 | 0 | 65 | - | NPC2 |
| 14 | 88945775 | SNV | C | T | 1 | 23727 | Hetero | 62 | 188 | 33.0 | 0.33 | 0 | 126 | Yes | PTPN21 |
| 14 | 100745869 | SNV | G | T | 1 | 23727 | Hetero | 26 | 74 | 35.1 | 0.43 | 0 | 74 | - | YY1 |
| 14 | 23450976 | Insertion | - | T | 1 | 23727 | Hetero | 24 | 50 | 48.0 | 0.24 | 0 | 57 | Insertion | AJUBA |
| 15 | 33381007 | SNV | T | G | 1 | 23727 | Hetero | 31 | 159 | 19.5 | 0.40 | 0 | 75 | Yes | FMN1 |
| 15 | 40660518 | SNV | C | T | 1 | 23727 | Hetero | 15 | 120 | 12.5 | 0.40 | 0 | 96 | No | DISP2 |
| 15 | 40861942 | SNV | G | C | 1 | 23727 | Hetero | 70 | 317 | 22.1 | 0.40 | 0 | 170 | Yes | RPUSD2 |
| 15 | 99504549 | SNV | G | C | 1 | 23727 | Hetero | 25 | 95 | 26.3 | 0.33 | 0 | 86 | - | IGF1R |
| 15 | 99504968 | SNV | G | C | 1 | 23727 | Hetero | 41 | 192 | 21.4 | 0.45 | 0 | 99 | - | IGF1R |
| 15 | 102194265 | SNV | T | C | 1 | 23727 | Hetero | 15 | 82 | 18.3 | 0.47 | 0 | 47 | - | TARSL2 |
| 16 | 354342 | SNV | C | T | 1 | 23727 | Hetero | 25 | 106 | 23.6 | 0.30 | 0 | 69 | Yes | AXIN1 |
| 16 | 1725106 | SNV | G | A | 1 | 23727 | Hetero | 51 | 176 | 29.0 | 0.28 | 0 | 118 | - | CRAMP1L |
| 16 | 7637288 | SNV | G | C | 1 | 23727 | Hetero | 7 | 32 | 21.9 | 0.50 | 0 | 31 | Yes | RBFOX1 |
| 16 | 15138293 | Deletion | C | - | 1 | 23727 | Hetero | 14 | 60 | 23.3 | 0.07 | 0 | 41 | - | PDXDC1 |
| 16 | 21217079 | SNV | G | A | 1 | 23727 | Hetero | 37 | 171 | 21.6 | 0.38 | 0 | 167 | No | ZP2 |
| 16 | 31724658 | SNV | C | T | 1 | 23727 | Hetero | 76 | 259 | 29.3 | 0.29 | 0 | 152 | - | ZNF720 |
| 16 | 51175952 | Deletion | G | - | 1 | 23727 | Hetero | 14 | 50 | 28.0 | 0.27 | 0 | 43 | Deletion | SALL1 |
| 16 | 53301892 | SNV | A | G | 1 | 23727 | Hetero | 27 | 114 | 23.7 | 0.39 | 0 | 70 | Yes | CHD9 |
| 16 | 57798147 | SNV | C | T | 1 | 23727 | Hetero | 37 | 110 | 33.6 | 0.05 | 0 | 57 | Yes | KIFC3 |
| 16 | 67914566 | SNV | C | G | 1 | 23727 | Hetero | 34 | 174 | 19.5 | 0.36 | 0 | 121 | Yes | EDC4 |
| 16 | 67914821 | SNV | C | T | 1 | 23727 | Hetero | 47 | 178 | 26.4 | 0.37 | 0 | 110 | Yes | EDC4 |
| 16 | 67915356 | SNV | C | G | 1 | 23727 | Hetero | 22 | 98 | 22.4 | 0.32 | 0 | 102 | - | EDC4 |
| 16 | 67916297 | SNV | C | G | 1 | 23727 | Hetero | 60 | 219 | 27.4 | 0.47 | 0 | 119 | - | EDC4 |
| 16 | 71423748 | SNV | T | C | 1 | 23727 | Hetero | 30 | 147 | 20.4 | 0.43 | 0 | 99 | Yes | CALB2 |
| 16 | 85708484 | SNV | C | T | 1 | 23727 | Hetero | 46 | 173 | 26.6 | 0.49 | 0 | 152 | - | KIAA0182 |
| 16 | 87343951 | SNV | G | A | 1 | 23727 | Hetero | 20 | 67 | 29.9 | 0.29 | 0 | 36 | - | C16orf95 |
| 16 | 88599577 | SNV | A | G | 1 | 23727 | Hetero | 37 | 124 | 29.8 | 0.28 | 0 | 72 | Yes | ZFPM1 |
| 16 | 89778353 | Insertion | - | A | 1 | 23727 | Hetero | 41 | 145 | 28.3 | 0.40 | 0 | 61 | Insertion | C16orf7 |
| 17 | 3030849 | SNV | T | C | 1 | 23727 | Hetero | 62 | 247 | 25.1 | 0.44 | 0 | 178 | - | OR1G1 |
| 17 | 4891901 | SNV | C | T | 1 | 23727 | Hetero | 13 | 60 | 21.7 | 0.21 | 0 | 47 | - | INCA1 |
| 17 | 7578266 | SNV | T | A | 1 | 23727 | Hetero | 51 | 178 | 28.7 | 0.44 | 0 | 108 | Yes | TP53 |
| 17 | 7578271 | SNV | T | C | 1 | 23727 | Hetero | 39 | 175 | 22.3 | 0.44 | 0 | 107 | Yes | TP53 |
| 17 | 16852129 | SNV | C | A | 1 | 23727 | Hetero | 26 | 86 | 30.2 | 0.19 | 0 | 48 | Yes | TNFRSF13B |
| 17 | 18219150 | SNV | A | C | 1 | 23727 | Hetero | 104 | 383 | 27.2 | 0.43 | 0 | 258 | Yes | SMCR8 |
| 17 | 28523302 | Deletion | A | - | 1 | 23727 | Hetero | 7 | 50 | 14.0 | 0.13 | 0 | 32 | - | SLC6A4 |
| 17 | 37075285 | SNV | G | A | 1 | 23727 | Hetero | 210 | 436 | 48.2 | 0.25 | 0 | 302 | - | LASP1 |
| 17 | 41197482 | SNV | C | G | 1 | 23727 | Hetero | 21 | 80 | 26.3 | 0.39 | 0 | 35 | - | BRCA1 |
| 17 | 43923631 | SNV | C | A | 1 | 23727 | Hetero | 78 | 300 | 26.0 | 0.44 | 0 | 194 | No | MAPT-AS1 |
| 17 | 46691688 | SNV | G | T | 1 | 23727 | Hetero | 65 | 229 | 28.4 | 0.19 | 0 | 138 | Yes | HOXB8 |
| 17 | 53239932 | Deletion | A | - | 1 | 23727 | Hetero | 5 | 37 | 13.5 | 0.33 | 0 | 30 | - | STXBP4 |
| 17 | 56565657 | SNV | C | T | 1 | 23727 | Hetero | 42 | 173 | 24.3 | 0.33 | 0 | 87 | - | HSF5 |
| 17 | 72948071 | SNV | G | C | 1 | 23727 | Hetero | 18 | 85 | 21.2 | 0.50 | 0 | 42 | Yes | C17orf28 |
| 17 | 74324929 | SNV | G | A | 1 | 23727 | Hetero | 28 | 141 | 19.9 | 0.38 | 0 | 61 | Yes | PRPSAP1 |
| 17 | 79803059 | SNV | G | T | 1 | 23727 | Hetero | 38 | 163 | 23.3 | 0.47 | 0 | 79 | Yes | P4HB |
| 17 | 48046628 | Insertion | - | C | 1 | 23727 | Hetero | 34 | 134 | 25.4 | 0.43 | 0 | 79 | - | DLX4 |
| 18 | 2688689 | SNV | G | C | 1 | 23727 | Hetero | 14 | 56 | 25.0 | 0.33 | 0 | 36 | Yes | SMCHD1 |
| 18 | 9936956 | SNV | C | T | 1 | 23727 | Hetero | 27 | 159 | 17.0 | 0.38 | 0 | 74 | - | VAPA |
| 18 | 12115179 | SNV | T | C | 1 | 23727 | Hetero | 16 | 72 | 22.2 | 0.43 | 0 | 33 | - | ANKRD62 |
| 18 | 12978808 | SNV | A | G | 1 | 23727 | Hetero | 12 | 76 | 15.8 | 0.29 | 0 | 40 | No | SEH1L |
| 18 | 13763154 | SNV | C | T | 1 | 23727 | Hetero | 84 | 421 | 20.0 | 0.44 | 0 | 224 | - | RNMT |
| 18 | 13763230 | SNV | C | G | 1 | 23727 | Hetero | 84 | 424 | 19.8 | 0.46 | 0 | 215 | - | RNMT |
| 18 | 28993789 | SNV | G | A | 1 | 23727 | Hetero | 17 | 70 | 24.3 | 0.50 | 0 | 53 | - | DSG4 |
| 18 | 33243630 | SNV | G | A | 1 | 23727 | Hetero | 18 | 96 | 18.8 | 0.38 | 0 | 44 | Yes | GALNT1 |
| 18 | 33559099 | Deletion | A | - | 1 | 23727 | Hetero | 34 | 74 | 45.9 | 0.48 | 0 | 44 | - | C18orf21 |
| 18 | 74071075 | SNV | T | C | 1 | 23727 | Hetero | 116 | 195 | 59.5 | 0.38 | 0 | 100 | - | ZNF516 |
| 18 | 33850674 | Insertion | - | A | 1 | 23727 | Hetero | 17 | 86 | 19.8 | 0.30 | 0 | 33 | - | MOCOS |
| 19 | 1089594 | SNV | C | T | 1 | 23727 | Hetero | 24 | 118 | 20.3 | 0.38 | 0 | 77 | - | POLR2E |
| 19 | 7585280 | SNV | C | T | 1 | 23727 | Hetero | 23 | 103 | 22.3 | 0.19 | 0 | 56 | No | ZNF358 |
| 19 | 8670435 | SNV | C | G | 1 | 23727 | Hetero | 16 | 54 | 29.6 | 0.06 | 0 | 45 | - | ADAMTS10 |
| 19 | 9027159 | SNV | G | C | 1 | 23727 | Hetero | 17 | 45 | 37.8 | 0.15 | 0 | 30 | - | MUC16 |
| 19 | 13368248 | SNV | G | A | 1 | 23727 | Hetero | 39 | 158 | 24.7 | 0.46 | 0 | 104 | No | CACNA1A |
| 19 | 19619588 | SNV | A | T | 1 | 23727 | Hetero | 20 | 63 | 31.7 | 0.48 | 0 | 50 | - | GATAD2A |
| 19 | 23922544 | SNV | T | C | 1 | 23727 | Hetero | 17 | 87 | 19.5 | 0.36 | 0 | 61 | - | ZNF681 |
| 19 | 33696402 | SNV | C | T | 1 | 23727 | Hetero | 49 | 189 | 25.9 | 0.44 | 0 | 94 | No | LRP3 |
| 19 | 51503875 | SNV | C | A | 1 | 23727 | Hetero | 28 | 130 | 21.5 | 0.39 | 0 | 66 | Yes | KLK8 |
| 19 | 54487557 | SNV | T | C | 1 | 23727 | Hetero | 41 | 148 | 27.7 | 0.40 | 0 | 106 | - | CACNG8 |
| 19 | 55738716 | SNV | C | T | 1 | 23727 | Hetero | 30 | 112 | 26.8 | 0.29 | 0 | 63 | Yes | TMEM86B |
| 19 | 56104049 | SNV | C | T | 1 | 23727 | Hetero | 34 | 181 | 18.8 | 0.43 | 0 | 120 | Yes | FIZ1 |
| 19 | 58258397 | SNV | C | G | 1 | 23727 | Hetero | 55 | 217 | 25.3 | 0.46 | 0 | 117 | - | ZNF776 |
| 20 | 462592 | SNV | C | T | 1 | 23727 | Hetero | 13 | 53 | 24.5 | 0.41 | 0 | 35 | - | CSNK2A1 |
| 20 | 6018401 | SNV | G | A | 1 | 23727 | Hetero | 29 | 154 | 18.8 | 0.47 | 0 | 69 | - | CRLS1 |
| 20 | 10626655 | SNV | A | G | 1 | 23727 | Hetero | 15 | 72 | 20.8 | 0.32 | 0 | 31 | Yes | JAG1 |
| 20 | 23728330 | SNV | C | T | 1 | 23727 | Hetero | 36 | 183 | 19.7 | 0.49 | 0 | 129 | - | CST1 |
| 20 | 24646099 | SNV | G | A | 1 | 23727 | Hetero | 65 | 411 | 15.8 | 0.47 | 0 | 194 | Yes | SYNDIG1 |
| 20 | 30102283 | SNV | G | C | 1 | 23727 | Hetero | 41 | 217 | 18.9 | 0.41 | 0 | 109 | - | HM13 |
| 20 | 44528498 | SNV | G | A | 1 | 23727 | Hetero | 19 | 87 | 21.8 | 0.32 | 0 | 35 | Yes | PLTP |
| 20 | 47241246 | SNV | T | A | 1 | 23727 | Hetero | 49 | 194 | 25.3 | 0.39 | 0 | 135 | - | PREX1 |
| 20 | 56140768 | SNV | G | T | 1 | 23727 | Hetero | 72 | 365 | 19.7 | 0.23 | 0 | 172 | Yes | PCK1 |
| 20 | 58441566 | SNV | G | T | 1 | 23727 | Hetero | 22 | 104 | 21.2 | 0.44 | 0 | 48 | Yes | SYCP2 |
| 20 | 58883578 | SNV | G | T | 1 | 23727 | Hetero | 64 | 311 | 20.6 | 0.42 | 0 | 139 | - | RP5-1043L13.1 |
| 20 | 60886681 | SNV | G | T | 1 | 23727 | Hetero | 16 | 97 | 16.5 | 0.44 | 0 | 62 | No | LAMA5 |
| 20 | 60965350 | SNV | G | C | 1 | 23727 | Hetero | 38 | 159 | 23.9 | 0.43 | 0 | 61 | - | CABLES2 |
| 20 | 62194430 | SNV | G | A | 1 | 23727 | Hetero | 57 | 237 | 24.1 | 0.49 | 0 | 96 | No | RP4-697K14.7 |
| 20 | 62517336 | SNV | C | T | 1 | 23727 | Hetero | 20 | 112 | 17.9 | 0.33 | 0 | 49 | - | TPD52L2 |
| 20 | 47652530 | Insertion | - | A | 1 | 23727 | Hetero | 5 | 39 | 12.8 | 0.20 | 0 | 32 | - | ARFGEF2 |
| 21 | 45089829 | SNV | G | A | 1 | 23727 | Hetero | 10 | 53 | 18.9 | 0.45 | 0 | 35 | No | RRP1B |
| 22 | 19207487 | SNV | G | A | 1 | 23727 | Hetero | 22 | 80 | 27.5 | 0.33 | 0 | 47 | No | CLTCL1 |
| 22 | 25755886 | SNV | G | A | 1 | 23727 | Hetero | 44 | 185 | 23.8 | 0.45 | 0 | 116 | No | LRP5L |
| 22 | 38026978 | SNV | C | T | 1 | 23727 | Hetero | 8 | 54 | 14.8 | 0.45 | 0 | 43 | Yes | GGA1 |
| 22 | 38934479 | SNV | G | C | 1 | 23727 | Hetero | 23 | 92 | 25.0 | 0.48 | 0 | 64 | - | DMC1 |
| X | 41206716 | SNV | C | T | 1 | 23727 | Hetero | 27 | 53 | 50.9 | 0.45 | 0 | 36 | - | DDX3X |
| X | 75651197 | SNV | G | C | 1 | 23727 | Hetero | 59 | 118 | 50.0 | 0.41 | 0 | 108 | Yes | MAGEE1 |
| X | 78216776 | SNV | C | T | 1 | 23727 | Hetero | 56 | 108 | 51.9 | 0.40 | 0 | 99 | No | P2RY10 |
| X | 83129541 | SNV | C | T | 1 | 23727 | Hetero | 41 | 94 | 43.6 | 0.44 | 0 | 61 | Yes | CYLC1 |
| X | 153053480 | SNV | C | A | 1 | 23727 | Hetero | 34 | 64 | 53.1 | 0.30 | 0 | 34 | - | IDH3G |
| 1 | 35881063 | SNV | C | T | 1 | 24311 | Hetero | 14 | 117 | 12.0 | 0.29 | 0 | 69 | - | ZMYM4 |
| 1 | 50883805 | Deletion | T | - | 1 | 24311 | Hetero | 10 | 82 | 12.2 | 0.20 | 0 | 44 | - | DMRTA2 |
| 1 | 53292476 | SNV | A | G | 1 | 24311 | Hetero | 6 | 60 | 10.0 | 0.17 | 0 | 45 | - | ZYG11B |
| 1 | 59248428 | SNV | A | G | 1 | 24311 | Hetero | 26 | 203 | 12.8 | 0.41 | 0 | 166 | No | JUN |
| 1 | 74665487 | SNV | T | A | 1 | 24311 | Hetero | 20 | 159 | 12.6 | 0.29 | 0 | 130 | No | FPGT |
| 1 | 91727978 | SNV | T | C | 1 | 24311 | Hetero | 7 | 63 | 11.1 | 0.29 | 0 | 50 | - | HFM1 |
| 1 | 114304710 | SNV | T | C | 1 | 24311 | Hetero | 21 | 129 | 16.3 | 0.46 | 0 | 100 | - | RSBN1 |
| 1 | 120279813 | SNV | A | T | 1 | 24311 | Hetero | 17 | 126 | 13.5 | 0.26 | 0 | 107 | Yes | PHGDH |
| 1 | 151018650 | SNV | C | T | 1 | 24311 | Hetero | 17 | 151 | 11.3 | 0.32 | 0 | 100 | - | BNIPL |
| 1 | 160342600 | SNV | C | G | 1 | 24311 | Hetero | 15 | 133 | 11.3 | 0.25 | 0 | 69 | - | NHLH1 |
| 1 | 169345988 | SNV | G | C | 1 | 24311 | Hetero | 14 | 123 | 11.4 | 0.47 | 0 | 73 | Yes | BLZF1 |
| 1 | 212277555 | Deletion | G | - | 1 | 24311 | Hetero | 5 | 46 | 10.9 | 0.20 | 0 | 31 | - | DTL |
| 1 | 220340755 | SNV | G | C | 1 | 24311 | Hetero | 22 | 177 | 12.4 | 0.32 | 0 | 137 | - | RAB3GAP2 |
| 1 | 228336246 | SNV | A | T | 1 | 24311 | Hetero | 36 | 333 | 10.8 | 0.48 | 0 | 187 | - | GUK1 |
| 1 | 228505691 | SNV | G | C | 1 | 24311 | Hetero | 22 | 209 | 10.5 | 0.41 | 0 | 147 | Yes | OBSCN |
| 1 | 236714676 | SNV | A | G | 1 | 24311 | Hetero | 19 | 149 | 12.8 | 0.43 | 0 | 102 | - | LGALS8 |
| 1 | 247694130 | Insertion | - | CG | 2 | 24311 | Hetero | 10 | 63 | 15.9 | 0.20 | 0 | 70 | - | GCSAML |
| 2 | 3624270 | SNV | A | G | 1 | 24311 | Hetero | 26 | 204 | 12.7 | 0.35 | 0 | 172 | - | RPS7 |
| 2 | 130909881 | SNV | G | A | 1 | 24311 | Hetero | 10 | 96 | 10.4 | 0.42 | 0 | 53 | - | SMPD4 |
| 2 | 219316694 | SNV | G | A | 1 | 24311 | Hetero | 16 | 103 | 15.5 | 0.50 | 0 | 66 | - | VIL1 |
| 2 | 227861176 | SNV | G | C | 1 | 24311 | Hetero | 7 | 68 | 10.3 | 0.25 | 0 | 66 | - | RHBDD1 |
| 2 | 228771820 | Deletion | T | - | 1 | 24311 | Hetero | 10 | 96 | 10.4 | 0.09 | 0 | 58 | - | WDR69 |
| 2 | 203000716 | Insertion | - | A | 1 | 24311 | Hetero | 4 | 39 | 10.3 | 0.50 | 0 | 40 | - | AC079354.1 |
| 2 | 44999603 | Insertion | - | T | 1 | 24311 | Hetero | 9 | 88 | 10.2 | 0.30 | 0 | 50 | - | CAMKMT |
| 3 | 44957699 | SNV | G | A | 1 | 24311 | Hetero | 15 | 126 | 11.9 | 0.42 | 0 | 101 | - | ZDHHC3 |
| 3 | 116164082 | SNV | C | T | 1 | 24311 | Hetero | 10 | 93 | 10.8 | 0.31 | 0 | 50 | - | LSAMP |
| 3 | 151150926 | SNV | C | G | 1 | 24311 | Hetero | 10 | 92 | 10.9 | 0.40 | 0 | 65 | - | MED12L |
| 3 | 196663950 | SNV | G | C | 1 | 24311 | Hetero | 38 | 320 | 11.9 | 0.30 | 0 | 219 | Yes | NCBP2 |
| 3 | 14184307 | Insertion | - | A | 1 | 24311 | Hetero | 14 | 94 | 14.9 | 0.39 | 0 | 57 | - | TMEM43 |
| 3 | 179481738 | Insertion | - | A | 1 | 24311 | Hetero | 9 | 63 | 14.3 | 0.50 | 0 | 45 | - | USP13 |
| 4 | 39850424 | SNV | T | A | 1 | 24311 | Hetero | 8 | 62 | 12.9 | 0.11 | 0 | 59 | - | PDS5A |
| 4 | 186508458 | Insertion | - | T | 1 | 24311 | Hetero | 8 | 50 | 16.0 | 0.38 | 0 | 37 | - | SORBS2 |
| 4 | 99218169 | Insertion | - | T | 1 | 24311 | Hetero | 10 | 74 | 13.5 | 0.18 | 0 | 47 | - | RAP1GDS1 |
| 5 | 142150011 | SNV | G | C | 1 | 24311 | Hetero | 10 | 95 | 10.5 | 0.36 | 0 | 87 | - | ARHGAP26 |
| 5 | 166711939 | SNV | C | T | 1 | 24311 | Hetero | 33 | 184 | 17.9 | 0.34 | 0 | 192 | Yes | ODZ2 |
| 5 | 180002603 | SNV | A | T | 1 | 24311 | Hetero | 12 | 91 | 13.2 | 0.43 | 0 | 91 | - | CNOT6 |
| 5 | 156820216 | Deletion | AA | - | 2 | 24311 | Hetero | 8 | 77 | 10.4 | 0.25 | 0 | 34 | - | CYFIP2 |
| 6 | 656202 | SNV | G | T | 1 | 24311 | Hetero | 19 | 178 | 10.7 | 0.43 | 0 | 173 | Yes | EXOC2 |
| 6 | 16144074 | SNV | A | G | 1 | 24311 | Hetero | 6 | 58 | 10.3 | 0.33 | 0 | 32 | No | MYLIP |
| 6 | 32939534 | SNV | C | T | 1 | 24311 | Hetero | 16 | 114 | 14.0 | 0.45 | 0 | 69 | No | BRD2 |
| 6 | 55378076 | SNV | C | G | 1 | 24311 | Hetero | 6 | 49 | 12.2 | 0.43 | 0 | 56 | - | HMGCLL1 |
| 6 | 56496094 | SNV | A | T | 1 | 24311 | Hetero | 26 | 176 | 14.8 | 0.35 | 0 | 199 | Yes | DST |
| 6 | 146480509 | SNV | C | T | 1 | 24311 | Hetero | 13 | 88 | 14.8 | 0.40 | 0 | 55 | No | GRM1 |
| 6 | 152476097 | SNV | C | A | 1 | 24311 | Hetero | 17 | 156 | 10.9 | 0.48 | 0 | 151 | Yes | SYNE1 |
| 6 | 32147292 | Deletion | TT | - | 2 | 24311 | Hetero | 12 | 102 | 11.8 | 0.27 | 0 | 53 | - | RNF5 |
| 6 | 37299299 | Insertion | - | T | 1 | 24311 | Hetero | 9 | 66 | 13.6 | 0.45 | 0 | 46 | - | TBC1D22B |
| 7 | 100881201 | SNV | T | G | 1 | 24311 | Hetero | 15 | 124 | 12.1 | 0.07 | 0 | 67 | - | CLDN15 |
| 7 | 147335934 | Deletion | T | - | 1 | 24311 | Hetero | 6 | 47 | 12.8 | 0.43 | 0 | 46 | Deletion | CNTNAP2 |
| 8 | 22472299 | SNV | G | C | 1 | 24311 | Hetero | 19 | 171 | 11.1 | 0.11 | 0 | 124 | - | KIAA1967 |
| 8 | 145689657 | SNV | C | T | 1 | 24311 | Hetero | 38 | 320 | 11.9 | 0.30 | 0 | 209 | No | CYHR1 |
| 9 | 33465830 | SNV | C | T | 1 | 24311 | Hetero | 32 | 190 | 16.8 | 0.47 | 0 | 138 | No | NOL6 |
| 9 | 130428606 | SNV | A | G | 1 | 24311 | Hetero | 15 | 137 | 10.9 | 0.33 | 0 | 95 | - | STXBP1 |
| 9 | 134398681 | SNV | G | A | 1 | 24311 | Hetero | 7 | 60 | 11.7 | 0.29 | 0 | 46 | - | POMT1 |
| 9 | 140991056 | SNV | G | A | 1 | 24311 | Hetero | 23 | 134 | 17.2 | 0.31 | 0 | 103 | Yes | CACNA1B |
| 10 | 88257016 | SNV | T | C | 1 | 24311 | Hetero | 16 | 115 | 13.9 | 0.50 | 0 | 92 | Yes | WAPAL |
| 10 | 52514701 | Insertion | - | GA | 2 | 24311 | Hetero | 11 | 54 | 20.4 | 0.38 | 0 | 44 | - | ASAH2B |
| 11 | 607956 | SNV | G | A | 1 | 24311 | Hetero | 36 | 269 | 13.4 | 0.49 | 0 | 194 | Yes | PHRF1 |
| 11 | 3249554 | SNV | C | T | 1 | 24311 | Hetero | 11 | 80 | 13.8 | 0.45 | 0 | 48 | Yes | AC109309.4 |
| 11 | 5718522 | SNV | A | G | 1 | 24311 | Hetero | 11 | 59 | 18.6 | 0.31 | 0 | 57 | No | TRIM5 |
| 11 | 10515159 | SNV | A | T | 1 | 24311 | Hetero | 26 | 236 | 11.0 | 0.37 | 0 | 139 | Yes | AMPD3 |
| 11 | 13753783 | SNV | A | G | 1 | 24311 | Hetero | 9 | 65 | 13.8 | 0.45 | 0 | 39 | - | FAR1 |
| 11 | 113563808 | SNV | C | A | 1 | 24311 | Hetero | 13 | 96 | 13.5 | 0.33 | 0 | 80 | Yes | TMPRSS5 |
| 12 | 32137802 | SNV | C | A | 1 | 24311 | Hetero | 12 | 120 | 10.0 | 0.43 | 0 | 96 | Yes | KIAA1551 |
| 12 | 48529131 | SNV | A | T | 1 | 24311 | Hetero | 16 | 126 | 12.7 | 0.40 | 0 | 118 | Yes | PFKM |
| 12 | 68052854 | SNV | A | G | 1 | 24311 | Hetero | 8 | 73 | 11.0 | 0.25 | 0 | 57 | - | DYRK2 |
| 12 | 88898961 | SNV | T | A | 1 | 24311 | Hetero | 22 | 214 | 10.3 | 0.29 | 0 | 147 | - | KITLG |
| 12 | 6980075 | Insertion | - | A | 1 | 24311 | Hetero | 10 | 91 | 11.0 | 0.18 | 0 | 47 | - | TPI1 |
| 12 | 70825286 | Insertion | - | T | 1 | 24311 | Hetero | 6 | 59 | 10.2 | 0.29 | 0 | 35 | - | KCNMB4 |
| 13 | 46057492 | SNV | T | C | 1 | 24311 | Hetero | 5 | 47 | 10.6 | 0.29 | 0 | 34 | - | COG3 |
| 13 | 78130714 | SNV | C | T | 1 | 24311 | Hetero | 14 | 117 | 12.0 | 0.38 | 0 | 117 | - | SCEL |
| 13 | 109610028 | SNV | C | A | 1 | 24311 | Hetero | 15 | 134 | 11.2 | 0.28 | 0 | 126 | - | MYO16 |
| 14 | 45716121 | SNV | C | A | 1 | 24311 | Hetero | 9 | 70 | 12.9 | 0.40 | 0 | 46 | No | MIS18BP1 |
| 15 | 74923389 | SNV | G | T | 1 | 24311 | Hetero | 12 | 109 | 11.0 | 0.25 | 0 | 100 | - | CLK3 |
| 15 | 102196231 | SNV | T | C | 1 | 24311 | Hetero | 35 | 199 | 17.6 | 0.46 | 0 | 149 | - | TARSL2 |
| 16 | 58036357 | SNV | C | T | 1 | 24311 | Hetero | 21 | 144 | 14.6 | 0.27 | 0 | 102 | - | USB1 |
| 16 | 69151931 | SNV | T | C | 1 | 24311 | Hetero | 18 | 138 | 13.0 | 0.30 | 0 | 90 | - | HAS3 |
| 16 | 69199216 | SNV | C | A | 1 | 24311 | Hetero | 11 | 69 | 15.9 | 0.15 | 0 | 65 | - | CIRH1A |
| 16 | 71669518 | Insertion | - | A | 1 | 24311 | Hetero | 4 | 39 | 10.3 | 0.40 | 0 | 31 | - | MARVELD3 |
| 17 | 6915715 | SNV | G | C | 1 | 24311 | Hetero | 9 | 85 | 10.6 | 0.50 | 0 | 61 | - | RP11-589P10.7 |
| 17 | 7578190 | SNV | T | C | 1 | 24311 | Hetero | 18 | 117 | 15.4 | 0.32 | 0 | 73 | Yes | TP53 |
| 17 | 7578263 | SNV | G | A | 1 | 24311 | Hetero | 19 | 175 | 10.9 | 0.30 | 0 | 111 | Yes | TP53 |
| 17 | 11455279 | SNV | G | A | 1 | 24311 | Hetero | 17 | 136 | 12.5 | 0.42 | 0 | 105 | - | SHISA6 |
| 17 | 16021225 | SNV | G | C | 1 | 24311 | Hetero | 16 | 99 | 16.2 | 0.17 | 0 | 69 | Yes | NCOR1 |
| 17 | 27401118 | SNV | G | A | 1 | 24311 | Hetero | 19 | 161 | 11.8 | 0.39 | 0 | 106 | Yes | MYO18A |
| 18 | 56963373 | SNV | A | G | 1 | 24311 | Hetero | 42 | 95 | 44.2 | 0.37 | 0 | 72 | - | CPLX4 |
| 18 | 72776127 | SNV | G | T | 1 | 24311 | Hetero | 23 | 167 | 13.8 | 0.38 | 0 | 129 | Yes | ZNF407 |
| 18 | 74726672 | SNV | T | C | 1 | 24311 | Hetero | 8 | 49 | 16.3 | 0.13 | 0 | 38 | - | MBP |
| 18 | 51905869 | Insertion | - | T | 1 | 24311 | Hetero | 7 | 66 | 10.6 | 0.14 | 0 | 38 | - | C18orf54 |
| 19 | 8160821 | SNV | G | A | 1 | 24311 | Hetero | 11 | 85 | 12.9 | 0.46 | 0 | 53 | - | FBN3 |
| 20 | 44670097 | SNV | C | T | 1 | 24311 | Hetero | 19 | 160 | 11.9 | 0.35 | 0 | 78 | No | SLC12A5 |
| 21 | 30439250 | SNV | T | C | 1 | 24311 | Hetero | 9 | 64 | 14.1 | 0.50 | 0 | 48 | Yes | CCT8 |
| 21 | 30717064 | SNV | C | T | 1 | 24311 | Hetero | 6 | 51 | 11.8 | 0.17 | 0 | 49 | - | BACH1 |
| 21 | 31588039 | SNV | A | T | 1 | 24311 | Hetero | 17 | 146 | 11.6 | 0.33 | 0 | 121 | Yes | CLDN8 |
| 22 | 31835865 | SNV | C | T | 1 | 24311 | Hetero | 18 | 164 | 11.0 | 0.33 | 0 | 97 | - | DRG1 |
| 22 | 36139402 | SNV | A | C | 1 | 24311 | Hetero | 18 | 72 | 25.0 | 0.35 | 0 | 64 | - | RBFOX2 |
| X | 43605440 | SNV | C | G | 1 | 24311 | Hetero | 15 | 65 | 23.1 | 0.47 | 0 | 36 | - | MAOA |
| X | 53620491 | SNV | G | A | 1 | 24311 | Hetero | 9 | 45 | 20.0 | 0.46 | 0 | 32 | Yes | HUWE1 |
| X | 75649665 | SNV | G | C | 1 | 24311 | Hetero | 18 | 116 | 15.5 | 0.35 | 0 | 79 | Yes | MAGEE1 |
| X | 139586359 | SNV | G | T | 1 | 24311 | Hetero | 15 | 61 | 24.6 | 0.18 | 0 | 69 | Yes | SOX3 |
| X | 84510289 | Insertion | - | T | 1 | 24311 | Hetero | 9 | 45 | 20.0 | 0.40 | 0 | 35 | Insertion | ZNF711 |
| 1 | 6161100 | SNV | T | A | 1 | 24411 | Hetero | 36 | 184 | 19.6 | 0.34 | 0 | 109 | - | KCNAB2 |
| 1 | 21582449 | SNV | G | A | 1 | 24411 | Hetero | 120 | 189 | 63.5 | 0.20 | 0 | 128 | No | ECE1 |
| 1 | 47746749 | SNV | G | A | 1 | 24411 | Hetero | 18 | 142 | 12.7 | 0.45 | 0 | 105 | Yes | STIL |
| 1 | 51032886 | SNV | G | C | 1 | 24411 | Hetero | 8 | 65 | 12.3 | 0.22 | 0 | 50 | Yes | FAF1 |
| 1 | 52551991 | SNV | C | A | 1 | 24411 | Hetero | 11 | 91 | 12.1 | 0.46 | 0 | 74 | - | BTF3L4 |
| 1 | 76260601 | SNV | A | G | 1 | 24411 | Hetero | 6 | 53 | 11.3 | 0.50 | 0 | 49 | - | RABGGTB |
| 1 | 79002928 | SNV | G | T | 1 | 24411 | Hetero | 6 | 60 | 10.0 | 0.38 | 0 | 78 | - | PTGFR |
| 1 | 89618056 | SNV | G | C | 1 | 24411 | Hetero | 28 | 110 | 25.5 | 0.38 | 0 | 110 | Yes | GBP7 |
| 1 | 117118471 | SNV | G | A | 1 | 24411 | Hetero | 10 | 73 | 13.7 | 0.42 | 0 | 59 | - | IGSF3 |
| 1 | 121309926 | SNV | T | C | 1 | 24411 | Hetero | 11 | 56 | 19.6 | 0.31 | 0 | 35 | - | EMBP1 |
| 1 | 151377000 | SNV | T | C | 1 | 24411 | Hetero | 43 | 89 | 48.3 | 0.46 | 0 | 42 | - | POGZ |
| 1 | 158615093 | SNV | T | C | 1 | 24411 | Hetero | 51 | 154 | 33.1 | 0.33 | 0 | 159 | Yes | SPTA1 |
| 1 | 160397457 | SNV | G | T | 1 | 24411 | Hetero | 52 | 101 | 51.5 | 0.23 | 0 | 73 | - | VANGL2 |
| 1 | 180248975 | Deletion | A | - | 1 | 24411 | Hetero | 6 | 59 | 10.2 | 0.33 | 0 | 34 | - | LHX4 |
| 1 | 205739456 | SNV | T | C | 1 | 24411 | Hetero | 42 | 306 | 13.7 | 0.43 | 0 | 212 | - | RAB7L1 |
| 1 | 212277632 | Deletion | A | - | 1 | 24411 | Hetero | 6 | 58 | 10.3 | 0.33 | 0 | 42 | - | DTL |
| 1 | 247003942 | SNV | G | C | 1 | 24411 | Hetero | 110 | 250 | 44.0 | 0.39 | 0 | 115 | - | AHCTF1 |
| 1 | 247461266 | SNV | C | T | 1 | 24411 | Hetero | 18 | 131 | 13.7 | 0.29 | 0 | 87 | - | ZNF496 |
| 1 | 1335712 | Insertion | - | CA | 2 | 24411 | Hetero | 19 | 161 | 11.8 | 0.19 | 0 | 73 | - | RP4-758J18.2 |
| 1 | 17299006 | Insertion | - | C | 1 | 24411 | Hetero | 5 | 46 | 10.9 | 0.40 | 0 | 43 | - | CROCC |
| 1 | 204393465 | Insertion | - | A | 1 | 24411 | Hetero | 7 | 70 | 10.0 | 0.14 | 0 | 48 | - | RP11-739N20.2 |
| 1 | 93602675 | Insertion | - | A | 1 | 24411 | Hetero | 4 | 38 | 10.5 | 0.40 | 0 | 32 | - | MTF2 |
| 2 | 50280722 | SNV | T | A | 1 | 24411 | Hetero | 21 | 109 | 19.3 | 0.36 | 0 | 76 | Yes | NRXN1 |
| 2 | 71595625 | SNV | G | A | 1 | 24411 | Hetero | 9 | 68 | 13.2 | 0.11 | 0 | 44 | No | ZNF638 |
| 2 | 73455384 | SNV | T | G | 1 | 24411 | Hetero | 22 | 128 | 17.2 | 0.43 | 0 | 82 | - | PRADC1 |
| 2 | 79750312 | SNV | C | T | 1 | 24411 | Hetero | 15 | 95 | 15.8 | 0.13 | 0 | 84 | - | CTNNA2 |
| 2 | 85282039 | SNV | C | T | 1 | 24411 | Hetero | 79 | 123 | 64.2 | 0.38 | 0 | 73 | - | KCMF1 |
| 2 | 100625329 | SNV | T | A | 1 | 24411 | Hetero | 108 | 121 | 89.3 | 0.45 | 0 | 112 | Yes | AFF3 |
| 2 | 118867324 | SNV | C | T | 1 | 24411 | Hetero | 21 | 99 | 21.2 | 0.33 | 0 | 78 | - | INSIG2 |
| 2 | 120197828 | SNV | C | T | 1 | 24411 | Hetero | 12 | 75 | 16.0 | 0.43 | 0 | 49 | No | SCTR |
| 2 | 170496309 | SNV | C | A | 1 | 24411 | Hetero | 90 | 102 | 88.2 | 0.41 | 0 | 45 | - | PPIG |
| 2 | 171572157 | SNV | C | T | 1 | 24411 | Hetero | 59 | 66 | 89.4 | 0.48 | 0 | 55 | - | SP5 |
| 2 | 179614300 | SNV | T | C | 1 | 24411 | Hetero | 11 | 104 | 10.6 | 0.33 | 0 | 68 | Yes | TTN |
| 2 | 203428969 | SNV | A | T | 1 | 24411 | Hetero | 42 | 47 | 89.4 | 0.46 | 0 | 35 | - | BMPR2 |
| 2 | 210559629 | SNV | T | G | 1 | 24411 | Hetero | 33 | 202 | 16.3 | 0.46 | 0 | 185 | Yes | MAP2 |
| 2 | 101521173 | Insertion | - | T | 1 | 24411 | Hetero | 137 | 210 | 65.2 | 0.22 | 0 | 158 | - | NPAS2 |
| 2 | 128949840 | Insertion | - | T | 1 | 24411 | Hetero | 8 | 68 | 11.8 | 0.36 | 0 | 38 | - | UGGT1 |
| 2 | 238688523 | Insertion | - | T | 1 | 24411 | Hetero | 9 | 82 | 11.0 | 0.40 | 0 | 48 | - | LRRFIP1 |
| 2 | 44445604 | Insertion | - | T | 1 | 24411 | Hetero | 33 | 85 | 38.8 | 0.44 | 0 | 41 | - | PPM1B |
| 3 | 9860731 | SNV | G | A | 1 | 24411 | Hetero | 155 | 248 | 62.5 | 0.31 | 0 | 188 | - | ARPC4-TTLL3 |
| 3 | 13612780 | SNV | A | T | 1 | 24411 | Hetero | 87 | 137 | 63.5 | 0.46 | 0 | 108 | Yes | FBLN2 |
| 3 | 15275381 | SNV | A | T | 1 | 24411 | Hetero | 25 | 114 | 21.9 | 0.44 | 0 | 76 | Yes | CAPN7 |
| 3 | 37032881 | SNV | T | C | 1 | 24411 | Hetero | 19 | 174 | 10.9 | 0.48 | 0 | 164 | Yes | EPM2AIP1 |
| 3 | 38797338 | SNV | C | G | 1 | 24411 | Hetero | 11 | 35 | 31.4 | 0.31 | 0 | 52 | Yes | SCN10A |
| 3 | 40576506 | SNV | T | G | 1 | 24411 | Hetero | 26 | 58 | 44.8 | 0.37 | 0 | 85 | - | ZNF621 |
| 3 | 42794171 | SNV | T | A | 1 | 24411 | Hetero | 28 | 59 | 47.5 | 0.49 | 0 | 66 | Yes | CCDC13 |
| 3 | 49571621 | Deletion | T | - | 1 | 24411 | Hetero | 8 | 68 | 11.8 | 0.38 | 0 | 54 | - | DAG1 |
| 3 | 78795951 | SNV | G | C | 1 | 24411 | Hetero | 19 | 54 | 35.2 | 0.32 | 0 | 77 | Yes | ROBO1 |
| 3 | 109049245 | SNV | T | G | 1 | 24411 | Hetero | 10 | 61 | 16.4 | 0.29 | 0 | 31 | - | DPPA4 |
| 3 | 127786733 | SNV | G | A | 1 | 24411 | Hetero | 19 | 169 | 11.2 | 0.33 | 0 | 69 | - | SEC61A1 |
| 3 | 138195100 | SNV | A | G | 1 | 24411 | Hetero | 40 | 90 | 44.4 | 0.29 | 0 | 38 | Yes | ESYT3 |
| 3 | 167413386 | SNV | G | C | 1 | 24411 | Hetero | 128 | 297 | 43.1 | 0.18 | 0 | 145 | Yes | PDCD10 |
| 3 | 188426191 | SNV | G | A | 1 | 24411 | Hetero | 39 | 373 | 10.5 | 0.36 | 0 | 174 | Yes | LPP |
| 3 | 190573380 | SNV | G | A | 1 | 24411 | Hetero | 41 | 296 | 13.9 | 0.48 | 0 | 153 | Yes | GMNC |
| 3 | 186918238 | Insertion | - | A | 1 | 24411 | Hetero | 16 | 140 | 11.4 | 0.41 | 0 | 53 | - | RP11-208N14.4 |
| 4 | 13592180 | SNV | A | G | 1 | 24411 | Hetero | 43 | 76 | 56.6 | 0.31 | 0 | 49 | - | BOD1L1 |
| 4 | 76692139 | SNV | A | G | 1 | 24411 | Hetero | 23 | 156 | 14.7 | 0.33 | 0 | 86 | - | USO1 |
| 4 | 84227379 | SNV | C | T | 1 | 24411 | Hetero | 30 | 270 | 11.1 | 0.35 | 0 | 139 | Yes | HPSE |
| 4 | 143326395 | SNV | C | G | 1 | 24411 | Hetero | 11 | 65 | 16.9 | 0.23 | 0 | 43 | Yes | INPP4B |
| 4 | 154553853 | SNV | C | T | 1 | 24411 | Hetero | 33 | 88 | 37.5 | 0.38 | 0 | 73 | - | KIAA0922 |
| 4 | 167022918 | SNV | A | C | 1 | 24411 | Hetero | 19 | 151 | 12.6 | 0.48 | 0 | 83 | - | TLL1 |
| 4 | 177116766 | SNV | G | A | 1 | 24411 | Hetero | 22 | 146 | 15.1 | 0.50 | 0 | 66 | - | SPATA4 |
| 4 | 186290893 | SNV | G | A | 1 | 24411 | Hetero | 97 | 174 | 55.7 | 0.19 | 0 | 83 | - | SNX25 |
| 4 | 119632075 | Insertion | - | A | 1 | 24411 | Hetero | 7 | 53 | 13.2 | 0.44 | 0 | 48 | - | METTL14 |
| 4 | 147363404 | Insertion | - | AT | 2 | 24411 | Hetero | 11 | 56 | 19.6 | 0.42 | 0 | 36 | - | SLC10A7 |
| 5 | 23522941 | SNV | G | A | 1 | 24411 | Hetero | 24 | 195 | 12.3 | 0.32 | 0 | 104 | Yes | PRDM9 |
| 5 | 85586685 | SNV | A | C | 1 | 24411 | Hetero | 26 | 89 | 29.2 | 0.38 | 0 | 84 | - | NBPF22P |
| 5 | 123984074 | SNV | T | A | 1 | 24411 | Hetero | 106 | 241 | 44.0 | 0.43 | 0 | 154 | Yes | ZNF608 |
| 5 | 131607038 | SNV | A | C | 1 | 24411 | Hetero | 12 | 115 | 10.4 | 0.08 | 0 | 53 | Yes | P4HA2 |
| 5 | 134236463 | SNV | A | T | 1 | 24411 | Hetero | 10 | 72 | 13.9 | 0.50 | 0 | 32 | - | TXNDC15 |
| 5 | 139941644 | SNV | G | T | 1 | 24411 | Hetero | 203 | 275 | 73.8 | 0.47 | 0 | 147 | - | APBB3 |
| 5 | 141247182 | Deletion | A | - | 1 | 24411 | Hetero | 11 | 97 | 11.3 | 0.45 | 0 | 37 | - | PCDH1 |
| 5 | 149460418 | SNV | G | A | 1 | 24411 | Hetero | 24 | 160 | 15.0 | 0.33 | 0 | 116 | No | CSF1R |
| 5 | 159820285 | SNV | T | C | 1 | 24411 | Hetero | 18 | 105 | 17.1 | 0.36 | 0 | 63 | - | C5orf54 |
| 5 | 131730903 | Insertion | - | T | 1 | 24411 | Hetero | 8 | 54 | 14.8 | 0.33 | 0 | 35 | - | SLC22A5 |
| 5 | 61874619 | Insertion | - | A | 1 | 24411 | Hetero | 9 | 90 | 10.0 | 0.33 | 0 | 68 | - | IPO11 |
| 6 | 350823 | SNV | C | T | 1 | 24411 | Hetero | 116 | 456 | 25.4 | 0.49 | 0 | 288 | No | DUSP22 |
| 6 | 1613386 | SNV | C | T | 1 | 24411 | Hetero | 8 | 76 | 10.5 | 0.44 | 0 | 69 | - | FOXC1 |
| 6 | 9697989 | SNV | C | T | 1 | 24411 | Hetero | 51 | 100 | 51.0 | 0.46 | 0 | 83 | No | OFCC1 |
| 6 | 11580562 | SNV | C | G | 1 | 24411 | Hetero | 22 | 44 | 50.0 | 0.13 | 0 | 33 | - | TMEM170B |
| 6 | 27819844 | SNV | A | G | 1 | 24411 | Hetero | 21 | 92 | 22.8 | 0.35 | 0 | 45 | - | HIST1H2BN |
| 6 | 27861304 | SNV | G | A | 1 | 24411 | Hetero | 94 | 213 | 44.1 | 0.45 | 0 | 176 | Yes | HIST1H2BO |
| 6 | 36334309 | SNV | G | A | 1 | 24411 | Hetero | 7 | 39 | 17.9 | 0.14 | 0 | 37 | - | ETV7 |
| 6 | 39053982 | SNV | T | C | 1 | 24411 | Hetero | 17 | 98 | 17.3 | 0.45 | 0 | 73 | - | GLP1R |
| 6 | 43309847 | SNV | T | A | 1 | 24411 | Hetero | 10 | 91 | 11.0 | 0.46 | 0 | 74 | - | ZNF318 |
| 6 | 49989573 | SNV | C | G | 1 | 24411 | Hetero | 15 | 102 | 14.7 | 0.26 | 0 | 87 | - | DEFB110 |
| 6 | 52869778 | SNV | T | A | 1 | 24411 | Hetero | 25 | 63 | 39.7 | 0.20 | 0 | 41 | - | ICK |
| 6 | 87964755 | SNV | A | G | 1 | 24411 | Hetero | 48 | 155 | 31.0 | 0.38 | 0 | 129 | Yes | ZNF292 |
| 6 | 134494587 | SNV | G | A | 1 | 24411 | Hetero | 15 | 113 | 13.3 | 0.44 | 0 | 77 | - | SGK1 |
| 6 | 30154350 | Insertion | - | A | 1 | 24411 | Hetero | 7 | 69 | 10.1 | 0.29 | 0 | 40 | - | TRIM26 |
| 6 | 32147291 | Insertion | - | T | 1 | 24411 | Hetero | 11 | 88 | 12.5 | 0.31 | 0 | 50 | - | RNF5 |
| 7 | 19156310 | SNV | G | T | 1 | 24411 | Hetero | 10 | 99 | 10.1 | 0.40 | 0 | 122 | - | TWIST1 |
| 7 | 26576120 | SNV | C | T | 1 | 24411 | Hetero | 25 | 53 | 47.2 | 0.43 | 0 | 31 | - | KIAA0087 |
| 7 | 78119036 | SNV | C | G | 1 | 24411 | Hetero | 28 | 94 | 29.8 | 0.20 | 0 | 58 | - | MAGI2 |
| 7 | 92730892 | SNV | C | T | 1 | 24411 | Hetero | 65 | 287 | 22.6 | 0.37 | 0 | 236 | Yes | SAMD9 |
| 7 | 108204885 | SNV | T | G | 1 | 24411 | Hetero | 23 | 204 | 11.3 | 0.46 | 0 | 146 | Yes | PNPLA8 |
| 7 | 127222285 | SNV | C | T | 1 | 24411 | Hetero | 102 | 202 | 50.5 | 0.45 | 0 | 164 | Yes | GCC1 |
| 7 | 134653611 | Deletion | A | - | 1 | 24411 | Hetero | 4 | 39 | 10.3 | 0.25 | 0 | 33 | - | CALD1 |
| 7 | 137798371 | SNV | G | A | 1 | 24411 | Hetero | 26 | 66 | 39.4 | 0.28 | 0 | 53 | - | AKR1D1 |
| 7 | 138444577 | SNV | G | T | 1 | 24411 | Hetero | 40 | 75 | 53.3 | 0.46 | 0 | 78 | No | ATP6V0A4 |
| 7 | 141920125 | SNV | T | A | 1 | 24411 | Hetero | 47 | 347 | 13.5 | 0.47 | 0 | 326 | No | RP11-1220K2.2 |
| 7 | 102883497 | Insertion | - | A | 1 | 24411 | Hetero | 6 | 54 | 11.1 | 0.14 | 0 | 37 | - | DPY19L2P2 |
| 7 | 138519717 | Insertion | - | A | 1 | 24411 | Hetero | 8 | 68 | 11.8 | 0.44 | 0 | 49 | - | TMEM213 |
| 7 | 95442689 | Deletion | TT | - | 2 | 24411 | Hetero | 8 | 66 | 12.1 | 0.20 | 0 | 39 | - | DYNC1I1 |
| 8 | 617700 | SNV | C | T | 1 | 24411 | Hetero | 23 | 136 | 16.9 | 0.28 | 0 | 71 | - | ERICH1 |
| 8 | 3565989 | SNV | G | C | 1 | 24411 | Hetero | 29 | 278 | 10.4 | 0.50 | 0 | 144 | Yes | CSMD1 |
| 8 | 8999162 | SNV | G | T | 1 | 24411 | Hetero | 39 | 307 | 12.7 | 0.50 | 0 | 139 | - | PPP1R3B |
| 8 | 10582982 | SNV | G | C | 1 | 24411 | Hetero | 45 | 129 | 34.9 | 0.36 | 0 | 76 | - | SOX7 |
| 8 | 35653795 | SNV | G | A | 1 | 24411 | Hetero | 28 | 66 | 42.4 | 0.29 | 0 | 42 | - | UNC5D |
| 8 | 48973640 | SNV | A | G | 1 | 24411 | Hetero | 53 | 132 | 40.2 | 0.44 | 0 | 70 | - | UBE2V2 |
| 8 | 56437358 | SNV | A | T | 1 | 24411 | Hetero | 15 | 64 | 23.4 | 0.47 | 0 | 37 | - | XKR4 |
| 8 | 86376176 | SNV | G | T | 1 | 24411 | Hetero | 75 | 205 | 36.6 | 0.36 | 0 | 106 | - | CA2 |
| 8 | 96166227 | SNV | A | G | 1 | 24411 | Hetero | 44 | 239 | 18.4 | 0.09 | 0 | 82 | - | PLEKHF2 |
| 8 | 102681711 | SNV | A | G | 1 | 24411 | Hetero | 68 | 132 | 51.5 | 0.49 | 0 | 70 | - | GRHL2 |
| 8 | 105360898 | SNV | G | C | 1 | 24411 | Hetero | 50 | 134 | 37.3 | 0.37 | 0 | 92 | Yes | DCSTAMP |
| 8 | 107670229 | SNV | G | A | 1 | 24411 | Hetero | 57 | 415 | 13.7 | 0.42 | 0 | 240 | No | OXR1 |
| 8 | 109796676 | SNV | C | A | 1 | 24411 | Hetero | 36 | 242 | 14.9 | 0.43 | 0 | 92 | Yes | TMEM74 |
| 8 | 139209841 | SNV | C | G | 1 | 24411 | Hetero | 48 | 251 | 19.1 | 0.48 | 0 | 129 | Yes | FAM135B |
| 8 | 127567087 | Insertion | - | A | 1 | 24411 | Hetero | 8 | 67 | 11.9 | 0.22 | 0 | 51 | - | FAM84B |
| 8 | 56923131 | Insertion | - | A | 1 | 24411 | Hetero | 20 | 127 | 15.7 | 0.43 | 0 | 48 | - | LYN |
| 9 | 8521425 | SNV | C | G | 1 | 24411 | Hetero | 84 | 100 | 84.0 | 0.41 | 0 | 165 | Yes | PTPRD |
| 9 | 24545101 | SNV | C | T | 1 | 24411 | Hetero | 11 | 94 | 11.7 | 0.45 | 0 | 109 | - | IZUMO3 |
| 9 | 37429002 | SNV | G | A | 1 | 24411 | Homo | 51 | 56 | 91.1 | 0.27 | 0 | 62 | - | GRHPR |
| 9 | 74970471 | SNV | C | G | 1 | 24411 | Hetero | 6 | 50 | 12.0 | 0.38 | 0 | 47 | - | ZFAND5 |
| 9 | 91727490 | SNV | T | C | 1 | 24411 | Hetero | 19 | 77 | 24.7 | 0.35 | 0 | 91 | Yes | SHC3 |
| 9 | 96051550 | SNV | G | A | 1 | 24411 | Hetero | 8 | 63 | 12.7 | 0.50 | 0 | 77 | Yes | WNK2 |
| 9 | 99700838 | SNV | C | T | 1 | 24411 | Hetero | 47 | 83 | 56.6 | 0.45 | 0 | 96 | Yes | RP11-330M2.7 |
| 9 | 111693439 | SNV | C | T | 1 | 24411 | Hetero | 10 | 89 | 11.2 | 0.36 | 0 | 101 | - | IKBKAP |
| 9 | 123628427 | SNV | A | G | 1 | 24411 | Hetero | 14 | 33 | 42.4 | 0.50 | 0 | 39 | - | PHF19 |
| 9 | 127215053 | SNV | C | A | 1 | 24411 | Hetero | 9 | 32 | 28.1 | 0.18 | 0 | 39 | - | GPR144 |
| 9 | 128128754 | SNV | A | G | 1 | 24411 | Hetero | 49 | 64 | 76.6 | 0.48 | 0 | 55 | - | GAPVD1 |
| 9 | 138899408 | SNV | G | T | 1 | 24411 | Hetero | 6 | 41 | 14.6 | 0.33 | 0 | 42 | - | NACC2 |
| 10 | 24918569 | Deletion | A | - | 1 | 24411 | Hetero | 4 | 39 | 10.3 | 0.25 | 0 | 39 | - | ARHGAP21 |
| 10 | 63422763 | SNV | C | T | 1 | 24411 | Hetero | 37 | 161 | 23.0 | 0.35 | 0 | 128 | Yes | C10orf107 |
| 10 | 97424088 | SNV | G | A | 1 | 24411 | Hetero | 39 | 126 | 31.0 | 0.33 | 0 | 75 | - | TCTN3 |
| 10 | 115534592 | Deletion | AA | - | 2 | 24411 | Hetero | 7 | 62 | 11.3 | 0.44 | 0 | 40 | Deletion | PLEKHS1 |
| 11 | 19076965 | SNV | G | C | 1 | 24411 | Hetero | 28 | 242 | 11.6 | 0.49 | 0 | 230 | Yes | MRGPRX2 |
| 11 | 45833966 | SNV | C | T | 1 | 24411 | Hetero | 16 | 82 | 19.5 | 0.44 | 0 | 62 | - | SLC35C1 |
| 11 | 47189479 | SNV | T | C | 1 | 24411 | Hetero | 34 | 163 | 20.9 | 0.33 | 0 | 111 | Yes | ARFGAP2 |
| 11 | 64065133 | SNV | C | T | 1 | 24411 | Hetero | 30 | 115 | 26.1 | 0.33 | 0 | 93 | - | KCNK4 |
| 11 | 102586005 | SNV | T | G | 1 | 24411 | Homo | 1876 | 1958 | 95.8 | 0.45 | 0 | 115 | - | MMP8 |
| 11 | 102987331 | SNV | A | C | 1 | 24411 | Homo | 1326 | 1392 | 95.3 | 0.29 | 0 | 70 | No | DYNC2H1 |
| 11 | 114315007 | SNV | A | G | 1 | 24411 | Hetero | 74 | 271 | 27.3 | 0.48 | 0 | 264 | - | REXO2 |
| 11 | 121447778 | SNV | G | A | 1 | 24411 | Hetero | 17 | 101 | 16.8 | 0.26 | 0 | 98 | - | SORL1 |
| 12 | 31116908 | SNV | G | A | 1 | 24411 | Hetero | 49 | 264 | 18.6 | 0.38 | 0 | 189 | Yes | TSPAN11 |
| 12 | 40917226 | SNV | G | T | 1 | 24411 | Hetero | 20 | 107 | 18.7 | 0.25 | 0 | 76 | - | MUC19 |
| 12 | 70216130 | SNV | C | T | 1 | 24411 | Hetero | 19 | 60 | 31.7 | 0.45 | 0 | 45 | - | RAB3IP |
| 12 | 109372462 | SNV | G | A | 1 | 24411 | Hetero | 21 | 81 | 25.9 | 0.32 | 0 | 69 | - | SVOP |
| 12 | 109994850 | SNV | C | T | 1 | 24411 | Hetero | 24 | 76 | 31.6 | 0.44 | 0 | 67 | Yes | MMAB |
| 12 | 117494751 | SNV | T | C | 1 | 24411 | Hetero | 118 | 215 | 54.9 | 0.29 | 0 | 127 | - | TESC |
| 12 | 117968869 | SNV | C | A | 1 | 24411 | Hetero | 30 | 212 | 14.2 | 0.43 | 0 | 189 | - | KSR2 |
| 12 | 76441881 | Insertion | - | A | 1 | 24411 | Hetero | 7 | 67 | 10.4 | 0.13 | 0 | 54 | - | NAP1L1 |
| 13 | 52548998 | SNV | C | A | 1 | 24411 | Hetero | 83 | 323 | 25.7 | 0.44 | 0 | 220 | Yes | ATP7B |
| 14 | 31592821 | SNV | T | C | 1 | 24411 | Hetero | 24 | 55 | 43.6 | 0.19 | 0 | 42 | Yes | HECTD1 |
| 14 | 45478301 | SNV | A | G | 1 | 24411 | Hetero | 27 | 136 | 19.9 | 0.20 | 0 | 103 | No | KLHL28 |
| 14 | 53098941 | SNV | G | A | 1 | 24411 | Hetero | 43 | 91 | 47.3 | 0.43 | 0 | 49 | Yes | GPR137C |
| 14 | 55257764 | SNV | G | A | 1 | 24411 | Hetero | 11 | 70 | 15.7 | 0.27 | 0 | 50 | - | SAMD4A |
| 14 | 55890915 | SNV | A | G | 1 | 24411 | Hetero | 18 | 78 | 23.1 | 0.46 | 0 | 48 | Yes | TBPL2 |
| 14 | 56766776 | SNV | C | A | 1 | 24411 | Hetero | 20 | 86 | 23.3 | 0.33 | 0 | 59 | - | PELI2 |
| 14 | 57735648 | SNV | A | T | 1 | 24411 | Hetero | 14 | 53 | 26.4 | 0.38 | 0 | 39 | - | EXOC5 |
| 14 | 60978741 | SNV | C | G | 1 | 24411 | Hetero | 8 | 54 | 14.8 | 0.25 | 0 | 31 | - | SIX6 |
| 14 | 103420538 | SNV | C | T | 1 | 24411 | Hetero | 13 | 101 | 12.9 | 0.31 | 0 | 64 | No | CDC42BPB |
| 15 | 39888066 | SNV | G | A | 1 | 24411 | Hetero | 100 | 395 | 25.3 | 0.44 | 0 | 418 | - | THBS1 |
| 15 | 70349852 | SNV | G | A | 1 | 24411 | Hetero | 55 | 141 | 39.0 | 0.45 | 0 | 96 | No | TLE3 |
| 15 | 85383893 | SNV | C | T | 1 | 24411 | Hetero | 25 | 200 | 12.5 | 0.42 | 0 | 154 | No | ALPK3 |
| 15 | 86809684 | SNV | C | A | 1 | 24411 | Hetero | 20 | 57 | 35.1 | 0.14 | 0 | 34 | Yes | AGBL1 |
| 16 | 1725307 | SNV | C | T | 1 | 24411 | Hetero | 247 | 408 | 60.5 | 0.47 | 0 | 162 | - | CRAMP1L |
| 16 | 21695822 | SNV | G | T | 1 | 24411 | Hetero | 63 | 231 | 27.3 | 0.47 | 0 | 129 | - | OTOA |
| 16 | 25182041 | SNV | C | T | 1 | 24411 | Hetero | 21 | 182 | 11.5 | 0.16 | 0 | 58 | Yes | LCMT1 |
| 16 | 28936207 | Deletion | T | - | 1 | 24411 | Hetero | 64 | 387 | 16.5 | 0.35 | 0 | 173 | - | RABEP2 |
| 16 | 46695716 | SNV | G | A | 1 | 24411 | Hetero | 26 | 213 | 12.2 | 0.38 | 0 | 108 | Yes | VPS35 |
| 16 | 57077414 | SNV | C | T | 1 | 24411 | Hetero | 70 | 514 | 13.6 | 0.39 | 0 | 236 | - | NLRC5 |
| 16 | 75033671 | SNV | C | T | 1 | 24411 | Hetero | 18 | 131 | 13.7 | 0.32 | 0 | 58 | No | WDR59 |
| 16 | 2868885 | Insertion | - | A | 1 | 24411 | Hetero | 47 | 332 | 14.2 | 0.40 | 0 | 123 | Insertion | PRSS21 |
| 17 | 4462017 | SNV | G | A | 1 | 24411 | Hetero | 50 | 176 | 28.4 | 0.42 | 0 | 101 | Yes | GGT6 |
| 17 | 4788798 | SNV | C | T | 1 | 24411 | Hetero | 27 | 75 | 36.0 | 0.44 | 0 | 55 | Yes | MINK1 |
| 17 | 7578281 | SNV | G | C | 1 | 24411 | Hetero | 88 | 193 | 45.6 | 0.44 | 0 | 124 | Yes | TP53 |
| 17 | 18145830 | SNV | G | T | 1 | 24411 | Hetero | 23 | 190 | 12.1 | 0.38 | 0 | 86 | Yes | LLGL1 |
| 17 | 21201721 | SNV | C | G | 1 | 24411 | Hetero | 76 | 753 | 10.1 | 0.47 | 0 | 585 | - | MAP2K3 |
| 17 | 33288445 | SNV | G | A | 1 | 24411 | Hetero | 31 | 88 | 35.2 | 0.42 | 0 | 77 | - | CCT6B |
| 17 | 38279639 | SNV | G | A | 1 | 24411 | Hetero | 13 | 128 | 10.2 | 0.20 | 0 | 75 | - | MSL1 |
| 17 | 40698892 | SNV | G | A | 1 | 24411 | Hetero | 15 | 74 | 20.3 | 0.13 | 0 | 50 | - | HSD17B1P1 |
| 17 | 61666469 | SNV | G | T | 1 | 24411 | Hetero | 29 | 117 | 24.8 | 0.39 | 0 | 66 | Yes | DCAF7 |
| 17 | 78175496 | SNV | T | C | 1 | 24411 | Hetero | 47 | 110 | 42.7 | 0.37 | 0 | 70 | - | CARD14 |
| 17 | 79006814 | SNV | C | A | 1 | 24411 | Hetero | 147 | 292 | 50.3 | 0.46 | 0 | 150 | - | BAIAP2-AS1 |
| 17 | 79390345 | SNV | G | A | 1 | 24411 | Hetero | 7 | 63 | 11.1 | 0.38 | 0 | 30 | - | BAHCC1 |
| 18 | 29672683 | SNV | C | T | 1 | 24411 | Hetero | 47 | 221 | 21.3 | 0.20 | 0 | 176 | - | RNF138 |
| 18 | 54305749 | SNV | G | A | 1 | 24411 | Hetero | 38 | 91 | 41.8 | 0.36 | 0 | 75 | - | TXNL1 |
| 18 | 63489373 | SNV | C | G | 1 | 24411 | Hetero | 40 | 124 | 32.3 | 0.45 | 0 | 92 | Yes | CDH7 |
| 19 | 2236224 | SNV | C | T | 1 | 24411 | Hetero | 76 | 103 | 73.8 | 0.44 | 0 | 52 | No | PLEKHJ1 |
| 19 | 33791299 | SNV | C | T | 1 | 24411 | Hetero | 29 | 161 | 18.0 | 0.44 | 0 | 101 | - | CTD-2540B15.11 |
| 19 | 35790707 | SNV | C | A | 1 | 24411 | Hetero | 56 | 117 | 47.9 | 0.21 | 0 | 62 | No | MAG |
| 19 | 41596098 | SNV | C | A | 1 | 24411 | Hetero | 35 | 128 | 27.3 | 0.30 | 0 | 82 | Yes | CYP2A13 |
| 19 | 41798282 | SNV | G | T | 1 | 24411 | Hetero | 53 | 152 | 34.9 | 0.41 | 0 | 110 | Yes | HNRNPUL1 |
| 19 | 53644731 | SNV | A | T | 1 | 24411 | Hetero | 10 | 80 | 12.5 | 0.38 | 0 | 63 | No | ZNF347 |
| 19 | 55146707 | SNV | C | G | 1 | 24411 | Hetero | 58 | 135 | 43.0 | 0.30 | 0 | 85 | - | LILRB1 |
| 19 | 57133466 | SNV | G | C | 1 | 24411 | Hetero | 142 | 336 | 42.3 | 0.42 | 0 | 226 | Yes | ZNF71 |
| 19 | 57985801 | SNV | G | A | 1 | 24411 | Hetero | 71 | 147 | 48.3 | 0.25 | 0 | 79 | - | ZNF772 |
| 20 | 19867392 | SNV | G | A | 1 | 24411 | Hetero | 52 | 498 | 10.4 | 0.37 | 0 | 255 | Yes | RIN2 |
| 20 | 34220602 | SNV | C | T | 1 | 24411 | Hetero | 52 | 373 | 13.9 | 0.33 | 0 | 114 | Yes | CPNE1 |
| 20 | 40040953 | SNV | T | C | 1 | 24411 | Hetero | 31 | 183 | 16.9 | 0.28 | 0 | 97 | - | CHD6 |
| 20 | 48481250 | SNV | C | T | 1 | 24411 | Hetero | 45 | 363 | 12.4 | 0.40 | 0 | 129 | - | SLC9A8 |
| 20 | 49574938 | SNV | C | A | 1 | 24411 | Hetero | 58 | 549 | 10.6 | 0.35 | 0 | 190 | No | DPM1 |
| 20 | 54945359 | SNV | G | A | 1 | 24411 | Hetero | 254 | 371 | 68.5 | 0.34 | 0 | 149 | Yes | AURKA |
| 20 | 57022600 | SNV | C | G | 1 | 24411 | Hetero | 23 | 106 | 21.7 | 0.31 | 0 | 37 | - | VAPB |
| 20 | 2633402 | Insertion | - | GG | 2 | 24411 | Hetero | 29 | 79 | 36.7 | 0.19 | 0 | 37 | - | NOP56 |
| 20 | 31023498 | Insertion | - | A | 1 | 24411 | Hetero | 181 | 250 | 72.4 | 0.46 | 0 | 82 | Insertion | ASXL1 |
| 21 | 34930252 | SNV | G | A | 1 | 24411 | Hetero | 7 | 57 | 12.3 | 0.22 | 0 | 37 | - | SON |
| 21 | 35263264 | SNV | G | C | 1 | 24411 | Hetero | 19 | 149 | 12.8 | 0.46 | 0 | 132 | - | AP000304.12 |
| 21 | 43187184 | SNV | C | G | 1 | 24411 | Hetero | 36 | 96 | 37.5 | 0.31 | 0 | 101 | No | RIPK4 |
| 21 | 38564530 | Insertion | - | T | 1 | 24411 | Hetero | 7 | 64 | 10.9 | 0.13 | 0 | 46 | - | TTC3 |
| 22 | 22869285 | SNV | C | T | 1 | 24411 | Hetero | 104 | 205 | 50.7 | 0.46 | 0 | 157 | Yes | ZNF280A |
| 22 | 36537815 | SNV | C | T | 1 | 24411 | Hetero | 49 | 235 | 20.9 | 0.35 | 0 | 150 | No | APOL3 |
| 22 | 46776711 | SNV | G | A | 1 | 24411 | Hetero | 24 | 162 | 14.8 | 0.43 | 0 | 99 | No | CELSR1 |
| X | 65819278 | SNV | C | T | 1 | 24411 | Hetero | 23 | 62 | 37.1 | 0.31 | 0 | 35 | - | EDA2R |
| X | 90691033 | SNV | G | C | 1 | 24411 | Hetero | 17 | 165 | 10.3 | 0.41 | 0 | 120 | Yes | PABPC5 |
| X | 133930096 | SNV | A | G | 1 | 24411 | Hetero | 72 | 183 | 39.3 | 0.37 | 0 | 115 | - | FAM122B |
| X | 13731222 | Insertion | - | A | 1 | 24411 | Hetero | 5 | 49 | 10.2 | 0.20 | 0 | 30 | - | TRAPPC2 |
| 1 | 1226769 | SNV | C | T | 1 | 25074 | Hetero | 42 | 76 | 55.3 | 0.27 | 0 | 41 | Yes | SCNN1D |
| 1 | 1269538 | SNV | G | A | 1 | 25074 | Hetero | 125 | 203 | 61.6 | 0.20 | 0 | 114 | No | TAS1R3 |
| 1 | 1459610 | SNV | G | A | 1 | 25074 | Hetero | 22 | 172 | 12.8 | 0.31 | 0 | 121 | - | ATAD3A |
| 1 | 1599940 | SNV | C | T | 1 | 25074 | Hetero | 24 | 67 | 35.8 | 0.15 | 0 | 42 | - | SLC35E2B |
| 1 | 1683420 | SNV | A | T | 1 | 25074 | Hetero | 29 | 44 | 65.9 | 0.14 | 0 | 178 | - | NADK |
| 1 | 1683794 | SNV | G | A | 1 | 25074 | Hetero | 64 | 143 | 44.8 | 0.46 | 0 | 84 | - | NADK |
| 1 | 3352514 | SNV | G | A | 1 | 25074 | Hetero | 224 | 362 | 61.9 | 0.48 | 0 | 243 | - | PRDM16 |
| 1 | 3546467 | SNV | C | T | 1 | 25074 | Hetero | 81 | 142 | 57.0 | 0.45 | 0 | 82 | - | TPRG1L |
| 1 | 9162051 | SNV | C | A | 1 | 25074 | Hetero | 60 | 96 | 62.5 | 0.48 | 0 | 49 | - | GPR157 |
| 1 | 12364769 | SNV | A | G | 1 | 25074 | Hetero | 22 | 62 | 35.5 | 0.21 | 0 | 38 | No | VPS13D |
| 1 | 13447650 | SNV | C | T | 1 | 25074 | Hetero | 31 | 136 | 22.8 | 0.48 | 0 | 107 | - | PRAMEF13 |
| 1 | 16482605 | SNV | G | A | 1 | 25074 | Hetero | 150 | 248 | 60.5 | 0.32 | 0 | 122 | - | RP11-276H7.2 |
| 1 | 16973072 | SNV | T | C | 1 | 25074 | Hetero | 24 | 168 | 14.3 | 0.33 | 0 | 127 | - | MST1P2 |
| 1 | 17033741 | SNV | G | A | 1 | 25074 | Hetero | 19 | 100 | 19.0 | 0.32 | 0 | 69 | - | RP1-163M9.6 |
| 1 | 17081710 | SNV | G | C | 1 | 25074 | Hetero | 56 | 511 | 11.0 | 0.37 | 0 | 360 | - | MST1P9 |
| 1 | 17081815 | SNV | C | T | 1 | 25074 | Hetero | 30 | 227 | 13.2 | 0.16 | 0 | 208 | - | MST1P9 |
| 1 | 17087038 | SNV | G | T | 1 | 25074 | Hetero | 46 | 410 | 11.2 | 0.38 | 0 | 417 | - | MST1P9 |
| 1 | 17087377 | SNV | A | C | 1 | 25074 | Hetero | 49 | 468 | 10.5 | 0.40 | 0 | 296 | - | MST1P9 |
| 1 | 17380345 | SNV | C | T | 1 | 25074 | Hetero | 97 | 151 | 64.2 | 0.20 | 0 | 79 | - | SDHB |
| 1 | 19484446 | SNV | T | C | 1 | 25074 | Hetero | 158 | 261 | 60.5 | 0.43 | 0 | 147 | Yes | UBR4 |
| 1 | 19609437 | SNV | T | C | 1 | 25074 | Hetero | 15 | 94 | 16.0 | 0.47 | 0 | 99 | - | AKR7A3 |
| 1 | 20637260 | SNV | G | A | 1 | 25074 | Hetero | 125 | 313 | 39.9 | 0.44 | 0 | 201 | - | VWA5B1 |
| 1 | 21546459 | SNV | C | T | 1 | 25074 | Hetero | 54 | 168 | 32.1 | 0.35 | 0 | 114 | Yes | ECE1 |
| 1 | 24799399 | Deletion | A | - | 1 | 25074 | Hetero | 8 | 72 | 11.1 | 0.22 | 0 | 46 | - | NIPAL3 |
| 1 | 26110849 | SNV | G | A | 1 | 25074 | Hetero | 36 | 99 | 36.4 | 0.32 | 0 | 61 | - | MAN1C1 |
| 1 | 29043402 | SNV | T | C | 1 | 25074 | Hetero | 25 | 76 | 32.9 | 0.38 | 0 | 49 | - | GMEB1 |
| 1 | 29438902 | SNV | A | G | 1 | 25074 | Hetero | 64 | 167 | 38.3 | 0.19 | 0 | 95 | Yes | EPB41 |
| 1 | 29445948 | SNV | G | A | 1 | 25074 | Hetero | 53 | 143 | 37.1 | 0.27 | 0 | 89 | - | EPB41 |
| 1 | 31342977 | SNV | G | A | 1 | 25074 | Hetero | 67 | 188 | 35.6 | 0.46 | 0 | 108 | - | SDC3 |
| 1 | 31845917 | SNV | C | T | 1 | 25074 | Hetero | 71 | 168 | 42.3 | 0.18 | 0 | 98 | - | FABP3 |
| 1 | 32832755 | SNV | A | G | 1 | 25074 | Hetero | 70 | 128 | 54.7 | 0.44 | 0 | 74 | - | BSDC1 |
| 1 | 34064891 | SNV | C | A | 1 | 25074 | Hetero | 46 | 76 | 60.5 | 0.45 | 0 | 56 | - | CSMD2 |
| 1 | 36387279 | SNV | C | T | 1 | 25074 | Hetero | 103 | 194 | 53.1 | 0.38 | 0 | 118 | - | EIF2C1 |
| 1 | 36521935 | SNV | C | T | 1 | 25074 | Hetero | 20 | 71 | 28.2 | 0.27 | 0 | 40 | - | EIF2C3 |
| 1 | 38280175 | Deletion | C | - | 1 | 25074 | Hetero | 74 | 265 | 27.9 | 0.48 | 0 | 186 | - | MTF1 |
| 1 | 39981462 | SNV | G | T | 1 | 25074 | Hetero | 27 | 149 | 18.1 | 0.14 | 0 | 76 | - | BMP8A |
| 1 | 40235277 | SNV | A | G | 1 | 25074 | Hetero | 39 | 71 | 54.9 | 0.45 | 0 | 65 | - | BMP8B |
| 1 | 41236838 | Deletion | T | - | 1 | 25074 | Hetero | 85 | 146 | 58.2 | 0.25 | 0 | 101 | - | NFYC |
| 1 | 41289836 | SNV | C | T | 1 | 25074 | Hetero | 56 | 93 | 60.2 | 0.38 | 0 | 49 | Yes | KCNQ4 |
| 1 | 43895725 | SNV | C | T | 1 | 25074 | Hetero | 47 | 144 | 32.6 | 0.47 | 0 | 87 | Yes | SZT2 |
| 1 | 44462024 | SNV | G | A | 1 | 25074 | Hetero | 21 | 49 | 42.9 | 0.45 | 0 | 48 | - | CCDC24 |
| 1 | 48763656 | SNV | T | C | 1 | 25074 | Hetero | 42 | 76 | 55.3 | 0.42 | 0 | 36 | - | SPATA6 |
| 1 | 54606886 | SNV | G | A | 1 | 25074 | Hetero | 146 | 226 | 64.6 | 0.38 | 0 | 99 | No | CDCP2 |
| 1 | 57176418 | SNV | A | T | 1 | 25074 | Hetero | 23 | 61 | 37.7 | 0.48 | 0 | 59 | - | PRKAA2 |
| 1 | 57185863 | SNV | C | T | 1 | 25074 | Hetero | 36 | 62 | 58.1 | 0.40 | 0 | 40 | Yes | C1orf168 |
| 1 | 61922501 | SNV | A | C | 1 | 25074 | Hetero | 30 | 79 | 38.0 | 0.16 | 0 | 56 | - | NFIA |
| 1 | 63870204 | SNV | G | A | 1 | 25074 | Hetero | 43 | 101 | 42.6 | 0.20 | 0 | 74 | Yes | ALG6 |
| 1 | 68895518 | SNV | G | A | 1 | 25074 | Hetero | 141 | 276 | 51.1 | 0.42 | 0 | 297 | Yes | RPE65 |
| 1 | 77763552 | SNV | A | T | 1 | 25074 | Hetero | 9 | 38 | 23.7 | 0.27 | 0 | 37 | Yes | AK5 |
| 1 | 77779452 | SNV | C | A | 1 | 25074 | Hetero | 26 | 69 | 37.7 | 0.13 | 0 | 66 | - | AK5 |
| 1 | 78046661 | SNV | A | G | 1 | 25074 | Hetero | 30 | 78 | 38.5 | 0.36 | 0 | 56 | - | ZZZ3 |
| 1 | 93342058 | SNV | C | A | 1 | 25074 | Hetero | 50 | 110 | 45.5 | 0.36 | 0 | 106 | - | FAM69A |
| 1 | 95362959 | Deletion | T | - | 1 | 25074 | Hetero | 17 | 41 | 41.5 | 0.35 | 0 | 40 | - | CNN3 |
| 1 | 98186009 | SNV | T | C | 1 | 25074 | Hetero | 38 | 68 | 55.9 | 0.36 | 0 | 62 | - | DPYD |
| 1 | 100436124 | SNV | T | C | 1 | 25074 | Hetero | 31 | 68 | 45.6 | 0.09 | 0 | 58 | Yes | SLC35A3 |
| 1 | 109193024 | SNV | G | A | 1 | 25074 | Hetero | 26 | 62 | 41.9 | 0.14 | 0 | 61 | - | HENMT1 |
| 1 | 109808484 | SNV | G | A | 1 | 25074 | Hetero | 101 | 204 | 49.5 | 0.26 | 0 | 157 | Yes | CELSR2 |
| 1 | 110039780 | SNV | G | C | 1 | 25074 | Hetero | 79 | 138 | 57.2 | 0.48 | 0 | 125 | - | CYB561D1 |
| 1 | 110049513 | SNV | C | G | 1 | 25074 | Hetero | 46 | 86 | 53.5 | 0.23 | 0 | 76 | - | AMIGO1 |
| 1 | 110881989 | SNV | T | C | 1 | 25074 | Hetero | 150 | 318 | 47.2 | 0.40 | 0 | 256 | - | RBM15 |
| 1 | 117664100 | SNV | C | T | 1 | 25074 | Hetero | 94 | 198 | 47.5 | 0.25 | 0 | 165 | - | TRIM45 |
| 1 | 120536204 | SNV | A | G | 1 | 25074 | Hetero | 40 | 227 | 17.6 | 0.38 | 0 | 619 | - | NOTCH2 |
| 1 | 120536329 | SNV | G | A | 1 | 25074 | Hetero | 151 | 565 | 26.7 | 0.43 | 0 | 611 | - | NOTCH2 |
| 1 | 120536821 | SNV | T | A | 1 | 25074 | Hetero | 115 | 409 | 28.1 | 0.34 | 0 | 648 | - | NOTCH2 |
| 1 | 120612084 | SNV | G | A | 1 | 25074 | Hetero | 12 | 57 | 21.1 | 0.43 | 0 | 98 | - | NOTCH2 |
| 1 | 144918954 | SNV | A | G | 1 | 25074 | Hetero | 53 | 241 | 22.0 | 0.43 | 0 | 152 | Yes | PDE4DIP |
| 1 | 145014096 | SNV | G | A | 1 | 25074 | Hetero | 22 | 111 | 19.8 | 0.38 | 0 | 131 | Yes | PDE4DIP |
| 1 | 145014325 | Deletion | C | - | 1 | 25074 | Hetero | 44 | 358 | 12.3 | 0.23 | 0 | 307 | - | PDE4DIP |
| 1 | 145285401 | SNV | T | C | 1 | 25074 | Hetero | 99 | 644 | 15.4 | 0.30 | 0 | 636 | - | NOTCH2NL |
| 1 | 145578545 | SNV | T | C | 1 | 25074 | Hetero | 118 | 195 | 60.5 | 0.40 | 0 | 138 | - | PIAS3 |
| 1 | 148902223 | SNV | G | A | 1 | 25074 | Hetero | 26 | 115 | 22.6 | 0.21 | 0 | 125 | - | DRD5P2 |
| 1 | 148902298 | SNV | G | A | 1 | 25074 | Hetero | 20 | 144 | 13.9 | 0.41 | 0 | 147 | - | DRD5P2 |
| 1 | 149761680 | SNV | G | C | 1 | 25074 | Hetero | 61 | 107 | 57.0 | 0.45 | 0 | 74 | No | FCGR1A |
| 1 | 151237590 | Deletion | C | - | 1 | 25074 | Hetero | 20 | 33 | 60.6 | 0.38 | 0 | 30 | - | PSMD4 |
| 1 | 151687079 | SNV | C | T | 1 | 25074 | Hetero | 54 | 88 | 61.4 | 0.35 | 0 | 73 | No | CELF3 |
| 1 | 152325375 | SNV | T | C | 1 | 25074 | Hetero | 133 | 278 | 47.8 | 0.49 | 0 | 256 | No | FLG2 |
| 1 | 153633768 | SNV | A | G | 1 | 25074 | Hetero | 99 | 259 | 38.2 | 0.48 | 0 | 187 | No | SNAPIN |
| 1 | 155582969 | SNV | C | T | 1 | 25074 | Hetero | 18 | 67 | 26.9 | 0.42 | 0 | 35 | Yes | MSTO1 |
| 1 | 156047067 | SNV | G | A | 1 | 25074 | Hetero | 99 | 449 | 22.0 | 0.42 | 0 | 253 | No | MEX3A |
| 1 | 159159604 | SNV | A | T | 1 | 25074 | Hetero | 49 | 85 | 57.6 | 0.28 | 0 | 66 | No | CADM3 |
| 1 | 159850424 | SNV | T | A | 1 | 25074 | Hetero | 55 | 132 | 41.7 | 0.39 | 0 | 74 | Yes | CCDC19 |
| 1 | 160771739 | SNV | T | C | 1 | 25074 | Hetero | 18 | 95 | 18.9 | 0.32 | 0 | 87 | - | LY9 |
| 1 | 161197814 | SNV | C | T | 1 | 25074 | Hetero | 45 | 121 | 37.2 | 0.32 | 0 | 79 | - | TOMM40L |
| 1 | 161931916 | Deletion | T | - | 1 | 25074 | Hetero | 58 | 102 | 56.9 | 0.41 | 0 | 66 | - | ATF6 |
| 1 | 168169847 | Deletion | A | - | 1 | 25074 | Homo | 68 | 69 | 98.6 | 0.48 | 0 | 38 | - | TIPRL |
| 1 | 173446446 | SNV | T | A | 1 | 25074 | Hetero | 23 | 64 | 35.9 | 0.12 | 0 | 44 | - | PRDX6 |
| 1 | 180257646 | SNV | G | A | 1 | 25074 | Hetero | 27 | 45 | 60.0 | 0.27 | 0 | 41 | Yes | ACBD6 |
| 1 | 180859025 | SNV | G | T | 1 | 25074 | Hetero | 35 | 107 | 32.7 | 0.48 | 0 | 95 | - | XPR1 |
| 1 | 183907332 | Deletion | T | - | 1 | 25074 | Hetero | 61 | 94 | 64.9 | 0.32 | 0 | 59 | - | GLT25D2 |
| 1 | 184661463 | SNV | G | A | 1 | 25074 | Hetero | 66 | 126 | 52.4 | 0.41 | 0 | 88 | - | EDEM3 |
| 1 | 197408412 | SNV | A | G | 1 | 25074 | Hetero | 47 | 68 | 69.1 | 0.37 | 0 | 69 | - | CRB1 |
| 1 | 197480875 | SNV | T | C | 1 | 25074 | Hetero | 53 | 78 | 67.9 | 0.24 | 0 | 65 | Yes | DENND1B |
| 1 | 197641192 | SNV | C | T | 1 | 25074 | Hetero | 50 | 84 | 59.5 | 0.33 | 0 | 67 | Yes | DENND1B |
| 1 | 201781025 | SNV | C | A | 1 | 25074 | Hetero | 75 | 203 | 36.9 | 0.38 | 0 | 157 | - | NAV1 |
| 1 | 201845792 | SNV | A | G | 1 | 25074 | Hetero | 58 | 89 | 65.2 | 0.35 | 0 | 75 | - | IPO9 |
| 1 | 202156133 | SNV | C | G | 1 | 25074 | Hetero | 7 | 61 | 11.5 | 0.29 | 0 | 32 | - | PTPRVP |
| 1 | 203767762 | SNV | C | G | 1 | 25074 | Hetero | 129 | 206 | 62.6 | 0.45 | 0 | 173 | Yes | ZC3H11A |
| 1 | 204218341 | SNV | G | A | 1 | 25074 | Hetero | 33 | 191 | 17.3 | 0.38 | 0 | 134 | Yes | PLEKHA6 |
| 1 | 204590748 | SNV | T | C | 1 | 25074 | Hetero | 82 | 133 | 61.7 | 0.42 | 0 | 103 | - | LRRN2 |
| 1 | 207224326 | SNV | C | A | 1 | 25074 | Hetero | 68 | 168 | 40.5 | 0.41 | 0 | 126 | Yes | YOD1 |
| 1 | 210194570 | SNV | G | A | 1 | 25074 | Hetero | 70 | 123 | 56.9 | 0.17 | 0 | 95 | Yes | SYT14 |
| 1 | 214531079 | Deletion | A | - | 1 | 25074 | Homo | 47 | 47 | 100.0 | 0.37 | 0 | 37 | - | PTPN14 |
| 1 | 221053554 | SNV | T | C | 1 | 25074 | Hetero | 45 | 122 | 36.9 | 0.44 | 0 | 89 | Yes | HLX |
| 1 | 224196666 | SNV | G | C | 1 | 25074 | Hetero | 9 | 65 | 13.8 | 0.10 | 0 | 37 | - | RP11-504P24.4 |
| 1 | 225268427 | SNV | A | T | 1 | 25074 | Hetero | 47 | 77 | 61.0 | 0.06 | 0 | 82 | Yes | DNAH14 |
| 1 | 227919862 | Deletion | G | - | 1 | 25074 | Hetero | 55 | 158 | 34.8 | 0.35 | 0 | 121 | - | SNAP47 |
| 1 | 228003828 | SNV | C | T | 1 | 25074 | Hetero | 55 | 103 | 53.4 | 0.26 | 0 | 89 | No | PRSS38 |
| 1 | 228528435 | SNV | G | A | 1 | 25074 | Hetero | 72 | 203 | 35.5 | 0.44 | 0 | 109 | Yes | OBSCN |
| 1 | 229406845 | SNV | C | G | 1 | 25074 | Hetero | 31 | 97 | 32.0 | 0.39 | 0 | 57 | - | RAB4A |
| 1 | 234742218 | Deletion | C | - | 1 | 25074 | Hetero | 34 | 108 | 31.5 | 0.39 | 0 | 77 | - | IRF2BP2 |
| 1 | 235922378 | SNV | C | T | 1 | 25074 | Hetero | 119 | 197 | 60.4 | 0.40 | 0 | 158 | Yes | LYST |
| 1 | 236389860 | Deletion | T | - | 1 | 25074 | Hetero | 32 | 61 | 52.5 | 0.33 | 0 | 57 | - | ERO1LB |
| 1 | 241798721 | SNV | A | G | 1 | 25074 | Hetero | 93 | 168 | 55.4 | 0.42 | 0 | 144 | No | OPN3 |
| 1 | 244219234 | SNV | C | T | 1 | 25074 | Hetero | 66 | 108 | 61.1 | 0.47 | 0 | 65 | - | ZNF238 |
| 1 | 245849127 | SNV | G | A | 1 | 25074 | Hetero | 77 | 211 | 36.5 | 0.50 | 0 | 184 | Yes | KIF26B |
| 1 | 247655180 | SNV | C | G | 1 | 25074 | Hetero | 12 | 71 | 16.9 | 0.08 | 0 | 155 | - | ENSG00000203664 |
| 1 | 249142054 | SNV | G | A | 1 | 25074 | Hetero | 62 | 161 | 38.5 | 0.39 | 0 | 172 | Yes | ZNF672 |
| 1 | 104076466 | Insertion | - | A | 1 | 25074 | Hetero | 6 | 34 | 17.6 | 0.44 | 0 | 30 | Insertion | RNPC3 |
| 1 | 110041478 | Deletion | AA | - | 2 | 25074 | Hetero | 17 | 66 | 25.8 | 0.28 | 0 | 46 | - | CYB561D1 |
| 1 | 111489897 | Insertion | - | C | 1 | 25074 | Hetero | 10 | 47 | 21.3 | 0.33 | 0 | 65 | - | RP11-96K19.2 |
| 1 | 114240319 | Insertion | - | A | 1 | 25074 | Homo | 186 | 187 | 99.5 | 0.44 | 0 | 161 | - | PHTF1 |
| 1 | 114516587 | Insertion | - | A | 1 | 25074 | Hetero | 19 | 58 | 32.8 | 0.38 | 0 | 48 | - | HIPK1 |
| 1 | 12091402 | Insertion | - | T | 1 | 25074 | Hetero | 103 | 251 | 41.0 | 0.48 | 0 | 155 | Insertion | MIIP |
| 1 | 155869435 | Insertion | - | A | 1 | 25074 | Hetero | 32 | 66 | 48.5 | 0.39 | 0 | 54 | - | RIT1 |
| 1 | 156389981 | Insertion | - | G | 1 | 25074 | Hetero | 77 | 120 | 64.2 | 0.25 | 0 | 63 | Insertion | C1orf61 |
| 1 | 162495766 | Insertion | - | T | 1 | 25074 | Hetero | 46 | 73 | 63.0 | 0.24 | 0 | 60 | - | UHMK1 |
| 1 | 16333744 | Deletion | AA | - | 2 | 25074 | Hetero | 14 | 84 | 16.7 | 0.07 | 0 | 38 | - | C1orf64 |
| 1 | 167674569 | Insertion | - | AA | 2 | 25074 | Hetero | 27 | 73 | 37.0 | 0.48 | 0 | 61 | - | RCSD1 |
| 1 | 180247012 | Insertion | - | GG | 2 | 25074 | Hetero | 33 | 63 | 52.4 | 0.49 | 0 | 46 | - | LHX4 |
| 1 | 181768859 | Insertion | - | T | 1 | 25074 | Hetero | 48 | 73 | 65.8 | 0.44 | 0 | 67 | - | CACNA1E |
| 1 | 183905132 | Insertion | - | G | 1 | 25074 | Hetero | 35 | 89 | 39.3 | 0.40 | 0 | 90 | - | GLT25D2 |
| 1 | 184590121 | Insertion | - | A | 1 | 25074 | Hetero | 17 | 38 | 44.7 | 0.44 | 0 | 33 | - | C1orf21 |
| 1 | 185069838 | Insertion | - | T | 1 | 25074 | Hetero | 10 | 49 | 20.4 | 0.30 | 0 | 38 | - | RNF2 |
| 1 | 186157206 | Deletion | AT | - | 2 | 25074 | Hetero | 88 | 147 | 59.9 | 0.33 | 0 | 124 | - | HMCN1 |
| 1 | 196642969 | Insertion | - | TT | 2 | 25074 | Homo | 77 | 82 | 93.9 | 0.29 | 0 | 58 | - | CFH |
| 1 | 19935118 | Insertion | - | A | 1 | 25074 | Hetero | 30 | 94 | 31.9 | 0.30 | 0 | 65 | - | MINOS1 |
| 1 | 201104341 | Insertion | - | A | 1 | 25074 | Hetero | 78 | 196 | 39.8 | 0.45 | 0 | 125 | - | TMEM9 |
| 1 | 201789944 | Insertion | - | T | 1 | 25074 | Homo | 48 | 53 | 90.6 | 0.46 | 0 | 37 | - | NAV1 |
| 1 | 201845882 | Insertion | - | AA | 2 | 25074 | Hetero | 9 | 75 | 12.0 | 0.11 | 0 | 47 | - | IPO9 |
| 1 | 204945678 | Insertion | - | T | 1 | 25074 | Hetero | 67 | 178 | 37.6 | 0.45 | 0 | 106 | - | NFASC |
| 1 | 208198902 | Deletion | CT | - | 2 | 25074 | Hetero | 77 | 215 | 35.8 | 0.40 | 0 | 160 | - | PLXNA2 |
| 1 | 210337097 | Deletion | AG | - | 2 | 25074 | Hetero | 32 | 70 | 45.7 | 0.43 | 0 | 55 | - | SYT14 |
| 1 | 211280725 | Deletion | AA | - | 2 | 25074 | Hetero | 67 | 104 | 64.4 | 0.21 | 0 | 80 | - | KCNH1 |
| 1 | 212900434 | Insertion | - | G | 1 | 25074 | Homo | 78 | 78 | 100.0 | 0.22 | 0 | 66 | - | NSL1 |
| 1 | 220205714 | Insertion | - | A | 1 | 25074 | Hetero | 48 | 143 | 33.6 | 0.10 | 0 | 81 | - | EPRS |
| 1 | 223804973 | Insertion | - | T | 1 | 25074 | Hetero | 30 | 73 | 41.1 | 0.42 | 0 | 63 | - | CAPN8 |
| 1 | 226332692 | Insertion | - | A | 1 | 25074 | Hetero | 54 | 87 | 62.1 | 0.44 | 0 | 60 | - | ACBD3 |
| 1 | 226819524 | Insertion | - | G | 1 | 25074 | Hetero | 50 | 85 | 58.8 | 0.44 | 0 | 61 | - | ITPKB |
| 1 | 236371755 | Insertion | - | T | 1 | 25074 | Hetero | 34 | 48 | 70.8 | 0.35 | 0 | 47 | - | GPR137B |
| 1 | 32662442 | Deletion | AT | - | 2 | 25074 | Hetero | 95 | 168 | 56.5 | 0.43 | 0 | 83 | - | TXLNA |
| 1 | 33764935 | Insertion | - | TT | 2 | 25074 | Hetero | 49 | 181 | 27.1 | 0.44 | 0 | 93 | - | ZNF362 |
| 1 | 3542106 | Insertion | - | G | 1 | 25074 | Hetero | 122 | 175 | 69.7 | 0.28 | 0 | 99 | - | TPRG1L |
| 1 | 35887397 | Insertion | - | T | 1 | 25074 | Hetero | 25 | 57 | 43.9 | 0.50 | 0 | 39 | - | ZMYM4 |
| 1 | 50883805 | Deletion | TT | - | 2 | 25074 | Hetero | 30 | 80 | 37.5 | 0.41 | 0 | 43 | - | DMRTA2 |
| 1 | 721724 | Insertion | - | T | 1 | 25074 | Hetero | 48 | 71 | 67.6 | 0.30 | 0 | 62 | - | RP11-206L10.9 |
| 1 | 75132462 | Insertion | - | A | 1 | 25074 | Hetero | 12 | 30 | 40.0 | 0.43 | 0 | 42 | - | C1orf173 |
| 2 | 3261156 | SNV | G | A | 1 | 25074 | Hetero | 53 | 127 | 41.7 | 0.44 | 0 | 75 | No | TSSC1 |
| 2 | 20872962 | SNV | G | A | 1 | 25074 | Homo | 65 | 67 | 97.0 | 0.36 | 0 | 60 | - | GDF7 |
| 2 | 24260812 | SNV | A | G | 1 | 25074 | Hetero | 46 | 123 | 37.4 | 0.44 | 0 | 90 | Yes | MFSD2B |
| 2 | 27455880 | SNV | T | G | 1 | 25074 | Hetero | 73 | 108 | 67.6 | 0.34 | 0 | 68 | - | CAD |
| 2 | 29294325 | SNV | C | T | 1 | 25074 | Hetero | 46 | 100 | 46.0 | 0.48 | 0 | 91 | Yes | C2orf71 |
| 2 | 30993184 | SNV | A | G | 1 | 25074 | Hetero | 14 | 44 | 31.8 | 0.14 | 0 | 36 | No | CAPN13 |
| 2 | 36777345 | SNV | A | G | 1 | 25074 | Hetero | 26 | 72 | 36.1 | 0.38 | 0 | 51 | - | CRIM1 |
| 2 | 46707812 | SNV | G | A | 1 | 25074 | Hetero | 26 | 206 | 12.6 | 0.33 | 0 | 119 | Yes | TMEM247 |
| 2 | 48825620 | SNV | A | G | 1 | 25074 | Hetero | 22 | 37 | 59.5 | 0.22 | 0 | 39 | - | STON1 |
| 2 | 55198181 | SNV | G | A | 1 | 25074 | Hetero | 13 | 48 | 27.1 | 0.20 | 0 | 46 | - | EML6 |
| 2 | 62449333 | SNV | C | T | 1 | 25074 | Hetero | 50 | 171 | 29.2 | 0.32 | 0 | 150 | - | B3GNT2 |
| 2 | 63273196 | SNV | G | A | 1 | 25074 | Hetero | 36 | 85 | 42.4 | 0.44 | 0 | 48 | - | EHBP1 |
| 2 | 65539600 | SNV | C | G | 1 | 25074 | Hetero | 14 | 61 | 23.0 | 0.43 | 0 | 61 | - | SPRED2 |
| 2 | 70438553 | SNV | T | G | 1 | 25074 | Hetero | 21 | 52 | 40.4 | 0.46 | 0 | 40 | - | C2orf42 |
| 2 | 85839134 | SNV | C | T | 1 | 25074 | Hetero | 79 | 121 | 65.3 | 0.17 | 0 | 75 | - | USP39 |
| 2 | 85925714 | SNV | C | T | 1 | 25074 | Hetero | 115 | 290 | 39.7 | 0.38 | 0 | 265 | No | GNLY |
| 2 | 86422793 | SNV | G | C | 1 | 25074 | Hetero | 37 | 131 | 28.2 | 0.50 | 0 | 105 | - | IMMT |
| 2 | 86459725 | SNV | A | C | 1 | 25074 | Hetero | 25 | 76 | 32.9 | 0.23 | 0 | 56 | - | REEP1 |
| 2 | 87131091 | SNV | G | A | 1 | 25074 | Hetero | 17 | 109 | 15.6 | 0.43 | 0 | 101 | - | ANAPC1P1 |
| 2 | 89028920 | SNV | G | T | 1 | 25074 | Hetero | 107 | 186 | 57.5 | 0.42 | 0 | 170 | - | RPIA |
| 2 | 97877440 | SNV | T | C | 1 | 25074 | Hetero | 76 | 156 | 48.7 | 0.40 | 0 | 51 | Yes | ANKRD36 |
| 2 | 98809516 | SNV | G | T | 1 | 25074 | Hetero | 37 | 80 | 46.3 | 0.43 | 0 | 62 | - | VWA3B |
| 2 | 102954786 | SNV | G | C | 1 | 25074 | Hetero | 23 | 86 | 26.7 | 0.22 | 0 | 56 | - | IL1RL1 |
| 2 | 105883674 | SNV | C | G | 1 | 25074 | Hetero | 10 | 100 | 10.0 | 0.20 | 0 | 105 | - | TGFBRAP1 |
| 2 | 114709374 | SNV | A | T | 1 | 25074 | Hetero | 86 | 253 | 34.0 | 0.38 | 0 | 209 | No | ACTR3 |
| 2 | 121978977 | SNV | A | T | 1 | 25074 | Hetero | 29 | 93 | 31.2 | 0.33 | 0 | 45 | - | TFCP2L1 |
| 2 | 128057010 | SNV | A | G | 1 | 25074 | Hetero | 42 | 84 | 50.0 | 0.44 | 0 | 87 | - | MAP3K2 |
| 2 | 128699005 | SNV | G | C | 1 | 25074 | Hetero | 36 | 62 | 58.1 | 0.42 | 0 | 46 | - | SAP130 |
| 2 | 129025141 | Deletion | C | - | 1 | 25074 | Hetero | 9 | 64 | 14.1 | 0.44 | 0 | 41 | - | HS6ST1 |
| 2 | 129025616 | SNV | A | T | 1 | 25074 | Hetero | 91 | 195 | 46.7 | 0.36 | 0 | 141 | - | HS6ST1 |
| 2 | 135210857 | SNV | A | C | 1 | 25074 | Hetero | 37 | 63 | 58.7 | 0.49 | 0 | 43 | - | MGAT5 |
| 2 | 141528542 | SNV | C | G | 1 | 25074 | Hetero | 18 | 58 | 31.0 | 0.40 | 0 | 49 | Yes | LRP1B |
| 2 | 152319900 | SNV | G | A | 1 | 25074 | Hetero | 42 | 172 | 24.4 | 0.35 | 0 | 125 | Yes | RIF1 |
| 2 | 152492875 | SNV | T | C | 1 | 25074 | Hetero | 13 | 53 | 24.5 | 0.20 | 0 | 59 | - | NEB |
| 2 | 163136575 | SNV | G | A | 1 | 25074 | Hetero | 18 | 33 | 54.5 | 0.24 | 0 | 31 | No | IFIH1 |
| 2 | 170494852 | Deletion | A | - | 1 | 25074 | Hetero | 6 | 58 | 10.3 | 0.17 | 0 | 47 | - | PPIG |
| 2 | 172700841 | SNV | T | C | 1 | 25074 | Hetero | 17 | 62 | 27.4 | 0.19 | 0 | 54 | - | SLC25A12 |
| 2 | 173782287 | SNV | A | G | 1 | 25074 | Hetero | 11 | 30 | 36.7 | 0.33 | 0 | 30 | - | RAPGEF4 |
| 2 | 174090336 | SNV | A | C | 1 | 25074 | Hetero | 29 | 62 | 46.8 | 0.17 | 0 | 58 | - | AC013461.1 |
| 2 | 174232941 | SNV | C | G | 1 | 25074 | Hetero | 37 | 56 | 66.1 | 0.36 | 0 | 59 | - | CDCA7 |
| 2 | 175304801 | SNV | A | G | 1 | 25074 | Hetero | 24 | 67 | 35.8 | 0.37 | 0 | 55 | - | GPR155 |
| 2 | 179263053 | SNV | C | T | 1 | 25074 | Hetero | 41 | 82 | 50.0 | 0.29 | 0 | 63 | - | OSBPL6 |
| 2 | 179843214 | SNV | T | G | 1 | 25074 | Hetero | 37 | 80 | 46.3 | 0.24 | 0 | 87 | Yes | CCDC141 |
| 2 | 190426147 | SNV | G | C | 1 | 25074 | Hetero | 34 | 57 | 59.6 | 0.31 | 0 | 56 | - | SLC40A1 |
| 2 | 191745632 | SNV | G | T | 1 | 25074 | Hetero | 28 | 160 | 17.5 | 0.14 | 0 | 151 | - | GLS |
| 2 | 191745637 | SNV | A | T | 1 | 25074 | Hetero | 21 | 171 | 12.3 | 0.19 | 0 | 157 | - | GLS |
| 2 | 196600869 | SNV | G | A | 1 | 25074 | Hetero | 33 | 63 | 52.4 | 0.36 | 0 | 38 | - | SLC39A10 |
| 2 | 200136284 | Deletion | A | - | 1 | 25074 | Hetero | 25 | 41 | 61.0 | 0.41 | 0 | 35 | - | SATB2 |
| 2 | 201281084 | SNV | T | G | 1 | 25074 | Hetero | 118 | 188 | 62.8 | 0.44 | 0 | 129 | - | SPATS2L |
| 2 | 202146556 | SNV | G | A | 1 | 25074 | Hetero | 37 | 116 | 31.9 | 0.43 | 0 | 118 | - | CASP8 |
| 2 | 207941429 | SNV | T | C | 1 | 25074 | Hetero | 52 | 136 | 38.2 | 0.41 | 0 | 124 | - | KLF7 |
| 2 | 209308094 | SNV | C | G | 1 | 25074 | Hetero | 69 | 125 | 55.2 | 0.18 | 0 | 96 | Yes | PTH2R |
| 2 | 210596838 | SNV | C | A | 1 | 25074 | Hetero | 10 | 43 | 23.3 | 0.18 | 0 | 36 | - | MAP2 |
| 2 | 218937684 | SNV | A | C | 1 | 25074 | Hetero | 29 | 88 | 33.0 | 0.44 | 0 | 49 | - | RUFY4 |
| 2 | 219127197 | SNV | T | C | 1 | 25074 | Hetero | 33 | 97 | 34.0 | 0.48 | 0 | 81 | - | GPBAR1 |
| 2 | 220084392 | SNV | C | T | 1 | 25074 | Hetero | 44 | 134 | 32.8 | 0.38 | 0 | 119 | - | ATG9A |
| 2 | 220155683 | SNV | A | G | 1 | 25074 | Hetero | 48 | 130 | 36.9 | 0.19 | 0 | 117 | - | PTPRN |
| 2 | 220163703 | SNV | C | T | 1 | 25074 | Hetero | 105 | 159 | 66.0 | 0.30 | 0 | 108 | - | PTPRN |
| 2 | 220326698 | SNV | G | A | 1 | 25074 | Hetero | 75 | 179 | 41.9 | 0.27 | 0 | 103 | No | SPEG |
| 2 | 220492665 | SNV | G | A | 1 | 25074 | Hetero | 26 | 61 | 42.6 | 0.34 | 0 | 37 | - | SLC4A3 |
| 2 | 225335226 | Deletion | A | - | 1 | 25074 | Hetero | 26 | 63 | 41.3 | 0.43 | 0 | 69 | - | CUL3 |
| 2 | 229890114 | SNV | C | G | 1 | 25074 | Hetero | 101 | 156 | 64.7 | 0.47 | 0 | 133 | - | PID1 |
| 2 | 231033864 | SNV | G | A | 1 | 25074 | Hetero | 70 | 169 | 41.4 | 0.36 | 0 | 119 | No | SP110 |
| 2 | 232262945 | SNV | G | A | 1 | 25074 | Hetero | 65 | 110 | 59.1 | 0.50 | 0 | 98 | Yes | B3GNT7 |
| 2 | 232575278 | SNV | C | G | 1 | 25074 | Hetero | 85 | 186 | 45.7 | 0.35 | 0 | 127 | Yes | PTMA |
| 2 | 232790344 | SNV | C | T | 1 | 25074 | Hetero | 57 | 92 | 62.0 | 0.22 | 0 | 52 | Yes | NPPC |
| 2 | 233164819 | SNV | G | A | 1 | 25074 | Hetero | 84 | 207 | 40.6 | 0.30 | 0 | 164 | Yes | DIS3L2 |
| 2 | 233274210 | SNV | G | T | 1 | 25074 | Hetero | 43 | 101 | 42.6 | 0.07 | 0 | 126 | - | ALPPL2 |
| 2 | 233349291 | SNV | G | A | 1 | 25074 | Hetero | 78 | 222 | 35.1 | 0.49 | 0 | 163 | No | ECEL1 |
| 2 | 234969077 | SNV | G | A | 1 | 25074 | Hetero | 100 | 146 | 68.5 | 0.39 | 0 | 78 | Yes | SPP2 |
| 2 | 239970299 | SNV | T | A | 1 | 25074 | Hetero | 7 | 67 | 10.4 | 0.43 | 0 | 56 | - | HDAC4 |
| 2 | 101581294 | Insertion | - | TT | 2 | 25074 | Hetero | 56 | 113 | 49.6 | 0.28 | 0 | 60 | - | NPAS2 |
| 2 | 120932994 | Insertion | - | T | 1 | 25074 | Hetero | 10 | 90 | 11.1 | 0.45 | 0 | 51 | - | EPB41L5 |
| 2 | 128620363 | Insertion | - | T | 1 | 25074 | Hetero | 15 | 47 | 31.9 | 0.25 | 0 | 34 | - | AMMECR1L |
| 2 | 135214418 | Insertion | - | T | 1 | 25074 | Hetero | 22 | 88 | 25.0 | 0.13 | 0 | 75 | - | TMEM163 |
| 2 | 158958551 | Insertion | - | A | 1 | 25074 | Hetero | 20 | 65 | 30.8 | 0.30 | 0 | 38 | - | UPP2 |
| 2 | 170606424 | Deletion | TA | - | 2 | 25074 | Hetero | 39 | 105 | 37.1 | 0.10 | 0 | 62 | - | KLHL23 |
| 2 | 174090336 | Insertion | - | C | 1 | 25074 | Hetero | 10 | 64 | 15.6 | 0.20 | 0 | 58 | - | AC013461.1 |
| 2 | 186603605 | Insertion | - | G | 1 | 25074 | Hetero | 26 | 83 | 31.3 | 0.29 | 0 | 60 | Insertion | FSIP2 |
| 2 | 196602328 | Deletion | TT | - | 2 | 25074 | Hetero | 15 | 42 | 35.7 | 0.13 | 0 | 32 | - | SLC39A10 |
| 2 | 197644939 | Insertion | - | T | 1 | 25074 | Hetero | 36 | 60 | 60.0 | 0.33 | 0 | 42 | - | GTF3C3 |
| 2 | 202529007 | Deletion | AC | - | 2 | 25074 | Hetero | 99 | 163 | 60.7 | 0.35 | 0 | 136 | Deletion | MPP4 |
| 2 | 20872960 | Insertion | - | AT | 2 | 25074 | Homo | 69 | 69 | 100.0 | 0.37 | 0 | 59 | - | GDF7 |
| 2 | 215590761 | Insertion | - | A | 1 | 25074 | Hetero | 21 | 39 | 53.8 | 0.38 | 0 | 45 | - | BARD1 |
| 2 | 218750453 | Insertion | - | G | 1 | 25074 | Homo | 81 | 81 | 100.0 | 0.40 | 0 | 64 | - | TNS1 |
| 2 | 224629851 | Insertion | - | T | 1 | 25074 | Hetero | 74 | 223 | 33.2 | 0.36 | 0 | 157 | - | AP1S3 |
| 2 | 234668879 | Insertion | - | AT | 2 | 25074 | Hetero | 25 | 41 | 61.0 | 0.22 | 0 | 44 | - | UGT1A8 |
| 2 | 27353979 | Insertion | - | A | 1 | 25074 | Hetero | 36 | 84 | 42.9 | 0.34 | 0 | 59 | - | PREB |
| 2 | 33788061 | Deletion | TT | - | 2 | 25074 | Hetero | 22 | 71 | 31.0 | 0.48 | 0 | 83 | - | RASGRP3 |
| 2 | 37333711 | Insertion | - | T | 1 | 25074 | Hetero | 5 | 44 | 11.4 | 0.33 | 0 | 30 | - | EIF2AK2 |
| 2 | 42577220 | Insertion | - | GG | 2 | 25074 | Hetero | 54 | 140 | 38.6 | 0.38 | 0 | 115 | - | COX7A2L |
| 2 | 50148140 | Insertion | - | A | 1 | 25074 | Hetero | 63 | 165 | 38.2 | 0.47 | 0 | 130 | - | NRXN1 |
| 2 | 64861511 | Insertion | - | G | 1 | 25074 | Hetero | 17 | 81 | 21.0 | 0.06 | 0 | 61 | - | SERTAD2 |
| 2 | 71175511 | Insertion | - | AA | 2 | 25074 | Hetero | 60 | 92 | 65.2 | 0.36 | 0 | 79 | - | ATP6V1B1 |
| 2 | 74751022 | Insertion | - | T | 1 | 25074 | Hetero | 61 | 153 | 39.9 | 0.25 | 0 | 129 | - | DQX1 |
| 2 | 75883007 | Insertion | - | AA | 2 | 25074 | Hetero | 67 | 108 | 62.0 | 0.39 | 0 | 83 | - | MRPL19 |
| 2 | 80875726 | Insertion | - | T | 1 | 25074 | Hetero | 19 | 55 | 34.5 | 0.48 | 0 | 52 | - | CTNNA2 |
| 2 | 95830737 | Insertion | - | A | 1 | 25074 | Homo | 74 | 74 | 100.0 | 0.15 | 0 | 66 | - | ZNF514 |
| 3 | 3133907 | SNV | C | G | 1 | 25074 | Hetero | 140 | 188 | 74.5 | 0.46 | 0 | 197 | Possible splice site disruption | IL5RA |
| 3 | 8543404 | SNV | C | T | 1 | 25074 | Hetero | 46 | 54 | 85.2 | 0.40 | 0 | 50 | - | AC087859.1 |
| 3 | 9986517 | SNV | A | G | 1 | 25074 | Hetero | 14 | 65 | 21.5 | 0.47 | 0 | 60 | - | CRELD1 |
| 3 | 10106408 | SNV | C | T | 1 | 25074 | Hetero | 7 | 45 | 15.6 | 0.14 | 0 | 30 | - | FANCD2 |
| 3 | 10319975 | SNV | C | T | 1 | 25074 | Hetero | 44 | 58 | 75.9 | 0.25 | 0 | 70 | No | TATDN2 |
| 3 | 11468322 | SNV | G | T | 1 | 25074 | Hetero | 60 | 100 | 60.0 | 0.26 | 0 | 89 | Yes | ATG7 |
| 3 | 16645935 | SNV | G | A | 1 | 25074 | Hetero | 16 | 93 | 17.2 | 0.43 | 0 | 88 | - | DAZL |
| 3 | 19432180 | SNV | C | T | 1 | 25074 | Hetero | 61 | 100 | 61.0 | 0.08 | 0 | 100 | - | KCNH8 |
| 3 | 33467071 | SNV | A | C | 1 | 25074 | Hetero | 47 | 57 | 82.5 | 0.16 | 0 | 72 | - | UBP1 |
| 3 | 36756370 | Deletion | T | - | 1 | 25074 | Homo | 52 | 53 | 98.1 | 0.40 | 0 | 58 | - | DCLK3 |
| 3 | 37095934 | SNV | A | G | 1 | 25074 | Hetero | 34 | 168 | 20.2 | 0.33 | 0 | 147 | No | MLH1 |
| 3 | 39452655 | SNV | C | G | 1 | 25074 | Hetero | 21 | 81 | 25.9 | 0.43 | 0 | 78 | - | RPSA |
| 3 | 41746553 | SNV | G | C | 1 | 25074 | Hetero | 16 | 36 | 44.4 | 0.13 | 0 | 30 | Yes | ULK4 |
| 3 | 41831195 | SNV | C | T | 1 | 25074 | Hetero | 18 | 125 | 14.4 | 0.50 | 0 | 144 | Yes | ULK4 |
| 3 | 42914137 | SNV | G | A | 1 | 25074 | Hetero | 74 | 84 | 88.1 | 0.43 | 0 | 79 | - | CCBP2 |
| 3 | 45879588 | SNV | A | T | 1 | 25074 | Hetero | 18 | 87 | 20.7 | 0.23 | 0 | 75 | - | LZTFL1 |
| 3 | 52087121 | SNV | C | G | 1 | 25074 | Hetero | 77 | 94 | 81.9 | 0.32 | 0 | 89 | - | DUSP7 |
| 3 | 53326891 | SNV | G | A | 1 | 25074 | Hetero | 6 | 53 | 11.3 | 0.33 | 0 | 46 | - | DCP1A |
| 3 | 53901491 | SNV | C | T | 1 | 25074 | Hetero | 44 | 57 | 77.2 | 0.39 | 0 | 83 | - | ACTR8 |
| 3 | 63601400 | SNV | C | T | 1 | 25074 | Hetero | 4 | 35 | 11.4 | 0.20 | 0 | 48 | - | SYNPR |
| 3 | 67060254 | SNV | A | G | 1 | 25074 | Hetero | 48 | 62 | 77.4 | 0.49 | 0 | 86 | - | KBTBD8 |
| 3 | 70015500 | SNV | G | A | 1 | 25074 | Hetero | 40 | 50 | 80.0 | 0.38 | 0 | 51 | - | MITF |
| 3 | 73457170 | SNV | A | G | 1 | 25074 | Hetero | 70 | 86 | 81.4 | 0.16 | 0 | 128 | - | PDZRN3 |
| 3 | 75832522 | SNV | G | A | 1 | 25074 | Hetero | 133 | 182 | 73.1 | 0.41 | 0 | 250 | - | ZNF717 |
| 3 | 98298460 | SNV | T | A | 1 | 25074 | Hetero | 31 | 52 | 59.6 | 0.41 | 0 | 37 | - | CPOX |
| 3 | 111432763 | SNV | C | T | 1 | 25074 | Hetero | 83 | 166 | 50.0 | 0.19 | 0 | 99 | No | PLCXD2 |
| 3 | 112714068 | SNV | A | C | 1 | 25074 | Hetero | 163 | 349 | 46.7 | 0.37 | 0 | 196 | Yes | GTPBP8 |
| 3 | 113300220 | SNV | G | T | 1 | 25074 | Hetero | 44 | 96 | 45.8 | 0.43 | 0 | 54 | Yes | SIDT1 |
| 3 | 113955736 | SNV | G | C | 1 | 25074 | Hetero | 77 | 146 | 52.7 | 0.25 | 0 | 88 | Yes | ZNF80 |
| 3 | 119367498 | SNV | C | T | 1 | 25074 | Hetero | 72 | 159 | 45.3 | 0.40 | 0 | 77 | No | POPDC2 |
| 3 | 122186128 | SNV | A | C | 1 | 25074 | Hetero | 42 | 85 | 49.4 | 0.16 | 0 | 47 | - | KPNA1 |
| 3 | 122291122 | SNV | G | A | 1 | 25074 | Hetero | 47 | 91 | 51.6 | 0.36 | 0 | 88 | - | DTX3L |
| 3 | 124630963 | SNV | C | T | 1 | 25074 | Hetero | 85 | 178 | 47.8 | 0.22 | 0 | 107 | Yes | MUC13 |
| 3 | 124949219 | SNV | T | A | 1 | 25074 | Hetero | 59 | 107 | 55.1 | 0.42 | 0 | 53 | - | SLC12A8 |
| 3 | 128631441 | SNV | G | A | 1 | 25074 | Hetero | 145 | 304 | 47.7 | 0.38 | 0 | 153 | No | ACAD9 |
| 3 | 128808767 | SNV | C | T | 1 | 25074 | Hetero | 106 | 172 | 61.6 | 0.16 | 0 | 135 | - | RAB43 |
| 3 | 129034521 | SNV | C | T | 1 | 25074 | Hetero | 141 | 272 | 51.8 | 0.48 | 0 | 127 | No | H1FX |
| 3 | 130285633 | SNV | C | T | 1 | 25074 | Hetero | 54 | 138 | 39.1 | 0.26 | 0 | 85 | Yes | COL6A6 |
| 3 | 133524442 | SNV | G | C | 1 | 25074 | Hetero | 64 | 110 | 58.2 | 0.18 | 0 | 71 | - | SRPRB |
| 3 | 133876676 | SNV | G | A | 1 | 25074 | Hetero | 32 | 77 | 41.6 | 0.40 | 0 | 69 | - | RYK |
| 3 | 137906844 | SNV | G | A | 1 | 25074 | Hetero | 126 | 247 | 51.0 | 0.42 | 0 | 147 | - | ARMC8 |
| 3 | 142513099 | Deletion | A | - | 1 | 25074 | Hetero | 32 | 86 | 37.2 | 0.40 | 0 | 43 | - | TRPC1 |
| 3 | 149038843 | Deletion | T | - | 1 | 25074 | Hetero | 33 | 56 | 58.9 | 0.39 | 0 | 33 | - | RP11-206M11.7 |
| 3 | 160939763 | SNV | T | C | 1 | 25074 | Hetero | 165 | 309 | 53.4 | 0.22 | 0 | 80 | - | NMD3 |
| 3 | 167727218 | Deletion | C | - | 1 | 25074 | Hetero | 13 | 62 | 21.0 | 0.08 | 0 | 35 | - | GOLIM4 |
| 3 | 173322501 | SNV | T | A | 1 | 25074 | Hetero | 36 | 322 | 11.2 | 0.31 | 0 | 220 | Yes | NLGN1 |
| 3 | 183206801 | SNV | A | G | 1 | 25074 | Hetero | 84 | 158 | 53.2 | 0.32 | 0 | 97 | - | KLHL6 |
| 3 | 183976690 | SNV | G | T | 1 | 25074 | Hetero | 158 | 319 | 49.5 | 0.40 | 0 | 208 | - | EIF2B5 |
| 3 | 184079116 | SNV | G | C | 1 | 25074 | Hetero | 61 | 135 | 45.2 | 0.21 | 0 | 87 | - | EIF2B5 |
| 3 | 184542684 | SNV | C | G | 1 | 25074 | Hetero | 33 | 87 | 37.9 | 0.24 | 0 | 58 | - | VPS8 |
| 3 | 185207968 | SNV | C | A | 1 | 25074 | Hetero | 62 | 110 | 56.4 | 0.41 | 0 | 84 | - | TMEM41A |
| 3 | 195345354 | SNV | T | C | 1 | 25074 | Hetero | 101 | 289 | 34.9 | 0.37 | 0 | 169 | - | AC069213.1 |
| 3 | 195395395 | SNV | G | A | 1 | 25074 | Hetero | 50 | 326 | 15.3 | 0.36 | 0 | 116 | - | MUC20 |
| 3 | 195451108 | SNV | G | A | 1 | 25074 | Hetero | 89 | 144 | 61.8 | 0.35 | 0 | 111 | - | MUC20 |
| 3 | 195594795 | SNV | G | A | 1 | 25074 | Hetero | 93 | 181 | 51.4 | 0.48 | 0 | 106 | Yes | TNK2 |
| 3 | 197241246 | SNV | C | T | 1 | 25074 | Hetero | 87 | 189 | 46.0 | 0.33 | 0 | 148 | Yes | BDH1 |
| 3 | 197510273 | SNV | T | G | 1 | 25074 | Hetero | 52 | 114 | 45.6 | 0.42 | 0 | 92 | - | FYTTD1 |
| 3 | 101541680 | Insertion | - | T | 1 | 25074 | Hetero | 16 | 74 | 21.6 | 0.47 | 0 | 55 | - | NXPE3 |
| 3 | 112218319 | Insertion | - | A | 1 | 25074 | Hetero | 4 | 40 | 10.0 | 0.50 | 0 | 33 | - | BTLA |
| 3 | 119540967 | Insertion | - | AA | 2 | 25074 | Hetero | 40 | 102 | 39.2 | 0.31 | 0 | 58 | - | GSK3B |
| 3 | 120363328 | Insertion | - | A | 1 | 25074 | Hetero | 97 | 229 | 42.4 | 0.50 | 0 | 154 | - | HGD |
| 3 | 121143384 | Insertion | - | T | 1 | 25074 | Hetero | 28 | 59 | 47.5 | 0.28 | 0 | 55 | - | STXBP5L |
| 3 | 121634171 | Insertion | - | T | 1 | 25074 | Homo | 136 | 136 | 100.0 | 0.26 | 0 | 70 | - | SLC15A2 |
| 3 | 124949837 | Insertion | - | A | 1 | 25074 | Hetero | 41 | 91 | 45.1 | 0.45 | 0 | 61 | - | SLC12A8 |
| 3 | 129252686 | Deletion | TT | - | 2 | 25074 | Hetero | 30 | 84 | 35.7 | 0.13 | 0 | 36 | - | RHO |
| 3 | 137748822 | Insertion | - | A | 1 | 25074 | Hetero | 31 | 64 | 48.4 | 0.09 | 0 | 46 | - | CLDN18 |
| 3 | 14509654 | Insertion | - | T | 1 | 25074 | Hetero | 15 | 54 | 27.8 | 0.47 | 0 | 51 | Insertion | SLC6A6 |
| 3 | 157296510 | Insertion | - | A | 1 | 25074 | Hetero | 21 | 71 | 29.6 | 0.43 | 0 | 32 | - | C3orf55 |
| 3 | 160137363 | Insertion | - | T | 1 | 25074 | Hetero | 23 | 52 | 44.2 | 0.08 | 0 | 30 | - | SMC4 |
| 3 | 173999114 | Insertion | - | T | 1 | 25074 | Hetero | 27 | 236 | 11.4 | 0.23 | 0 | 205 | - | NLGN1 |
| 3 | 179342302 | Insertion | - | T | 1 | 25074 | Hetero | 16 | 38 | 42.1 | 0.25 | 0 | 35 | - | NDUFB5 |
| 3 | 184971025 | Deletion | AA | - | 2 | 25074 | Hetero | 9 | 87 | 10.3 | 0.44 | 0 | 34 | - | EHHADH |
| 3 | 186936376 | Insertion | - | AT | 2 | 25074 | Hetero | 65 | 75 | 86.7 | 0.47 | 0 | 44 | - | MASP1 |
| 3 | 188326926 | Insertion | - | T | 1 | 25074 | Hetero | 65 | 117 | 55.6 | 0.14 | 0 | 50 | - | LPP |
| 3 | 194219775 | Insertion | - | A | 1 | 25074 | Hetero | 4 | 33 | 12.1 | 0.20 | 0 | 36 | - | AC046143.7 |
| 3 | 194896240 | Insertion | - | G | 1 | 25074 | Hetero | 70 | 127 | 55.1 | 0.25 | 0 | 69 | - | XXYLT1 |
| 3 | 25639973 | Insertion | - | T | 1 | 25074 | Homo | 91 | 92 | 98.9 | 0.11 | 0 | 109 | - | TOP2B |
| 3 | 28362314 | Insertion | - | T | 1 | 25074 | Homo | 43 | 43 | 100.0 | 0.49 | 0 | 57 | - | CMC1 |
| 3 | 38319245 | Insertion | - | T | 1 | 25074 | Hetero | 21 | 106 | 19.8 | 0.45 | 0 | 122 | - | SLC22A13 |
| 3 | 42706335 | Insertion | - | T | 1 | 25074 | Hetero | 30 | 84 | 35.7 | 0.39 | 0 | 51 | - | ZBTB47 |
| 3 | 52728312 | Insertion | - | T | 1 | 25074 | Homo | 114 | 121 | 94.2 | 0.33 | 0 | 96 | - | GNL3 |
| 4 | 338228 | SNV | G | A | 1 | 25074 | Hetero | 10 | 55 | 18.2 | 0.31 | 0 | 65 | - | ZNF141 |
| 4 | 378347 | SNV | T | G | 1 | 25074 | Hetero | 30 | 128 | 23.4 | 0.32 | 0 | 154 | - | ZNF141 |
| 4 | 433839 | SNV | T | C | 1 | 25074 | Hetero | 8 | 45 | 17.8 | 0.33 | 0 | 50 | - | ABCA11P |
| 4 | 502725 | SNV | G | T | 1 | 25074 | Hetero | 31 | 111 | 27.9 | 0.41 | 0 | 120 | No | PIGG |
| 4 | 727648 | SNV | C | T | 1 | 25074 | Hetero | 203 | 270 | 75.2 | 0.35 | 0 | 269 | Yes | PCGF3 |
| 4 | 946995 | SNV | C | T | 1 | 25074 | Hetero | 63 | 245 | 25.7 | 0.39 | 0 | 159 | No | TMEM175 |
| 4 | 950156 | SNV | C | T | 1 | 25074 | Hetero | 157 | 208 | 75.5 | 0.45 | 0 | 177 | - | TMEM175 |
| 4 | 1719923 | SNV | G | C | 1 | 25074 | Hetero | 44 | 67 | 65.7 | 0.11 | 0 | 55 | Yes | TMEM129 |
| 4 | 1746455 | SNV | G | A | 1 | 25074 | Hetero | 57 | 126 | 45.2 | 0.43 | 0 | 53 | Yes | TACC3 |
| 4 | 1807555 | SNV | T | C | 1 | 25074 | Hetero | 63 | 113 | 55.8 | 0.39 | 0 | 65 | Yes | FGFR3 |
| 4 | 2968181 | SNV | T | G | 1 | 25074 | Hetero | 44 | 82 | 53.7 | 0.27 | 0 | 42 | - | GRK4 |
| 4 | 3445089 | SNV | C | T | 1 | 25074 | Hetero | 63 | 157 | 40.1 | 0.45 | 0 | 87 | No | HGFAC |
| 4 | 6882580 | SNV | C | T | 1 | 25074 | Hetero | 66 | 158 | 41.8 | 0.44 | 0 | 122 | Yes | KIAA0232 |
| 4 | 7760937 | SNV | C | T | 1 | 25074 | Hetero | 49 | 101 | 48.5 | 0.34 | 0 | 87 | - | AFAP1 |
| 4 | 8234208 | SNV | G | C | 1 | 25074 | Hetero | 100 | 174 | 57.5 | 0.45 | 0 | 120 | - | SH3TC1 |
| 4 | 8603180 | SNV | C | T | 1 | 25074 | Hetero | 169 | 317 | 53.3 | 0.41 | 0 | 253 | Yes | GPR78 |
| 4 | 8603240 | SNV | G | A | 1 | 25074 | Hetero | 89 | 178 | 50.0 | 0.23 | 0 | 139 | - | GPR78 |
| 4 | 10118323 | SNV | G | T | 1 | 25074 | Hetero | 69 | 119 | 58.0 | 0.42 | 0 | 84 | - | WDR1 |
| 4 | 13475839 | SNV | A | G | 1 | 25074 | Hetero | 68 | 115 | 59.1 | 0.45 | 0 | 80 | - | RAB28 |
| 4 | 15504086 | SNV | C | T | 1 | 25074 | Hetero | 22 | 72 | 30.6 | 0.36 | 0 | 41 | No | CC2D2A |
| 4 | 15606692 | SNV | T | A | 1 | 25074 | Hetero | 46 | 82 | 56.1 | 0.49 | 0 | 57 | - | FBXL5 |
| 4 | 16020080 | SNV | T | G | 1 | 25074 | Hetero | 18 | 37 | 48.6 | 0.25 | 0 | 30 | Yes | PROM1 |
| 4 | 25004713 | Deletion | T | - | 1 | 25074 | Homo | 77 | 77 | 100.0 | 0.44 | 0 | 60 | - | LGI2 |
| 4 | 26274356 | SNV | G | A | 1 | 25074 | Hetero | 136 | 253 | 53.8 | 0.50 | 0 | 131 | Yes | RBPJ |
| 4 | 37448960 | SNV | T | G | 1 | 25074 | Hetero | 26 | 150 | 17.3 | 0.24 | 0 | 49 | - | KIAA1239 |
| 4 | 37649129 | SNV | G | T | 1 | 25074 | Hetero | 45 | 299 | 15.1 | 0.33 | 0 | 83 | - | RELL1 |
| 4 | 38690508 | SNV | G | A | 1 | 25074 | Hetero | 227 | 361 | 62.9 | 0.39 | 0 | 284 | No | KLF3 |
| 4 | 38798792 | SNV | T | C | 1 | 25074 | Hetero | 163 | 263 | 62.0 | 0.44 | 0 | 196 | Yes | TLR1 |
| 4 | 41687843 | SNV | C | T | 1 | 25074 | Hetero | 75 | 120 | 62.5 | 0.09 | 0 | 105 | Yes | LIMCH1 |
| 4 | 46040018 | SNV | T | C | 1 | 25074 | Hetero | 42 | 65 | 64.6 | 0.49 | 0 | 58 | - | GABRG1 |
| 4 | 46040579 | SNV | A | C | 1 | 25074 | Hetero | 45 | 105 | 42.9 | 0.38 | 0 | 59 | - | GABRG1 |
| 4 | 46390785 | SNV | T | A | 1 | 25074 | Hetero | 50 | 215 | 23.3 | 0.48 | 0 | 168 | - | GABRA2 |
| 4 | 46923181 | SNV | A | C | 1 | 25074 | Hetero | 23 | 40 | 57.5 | 0.48 | 0 | 59 | - | GABRA4 |
| 4 | 48582762 | SNV | C | T | 1 | 25074 | Hetero | 23 | 39 | 59.0 | 0.43 | 0 | 40 | - | FRYL |
| 4 | 54244102 | SNV | C | T | 1 | 25074 | Hetero | 103 | 296 | 34.8 | 0.42 | 0 | 234 | - | FIP1L1 |
| 4 | 55956163 | SNV | C | T | 1 | 25074 | Hetero | 43 | 112 | 38.4 | 0.24 | 0 | 71 | Yes | KDR |
| 4 | 57333820 | SNV | G | T | 1 | 25074 | Hetero | 164 | 268 | 61.2 | 0.33 | 0 | 224 | Yes | SRP72 |
| 4 | 74352723 | SNV | C | T | 1 | 25074 | Hetero | 69 | 105 | 65.7 | 0.28 | 0 | 84 | No | AFM |
| 4 | 74486100 | SNV | G | T | 1 | 25074 | Hetero | 81 | 152 | 53.3 | 0.39 | 0 | 114 | - | RASSF6 |
| 4 | 74702360 | SNV | T | G | 1 | 25074 | Hetero | 22 | 79 | 27.8 | 0.47 | 0 | 69 | - | CXCL6 |
| 4 | 76676600 | SNV | C | G | 1 | 25074 | Hetero | 40 | 100 | 40.0 | 0.16 | 0 | 82 | - | USO1 |
| 4 | 76734697 | SNV | A | G | 1 | 25074 | Hetero | 28 | 52 | 53.8 | 0.45 | 0 | 58 | - | USO1 |
| 4 | 79833808 | SNV | G | T | 1 | 25074 | Hetero | 49 | 89 | 55.1 | 0.35 | 0 | 71 | - | BMP2K |
| 4 | 80824525 | SNV | T | A | 1 | 25074 | Hetero | 5 | 47 | 10.6 | 0.40 | 0 | 39 | - | ANTXR2 |
| 4 | 84240515 | SNV | T | G | 1 | 25074 | Hetero | 69 | 161 | 42.9 | 0.46 | 0 | 124 | Yes | HPSE |
| 4 | 85570768 | SNV | G | A | 1 | 25074 | Hetero | 60 | 130 | 46.2 | 0.41 | 0 | 97 | - | CDS1 |
| 4 | 85572427 | SNV | A | G | 1 | 25074 | Hetero | 17 | 40 | 42.5 | 0.41 | 0 | 34 | - | CDS1 |
| 4 | 85591953 | SNV | A | G | 1 | 25074 | Hetero | 44 | 67 | 65.7 | 0.45 | 0 | 49 | - | WDFY3 |
| 4 | 87593550 | SNV | A | G | 1 | 25074 | Hetero | 78 | 140 | 55.7 | 0.35 | 0 | 99 | Yes | PTPN13 |
| 4 | 88813601 | SNV | A | T | 1 | 25074 | Hetero | 58 | 133 | 43.6 | 0.35 | 0 | 142 | - | ENSG00000183199 |
| 4 | 89660139 | SNV | C | T | 1 | 25074 | Hetero | 49 | 126 | 38.9 | 0.46 | 0 | 100 | - | FAM13A |
| 4 | 94695216 | SNV | G | C | 1 | 25074 | Hetero | 32 | 81 | 39.5 | 0.42 | 0 | 74 | - | GRID2 |
| 4 | 96025637 | SNV | C | T | 1 | 25074 | Hetero | 166 | 288 | 57.6 | 0.49 | 0 | 277 | Yes | BMPR1B |
| 4 | 96087646 | SNV | C | T | 1 | 25074 | Hetero | 43 | 66 | 65.2 | 0.46 | 0 | 47 | - | UNC5C |
| 4 | 103173457 | SNV | G | A | 1 | 25074 | Hetero | 20 | 71 | 28.2 | 0.50 | 0 | 43 | - | SLC39A8 |
| 4 | 104007643 | SNV | C | T | 1 | 25074 | Hetero | 28 | 72 | 38.9 | 0.48 | 0 | 60 | Yes | BDH2 |
| 4 | 104060934 | SNV | C | T | 1 | 25074 | Hetero | 20 | 49 | 40.8 | 0.13 | 0 | 30 | - | CENPE |
| 4 | 106603861 | SNV | A | G | 1 | 25074 | Hetero | 108 | 260 | 41.5 | 0.25 | 0 | 239 | - | ARHGEF38 |
| 4 | 110608696 | SNV | G | A | 1 | 25074 | Hetero | 136 | 351 | 38.7 | 0.45 | 0 | 269 | Yes | CCDC109B |
| 4 | 110773163 | SNV | T | C | 1 | 25074 | Hetero | 87 | 127 | 68.5 | 0.25 | 0 | 101 | - | LRIT3 |
| 4 | 113569492 | SNV | A | G | 1 | 25074 | Hetero | 39 | 120 | 32.5 | 0.48 | 0 | 96 | - | LARP7 |
| 4 | 114275397 | SNV | A | C | 1 | 25074 | Hetero | 229 | 394 | 58.1 | 0.39 | 0 | 354 | Yes | ANK2 |
| 4 | 120221784 | SNV | G | A | 1 | 25074 | Hetero | 224 | 362 | 61.9 | 0.40 | 0 | 282 | No | C4orf3 |
| 4 | 120239127 | SNV | G | A | 1 | 25074 | Hetero | 19 | 72 | 26.4 | 0.33 | 0 | 83 | - | FABP2 |
| 4 | 122722674 | SNV | G | T | 1 | 25074 | Hetero | 155 | 249 | 62.2 | 0.37 | 0 | 215 | - | EXOSC9 |
| 4 | 126240485 | SNV | C | T | 1 | 25074 | Hetero | 56 | 148 | 37.8 | 0.31 | 0 | 153 | No | FAT4 |
| 4 | 126241690 | SNV | C | A | 1 | 25074 | Hetero | 110 | 219 | 50.2 | 0.44 | 0 | 168 | Yes | FAT4 |
| 4 | 128554293 | SNV | T | C | 1 | 25074 | Hetero | 45 | 106 | 42.5 | 0.42 | 0 | 86 | Yes | INTU |
| 4 | 129190985 | SNV | T | G | 1 | 25074 | Hetero | 44 | 64 | 68.8 | 0.46 | 0 | 45 | - | PGRMC2 |
| 4 | 129958631 | SNV | G | A | 1 | 25074 | Hetero | 51 | 74 | 68.9 | 0.47 | 0 | 80 | - | SCLT1 |
| 4 | 129959011 | SNV | C | T | 1 | 25074 | Hetero | 18 | 64 | 28.1 | 0.20 | 0 | 50 | - | SCLT1 |
| 4 | 144477942 | SNV | C | T | 1 | 25074 | Hetero | 43 | 65 | 66.2 | 0.44 | 0 | 61 | - | SMARCA5 |
| 4 | 146046303 | SNV | C | T | 1 | 25074 | Hetero | 40 | 65 | 61.5 | 0.42 | 0 | 48 | - | ABCE1 |
| 4 | 146653534 | SNV | A | C | 1 | 25074 | Hetero | 28 | 77 | 36.4 | 0.43 | 0 | 73 | - | C4orf51 |
| 4 | 146791392 | SNV | A | T | 1 | 25074 | Hetero | 52 | 92 | 56.5 | 0.22 | 0 | 65 | - | ZNF827 |
| 4 | 151186932 | SNV | A | G | 1 | 25074 | Hetero | 85 | 236 | 36.0 | 0.31 | 0 | 184 | Yes | LRBA |
| 4 | 153897081 | SNV | G | A | 1 | 25074 | Hetero | 32 | 210 | 15.2 | 0.49 | 0 | 163 | Yes | FHDC1 |
| 4 | 154336193 | SNV | C | T | 1 | 25074 | Hetero | 40 | 99 | 40.4 | 0.44 | 0 | 74 | - | MND1 |
| 4 | 164534736 | SNV | A | G | 1 | 25074 | Hetero | 49 | 210 | 23.3 | 0.35 | 0 | 191 | - | MARCH1 |
| 4 | 165999216 | SNV | A | G | 1 | 25074 | Hetero | 8 | 34 | 23.5 | 0.45 | 0 | 33 | - | TMEM192 |
| 4 | 165999799 | SNV | G | C | 1 | 25074 | Hetero | 12 | 36 | 33.3 | 0.46 | 0 | 34 | - | TMEM192 |
| 4 | 174458070 | SNV | G | A | 1 | 25074 | Hetero | 47 | 172 | 27.3 | 0.31 | 0 | 166 | - | RP11-471J12.1 |
| 4 | 183694809 | SNV | G | A | 1 | 25074 | Hetero | 84 | 126 | 66.7 | 0.20 | 0 | 98 | - | ODZ3 |
| 4 | 184237195 | SNV | A | T | 1 | 25074 | Hetero | 31 | 49 | 63.3 | 0.46 | 0 | 49 | - | WWC2 |
| 4 | 185678840 | SNV | G | A | 1 | 25074 | Hetero | 54 | 82 | 65.9 | 0.20 | 0 | 66 | No | ACSL1 |
| 4 | 186081250 | Deletion | A | - | 1 | 25074 | Hetero | 44 | 64 | 68.8 | 0.22 | 0 | 68 | - | KIAA1430 |
| 4 | 186287234 | SNV | T | A | 1 | 25074 | Hetero | 42 | 71 | 59.2 | 0.37 | 0 | 53 | - | SNX25 |
| 4 | 188926025 | SNV | A | G | 1 | 25074 | Hetero | 12 | 44 | 27.3 | 0.37 | 0 | 50 | - | ZFP42 |
| 4 | 189060983 | SNV | G | C | 1 | 25074 | Hetero | 75 | 125 | 60.0 | 0.43 | 0 | 103 | Yes | TRIML1 |
| 4 | 110635488 | Insertion | - | T | 1 | 25074 | Hetero | 14 | 36 | 38.9 | 0.36 | 0 | 40 | - | PLA2G12A |
| 4 | 114900260 | Insertion | - | A | 1 | 25074 | Hetero | 92 | 141 | 65.2 | 0.42 | 0 | 136 | - | ARSJ |
| 4 | 122800387 | Deletion | AA | - | 2 | 25074 | Hetero | 17 | 104 | 16.3 | 0.45 | 0 | 74 | - | TRPC3 |
| 4 | 129784049 | Insertion | - | TG | 2 | 25074 | Hetero | 55 | 86 | 64.0 | 0.44 | 0 | 56 | - | PHF17 |
| 4 | 140340060 | Insertion | - | TT | 2 | 25074 | Hetero | 37 | 86 | 43.0 | 0.42 | 0 | 47 | - | NAA15 |
| 4 | 144475127 | Insertion | - | T | 1 | 25074 | Hetero | 47 | 73 | 64.4 | 0.35 | 0 | 43 | - | SMARCA5 |
| 4 | 15444979 | Deletion | TG | - | 2 | 25074 | Hetero | 53 | 91 | 58.2 | 0.47 | 0 | 91 | - | C1QTNF7 |
| 4 | 162463660 | Insertion | - | A | 1 | 25074 | Hetero | 30 | 74 | 40.5 | 0.21 | 0 | 74 | - | FSTL5 |
| 4 | 165998594 | Insertion | - | A | 1 | 25074 | Hetero | 23 | 58 | 39.7 | 0.16 | 0 | 39 | - | TMEM192 |
| 4 | 169337957 | Insertion | - | A | 1 | 25074 | Hetero | 31 | 87 | 35.6 | 0.06 | 0 | 58 | - | DDX60L |
| 4 | 186081821 | Deletion | CT | - | 2 | 25074 | Hetero | 34 | 62 | 54.8 | 0.33 | 0 | 89 | - | KIAA1430 |
| 4 | 186083620 | Insertion | - | TA | 2 | 25074 | Hetero | 52 | 79 | 65.8 | 0.43 | 0 | 72 | - | KIAA1430 |
| 4 | 20733708 | Insertion | - | A | 1 | 25074 | Hetero | 13 | 38 | 34.2 | 0.50 | 0 | 36 | - | PACRGL |
| 4 | 2272083 | Insertion | - | G | 1 | 25074 | Hetero | 109 | 182 | 59.9 | 0.45 | 0 | 122 | - | ZFYVE28 |
| 4 | 25003867 | Insertion | - | GG | 2 | 25074 | Homo | 99 | 100 | 99.0 | 0.45 | 0 | 85 | - | LGI2 |
| 4 | 368452 | Insertion | - | AA | 2 | 25074 | Hetero | 12 | 40 | 30.0 | 0.46 | 0 | 54 | - | ZNF141 |
| 4 | 44708325 | Insertion | - | TT | 2 | 25074 | Hetero | 15 | 52 | 28.8 | 0.47 | 0 | 59 | - | GNPDA2 |
| 4 | 48137977 | Insertion | - | T | 1 | 25074 | Homo | 109 | 109 | 100.0 | 0.29 | 0 | 91 | - | TEC |
| 4 | 671895 | Insertion | - | AA | 2 | 25074 | Hetero | 57 | 74 | 77.0 | 0.28 | 0 | 34 | - | MYL5 |
| 4 | 7743575 | Insertion | - | G | 1 | 25074 | Hetero | 57 | 167 | 34.1 | 0.43 | 0 | 100 | - | SORCS2 |
| 5 | 191923 | SNV | G | A | 1 | 25074 | Hetero | 35 | 65 | 53.8 | 0.22 | 0 | 64 | No | LRRC14B |
| 5 | 635553 | SNV | G | A | 1 | 25074 | Hetero | 81 | 193 | 42.0 | 0.48 | 0 | 89 | Yes | CEP72 |
| 5 | 661419 | SNV | G | C | 1 | 25074 | Hetero | 59 | 554 | 10.6 | 0.35 | 0 | 312 | - | CEP72 |
| 5 | 15500614 | SNV | A | T | 1 | 25074 | Hetero | 29 | 51 | 56.9 | 0.32 | 0 | 48 | - | FBXL7 |
| 5 | 31322968 | SNV | A | G | 1 | 25074 | Hetero | 119 | 244 | 48.8 | 0.39 | 0 | 161 | No | CDH6 |
| 5 | 38462977 | SNV | C | T | 1 | 25074 | Hetero | 13 | 61 | 21.3 | 0.23 | 0 | 34 | - | EGFLAM |
| 5 | 38886751 | SNV | T | G | 1 | 25074 | Hetero | 17 | 48 | 35.4 | 0.37 | 0 | 38 | - | OSMR |
| 5 | 42629217 | SNV | A | G | 1 | 25074 | Hetero | 55 | 102 | 53.9 | 0.25 | 0 | 61 | - | GHR |
| 5 | 56191200 | SNV | T | A | 1 | 25074 | Hetero | 46 | 88 | 52.3 | 0.25 | 0 | 64 | - | MAP3K1 |
| 5 | 61745736 | SNV | A | G | 1 | 25074 | Hetero | 87 | 171 | 50.9 | 0.20 | 0 | 111 | - | KIF2A |
| 5 | 66492307 | SNV | G | C | 1 | 25074 | Hetero | 76 | 153 | 49.7 | 0.21 | 0 | 100 | - | CD180 |
| 5 | 74715257 | SNV | A | G | 1 | 25074 | Hetero | 32 | 82 | 39.0 | 0.11 | 0 | 46 | - | COL4A3BP |
| 5 | 76028371 | SNV | T | C | 1 | 25074 | Hetero | 139 | 276 | 50.4 | 0.50 | 0 | 172 | No | F2R |
| 5 | 82833627 | SNV | C | A | 1 | 25074 | Hetero | 77 | 187 | 41.2 | 0.36 | 0 | 148 | Yes | VCAN |
| 5 | 83680361 | SNV | T | C | 1 | 25074 | Hetero | 68 | 190 | 35.8 | 0.42 | 0 | 170 | - | EDIL3 |
| 5 | 90106729 | SNV | G | A | 1 | 25074 | Hetero | 104 | 215 | 48.4 | 0.39 | 0 | 154 | Yes | GPR98 |
| 5 | 94041213 | Deletion | A | - | 1 | 25074 | Hetero | 8 | 76 | 10.5 | 0.25 | 0 | 56 | - | ANKRD32 |
| 5 | 106715354 | SNV | A | C | 1 | 25074 | Hetero | 63 | 138 | 45.7 | 0.45 | 0 | 114 | - | EFNA5 |
| 5 | 112204434 | SNV | A | G | 1 | 25074 | Hetero | 28 | 58 | 48.3 | 0.39 | 0 | 31 | - | SRP19 |
| 5 | 112227541 | SNV | T | C | 1 | 25074 | Hetero | 66 | 150 | 44.0 | 0.28 | 0 | 97 | No | SRP19 |
| 5 | 112355824 | SNV | A | G | 1 | 25074 | Hetero | 62 | 130 | 47.7 | 0.49 | 0 | 86 | - | DCP2 |
| 5 | 114952140 | SNV | C | T | 1 | 25074 | Hetero | 81 | 217 | 37.3 | 0.18 | 0 | 139 | Yes | TICAM2 |
| 5 | 130721345 | SNV | T | C | 1 | 25074 | Hetero | 200 | 395 | 50.6 | 0.26 | 0 | 284 | - | CDC42SE2 |
| 5 | 133746842 | SNV | A | G | 1 | 25074 | Hetero | 71 | 154 | 46.1 | 0.28 | 0 | 92 | - | CDKN2AIPNL |
| 5 | 133917070 | SNV | A | C | 1 | 25074 | Hetero | 84 | 154 | 54.5 | 0.35 | 0 | 94 | - | PHF15 |
| 5 | 137492707 | SNV | C | A | 1 | 25074 | Hetero | 74 | 159 | 46.5 | 0.19 | 0 | 111 | - | BRD8 |
| 5 | 138208377 | SNV | A | T | 1 | 25074 | Hetero | 38 | 57 | 66.7 | 0.35 | 0 | 41 | - | CTNNA1 |
| 5 | 139201406 | SNV | G | C | 1 | 25074 | Hetero | 23 | 44 | 52.3 | 0.28 | 0 | 32 | - | PSD2 |
| 5 | 139928657 | SNV | A | G | 1 | 25074 | Hetero | 95 | 218 | 43.6 | 0.48 | 0 | 137 | Yes | ANKHD1 |
| 5 | 140011478 | SNV | G | A | 1 | 25074 | Hetero | 131 | 383 | 34.2 | 0.40 | 0 | 233 | Yes | CD14 |
| 5 | 140236426 | SNV | G | C | 1 | 25074 | Hetero | 165 | 355 | 46.5 | 0.47 | 0 | 250 | Yes | PCDHA1 |
| 5 | 140504742 | SNV | C | T | 1 | 25074 | Hetero | 55 | 134 | 41.0 | 0.23 | 0 | 120 | - | PCDHB4 |
| 5 | 145893630 | Deletion | A | - | 1 | 25074 | Hetero | 83 | 141 | 58.9 | 0.44 | 0 | 104 | - | GPR151 |
| 5 | 146730633 | SNV | G | T | 1 | 25074 | Hetero | 46 | 141 | 32.6 | 0.34 | 0 | 78 | Yes | STK32A |
| 5 | 148384123 | SNV | C | G | 1 | 25074 | Hetero | 39 | 119 | 32.8 | 0.48 | 0 | 93 | - | SH3TC2 |
| 5 | 148431701 | SNV | A | G | 1 | 25074 | Hetero | 127 | 266 | 47.7 | 0.49 | 0 | 184 | - | SH3TC2 |
| 5 | 148625972 | SNV | T | A | 1 | 25074 | Hetero | 82 | 196 | 41.8 | 0.25 | 0 | 97 | - | ABLIM3 |
| 5 | 148999940 | SNV | C | T | 1 | 25074 | Hetero | 43 | 84 | 51.2 | 0.20 | 0 | 59 | No | ARHGEF37 |
| 5 | 149375966 | SNV | G | A | 1 | 25074 | Hetero | 131 | 304 | 43.1 | 0.50 | 0 | 181 | - | TIGD6 |
| 5 | 156479568 | SNV | C | T | 1 | 25074 | Hetero | 32 | 303 | 10.6 | 0.30 | 0 | 247 | No | HAVCR1 |
| 5 | 158588391 | SNV | C | T | 1 | 25074 | Hetero | 47 | 98 | 48.0 | 0.49 | 0 | 57 | No | RNF145 |
| 5 | 170597210 | SNV | G | C | 1 | 25074 | Hetero | 5 | 43 | 11.6 | 0.43 | 0 | 34 | - | RANBP17 |
| 5 | 171762207 | SNV | T | C | 1 | 25074 | Hetero | 49 | 104 | 47.1 | 0.39 | 0 | 69 | - | SH3PXD2B |
| 5 | 172660329 | SNV | G | T | 1 | 25074 | Hetero | 132 | 281 | 47.0 | 0.47 | 0 | 149 | - | NKX2-5 |
| 5 | 176563847 | SNV | A | C | 1 | 25074 | Hetero | 107 | 219 | 48.9 | 0.18 | 0 | 116 | - | NSD1 |
| 5 | 176931224 | SNV | C | T | 1 | 25074 | Hetero | 32 | 181 | 17.7 | 0.44 | 0 | 89 | No | DOK3 |
| 5 | 179157298 | SNV | T | G | 1 | 25074 | Hetero | 70 | 143 | 49.0 | 0.48 | 0 | 99 | - | CANX |
| 5 | 131792639 | Insertion | - | T | 1 | 25074 | Hetero | 53 | 115 | 46.1 | 0.11 | 0 | 62 | - | C5orf56 |
| 5 | 38476624 | Insertion | - | T | 1 | 25074 | Hetero | 39 | 107 | 36.4 | 0.45 | 0 | 87 | - | LIFR |
| 5 | 50690278 | Insertion | - | AA | 2 | 25074 | Hetero | 39 | 134 | 29.1 | 0.49 | 0 | 109 | - | ISL1 |
| 5 | 56191192 | Deletion | TG | - | 2 | 25074 | Hetero | 57 | 96 | 59.4 | 0.25 | 0 | 65 | - | MAP3K1 |
| 5 | 78281383 | Insertion | - | A | 1 | 25074 | Homo | 96 | 100 | 96.0 | 0.47 | 0 | 58 | - | ARSB |
| 5 | 94749903 | Insertion | - | T | 1 | 25074 | Hetero | 97 | 222 | 43.7 | 0.21 | 0 | 137 | - | FAM81B |
| 5 | 94993645 | Insertion | - | TG | 2 | 25074 | Hetero | 24 | 51 | 47.1 | 0.40 | 0 | 36 | - | RFESD |
| 5 | 96271602 | Insertion | - | C | 1 | 25074 | Homo | 275 | 275 | 100.0 | 0.49 | 0 | 175 | - | LNPEP |
| 6 | 408257 | SNV | G | T | 1 | 25074 | Hetero | 80 | 197 | 40.6 | 0.26 | 0 | 162 | - | IRF4 |
| 6 | 410336 | SNV | C | A | 1 | 25074 | Hetero | 46 | 142 | 32.4 | 0.43 | 0 | 143 | - | IRF4 |
| 6 | 11578023 | SNV | C | A | 1 | 25074 | Hetero | 67 | 116 | 57.8 | 0.33 | 0 | 97 | - | TMEM170B |
| 6 | 11770335 | Deletion | A | - | 1 | 25074 | Hetero | 65 | 91 | 71.4 | 0.22 | 0 | 58 | - | ADTRP |
| 6 | 24450035 | SNV | G | A | 1 | 25074 | Hetero | 111 | 184 | 60.3 | 0.39 | 0 | 131 | No | GPLD1 |
| 6 | 28294500 | SNV | G | A | 1 | 25074 | Hetero | 73 | 130 | 56.2 | 0.49 | 0 | 76 | Yes | ZNF323 |
| 6 | 30673177 | SNV | A | G | 1 | 25074 | Hetero | 33 | 237 | 13.9 | 0.37 | 0 | 207 | No | MDC1 |
| 6 | 32630099 | SNV | G | T | 1 | 25074 | Hetero | 19 | 54 | 35.2 | 0.25 | 0 | 32 | - | HLA-DQB1 |
| 6 | 32630101 | SNV | A | G | 1 | 25074 | Hetero | 20 | 54 | 37.0 | 0.24 | 0 | 30 | - | HLA-DQB1 |
| 6 | 41035090 | SNV | G | C | 1 | 25074 | Hetero | 145 | 317 | 45.7 | 0.43 | 0 | 265 | Yes | C6orf130 |
| 6 | 41158079 | SNV | G | A | 1 | 25074 | Hetero | 31 | 83 | 37.3 | 0.39 | 0 | 64 | - | TREML2 |
| 6 | 42644597 | SNV | C | A | 1 | 25074 | Hetero | 52 | 90 | 57.8 | 0.38 | 0 | 40 | Yes | UBR2 |
| 6 | 42946479 | SNV | C | T | 1 | 25074 | Hetero | 41 | 61 | 67.2 | 0.24 | 0 | 50 | Yes | PEX6 |
| 6 | 43006088 | SNV | G | A | 1 | 25074 | Hetero | 126 | 223 | 56.5 | 0.44 | 0 | 134 | Yes | CUL7 |
| 6 | 44201182 | SNV | G | A | 1 | 25074 | Hetero | 117 | 185 | 63.2 | 0.15 | 0 | 133 | Yes | SLC29A1 |
| 6 | 46658695 | SNV | T | C | 1 | 25074 | Hetero | 132 | 217 | 60.8 | 0.41 | 0 | 154 | Yes | TDRD6 |
| 6 | 53659794 | SNV | G | T | 1 | 25074 | Hetero | 31 | 78 | 39.7 | 0.06 | 0 | 49 | - | LRRC1 |
| 6 | 56472545 | SNV | T | C | 1 | 25074 | Hetero | 57 | 105 | 54.3 | 0.25 | 0 | 110 | Yes | DST |
| 6 | 57244834 | SNV | T | A | 1 | 25074 | Hetero | 14 | 59 | 23.7 | 0.07 | 0 | 45 | - | PRIM2 |
| 6 | 57372365 | SNV | A | G | 1 | 25074 | Hetero | 74 | 300 | 24.7 | 0.16 | 0 | 245 | - | PRIM2 |
| 6 | 71270849 | SNV | C | T | 1 | 25074 | Hetero | 23 | 49 | 46.9 | 0.30 | 0 | 43 | - | FAM135A |
| 6 | 74078336 | SNV | C | G | 1 | 25074 | Hetero | 38 | 79 | 48.1 | 0.49 | 0 | 81 | - | OOEP |
| 6 | 78172581 | SNV | G | T | 1 | 25074 | Hetero | 102 | 249 | 41.0 | 0.47 | 0 | 187 | No | HTR1B |
| 6 | 86299303 | SNV | T | C | 1 | 25074 | Hetero | 121 | 236 | 51.3 | 0.42 | 0 | 167 | - | SNX14 |
| 6 | 106968837 | SNV | C | A | 1 | 25074 | Hetero | 161 | 346 | 46.5 | 0.47 | 0 | 295 | Yes | AIM1 |
| 6 | 114181577 | SNV | C | T | 1 | 25074 | Homo | 52 | 52 | 100.0 | 0.08 | 0 | 41 | Yes | MARCKS |
| 6 | 114378808 | SNV | A | G | 1 | 25074 | Hetero | 184 | 364 | 50.5 | 0.44 | 0 | 227 | No | RP3-399L15.3 |
| 6 | 119191384 | SNV | C | T | 1 | 25074 | Hetero | 33 | 63 | 52.4 | 0.49 | 0 | 44 | - | MCM9 |
| 6 | 119326524 | SNV | A | T | 1 | 25074 | Hetero | 38 | 80 | 47.5 | 0.28 | 0 | 30 | - | FAM184A |
| 6 | 125412462 | SNV | G | A | 1 | 25074 | Hetero | 54 | 98 | 55.1 | 0.41 | 0 | 77 | - | RNF217 |
| 6 | 130764061 | SNV | G | A | 1 | 25074 | Hetero | 52 | 100 | 52.0 | 0.30 | 0 | 64 | - | TMEM200A |
| 6 | 132860063 | SNV | C | T | 1 | 25074 | Hetero | 72 | 168 | 42.9 | 0.34 | 0 | 99 | Yes | TAAR9 |
| 6 | 133078915 | SNV | A | T | 1 | 25074 | Hetero | 192 | 428 | 44.9 | 0.37 | 0 | 306 | No | VNN2 |
| 6 | 133783458 | SNV | T | C | 1 | 25074 | Hetero | 72 | 151 | 47.7 | 0.15 | 0 | 96 | - | EYA4 |
| 6 | 134305844 | SNV | A | G | 1 | 25074 | Hetero | 136 | 278 | 48.9 | 0.43 | 0 | 215 | - | TBPL1 |
| 6 | 135606703 | SNV | G | A | 1 | 25074 | Hetero | 132 | 268 | 49.3 | 0.43 | 0 | 180 | - | AHI1 |
| 6 | 138645332 | SNV | C | T | 1 | 25074 | Hetero | 49 | 117 | 41.9 | 0.35 | 0 | 78 | - | KIAA1244 |
| 6 | 139145176 | SNV | A | G | 1 | 25074 | Homo | 139 | 140 | 99.3 | 0.16 | 0 | 169 | - | ECT2L |
| 6 | 139309269 | SNV | T | C | 1 | 25074 | Hetero | 53 | 119 | 44.5 | 0.36 | 0 | 73 | - | REPS1 |
| 6 | 139500306 | SNV | A | G | 1 | 25074 | Hetero | 82 | 153 | 53.6 | 0.46 | 0 | 84 | - | HECA |
| 6 | 152422459 | SNV | T | C | 1 | 25074 | Hetero | 32 | 74 | 43.2 | 0.29 | 0 | 39 | - | ESR1 |
| 6 | 159054175 | Deletion | A | - | 1 | 25074 | Hetero | 52 | 118 | 44.1 | 0.26 | 0 | 85 | - | TMEM181 |
| 6 | 166721220 | SNV | C | T | 1 | 25074 | Hetero | 74 | 160 | 46.3 | 0.43 | 0 | 87 | No | PRR18 |
| 6 | 167446051 | SNV | C | T | 1 | 25074 | Hetero | 11 | 62 | 17.7 | 0.18 | 0 | 37 | - | FGFR1OP |
| 6 | 167550069 | SNV | G | A | 1 | 25074 | Hetero | 55 | 99 | 55.6 | 0.25 | 0 | 78 | No | CCR6 |
| 6 | 167584309 | SNV | G | A | 1 | 25074 | Hetero | 20 | 125 | 16.0 | 0.13 | 0 | 132 | - | TCP10L2 |
| 6 | 167786439 | SNV | C | T | 1 | 25074 | Hetero | 54 | 175 | 30.9 | 0.26 | 0 | 330 | - | TCP10 |
| 6 | 167786554 | SNV | T | C | 1 | 25074 | Hetero | 108 | 506 | 21.3 | 0.34 | 0 | 478 | - | TCP10 |
| 6 | 105300262 | Insertion | - | AG | 2 | 25074 | Hetero | 66 | 155 | 42.6 | 0.27 | 0 | 124 | - | HACE1 |
| 6 | 11714197 | Insertion | - | A | 1 | 25074 | Hetero | 42 | 108 | 38.9 | 0.26 | 0 | 74 | - | ADTRP |
| 6 | 121402008 | Deletion | CT | - | 2 | 25074 | Hetero | 138 | 262 | 52.7 | 0.24 | 0 | 189 | Deletion | C6orf170 |
| 6 | 130764059 | Insertion | - | A | 1 | 25074 | Hetero | 54 | 102 | 52.9 | 0.31 | 0 | 65 | - | TMEM200A |
| 6 | 139013283 | Insertion | - | C | 1 | 25074 | Hetero | 121 | 287 | 42.2 | 0.50 | 0 | 179 | - | NHSL1 |
| 6 | 158098882 | Insertion | - | G | 1 | 25074 | Hetero | 17 | 72 | 23.6 | 0.22 | 0 | 60 | - | ZDHHC14 |
| 6 | 168372109 | Insertion | - | T | 1 | 25074 | Hetero | 37 | 75 | 49.3 | 0.46 | 0 | 59 | - | MLLT4 |
| 6 | 18215166 | Insertion | - | A | 1 | 25074 | Hetero | 91 | 133 | 68.4 | 0.28 | 0 | 116 | - | KDM1B |
| 6 | 24705301 | Insertion | - | A | 1 | 25074 | Hetero | 19 | 52 | 36.5 | 0.37 | 0 | 39 | - | C6orf62 |
| 6 | 24804674 | Insertion | - | A | 1 | 25074 | Homo | 86 | 86 | 100.0 | 0.50 | 0 | 54 | - | FAM65B |
| 6 | 36789940 | Insertion | - | G | 1 | 25074 | Hetero | 38 | 143 | 26.6 | 0.37 | 0 | 79 | - | PI16 |
| 6 | 39313618 | Insertion | - | A | 1 | 25074 | Hetero | 59 | 96 | 61.5 | 0.36 | 0 | 72 | - | KIF6 |
| 6 | 52374319 | Insertion | - | AC | 2 | 25074 | Homo | 91 | 91 | 100.0 | 0.15 | 0 | 74 | - | EFHC1 |
| 6 | 53133217 | Insertion | - | C | 1 | 25074 | Hetero | 37 | 101 | 36.6 | 0.43 | 0 | 82 | - | ELOVL5 |
| 6 | 74214904 | Insertion | - | A | 1 | 25074 | Hetero | 22 | 58 | 37.9 | 0.20 | 0 | 43 | - | MTO1 |
| 6 | 89868190 | Insertion | - | T | 1 | 25074 | Hetero | 32 | 74 | 43.2 | 0.39 | 0 | 42 | - | PM20D2 |
| 6 | 91225340 | Insertion | - | T | 1 | 25074 | Hetero | 35 | 82 | 42.7 | 0.28 | 0 | 62 | - | MAP3K7 |
| 6 | 97593972 | Insertion | - | T | 1 | 25074 | Hetero | 49 | 130 | 37.7 | 0.48 | 0 | 94 | - | MMS22L |
| 7 | 731500 | SNV | G | A | 1 | 25074 | Hetero | 95 | 119 | 79.8 | 0.36 | 0 | 108 | - | PRKAR1B |
| 7 | 1589138 | SNV | C | T | 1 | 25074 | Hetero | 18 | 71 | 25.4 | 0.40 | 0 | 58 | - | TMEM184A |
| 7 | 2472939 | SNV | G | T | 1 | 25074 | Hetero | 194 | 230 | 84.3 | 0.44 | 0 | 214 | Yes | CHST12 |
| 7 | 3997566 | SNV | G | A | 1 | 25074 | Hetero | 80 | 101 | 79.2 | 0.31 | 0 | 113 | - | SDK1 |
| 7 | 5161640 | SNV | G | C | 1 | 25074 | Hetero | 40 | 51 | 78.4 | 0.41 | 0 | 111 | - | ZNF890P |
| 7 | 5385392 | SNV | G | A | 1 | 25074 | Hetero | 83 | 101 | 82.2 | 0.45 | 0 | 75 | No | TNRC18 |
| 7 | 13931193 | SNV | A | T | 1 | 25074 | Hetero | 44 | 58 | 75.9 | 0.45 | 0 | 96 | - | ETV1 |
| 7 | 16684398 | SNV | G | C | 1 | 25074 | Hetero | 37 | 51 | 72.5 | 0.15 | 0 | 60 | Yes | ANKMY2 |
| 7 | 20198606 | SNV | A | G | 1 | 25074 | Hetero | 73 | 94 | 77.7 | 0.43 | 0 | 92 | No | MACC1 |
| 7 | 25161698 | Deletion | G | - | 1 | 25074 | Hetero | 48 | 56 | 85.7 | 0.46 | 0 | 63 | - | CYCS |
| 7 | 26574393 | SNV | G | C | 1 | 25074 | Hetero | 15 | 122 | 12.3 | 0.47 | 0 | 127 | - | KIAA0087 |
| 7 | 27132968 | SNV | C | T | 1 | 25074 | Hetero | 15 | 72 | 20.8 | 0.44 | 0 | 77 | - | HOXA1 |
| 7 | 27285914 | SNV | C | T | 1 | 25074 | Hetero | 70 | 90 | 77.8 | 0.44 | 0 | 65 | Yes | EVX1 |
| 7 | 30634582 | SNV | T | G | 1 | 25074 | Hetero | 53 | 66 | 80.3 | 0.40 | 0 | 58 | No | GARS |
| 7 | 31913075 | SNV | C | T | 1 | 25074 | Hetero | 41 | 49 | 83.7 | 0.16 | 0 | 84 | - | PDE1C |
| 7 | 33003171 | SNV | G | A | 1 | 25074 | Hetero | 30 | 41 | 73.2 | 0.34 | 0 | 60 | No | AVL9 |
| 7 | 34193636 | SNV | A | C | 1 | 25074 | Hetero | 49 | 64 | 76.6 | 0.41 | 0 | 72 | - | BMPER |
| 7 | 35734644 | SNV | G | A | 1 | 25074 | Hetero | 63 | 78 | 80.8 | 0.48 | 0 | 73 | - | HERPUD2 |
| 7 | 36194076 | SNV | A | G | 1 | 25074 | Hetero | 164 | 200 | 82.0 | 0.47 | 0 | 203 | Yes | EEPD1 |
| 7 | 38466459 | SNV | T | C | 1 | 25074 | Hetero | 9 | 36 | 25.0 | 0.20 | 0 | 32 | - | AMPH |
| 7 | 38948897 | SNV | G | A | 1 | 25074 | Hetero | 139 | 164 | 84.8 | 0.22 | 0 | 177 | - | VPS41 |
| 7 | 38948902 | SNV | G | A | 1 | 25074 | Hetero | 132 | 155 | 85.2 | 0.19 | 0 | 168 | - | VPS41 |
| 7 | 42004038 | SNV | G | C | 1 | 25074 | Hetero | 217 | 256 | 84.8 | 0.38 | 0 | 271 | Yes | GLI3 |
| 7 | 47852787 | SNV | G | A | 1 | 25074 | Hetero | 29 | 37 | 78.4 | 0.38 | 0 | 46 | No | HUS1 |
| 7 | 48273693 | SNV | G | T | 1 | 25074 | Hetero | 84 | 111 | 75.7 | 0.47 | 0 | 87 | Yes | ABCA13 |
| 7 | 50611828 | SNV | A | G | 1 | 25074 | Hetero | 33 | 189 | 17.5 | 0.48 | 0 | 217 | - | DDC |
| 7 | 50671954 | SNV | G | C | 1 | 25074 | Hetero | 6 | 31 | 19.4 | 0.38 | 0 | 36 | - | GRB10 |
| 7 | 63355397 | SNV | A | T | 1 | 25074 | Homo | 48 | 48 | 100.0 | 0.15 | 0 | 206 | - | RP11-340I6.7 |
| 7 | 72413593 | SNV | T | C | 1 | 25074 | Hetero | 37 | 147 | 25.2 | 0.18 | 0 | 148 | Yes | POM121 |
| 7 | 72727091 | SNV | G | A | 1 | 25074 | Hetero | 76 | 203 | 37.4 | 0.47 | 0 | 119 | No | TRIM50 |
| 7 | 73112467 | SNV | G | A | 1 | 25074 | Hetero | 20 | 55 | 36.4 | 0.28 | 0 | 35 | - | WBSCR22 |
| 7 | 73254460 | Deletion | T | - | 1 | 25074 | Hetero | 8 | 72 | 11.1 | 0.25 | 0 | 79 | - | WBSCR27 |
| 7 | 74119580 | SNV | A | T | 1 | 25074 | Hetero | 73 | 124 | 58.9 | 0.38 | 0 | 98 | - | GTF2I |
| 7 | 76069902 | SNV | G | C | 1 | 25074 | Hetero | 30 | 91 | 33.0 | 0.45 | 0 | 109 | Yes | ZP3 |
| 7 | 76713304 | SNV | T | G | 1 | 25074 | Hetero | 31 | 92 | 33.7 | 0.09 | 0 | 100 | - | FAM185BP |
| 7 | 80456848 | SNV | T | C | 1 | 25074 | Hetero | 33 | 50 | 66.0 | 0.18 | 0 | 30 | - | SEMA3C |
| 7 | 82993672 | SNV | A | T | 1 | 25074 | Hetero | 27 | 69 | 39.1 | 0.37 | 0 | 61 | - | SEMA3E |
| 7 | 83037663 | SNV | T | C | 1 | 25074 | Hetero | 55 | 111 | 49.5 | 0.35 | 0 | 81 | - | SEMA3E |
| 7 | 86688409 | SNV | A | G | 1 | 25074 | Hetero | 212 | 320 | 66.3 | 0.42 | 0 | 248 | - | KIAA1324L |
| 7 | 88965755 | SNV | C | T | 1 | 25074 | Hetero | 101 | 160 | 63.1 | 0.43 | 0 | 159 | No | ZNF804B |
| 7 | 89938617 | SNV | G | A | 1 | 25074 | Hetero | 24 | 59 | 40.7 | 0.37 | 0 | 36 | Yes | C7orf63 |
| 7 | 89976015 | SNV | T | G | 1 | 25074 | Hetero | 79 | 201 | 39.3 | 0.15 | 0 | 160 | - | GTPBP10 |
| 7 | 90043259 | SNV | C | G | 1 | 25074 | Hetero | 51 | 102 | 50.0 | 0.46 | 0 | 99 | - | CLDN12 |
| 7 | 91793485 | SNV | T | G | 1 | 25074 | Hetero | 16 | 85 | 18.8 | 0.31 | 0 | 50 | Yes | LRRD1 |
| 7 | 94058671 | SNV | T | C | 1 | 25074 | Hetero | 64 | 93 | 68.8 | 0.48 | 0 | 101 | Yes | COL1A2 |
| 7 | 99720174 | SNV | C | T | 1 | 25074 | Hetero | 67 | 140 | 47.9 | 0.38 | 0 | 105 | Yes | RP11-506M12.1 |
| 7 | 100383685 | SNV | T | C | 1 | 25074 | Hetero | 84 | 133 | 63.2 | 0.47 | 0 | 84 | Yes | ZAN |
| 7 | 100549803 | SNV | C | T | 1 | 25074 | Hetero | 108 | 647 | 16.7 | 0.48 | 0 | 520 | No | MUC3A |
| 7 | 100552607 | SNV | C | A | 1 | 25074 | Hetero | 134 | 702 | 19.1 | 0.50 | 0 | 546 | Yes | MUC3A |
| 7 | 100552673 | SNV | C | T | 1 | 25074 | Hetero | 159 | 727 | 21.9 | 0.48 | 0 | 559 | Yes | MUC3A |
| 7 | 102412901 | SNV | T | C | 1 | 25074 | Hetero | 68 | 192 | 35.4 | 0.36 | 0 | 88 | - | FAM185A |
| 7 | 102993452 | SNV | A | G | 1 | 25074 | Hetero | 31 | 88 | 35.2 | 0.06 | 0 | 64 | - | PSMC2 |
| 7 | 107560202 | SNV | T | G | 1 | 25074 | Hetero | 22 | 55 | 40.0 | 0.25 | 0 | 46 | - | DLD |
| 7 | 112757960 | SNV | C | A | 1 | 25074 | Hetero | 6 | 60 | 10.0 | 0.17 | 0 | 47 | - | RP11-736E3.1 |
| 7 | 115898616 | Deletion | T | - | 1 | 25074 | Hetero | 25 | 82 | 30.5 | 0.37 | 0 | 77 | - | TES |
| 7 | 117307110 | SNV | T | C | 1 | 25074 | Hetero | 25 | 196 | 12.8 | 0.41 | 0 | 162 | Yes | CFTR |
| 7 | 127999221 | SNV | C | T | 1 | 25074 | Hetero | 80 | 174 | 46.0 | 0.48 | 0 | 79 | Yes | PRRT4 |
| 7 | 128587389 | SNV | A | T | 1 | 25074 | Hetero | 9 | 90 | 10.0 | 0.44 | 0 | 62 | Yes | IRF5 |
| 7 | 130292707 | SNV | T | G | 1 | 25074 | Hetero | 77 | 202 | 38.1 | 0.32 | 0 | 140 | - | COPG2 |
| 7 | 134253042 | SNV | G | A | 1 | 25074 | Hetero | 91 | 287 | 31.7 | 0.30 | 0 | 216 | Yes | AKR1B15 |
| 7 | 135072788 | SNV | C | T | 1 | 25074 | Hetero | 38 | 61 | 62.3 | 0.43 | 0 | 56 | - | CNOT4 |
| 7 | 135298865 | SNV | A | C | 1 | 25074 | Hetero | 53 | 79 | 67.1 | 0.07 | 0 | 75 | - | NUP205 |
| 7 | 135370476 | SNV | A | T | 1 | 25074 | Hetero | 62 | 96 | 64.6 | 0.25 | 0 | 70 | - | C7orf73 |
| 7 | 141538813 | Deletion | A | - | 1 | 25074 | Hetero | 9 | 66 | 13.6 | 0.33 | 0 | 33 | - | PRSS37 |
| 7 | 142470614 | SNV | C | T | 1 | 25074 | Hetero | 40 | 366 | 10.9 | 0.20 | 0 | 295 | - | PRSS3P1 |
| 7 | 142480012 | SNV | C | T | 1 | 25074 | Hetero | 41 | 81 | 50.6 | 0.39 | 0 | 78 | - | PRSS3P2 |
| 7 | 142480034 | SNV | C | A | 1 | 25074 | Hetero | 31 | 71 | 43.7 | 0.44 | 0 | 74 | - | PRSS3P2 |
| 7 | 142498902 | SNV | C | T | 1 | 25074 | Hetero | 100 | 295 | 33.9 | 0.44 | 0 | 264 | Yes | TRBC2 |
| 7 | 143004046 | SNV | A | G | 1 | 25074 | Hetero | 37 | 100 | 37.0 | 0.32 | 0 | 75 | - | CASP2 |
| 7 | 143549350 | SNV | A | G | 1 | 25074 | Hetero | 29 | 124 | 23.4 | 0.38 | 0 | 373 | - | FAM115A |
| 7 | 149525816 | SNV | T | C | 1 | 25074 | Hetero | 34 | 114 | 29.8 | 0.19 | 0 | 74 | - | SSPO |
| 7 | 149562496 | SNV | C | T | 1 | 25074 | Hetero | 174 | 259 | 67.2 | 0.44 | 0 | 187 | - | ZNF862 |
| 7 | 150420648 | SNV | C | T | 1 | 25074 | Hetero | 79 | 122 | 64.8 | 0.49 | 0 | 68 | - | GIMAP1 |
| 7 | 150910323 | SNV | T | C | 1 | 25074 | Hetero | 42 | 70 | 60.0 | 0.50 | 0 | 57 | - | ABCF2 |
| 7 | 153749831 | SNV | G | C | 1 | 25074 | Hetero | 88 | 154 | 57.1 | 0.49 | 0 | 136 | - | DPP6 |
| 7 | 157000132 | SNV | C | T | 1 | 25074 | Hetero | 114 | 190 | 60.0 | 0.40 | 0 | 151 | Yes | UBE3C |
| 7 | 105146593 | Insertion | - | TT | 2 | 25074 | Hetero | 64 | 96 | 66.7 | 0.29 | 0 | 90 | - | PUS7 |
| 7 | 111474749 | Insertion | - | A | 1 | 25074 | Hetero | 13 | 32 | 40.6 | 0.21 | 0 | 31 | - | DOCK4 |
| 7 | 115897792 | Insertion | - | AG | 2 | 25074 | Hetero | 24 | 69 | 34.8 | 0.43 | 0 | 57 | - | TES |
| 7 | 117188660 | Insertion | - | TG | 2 | 25074 | Hetero | 11 | 41 | 26.8 | 0.31 | 0 | 32 | - | CFTR |
| 7 | 137563925 | Insertion | - | A | 1 | 25074 | Hetero | 98 | 285 | 34.4 | 0.45 | 0 | 211 | - | CREB3L2 |
| 7 | 139097282 | Insertion | - | C | 1 | 25074 | Homo | 46 | 48 | 95.8 | 0.25 | 0 | 35 | - | LUC7L2 |
| 7 | 143050960 | Insertion | - | C | 1 | 25074 | Homo | 66 | 67 | 98.5 | 0.46 | 0 | 59 | - | FAM131B |
| 7 | 149589496 | Insertion | - | C | 1 | 25074 | Homo | 76 | 81 | 93.8 | 0.32 | 0 | 40 | - | RP11-728K20.1 |
| 7 | 150706436 | Insertion | - | C | 1 | 25074 | Hetero | 22 | 77 | 28.6 | 0.38 | 0 | 55 | - | NOS3 |
| 7 | 150752470 | Insertion | - | C | 1 | 25074 | Hetero | 68 | 91 | 74.7 | 0.32 | 0 | 88 | - | CDK5 |
| 7 | 27181225 | Insertion | - | T | 1 | 25074 | Hetero | 11 | 59 | 18.6 | 0.33 | 0 | 64 | - | HOXA3 |
| 7 | 31697913 | Insertion | - | T | 1 | 25074 | Hetero | 4 | 31 | 12.9 | 0.40 | 0 | 36 | Insertion | CCDC129 |
| 7 | 33397406 | Insertion | - | T | 1 | 25074 | Hetero | 38 | 46 | 82.6 | 0.19 | 0 | 48 | - | BBS9 |
| 7 | 42000586 | Insertion | - | T | 1 | 25074 | Hetero | 42 | 55 | 76.4 | 0.28 | 0 | 101 | - | GLI3 |
| 7 | 50800100 | Insertion | - | A | 1 | 25074 | Hetero | 14 | 111 | 12.6 | 0.33 | 0 | 141 | - | GRB10 |
| 7 | 66276142 | Insertion | - | T | 1 | 25074 | Hetero | 69 | 101 | 68.3 | 0.49 | 0 | 71 | - | KCTD7 |
| 7 | 8267926 | Insertion | - | A | 1 | 25074 | Hetero | 9 | 30 | 30.0 | 0.36 | 0 | 36 | - | ICA1 |
| 7 | 87902816 | Insertion | - | GT | 2 | 25074 | Hetero | 23 | 56 | 41.1 | 0.46 | 0 | 53 | - | AC003991.3 |
| 8 | 1618024 | SNV | C | T | 1 | 25074 | Hetero | 43 | 63 | 68.3 | 0.11 | 0 | 39 | - | DLGAP2 |
| 8 | 1655714 | SNV | G | A | 1 | 25074 | Hetero | 69 | 157 | 43.9 | 0.49 | 0 | 103 | - | DLGAP2 |
| 8 | 3015457 | SNV | G | C | 1 | 25074 | Hetero | 57 | 113 | 50.4 | 0.27 | 0 | 84 | Yes | CSMD1 |
| 8 | 21955161 | SNV | G | C | 1 | 25074 | Hetero | 27 | 56 | 48.2 | 0.36 | 0 | 39 | - | FAM160B2 |
| 8 | 21955246 | SNV | G | A | 1 | 25074 | Hetero | 29 | 69 | 42.0 | 0.31 | 0 | 41 | Yes | FAM160B2 |
| 8 | 22926598 | SNV | G | A | 1 | 25074 | Hetero | 51 | 127 | 40.2 | 0.49 | 0 | 90 | - | TNFRSF10B |
| 8 | 22993189 | SNV | T | C | 1 | 25074 | Hetero | 50 | 98 | 51.0 | 0.39 | 0 | 58 | - | TNFRSF10D |
| 8 | 22994121 | Deletion | C | - | 1 | 25074 | Hetero | 33 | 109 | 30.3 | 0.45 | 0 | 74 | - | TNFRSF10D |
| 8 | 25042479 | SNV | C | G | 1 | 25074 | Hetero | 47 | 98 | 48.0 | 0.42 | 0 | 66 | - | DOCK5 |
| 8 | 27661085 | SNV | G | A | 1 | 25074 | Hetero | 31 | 59 | 52.5 | 0.42 | 0 | 55 | - | ESCO2 |
| 8 | 38187215 | SNV | C | T | 1 | 25074 | Hetero | 61 | 141 | 43.3 | 0.46 | 0 | 85 | Yes | WHSC1L1 |
| 8 | 42188457 | SNV | C | T | 1 | 25074 | Hetero | 60 | 119 | 50.4 | 0.43 | 0 | 96 | Yes | IKBKB |
| 8 | 48504643 | SNV | A | G | 1 | 25074 | Hetero | 98 | 247 | 39.7 | 0.47 | 0 | 154 | - | KIAA0146 |
| 8 | 48873646 | SNV | G | A | 1 | 25074 | Hetero | 125 | 182 | 68.7 | 0.40 | 0 | 99 | - | MCM4 |
| 8 | 69359766 | SNV | C | T | 1 | 25074 | Hetero | 85 | 122 | 69.7 | 0.50 | 0 | 80 | - | C8orf34 |
| 8 | 70674165 | SNV | G | C | 1 | 25074 | Hetero | 13 | 110 | 11.8 | 0.27 | 0 | 78 | - | SLCO5A1 |
| 8 | 71128925 | SNV | C | A | 1 | 25074 | Hetero | 285 | 468 | 60.9 | 0.40 | 0 | 291 | Yes | NCOA2 |
| 8 | 97156398 | SNV | G | C | 1 | 25074 | Hetero | 22 | 73 | 30.1 | 0.46 | 0 | 31 | - | GDF6 |
| 8 | 101933029 | SNV | G | A | 1 | 25074 | Hetero | 104 | 160 | 65.0 | 0.46 | 0 | 62 | - | YWHAZ |
| 8 | 104383848 | SNV | C | G | 1 | 25074 | Hetero | 61 | 88 | 69.3 | 0.28 | 0 | 44 | - | CTHRC1 |
| 8 | 107738292 | SNV | G | T | 1 | 25074 | Hetero | 152 | 242 | 62.8 | 0.37 | 0 | 149 | - | OXR1 |
| 8 | 110457114 | SNV | A | G | 1 | 25074 | Hetero | 92 | 284 | 32.4 | 0.45 | 0 | 209 | No | PKHD1L1 |
| 8 | 110978437 | SNV | G | C | 1 | 25074 | Hetero | 18 | 88 | 20.5 | 0.38 | 0 | 46 | - | KCNV1 |
| 8 | 113323365 | SNV | G | C | 1 | 25074 | Hetero | 37 | 195 | 19.0 | 0.31 | 0 | 113 | Yes | CSMD3 |
| 8 | 120598428 | SNV | T | C | 1 | 25074 | Hetero | 179 | 270 | 66.3 | 0.41 | 0 | 143 | No | ENPP2 |
| 8 | 124693405 | SNV | C | T | 1 | 25074 | Hetero | 58 | 84 | 69.0 | 0.43 | 0 | 54 | - | ANXA13 |
| 8 | 130496338 | SNV | T | C | 1 | 25074 | Hetero | 24 | 81 | 29.6 | 0.34 | 0 | 52 | - | CCDC26 |
| 8 | 130764514 | SNV | T | C | 1 | 25074 | Hetero | 123 | 369 | 33.3 | 0.28 | 0 | 233 | - | GSDMC |
| 8 | 139142935 | SNV | G | T | 1 | 25074 | Hetero | 16 | 131 | 12.2 | 0.42 | 0 | 76 | - | FAM135B |
| 8 | 140744261 | SNV | G | A | 1 | 25074 | Hetero | 57 | 151 | 37.7 | 0.43 | 0 | 77 | No | TRAPPC9 |
| 8 | 144644681 | SNV | C | T | 1 | 25074 | Hetero | 67 | 171 | 39.2 | 0.25 | 0 | 59 | Yes | GSDMD |
| 8 | 144999996 | SNV | G | A | 1 | 25074 | Hetero | 36 | 61 | 59.0 | 0.30 | 0 | 37 | No | PLEC |
| 8 | 145747468 | SNV | C | A | 1 | 25074 | Hetero | 166 | 499 | 33.3 | 0.22 | 0 | 247 | - | LRRC14 |
| 8 | 119123567 | Insertion | - | T | 1 | 25074 | Hetero | 30 | 155 | 19.4 | 0.46 | 0 | 68 | - | EXT1 |
| 8 | 127567597 | Insertion | - | A | 1 | 25074 | Homo | 71 | 72 | 98.6 | 0.42 | 0 | 52 | - | FAM84B |
| 8 | 133837109 | Insertion | - | T | 1 | 25074 | Hetero | 24 | 43 | 55.8 | 0.32 | 0 | 32 | - | PHF20L1 |
| 8 | 41508550 | Insertion | - | TT | 2 | 25074 | Hetero | 20 | 75 | 26.7 | 0.50 | 0 | 67 | - | NKX6-3 |
| 8 | 52732790 | Insertion | - | T | 1 | 25074 | Homo | 124 | 128 | 96.9 | 0.42 | 0 | 84 | - | PCMTD1 |
| 8 | 55543160 | Insertion | - | TT | 2 | 25074 | Hetero | 65 | 88 | 73.9 | 0.38 | 0 | 65 | - | RP1 |
| 8 | 74231476 | Insertion | - | GG | 2 | 25074 | Hetero | 51 | 84 | 60.7 | 0.21 | 0 | 78 | - | RDH10 |
| 8 | 92971183 | Insertion | - | T | 1 | 25074 | Hetero | 28 | 73 | 38.4 | 0.44 | 0 | 58 | - | RUNX1T1 |
| 9 | 2718555 | SNV | C | G | 1 | 25074 | Hetero | 47 | 60 | 78.3 | 0.43 | 0 | 86 | No | KCNV2 |
| 9 | 3824934 | SNV | A | T | 1 | 25074 | Hetero | 19 | 35 | 54.3 | 0.43 | 0 | 56 | - | GLIS3 |
| 9 | 6533204 | SNV | G | C | 1 | 25074 | Hetero | 22 | 71 | 31.0 | 0.38 | 0 | 99 | - | GLDC |
| 9 | 15972674 | SNV | A | G | 1 | 25074 | Hetero | 17 | 69 | 24.6 | 0.44 | 0 | 61 | - | CCDC171 |
| 9 | 16418690 | Deletion | A | - | 1 | 25074 | Hetero | 4 | 39 | 10.3 | 0.25 | 0 | 53 | - | BNC2 |
| 9 | 16418692 | Deletion | G | - | 1 | 25074 | Hetero | 4 | 38 | 10.5 | 0.25 | 0 | 50 | - | BNC2 |
| 9 | 20363634 | Deletion | A | - | 1 | 25074 | Hetero | 39 | 256 | 15.2 | 0.41 | 0 | 266 | - | MLLT3 |
| 9 | 20770081 | SNV | A | G | 1 | 25074 | Hetero | 76 | 94 | 80.9 | 0.32 | 0 | 125 | No | FOCAD |
| 9 | 34726202 | SNV | A | G | 1 | 25074 | Hetero | 83 | 105 | 79.0 | 0.33 | 0 | 158 | No | FAM205A |
| 9 | 34835264 | SNV | A | G | 1 | 25074 | Hetero | 122 | 441 | 27.7 | 0.37 | 0 | 345 | No | FAM205B |
| 9 | 35062354 | SNV | G | C | 1 | 25074 | Hetero | 105 | 128 | 82.0 | 0.28 | 0 | 136 | - | VCP |
| 9 | 35399063 | SNV | G | T | 1 | 25074 | Hetero | 188 | 238 | 79.0 | 0.49 | 0 | 229 | - | UNC13B |
| 9 | 40498917 | SNV | G | A | 1 | 25074 | Hetero | 15 | 121 | 12.4 | 0.18 | 0 | 174 | - | RP11-292B8.1 |
| 9 | 72961584 | SNV | T | C | 1 | 25074 | Hetero | 36 | 153 | 23.5 | 0.38 | 0 | 162 | - | SMC5 |
| 9 | 74533312 | SNV | T | C | 1 | 25074 | Hetero | 42 | 55 | 76.4 | 0.23 | 0 | 66 | - | C9orf85 |
| 9 | 77416900 | SNV | C | T | 1 | 25074 | Hetero | 97 | 113 | 85.8 | 0.35 | 0 | 114 | No | TRPM6 |
| 9 | 79438554 | SNV | G | C | 1 | 25074 | Hetero | 26 | 116 | 22.4 | 0.47 | 0 | 154 | Yes | PRUNE2 |
| 9 | 80888008 | SNV | C | T | 1 | 25074 | Hetero | 37 | 52 | 71.2 | 0.44 | 0 | 66 | - | CEP78 |
| 9 | 88961359 | SNV | C | T | 1 | 25074 | Hetero | 71 | 90 | 78.9 | 0.44 | 0 | 73 | Yes | ZCCHC6 |
| 9 | 95064018 | SNV | A | G | 1 | 25074 | Hetero | 17 | 49 | 34.7 | 0.48 | 0 | 32 | Yes | NOL8 |
| 9 | 96070828 | SNV | G | A | 1 | 25074 | Hetero | 32 | 44 | 72.7 | 0.17 | 0 | 38 | Yes | WNK2 |
| 9 | 97741730 | SNV | T | C | 1 | 25074 | Hetero | 39 | 47 | 83.0 | 0.15 | 0 | 53 | - | C9orf3 |
| 9 | 98206399 | SNV | T | A | 1 | 25074 | Hetero | 36 | 49 | 73.5 | 0.42 | 0 | 65 | - | PTCH1 |
| 9 | 100122356 | SNV | G | T | 1 | 25074 | Hetero | 63 | 77 | 81.8 | 0.37 | 0 | 73 | Yes | C9orf174 |
| 9 | 100616020 | SNV | G | C | 1 | 25074 | Hetero | 32 | 40 | 80.0 | 0.26 | 0 | 61 | - | FOXE1 |
| 9 | 104324984 | SNV | G | C | 1 | 25074 | Hetero | 14 | 63 | 22.2 | 0.29 | 0 | 79 | - | RNF20 |
| 9 | 113550224 | SNV | G | A | 1 | 25074 | Hetero | 48 | 309 | 15.5 | 0.43 | 0 | 296 | - | MUSK |
| 9 | 115203997 | SNV | A | C | 1 | 25074 | Hetero | 66 | 77 | 85.7 | 0.48 | 0 | 59 | Yes | HSDL2 |
| 9 | 117120223 | SNV | C | T | 1 | 25074 | Hetero | 31 | 37 | 83.8 | 0.46 | 0 | 33 | Yes | AKNA |
| 9 | 126140057 | SNV | C | T | 1 | 25074 | Hetero | 104 | 127 | 81.9 | 0.35 | 0 | 103 | - | CRB2 |
| 9 | 128127792 | SNV | G | A | 1 | 25074 | Hetero | 58 | 70 | 82.9 | 0.46 | 0 | 57 | - | GAPVD1 |
| 9 | 130914319 | SNV | A | G | 1 | 25074 | Hetero | 20 | 92 | 21.7 | 0.41 | 0 | 81 | - | LCN2 |
| 9 | 131095880 | SNV | C | A | 1 | 25074 | Hetero | 14 | 93 | 15.1 | 0.47 | 0 | 66 | Yes | COQ4 |
| 9 | 131150964 | SNV | A | C | 1 | 25074 | Hetero | 13 | 82 | 15.9 | 0.43 | 0 | 76 | - | URM1 |
| 9 | 132575837 | Deletion | C | - | 1 | 25074 | Hetero | 50 | 60 | 83.3 | 0.47 | 0 | 60 | - | TOR1A |
| 9 | 135211759 | SNV | A | G | 1 | 25074 | Hetero | 64 | 78 | 82.1 | 0.25 | 0 | 76 | No | SETX |
| 9 | 136131397 | SNV | G | A | 1 | 25074 | Hetero | 26 | 163 | 16.0 | 0.35 | 0 | 129 | - | ABO |
| 9 | 136249588 | SNV | C | T | 1 | 25074 | Hetero | 29 | 34 | 85.3 | 0.19 | 0 | 40 | - | C9orf96 |
| 9 | 136323014 | SNV | C | T | 1 | 25074 | Hetero | 118 | 142 | 83.1 | 0.28 | 0 | 115 | - | ADAMTS13 |
| 9 | 136433542 | SNV | C | T | 1 | 25074 | Hetero | 83 | 107 | 77.6 | 0.43 | 0 | 100 | No | ADAMTSL2 |
| 9 | 136434598 | SNV | A | G | 1 | 25074 | Hetero | 133 | 173 | 76.9 | 0.49 | 0 | 139 | No | ADAMTSL2 |
| 9 | 136438985 | SNV | G | A | 1 | 25074 | Hetero | 117 | 149 | 78.5 | 0.47 | 0 | 96 | No | ADAMTSL2 |
| 9 | 139612156 | SNV | T | C | 1 | 25074 | Hetero | 183 | 241 | 75.9 | 0.41 | 0 | 188 | Yes | FAM69B |
| 9 | 139642876 | SNV | G | A | 1 | 25074 | Hetero | 118 | 149 | 79.2 | 0.28 | 0 | 117 | No | LCN6 |
| 9 | 139751699 | SNV | C | T | 1 | 25074 | Hetero | 96 | 113 | 85.0 | 0.36 | 0 | 115 | No | MAMDC4 |
| 9 | 140094300 | SNV | G | A | 1 | 25074 | Hetero | 94 | 117 | 80.3 | 0.32 | 0 | 84 | No | TPRN |
| 9 | 111935691 | Insertion | - | T | 1 | 25074 | Hetero | 37 | 46 | 80.4 | 0.21 | 0 | 42 | - | EPB41L4B |
| 9 | 115060100 | Insertion | - | C | 1 | 25074 | Hetero | 86 | 109 | 78.9 | 0.09 | 0 | 91 | - | PTBP3 |
| 9 | 131235119 | Deletion | TG | - | 2 | 25074 | Hetero | 18 | 84 | 21.4 | 0.26 | 0 | 70 | - | ODF2 |
| 9 | 6553514 | Insertion | - | T | 1 | 25074 | Hetero | 25 | 102 | 24.5 | 0.45 | 0 | 142 | - | GLDC |
| 9 | 77596827 | Insertion | - | T | 1 | 25074 | Hetero | 39 | 51 | 76.5 | 0.43 | 0 | 43 | - | C9orf41 |
| 9 | 85987713 | Insertion | - | C | 1 | 25074 | Hetero | 68 | 92 | 73.9 | 0.28 | 0 | 109 | - | FRMD3 |
| 10 | 1094190 | SNV | C | T | 1 | 25074 | Hetero | 46 | 209 | 22.0 | 0.29 | 0 | 150 | - | IDI1 |
| 10 | 5807614 | SNV | A | C | 1 | 25074 | Hetero | 31 | 41 | 75.6 | 0.43 | 0 | 39 | - | GDI2 |
| 10 | 7679383 | SNV | C | T | 1 | 25074 | Hetero | 29 | 110 | 26.4 | 0.43 | 0 | 110 | Yes | ITIH5 |
| 10 | 13043224 | SNV | T | C | 1 | 25074 | Hetero | 101 | 129 | 78.3 | 0.40 | 0 | 131 | Yes | CCDC3 |
| 10 | 15058958 | SNV | G | A | 1 | 25074 | Hetero | 45 | 192 | 23.4 | 0.40 | 0 | 183 | - | RP11-271M1.2 |
| 10 | 25140422 | SNV | A | G | 1 | 25074 | Hetero | 21 | 73 | 28.8 | 0.32 | 0 | 65 | - | PRTFDC1 |
| 10 | 27485129 | Deletion | A | - | 1 | 25074 | Hetero | 26 | 37 | 70.3 | 0.26 | 0 | 33 | - | ACBD5 |
| 10 | 46281224 | SNV | T | C | 1 | 25074 | Hetero | 204 | 271 | 75.3 | 0.48 | 0 | 243 | - | FAM21C |
| 10 | 47903185 | SNV | T | C | 1 | 25074 | Hetero | 6 | 46 | 13.0 | 0.17 | 0 | 88 | No | FAM21B |
| 10 | 50122287 | SNV | G | T | 1 | 25074 | Hetero | 42 | 234 | 17.9 | 0.41 | 0 | 145 | - | WDFY4 |
| 10 | 58120111 | SNV | A | G | 1 | 25074 | Hetero | 66 | 300 | 22.0 | 0.33 | 0 | 225 | No | ZWINT |
| 10 | 61424004 | SNV | C | T | 1 | 25074 | Hetero | 139 | 182 | 76.4 | 0.45 | 0 | 137 | No | SLC16A9 |
| 10 | 64161246 | Deletion | G | - | 1 | 25074 | Homo | 103 | 103 | 100.0 | 0.50 | 0 | 82 | - | ZNF365 |
| 10 | 69971540 | SNV | A | G | 1 | 25074 | Hetero | 58 | 86 | 67.4 | 0.41 | 0 | 91 | - | MYPN |
| 10 | 73567358 | SNV | C | G | 1 | 25074 | Hetero | 250 | 324 | 77.2 | 0.45 | 0 | 196 | Yes | CDH23 |
| 10 | 73773212 | SNV | C | A | 1 | 25074 | Hetero | 178 | 239 | 74.5 | 0.44 | 0 | 154 | - | CHST3 |
| 10 | 73976612 | SNV | C | A | 1 | 25074 | Hetero | 20 | 97 | 20.6 | 0.46 | 0 | 63 | - | ASCC1 |
| 10 | 81143961 | SNV | C | A | 1 | 25074 | Hetero | 28 | 171 | 16.4 | 0.31 | 0 | 124 | - | ZCCHC24 |
| 10 | 81928910 | SNV | C | G | 1 | 25074 | Hetero | 24 | 121 | 19.8 | 0.46 | 0 | 73 | Yes | ANXA11 |
| 10 | 81995007 | SNV | A | G | 1 | 25074 | Hetero | 66 | 139 | 47.5 | 0.14 | 0 | 34 | - | RP11-40F6.1 |
| 10 | 82404340 | SNV | A | G | 1 | 25074 | Hetero | 67 | 76 | 88.2 | 0.47 | 0 | 55 | - | SH2D4B |
| 10 | 88281450 | SNV | A | C | 1 | 25074 | Hetero | 138 | 172 | 80.2 | 0.42 | 0 | 139 | Yes | WAPAL |
| 10 | 90575228 | SNV | C | A | 1 | 25074 | Hetero | 61 | 77 | 79.2 | 0.46 | 0 | 70 | Yes | LIPM |
| 10 | 101474424 | SNV | C | T | 1 | 25074 | Hetero | 30 | 135 | 22.2 | 0.07 | 0 | 85 | Yes | CUTC |
| 10 | 101558897 | SNV | C | A | 1 | 25074 | Hetero | 30 | 34 | 88.2 | 0.15 | 0 | 32 | - | ABCC2 |
| 10 | 101560313 | SNV | A | G | 1 | 25074 | Hetero | 122 | 161 | 75.8 | 0.23 | 0 | 111 | Yes | ABCC2 |
| 10 | 102058400 | SNV | C | T | 1 | 25074 | Hetero | 72 | 105 | 68.6 | 0.44 | 0 | 98 | Yes | PKD2L1 |
| 10 | 103338754 | SNV | G | A | 1 | 25074 | Hetero | 31 | 147 | 21.1 | 0.42 | 0 | 107 | - | DPCD |
| 10 | 105206792 | SNV | C | G | 1 | 25074 | Hetero | 9 | 65 | 13.8 | 0.18 | 0 | 57 | - | CALHM2 |
| 10 | 114849322 | SNV | G | A | 1 | 25074 | Hetero | 48 | 58 | 82.8 | 0.25 | 0 | 52 | - | TCF7L2 |
| 10 | 121589929 | SNV | G | A | 1 | 25074 | Hetero | 20 | 88 | 22.7 | 0.29 | 0 | 67 | - | MCMBP |
| 10 | 125453912 | SNV | C | T | 1 | 25074 | Hetero | 45 | 74 | 60.8 | 0.28 | 0 | 49 | - | GPR26 |
| 10 | 129676143 | SNV | G | A | 1 | 25074 | Hetero | 17 | 98 | 17.3 | 0.33 | 0 | 43 | - | CLRN3 |
| 10 | 131633807 | SNV | G | A | 1 | 25074 | Hetero | 50 | 70 | 71.4 | 0.49 | 0 | 54 | - | EBF3 |
| 10 | 131635007 | SNV | G | A | 1 | 25074 | Hetero | 13 | 61 | 21.3 | 0.36 | 0 | 45 | - | EBF3 |
| 10 | 134161838 | SNV | C | T | 1 | 25074 | Hetero | 205 | 258 | 79.5 | 0.45 | 0 | 229 | Yes | LRRC27 |
| 10 | 101635517 | Insertion | - | G | 1 | 25074 | Homo | 117 | 118 | 99.2 | 0.30 | 0 | 83 | - | DNMBP |
| 10 | 115883098 | Insertion | - | T | 1 | 25074 | Hetero | 7 | 62 | 11.3 | 0.50 | 0 | 39 | - | C10orf118 |
| 10 | 118969015 | Insertion | - | T | 1 | 25074 | Hetero | 44 | 207 | 21.3 | 0.39 | 0 | 164 | Insertion | KCNK18 |
| 10 | 124924485 | Deletion | TT | - | 2 | 25074 | Homo | 181 | 183 | 98.9 | 0.42 | 0 | 106 | - | BUB3 |
| 10 | 126097585 | Insertion | - | T | 1 | 25074 | Hetero | 12 | 49 | 24.5 | 0.50 | 0 | 33 | - | OAT |
| 10 | 127529756 | Insertion | - | TG | 2 | 25074 | Hetero | 145 | 176 | 82.4 | 0.36 | 0 | 126 | - | BCCIP |
| 10 | 135367430 | Insertion | - | T | 1 | 25074 | Hetero | 15 | 58 | 25.9 | 0.40 | 0 | 40 | - | SPRN |
| 10 | 3214995 | Insertion | - | C | 1 | 25074 | Hetero | 177 | 232 | 76.3 | 0.47 | 0 | 158 | - | PITRM1 |
| 10 | 33467411 | Insertion | - | T | 1 | 25074 | Hetero | 69 | 81 | 85.2 | 0.35 | 0 | 73 | - | NRP1 |
| 10 | 3819321 | Insertion | - | A | 1 | 25074 | Hetero | 42 | 66 | 63.6 | 0.48 | 0 | 39 | - | KLF6 |
| 10 | 5030198 | Insertion | - | T | 1 | 25074 | Hetero | 20 | 82 | 24.4 | 0.33 | 0 | 81 | - | AKR1C2 |
| 10 | 59951461 | Insertion | - | A | 1 | 25074 | Hetero | 128 | 163 | 78.5 | 0.47 | 0 | 152 | - | IPMK |
| 10 | 61411522 | Deletion | CA | - | 2 | 25074 | Hetero | 5 | 39 | 12.8 | 0.40 | 0 | 32 | - | SLC16A9 |
| 10 | 70137270 | Deletion | TG | - | 2 | 25074 | Hetero | 7 | 63 | 11.1 | 0.29 | 0 | 54 | - | RUFY2 |
| 10 | 75135411 | Insertion | - | A | 1 | 25074 | Hetero | 17 | 89 | 19.1 | 0.50 | 0 | 61 | - | ANXA7 |
| 10 | 81275626 | Insertion | - | T | 1 | 25074 | Hetero | 141 | 178 | 79.2 | 0.47 | 0 | 134 | - | EIF5AL1 |
| 10 | 89720633 | Insertion | - | T | 1 | 25074 | Hetero | 14 | 57 | 24.6 | 0.07 | 0 | 38 | - | PTEN |
| 11 | 197279 | SNV | C | T | 1 | 25074 | Hetero | 75 | 164 | 45.7 | 0.37 | 0 | 78 | - | BET1L |
| 11 | 783802 | SNV | G | A | 1 | 25074 | Hetero | 161 | 339 | 47.5 | 0.35 | 0 | 181 | Yes | AP006621.5 |
| 11 | 831871 | SNV | G | A | 1 | 25074 | Hetero | 265 | 507 | 52.3 | 0.31 | 0 | 250 | - | AP006621.8 |
| 11 | 1267192 | SNV | A | C | 1 | 25074 | Hetero | 27 | 173 | 15.6 | 0.38 | 0 | 139 | Yes | MUC5B |
| 11 | 1587182 | SNV | T | G | 1 | 25074 | Hetero | 28 | 63 | 44.4 | 0.46 | 0 | 33 | - | DUSP8 |
| 11 | 1774742 | SNV | G | A | 1 | 25074 | Hetero | 185 | 404 | 45.8 | 0.35 | 0 | 193 | No | CTSD |
| 11 | 1890855 | SNV | C | T | 1 | 25074 | Hetero | 91 | 182 | 50.0 | 0.25 | 0 | 92 | - | LSP1 |
| 11 | 1977739 | SNV | C | T | 1 | 25074 | Hetero | 38 | 96 | 39.6 | 0.40 | 0 | 57 | - | MRPL23 |
| 11 | 2869583 | SNV | G | T | 1 | 25074 | Hetero | 76 | 135 | 56.3 | 0.46 | 0 | 66 | - | KCNQ1 |
| 11 | 5633709 | SNV | A | C | 1 | 25074 | Hetero | 35 | 71 | 49.3 | 0.38 | 0 | 61 | - | HBG2 |
| 11 | 5727880 | SNV | C | A | 1 | 25074 | Hetero | 53 | 87 | 60.9 | 0.37 | 0 | 34 | - | TRIM5 |
| 11 | 6633646 | SNV | G | C | 1 | 25074 | Hetero | 97 | 189 | 51.3 | 0.36 | 0 | 105 | - | TAF10 |
| 11 | 6662955 | SNV | G | A | 1 | 25074 | Hetero | 62 | 126 | 49.2 | 0.49 | 0 | 70 | - | DCHS1 |
| 11 | 9302810 | SNV | G | A | 1 | 25074 | Hetero | 36 | 68 | 52.9 | 0.40 | 0 | 39 | - | TMEM41B |
| 11 | 10014643 | SNV | T | G | 1 | 25074 | Hetero | 49 | 95 | 51.6 | 0.08 | 0 | 44 | Yes | SBF2 |
| 11 | 20066528 | SNV | G | A | 1 | 25074 | Hetero | 40 | 102 | 39.2 | 0.27 | 0 | 65 | Yes | NAV2 |
| 11 | 27494163 | SNV | C | G | 1 | 25074 | Hetero | 39 | 97 | 40.2 | 0.23 | 0 | 96 | - | LGR4 |
| 11 | 45263064 | SNV | G | C | 1 | 25074 | Hetero | 4 | 36 | 11.1 | 0.50 | 0 | 37 | - | SYT13 |
| 11 | 45263072 | Deletion | A | - | 1 | 25074 | Hetero | 4 | 37 | 10.8 | 0.50 | 0 | 47 | - | SYT13 |
| 11 | 47369412 | SNV | G | A | 1 | 25074 | Hetero | 32 | 71 | 45.1 | 0.25 | 0 | 40 | Yes | MYBPC3 |
| 11 | 57970851 | SNV | G | A | 1 | 25074 | Hetero | 120 | 291 | 41.2 | 0.49 | 0 | 227 | Yes | OR1S2 |
| 11 | 59620410 | SNV | C | A | 1 | 25074 | Hetero | 17 | 99 | 17.2 | 0.30 | 0 | 83 | - | TCN1 |
| 11 | 59947375 | SNV | A | T | 1 | 25074 | Hetero | 84 | 185 | 45.4 | 0.49 | 0 | 123 | Yes | MS4A6A |
| 11 | 61513588 | SNV | G | T | 1 | 25074 | Hetero | 191 | 405 | 47.2 | 0.48 | 0 | 234 | - | DAGLA |
| 11 | 61981356 | SNV | A | G | 1 | 25074 | Hetero | 156 | 293 | 53.2 | 0.16 | 0 | 179 | - | SCGB2A1 |
| 11 | 62430764 | SNV | C | T | 1 | 25074 | Hetero | 53 | 112 | 47.3 | 0.22 | 0 | 58 | Yes | C11orf48 |
| 11 | 62995867 | SNV | G | C | 1 | 25074 | Hetero | 19 | 64 | 29.7 | 0.11 | 0 | 41 | - | SLC22A10 |
| 11 | 63177729 | SNV | A | G | 1 | 25074 | Hetero | 21 | 74 | 28.4 | 0.33 | 0 | 37 | - | SLC22A9 |
| 11 | 63177732 | SNV | A | G | 1 | 25074 | Hetero | 27 | 65 | 41.5 | 0.33 | 0 | 37 | - | SLC22A9 |
| 11 | 63723599 | SNV | A | G | 1 | 25074 | Hetero | 87 | 168 | 51.8 | 0.37 | 0 | 84 | - | NAA40 |
| 11 | 65272791 | SNV | G | A | 1 | 25074 | Hetero | 43 | 226 | 19.0 | 0.38 | 0 | 92 | - | MALAT1 |
| 11 | 71576918 | SNV | G | A | 1 | 25074 | Homo | 84 | 84 | 100.0 | 0.23 | 0 | 81 | - | RP11-849H4.2 |
| 11 | 73638742 | SNV | T | C | 1 | 25074 | Hetero | 176 | 208 | 84.6 | 0.41 | 0 | 33 | - | PAAF1 |
| 11 | 76751587 | SNV | G | A | 1 | 25074 | Hetero | 144 | 175 | 82.3 | 0.47 | 0 | 162 | Possible splice site disruption | B3GNT6 |
| 11 | 77937712 | SNV | G | A | 1 | 25074 | Hetero | 83 | 102 | 81.4 | 0.47 | 0 | 112 | Yes | GAB2 |
| 11 | 78440588 | SNV | G | A | 1 | 25074 | Hetero | 14 | 75 | 18.7 | 0.44 | 0 | 70 | Yes | ODZ4 |
| 11 | 102584466 | SNV | G | T | 1 | 25074 | Homo | 217 | 218 | 99.5 | 0.22 | 0 | 178 | - | MMP8 |
| 11 | 110334891 | SNV | C | T | 1 | 25074 | Hetero | 7 | 49 | 14.3 | 0.38 | 0 | 69 | - | FDX1 |
| 11 | 116661392 | SNV | C | A | 1 | 25074 | Hetero | 126 | 157 | 80.3 | 0.33 | 0 | 184 | Yes | APOA5 |
| 11 | 117169899 | SNV | G | T | 1 | 25074 | Hetero | 18 | 117 | 15.4 | 0.43 | 0 | 111 | - | BACE1 |
| 11 | 117708665 | SNV | G | T | 1 | 25074 | Hetero | 50 | 59 | 84.7 | 0.33 | 0 | 66 | - | FXYD6-FXYD2 |
| 11 | 117713539 | SNV | C | G | 1 | 25074 | Hetero | 29 | 155 | 18.7 | 0.44 | 0 | 128 | - | FXYD6 |
| 11 | 118915242 | SNV | C | G | 1 | 25074 | Hetero | 33 | 166 | 19.9 | 0.42 | 0 | 163 | - | HYOU1 |
| 11 | 120358786 | SNV | T | C | 1 | 25074 | Hetero | 52 | 70 | 74.3 | 0.42 | 0 | 83 | - | ARHGEF12 |
| 11 | 120743746 | SNV | A | G | 1 | 25074 | Hetero | 65 | 81 | 80.2 | 0.29 | 0 | 72 | - | GRIK4 |
| 11 | 123886320 | SNV | A | G | 1 | 25074 | Homo | 50 | 50 | 100.0 | 0.17 | 0 | 220 | No | OR10G4 |
| 11 | 125302071 | SNV | A | T | 1 | 25074 | Hetero | 19 | 125 | 15.2 | 0.43 | 0 | 115 | - | PKNOX2 |
| 11 | 125490765 | Deletion | A | - | 1 | 25074 | Hetero | 42 | 77 | 54.5 | 0.43 | 0 | 58 | - | STT3A |
| 11 | 126865019 | SNV | G | A | 1 | 25074 | Hetero | 21 | 85 | 24.7 | 0.46 | 0 | 87 | - | KIRREL3 |
| 11 | 134023210 | SNV | G | A | 1 | 25074 | Hetero | 26 | 116 | 22.4 | 0.50 | 0 | 94 | Yes | NCAPD3 |
| 11 | 134027913 | SNV | C | T | 1 | 25074 | Hetero | 21 | 92 | 22.8 | 0.35 | 0 | 119 | Yes | NCAPD3 |
| 11 | 101771148 | Insertion | - | A | 1 | 25074 | Hetero | 65 | 82 | 79.3 | 0.11 | 0 | 61 | - | ANGPTL5 |
| 11 | 103175289 | Insertion | - | T | 1 | 25074 | Hetero | 30 | 34 | 88.2 | 0.06 | 0 | 39 | - | DYNC2H1 |
| 11 | 10597292 | Insertion | - | T | 1 | 25074 | Hetero | 22 | 63 | 34.9 | 0.35 | 0 | 43 | - | MRVI1-AS1 |
| 11 | 19263370 | Insertion | - | TT | 2 | 25074 | Hetero | 4 | 36 | 11.1 | 0.40 | 0 | 31 | - | RP11-428C19.4 |
| 11 | 1959736 | Insertion | - | C | 1 | 25074 | Hetero | 67 | 167 | 40.1 | 0.46 | 0 | 96 | - | TNNT3 |
| 11 | 22400105 | Insertion | - | AA | 2 | 25074 | Homo | 80 | 82 | 97.6 | 0.40 | 0 | 75 | - | SLC17A6 |
| 11 | 26683861 | Insertion | - | C | 1 | 25074 | Hetero | 70 | 164 | 42.7 | 0.48 | 0 | 98 | - | ANO3 |
| 11 | 49208169 | Insertion | - | A | 1 | 25074 | Hetero | 11 | 48 | 22.9 | 0.08 | 0 | 43 | - | FOLH1 |
| 11 | 5013421 | Insertion | - | T | 1 | 25074 | Hetero | 56 | 147 | 38.1 | 0.23 | 0 | 96 | - | MMP26 |
| 11 | 5274452 | Insertion | - | T | 1 | 25074 | Hetero | 121 | 251 | 48.2 | 0.47 | 0 | 209 | - | HBG2 |
| 11 | 5775903 | Insertion | - | TT | 2 | 25074 | Hetero | 69 | 184 | 37.5 | 0.33 | 0 | 157 | - | TRIM5 |
| 11 | 67165217 | Insertion | - | CT | 2 | 25074 | Hetero | 410 | 572 | 71.7 | 0.40 | 0 | 145 | - | RAD9A |
| 12 | 4410520 | Deletion | T | - | 1 | 25074 | Homo | 69 | 78 | 88.5 | 0.47 | 0 | 50 | - | CCND2 |
| 12 | 5853432 | SNV | G | T | 1 | 25074 | Hetero | 79 | 181 | 43.6 | 0.43 | 0 | 87 | Yes | ANO2 |
| 12 | 7247156 | SNV | T | C | 1 | 25074 | Hetero | 40 | 68 | 58.8 | 0.36 | 0 | 38 | - | C1RL |
| 12 | 7248687 | SNV | C | T | 1 | 25074 | Hetero | 18 | 135 | 13.3 | 0.45 | 0 | 60 | - | C1RL |
| 12 | 10339247 | SNV | G | C | 1 | 25074 | Hetero | 53 | 110 | 48.2 | 0.24 | 0 | 71 | - | TMEM52B |
| 12 | 10977856 | Deletion | C | - | 1 | 25074 | Hetero | 115 | 214 | 53.7 | 0.20 | 0 | 134 | - | PRR4 |
| 12 | 14720554 | SNV | G | T | 1 | 25074 | Hetero | 33 | 83 | 39.8 | 0.45 | 0 | 72 | Yes | PLBD1 |
| 12 | 14947676 | Deletion | A | - | 1 | 25074 | Hetero | 44 | 98 | 44.9 | 0.22 | 0 | 62 | - | WBP11 |
| 12 | 15748555 | SNV | C | T | 1 | 25074 | Hetero | 32 | 63 | 50.8 | 0.46 | 0 | 30 | - | PTPRO |
| 12 | 16036148 | SNV | C | T | 1 | 25074 | Hetero | 63 | 153 | 41.2 | 0.26 | 0 | 105 | - | STRAP |
| 12 | 18836251 | SNV | A | G | 1 | 25074 | Hetero | 123 | 261 | 47.1 | 0.29 | 0 | 163 | No | PLCZ1 |
| 12 | 21242865 | SNV | T | G | 1 | 25074 | Hetero | 14 | 40 | 35.0 | 0.21 | 0 | 41 | Possible splice site disruption | SLCO1B3 |
| 12 | 29608226 | SNV | G | T | 1 | 25074 | Hetero | 54 | 125 | 43.2 | 0.33 | 0 | 96 | Yes | RP11-677C1.2 |
| 12 | 31648729 | Deletion | A | - | 1 | 25074 | Homo | 114 | 114 | 100.0 | 0.28 | 0 | 71 | - | DENND5B |
| 12 | 39047431 | SNV | G | A | 1 | 25074 | Hetero | 17 | 36 | 47.2 | 0.06 | 0 | 33 | - | CPNE8 |
| 12 | 40868943 | Deletion | G | - | 1 | 25074 | Hetero | 37 | 96 | 38.5 | 0.42 | 0 | 75 | Deletion | MUC19 |
| 12 | 45742339 | SNV | A | G | 1 | 25074 | Hetero | 27 | 197 | 13.7 | 0.31 | 0 | 170 | Yes | ANO6 |
| 12 | 46246442 | SNV | C | G | 1 | 25074 | Hetero | 93 | 232 | 40.1 | 0.45 | 0 | 181 | Yes | ARID2 |
| 12 | 51236870 | SNV | C | T | 1 | 25074 | Hetero | 55 | 221 | 24.9 | 0.22 | 0 | 136 | No | TMPRSS12 |
| 12 | 52913807 | SNV | C | A | 1 | 25074 | Hetero | 58 | 244 | 23.8 | 0.34 | 0 | 158 | Yes | KRT5 |
| 12 | 53012168 | SNV | G | C | 1 | 25074 | Hetero | 161 | 255 | 63.1 | 0.31 | 0 | 212 | Yes | KRT73 |
| 12 | 53579749 | SNV | G | A | 1 | 25074 | Hetero | 41 | 97 | 42.3 | 0.27 | 0 | 72 | No | ZNF740 |
| 12 | 53581342 | SNV | T | G | 1 | 25074 | Hetero | 150 | 449 | 33.4 | 0.44 | 0 | 259 | Yes | ZNF740 |
| 12 | 53720493 | SNV | G | C | 1 | 25074 | Hetero | 32 | 96 | 33.3 | 0.42 | 0 | 69 | - | SP7 |
| 12 | 56826758 | SNV | A | G | 1 | 25074 | Hetero | 33 | 106 | 31.1 | 0.39 | 0 | 66 | - | TIMELESS |
| 12 | 57036326 | SNV | G | C | 1 | 25074 | Hetero | 24 | 62 | 38.7 | 0.43 | 0 | 41 | No | ATP5B |
| 12 | 57081864 | SNV | C | T | 1 | 25074 | Hetero | 30 | 66 | 45.5 | 0.48 | 0 | 51 | - | PTGES3 |
| 12 | 57109860 | SNV | C | T | 1 | 25074 | Hetero | 37 | 141 | 26.2 | 0.37 | 0 | 96 | No | NACA |
| 12 | 57993020 | SNV | G | A | 1 | 25074 | Hetero | 30 | 90 | 33.3 | 0.24 | 0 | 53 | - | PIP4K2C |
| 12 | 65445246 | SNV | G | A | 1 | 25074 | Hetero | 103 | 150 | 68.7 | 0.30 | 0 | 140 | No | WIF1 |
| 12 | 66849263 | SNV | C | T | 1 | 25074 | Hetero | 61 | 82 | 74.4 | 0.24 | 0 | 67 | Yes | GRIP1 |
| 12 | 69969503 | SNV | A | G | 1 | 25074 | Hetero | 40 | 115 | 34.8 | 0.46 | 0 | 93 | - | FRS2 |
| 12 | 69980631 | SNV | A | G | 1 | 25074 | Hetero | 59 | 139 | 42.4 | 0.11 | 0 | 118 | - | CCT2 |
| 12 | 70070938 | SNV | T | C | 1 | 25074 | Hetero | 76 | 119 | 63.9 | 0.35 | 0 | 114 | - | BEST3 |
| 12 | 75436640 | SNV | T | C | 1 | 25074 | Hetero | 51 | 82 | 62.2 | 0.41 | 0 | 66 | - | KCNC2 |
| 12 | 76747085 | SNV | T | G | 1 | 25074 | Hetero | 44 | 74 | 59.5 | 0.48 | 0 | 54 | - | OSBPL8 |
| 12 | 77247216 | Deletion | T | - | 1 | 25074 | Hetero | 38 | 70 | 54.3 | 0.22 | 0 | 33 | - | ZDHHC17 |
| 12 | 78571503 | SNV | C | A | 1 | 25074 | Hetero | 55 | 121 | 45.5 | 0.27 | 0 | 104 | - | NAV3 |
| 12 | 104168482 | SNV | A | G | 1 | 25074 | Hetero | 66 | 103 | 64.1 | 0.48 | 0 | 65 | - | NT5DC3 |
| 12 | 106631674 | SNV | A | G | 1 | 25074 | Hetero | 29 | 61 | 47.5 | 0.44 | 0 | 46 | - | CKAP4 |
| 12 | 107712242 | SNV | C | G | 1 | 25074 | Hetero | 51 | 97 | 52.6 | 0.32 | 0 | 77 | - | BTBD11 |
| 12 | 108643095 | SNV | C | T | 1 | 25074 | Hetero | 40 | 95 | 42.1 | 0.41 | 0 | 73 | - | WSCD2 |
| 12 | 109017650 | SNV | G | C | 1 | 25074 | Hetero | 14 | 116 | 12.1 | 0.21 | 0 | 115 | Yes | SELPLG |
| 12 | 111800366 | SNV | G | A | 1 | 25074 | Hetero | 140 | 485 | 28.9 | 0.41 | 0 | 360 | Yes | FAM109A |
| 12 | 118854855 | SNV | G | T | 1 | 25074 | Hetero | 27 | 69 | 39.1 | 0.31 | 0 | 73 | - | SUDS3 |
| 12 | 120898146 | SNV | G | C | 1 | 25074 | Hetero | 52 | 93 | 55.9 | 0.47 | 0 | 61 | - | GATC |
| 12 | 121163633 | SNV | C | G | 1 | 25074 | Hetero | 15 | 43 | 34.9 | 0.44 | 0 | 33 | - | ACADS |
| 12 | 121202652 | SNV | C | T | 1 | 25074 | Hetero | 63 | 140 | 45.0 | 0.41 | 0 | 102 | - | SPPL3 |
| 12 | 122715756 | SNV | A | G | 1 | 25074 | Hetero | 36 | 72 | 50.0 | 0.45 | 0 | 43 | - | RP11-512M8.5 |
| 12 | 123082467 | SNV | T | C | 1 | 25074 | Hetero | 33 | 53 | 62.3 | 0.27 | 0 | 38 | No | KNTC1 |
| 12 | 123185864 | SNV | A | G | 1 | 25074 | Hetero | 102 | 510 | 20.0 | 0.25 | 0 | 230 | - | HCAR1 |
| 12 | 123463511 | SNV | G | C | 1 | 25074 | Hetero | 74 | 186 | 39.8 | 0.42 | 0 | 124 | Yes | ABCB9 |
| 12 | 123983948 | Deletion | A | - | 1 | 25074 | Hetero | 48 | 132 | 36.4 | 0.29 | 0 | 116 | - | RILPL1 |
| 12 | 124317867 | SNV | G | A | 1 | 25074 | Hetero | 88 | 233 | 37.8 | 0.40 | 0 | 191 | No | DNAH10 |
| 12 | 129558057 | SNV | T | A | 1 | 25074 | Hetero | 32 | 58 | 55.2 | 0.38 | 0 | 50 | - | TMEM132D |
| 12 | 132398256 | SNV | C | A | 1 | 25074 | Hetero | 11 | 80 | 13.8 | 0.43 | 0 | 48 | - | ULK1 |
| 12 | 132514460 | SNV | T | C | 1 | 25074 | Hetero | 25 | 53 | 47.2 | 0.42 | 0 | 62 | - | EP400 |
| 12 | 133104669 | SNV | A | G | 1 | 25074 | Hetero | 88 | 249 | 35.3 | 0.17 | 0 | 199 | - | FBRSL1 |
| 12 | 108682731 | Insertion | - | C | 1 | 25074 | Hetero | 31 | 88 | 35.2 | 0.25 | 0 | 99 | - | CMKLR1 |
| 12 | 110476873 | Insertion | - | T | 1 | 25074 | Hetero | 11 | 44 | 25.0 | 0.25 | 0 | 36 | - | ANKRD13A |
| 12 | 110479725 | Insertion | - | A | 1 | 25074 | Hetero | 12 | 37 | 32.4 | 0.33 | 0 | 46 | - | C12orf76 |
| 12 | 117014131 | Insertion | - | A | 1 | 25074 | Hetero | 98 | 243 | 40.3 | 0.48 | 0 | 250 | - | MAP1LC3B2 |
| 12 | 121647751 | Insertion | - | C | 1 | 25074 | Hetero | 20 | 55 | 36.4 | 0.32 | 0 | 46 | - | P2RX4 |
| 12 | 123103008 | Insertion | - | T | 1 | 25074 | Hetero | 48 | 143 | 33.6 | 0.43 | 0 | 106 | - | KNTC1 |
| 12 | 123199966 | Insertion | - | AA | 2 | 25074 | Hetero | 119 | 567 | 21.0 | 0.43 | 0 | 376 | - | HCAR1 |
| 12 | 14781678 | Insertion | - | A | 1 | 25074 | Homo | 89 | 95 | 93.7 | 0.37 | 0 | 41 | - | GUCY2C |
| 12 | 19410474 | Insertion | - | T | 1 | 25074 | Hetero | 29 | 79 | 36.7 | 0.28 | 0 | 41 | - | PLEKHA5 |
| 12 | 27132691 | Insertion | - | A | 1 | 25074 | Hetero | 50 | 91 | 54.9 | 0.26 | 0 | 57 | - | TM7SF3 |
| 12 | 27955071 | Insertion | - | T | 1 | 25074 | Hetero | 54 | 93 | 58.1 | 0.32 | 0 | 47 | - | KLHDC5 |
| 12 | 47182405 | Insertion | - | AC | 2 | 25074 | Hetero | 32 | 86 | 37.2 | 0.26 | 0 | 72 | - | SLC38A4 |
| 12 | 49312694 | Deletion | AG | - | 2 | 25074 | Homo | 70 | 71 | 98.6 | 0.27 | 0 | 41 | - | ARF3 |
| 12 | 49523524 | Deletion | AT | - | 2 | 25074 | Homo | 192 | 192 | 100.0 | 0.37 | 0 | 114 | - | TUBA1B |
| 12 | 50475997 | Deletion | TC | - | 2 | 25074 | Hetero | 55 | 133 | 41.4 | 0.43 | 0 | 94 | - | ASIC1 |
| 12 | 51374352 | Insertion | - | A | 1 | 25074 | Hetero | 20 | 76 | 26.3 | 0.25 | 0 | 61 | - | SLC11A2 |
| 12 | 52389473 | Insertion | - | A | 1 | 25074 | Hetero | 67 | 105 | 63.8 | 0.46 | 0 | 95 | - | ACVR1B |
| 12 | 52881260 | Insertion | - | AA | 2 | 25074 | Hetero | 31 | 47 | 66.0 | 0.49 | 0 | 31 | - | KRT6A |
| 12 | 56510943 | Deletion | AA | - | 2 | 25074 | Hetero | 16 | 77 | 20.8 | 0.11 | 0 | 31 | - | RPL41 |
| 12 | 65857611 | Insertion | - | T | 1 | 25074 | Hetero | 35 | 66 | 53.0 | 0.39 | 0 | 37 | - | MSRB3 |
| 12 | 89815097 | Insertion | - | TC | 2 | 25074 | Hetero | 55 | 82 | 67.1 | 0.29 | 0 | 47 | - | POC1B |
| 12 | 9009999 | Insertion | - | AA | 2 | 25074 | Hetero | 30 | 67 | 44.8 | 0.33 | 0 | 36 | - | A2ML1 |
| 12 | 96360224 | Insertion | - | CA | 2 | 25074 | Hetero | 76 | 125 | 60.8 | 0.44 | 0 | 84 | Insertion | AMDHD1 |
| 13 | 24466200 | SNV | C | T | 1 | 25074 | Hetero | 70 | 93 | 75.3 | 0.28 | 0 | 101 | Possible splice site disruption | C1QTNF9B-AS1 |
| 13 | 30339126 | Deletion | T | - | 1 | 25074 | Homo | 58 | 58 | 100.0 | 0.35 | 0 | 51 | - | UBL3 |
| 13 | 32976491 | SNV | G | T | 1 | 25074 | Hetero | 35 | 39 | 89.7 | 0.44 | 0 | 45 | - | N4BP2L1 |
| 13 | 51397436 | SNV | G | A | 1 | 25074 | Hetero | 209 | 248 | 84.3 | 0.38 | 0 | 222 | - | DLEU7 |
| 13 | 72013354 | SNV | C | A | 1 | 25074 | Hetero | 39 | 46 | 84.8 | 0.41 | 0 | 46 | - | DACH1 |
| 13 | 77564983 | SNV | T | C | 1 | 25074 | Hetero | 10 | 78 | 12.8 | 0.46 | 0 | 86 | - | CLN5 |
| 13 | 77565592 | SNV | A | G | 1 | 25074 | Hetero | 18 | 131 | 13.7 | 0.43 | 0 | 133 | - | CLN5 |
| 13 | 77792041 | SNV | A | C | 1 | 25074 | Hetero | 21 | 112 | 18.8 | 0.48 | 0 | 81 | No | MYCBP2 |
| 13 | 79190828 | SNV | T | A | 1 | 25074 | Hetero | 15 | 99 | 15.2 | 0.40 | 0 | 109 | No | RNF219 |
| 13 | 99853074 | SNV | C | T | 1 | 25074 | Hetero | 56 | 75 | 74.7 | 0.13 | 0 | 67 | - | UBAC2 |
| 13 | 111291256 | SNV | G | A | 1 | 25074 | Hetero | 109 | 128 | 85.2 | 0.44 | 0 | 121 | - | CARKD |
| 13 | 111291261 | Deletion | T | - | 1 | 25074 | Hetero | 106 | 125 | 84.8 | 0.45 | 0 | 122 | - | CARKD |
| 13 | 111291266 | SNV | T | C | 1 | 25074 | Hetero | 104 | 125 | 83.2 | 0.49 | 0 | 121 | - | CARKD |
| 13 | 103493063 | Insertion | - | T | 1 | 25074 | Homo | 53 | 54 | 98.1 | 0.31 | 0 | 37 | - | BIVM |
| 13 | 43358329 | Insertion | - | C | 1 | 25074 | Hetero | 11 | 70 | 15.7 | 0.15 | 0 | 59 | - | FAM216B |
| 13 | 51939527 | Insertion | - | T | 1 | 25074 | Homo | 69 | 73 | 94.5 | 0.40 | 0 | 67 | - | INTS6 |
| 14 | 21699167 | SNV | C | T | 1 | 25074 | Hetero | 113 | 214 | 52.8 | 0.34 | 0 | 130 | No | HNRNPC |
| 14 | 21827786 | SNV | A | T | 1 | 25074 | Hetero | 33 | 105 | 31.4 | 0.49 | 0 | 74 | - | SUPT16H |
| 14 | 22932018 | SNV | A | G | 1 | 25074 | Hetero | 191 | 338 | 56.5 | 0.49 | 0 | 214 | No | AE000661.37 |
| 14 | 24436702 | SNV | C | G | 1 | 25074 | Hetero | 39 | 362 | 10.8 | 0.24 | 0 | 223 | - | DHRS4-AS1 |
| 14 | 24473786 | SNV | C | A | 1 | 25074 | Hetero | 74 | 252 | 29.4 | 0.39 | 0 | 245 | - | DHRS4L2 |
| 14 | 31380216 | Deletion | A | - | 1 | 25074 | Homo | 39 | 40 | 97.5 | 0.14 | 0 | 31 | - | STRN3 |
| 14 | 38020342 | SNV | T | G | 1 | 25074 | Hetero | 58 | 100 | 58.0 | 0.44 | 0 | 55 | - | MIPOL1 |
| 14 | 52195211 | SNV | A | G | 1 | 25074 | Hetero | 29 | 61 | 47.5 | 0.44 | 0 | 50 | - | FRMD6 |
| 14 | 53162293 | SNV | G | A | 1 | 25074 | Hetero | 39 | 76 | 51.3 | 0.35 | 0 | 46 | - | ERO1L |
| 14 | 54950647 | SNV | C | T | 1 | 25074 | Hetero | 68 | 139 | 48.9 | 0.14 | 0 | 117 | - | GMFB |
| 14 | 58469891 | SNV | C | A | 1 | 25074 | Hetero | 53 | 93 | 57.0 | 0.39 | 0 | 71 | - | C14orf37 |
| 14 | 68191284 | SNV | A | G | 1 | 25074 | Hetero | 59 | 198 | 29.8 | 0.30 | 0 | 146 | Yes | RDH12 |
| 14 | 68264311 | SNV | G | A | 1 | 25074 | Hetero | 65 | 138 | 47.1 | 0.16 | 0 | 61 | - | ZFYVE26 |
| 14 | 69256418 | SNV | G | C | 1 | 25074 | Hetero | 82 | 168 | 48.8 | 0.49 | 0 | 88 | No | ZFP36L1 |
| 14 | 70478176 | SNV | C | T | 1 | 25074 | Hetero | 77 | 159 | 48.4 | 0.41 | 0 | 104 | - | SMOC1 |
| 14 | 70936840 | SNV | C | T | 1 | 25074 | Hetero | 34 | 143 | 23.8 | 0.47 | 0 | 77 | - | ADAM20P1 |
| 14 | 71209232 | SNV | C | T | 1 | 25074 | Hetero | 62 | 246 | 25.2 | 0.35 | 0 | 164 | Yes | MAP3K9 |
| 14 | 71578552 | SNV | A | T | 1 | 25074 | Hetero | 35 | 90 | 38.9 | 0.33 | 0 | 76 | - | PCNX |
| 14 | 73689515 | SNV | T | C | 1 | 25074 | Hetero | 54 | 103 | 52.4 | 0.46 | 0 | 54 | - | PSEN1 |
| 14 | 73973190 | SNV | A | C | 1 | 25074 | Hetero | 42 | 85 | 49.4 | 0.38 | 0 | 37 | Yes | HEATR4 |
| 14 | 74428049 | SNV | G | A | 1 | 25074 | Hetero | 294 | 589 | 49.9 | 0.44 | 0 | 365 | No | COQ6 |
| 14 | 77249053 | SNV | T | C | 1 | 25074 | Hetero | 79 | 150 | 52.7 | 0.45 | 0 | 102 | - | VASH1 |
| 14 | 77807274 | Deletion | A | - | 1 | 25074 | Hetero | 9 | 64 | 14.1 | 0.11 | 0 | 42 | - | TMED8 |
| 14 | 79111653 | SNV | C | T | 1 | 25074 | Hetero | 56 | 109 | 51.4 | 0.39 | 0 | 72 | No | NRXN3 |
| 14 | 80329871 | SNV | A | C | 1 | 25074 | Hetero | 28 | 56 | 50.0 | 0.45 | 0 | 45 | - | NRXN3 |
| 14 | 81574839 | Deletion | T | - | 1 | 25074 | Hetero | 50 | 282 | 17.7 | 0.18 | 0 | 166 | - | TSHR |
| 14 | 91699651 | SNV | T | A | 1 | 25074 | Hetero | 86 | 155 | 55.5 | 0.43 | 0 | 75 | - | GPR68 |
| 14 | 92264081 | Deletion | A | - | 1 | 25074 | Hetero | 17 | 81 | 21.0 | 0.11 | 0 | 39 | - | TC2N |
| 14 | 93151917 | SNV | C | T | 1 | 25074 | Hetero | 53 | 132 | 40.2 | 0.31 | 0 | 83 | - | RIN3 |
| 14 | 95570254 | SNV | G | T | 1 | 25074 | Hetero | 103 | 240 | 42.9 | 0.48 | 0 | 189 | Yes | DICER1 |
| 14 | 102516867 | SNV | C | T | 1 | 25074 | Hetero | 115 | 213 | 54.0 | 0.31 | 0 | 92 | No | DYNC1H1 |
| 14 | 104198931 | SNV | C | T | 1 | 25074 | Hetero | 49 | 111 | 44.1 | 0.45 | 0 | 56 | - | ZFYVE21 |
| 14 | 104431780 | SNV | C | T | 1 | 25074 | Hetero | 15 | 128 | 11.7 | 0.42 | 0 | 61 | No | TDRD9 |
| 14 | 105055914 | SNV | C | T | 1 | 25074 | Hetero | 172 | 343 | 50.1 | 0.48 | 0 | 159 | - | C14orf180 |
| 14 | 105071471 | SNV | T | C | 1 | 25074 | Hetero | 141 | 281 | 50.2 | 0.29 | 0 | 146 | - | TMEM179 |
| 14 | 105350846 | SNV | A | G | 1 | 25074 | Hetero | 33 | 72 | 45.8 | 0.42 | 0 | 51 | Yes | KIAA0284 |
| 14 | 105418744 | SNV | T | C | 1 | 25074 | Hetero | 112 | 219 | 51.1 | 0.47 | 0 | 142 | Yes | AHNAK2 |
| 14 | 105879999 | SNV | G | A | 1 | 25074 | Hetero | 116 | 223 | 52.0 | 0.46 | 0 | 126 | - | TEX22 |
| 14 | 102550069 | Insertion | - | A | 1 | 25074 | Hetero | 15 | 114 | 13.2 | 0.47 | 0 | 53 | - | HSP90AA1 |
| 14 | 23731855 | Insertion | - | T | 1 | 25074 | Hetero | 44 | 117 | 37.6 | 0.33 | 0 | 97 | - | C14orf164 |
| 14 | 31029317 | Insertion | - | T | 1 | 25074 | Hetero | 25 | 81 | 30.9 | 0.44 | 0 | 46 | - | RP11-1103G16.1 |
| 14 | 52195089 | Insertion | - | T | 1 | 25074 | Hetero | 68 | 114 | 59.6 | 0.25 | 0 | 61 | - | FRMD6 |
| 14 | 56764487 | Insertion | - | T | 1 | 25074 | Hetero | 65 | 112 | 58.0 | 0.45 | 0 | 63 | - | PELI2 |
| 14 | 63779846 | Insertion | - | A | 1 | 25074 | Homo | 113 | 113 | 100.0 | 0.16 | 0 | 67 | - | GPHB5 |
| 14 | 65267609 | Insertion | - | T | 1 | 25074 | Hetero | 71 | 156 | 45.5 | 0.49 | 0 | 73 | - | SPTB |
| 14 | 71540242 | Insertion | - | CT | 2 | 25074 | Hetero | 46 | 109 | 42.2 | 0.13 | 0 | 70 | - | PCNX |
| 14 | 73014090 | Insertion | - | CG | 2 | 25074 | Hetero | 25 | 72 | 34.7 | 0.15 | 0 | 42 | - | RGS6 |
| 14 | 74525299 | Insertion | - | T | 1 | 25074 | Hetero | 27 | 94 | 28.7 | 0.43 | 0 | 69 | - | C14orf45 |
| 14 | 74667520 | Insertion | - | TA | 2 | 25074 | Hetero | 29 | 73 | 39.7 | 0.24 | 0 | 35 | - | LIN52 |
| 14 | 75128309 | Deletion | AA | - | 2 | 25074 | Hetero | 35 | 117 | 29.9 | 0.43 | 0 | 66 | - | KIAA0317 |
| 14 | 76619282 | Insertion | - | T | 1 | 25074 | Hetero | 54 | 122 | 44.3 | 0.48 | 0 | 57 | - | C14orf118 |
| 14 | 76662376 | Insertion | - | T | 1 | 25074 | Hetero | 43 | 114 | 37.7 | 0.24 | 0 | 66 | Insertion | C14orf118 |
| 14 | 92588155 | Insertion | - | T | 1 | 25074 | Hetero | 36 | 254 | 14.2 | 0.41 | 0 | 168 | - | NDUFB1 |
| 15 | 20464748 | SNV | A | C | 1 | 25074 | Hetero | 20 | 188 | 10.6 | 0.15 | 0 | 239 | - | RP11-492D6.3 |
| 15 | 20464792 | SNV | G | A | 1 | 25074 | Hetero | 58 | 240 | 24.2 | 0.48 | 0 | 316 | - | RP11-492D6.3 |
| 15 | 20466465 | SNV | C | A | 1 | 25074 | Hetero | 12 | 76 | 15.8 | 0.40 | 0 | 109 | - | RP11-492D6.3 |
| 15 | 24926815 | SNV | T | C | 1 | 25074 | Hetero | 29 | 35 | 82.9 | 0.24 | 0 | 45 | - | NPAP1 |
| 15 | 34657213 | SNV | A | G | 1 | 25074 | Hetero | 9 | 58 | 15.5 | 0.11 | 0 | 63 | No | LPCAT4 |
| 15 | 38780595 | Deletion | T | - | 1 | 25074 | Hetero | 23 | 54 | 42.6 | 0.32 | 0 | 42 | - | RASGRP1 |
| 15 | 40650547 | SNV | A | G | 1 | 25074 | Hetero | 8 | 32 | 25.0 | 0.30 | 0 | 32 | Yes | DISP2 |
| 15 | 40831597 | Deletion | A | - | 1 | 25074 | Hetero | 100 | 125 | 80.0 | 0.42 | 0 | 122 | - | C15orf57 |
| 15 | 41277662 | SNV | G | A | 1 | 25074 | Hetero | 14 | 77 | 18.2 | 0.41 | 0 | 45 | - | INO80 |
| 15 | 42192772 | SNV | C | T | 1 | 25074 | Homo | 67 | 67 | 100.0 | 0.47 | 0 | 50 | - | EHD4 |
| 15 | 42470373 | Deletion | C | - | 1 | 25074 | Hetero | 70 | 87 | 80.5 | 0.22 | 0 | 62 | - | VPS39 |
| 15 | 42860878 | SNV | C | G | 1 | 25074 | Hetero | 9 | 42 | 21.4 | 0.42 | 0 | 30 | - | HAUS2 |
| 15 | 43010268 | SNV | G | A | 1 | 25074 | Hetero | 50 | 57 | 87.7 | 0.35 | 0 | 57 | No | STARD9 |
| 15 | 44486926 | SNV | C | T | 1 | 25074 | Hetero | 59 | 67 | 88.1 | 0.22 | 0 | 39 | - | FRMD5 |
| 15 | 45365905 | SNV | G | C | 1 | 25074 | Hetero | 36 | 268 | 13.4 | 0.48 | 0 | 298 | - | SORD |
| 15 | 48065850 | SNV | T | A | 1 | 25074 | Hetero | 23 | 33 | 69.7 | 0.38 | 0 | 52 | - | SEMA6D |
| 15 | 51535156 | SNV | A | G | 1 | 25074 | Hetero | 157 | 195 | 80.5 | 0.46 | 0 | 223 | - | CYP19A1 |
| 15 | 52901920 | SNV | A | G | 1 | 25074 | Hetero | 155 | 201 | 77.1 | 0.49 | 0 | 190 | No | FAM214A |
| 15 | 53808462 | SNV | T | C | 1 | 25074 | Hetero | 46 | 61 | 75.4 | 0.45 | 0 | 55 | - | WDR72 |
| 15 | 56992715 | Deletion | T | - | 1 | 25074 | Hetero | 77 | 94 | 81.9 | 0.39 | 0 | 104 | - | ZNF280D |
| 15 | 57540300 | SNV | C | G | 1 | 25074 | Hetero | 92 | 125 | 73.6 | 0.42 | 0 | 160 | - | TCF12 |
| 15 | 59981659 | SNV | G | A | 1 | 25074 | Hetero | 17 | 105 | 16.2 | 0.47 | 0 | 113 | - | BNIP2 |
| 15 | 64968438 | SNV | A | G | 1 | 25074 | Hetero | 13 | 77 | 16.9 | 0.50 | 0 | 80 | Yes | ZNF609 |
| 15 | 65620655 | SNV | T | A | 1 | 25074 | Hetero | 19 | 30 | 63.3 | 0.32 | 0 | 33 | - | IGDCC3 |
| 15 | 65948444 | SNV | G | A | 1 | 25074 | Homo | 71 | 71 | 100.0 | 0.46 | 0 | 75 | - | SLC24A1 |
| 15 | 65959942 | SNV | C | G | 1 | 25074 | Hetero | 22 | 117 | 18.8 | 0.46 | 0 | 105 | Yes | DENND4A |
| 15 | 66977918 | SNV | T | A | 1 | 25074 | Hetero | 83 | 100 | 83.0 | 0.50 | 0 | 82 | - | RP11-321F6.1 |
| 15 | 71144095 | SNV | G | A | 1 | 25074 | Hetero | 34 | 252 | 13.5 | 0.34 | 0 | 285 | - | LARP6 |
| 15 | 72072186 | Deletion | T | - | 1 | 25074 | Hetero | 6 | 58 | 10.3 | 0.43 | 0 | 64 | - | THSD4 |
| 15 | 73852351 | SNV | C | A | 1 | 25074 | Hetero | 55 | 63 | 87.3 | 0.22 | 0 | 58 | - | C15orf60 |
| 15 | 74421035 | SNV | G | A | 1 | 25074 | Hetero | 69 | 97 | 71.1 | 0.38 | 0 | 103 | Yes | ISLR2 |
| 15 | 75130955 | SNV | C | T | 1 | 25074 | Hetero | 37 | 162 | 22.8 | 0.36 | 0 | 107 | - | ULK3 |
| 15 | 75782545 | SNV | C | T | 1 | 25074 | Hetero | 10 | 66 | 15.2 | 0.36 | 0 | 69 | - | PTPN9 |
| 15 | 76507923 | SNV | C | T | 1 | 25074 | Hetero | 24 | 151 | 15.9 | 0.50 | 0 | 162 | - | C15orf27 |
| 15 | 86118477 | SNV | G | A | 1 | 25074 | Hetero | 56 | 70 | 80.0 | 0.32 | 0 | 61 | Yes | AKAP13 |
| 15 | 89388900 | SNV | G | A | 1 | 25074 | Hetero | 36 | 207 | 17.4 | 0.46 | 0 | 192 | Yes | ACAN |
| 15 | 98516502 | SNV | C | T | 1 | 25074 | Hetero | 43 | 49 | 87.8 | 0.38 | 0 | 79 | - | ARRDC4 |
| 15 | 99192604 | SNV | C | A | 1 | 25074 | Hetero | 9 | 51 | 17.6 | 0.11 | 0 | 56 | - | IGF1R |
| 15 | 100693032 | Insertion | - | TT | 2 | 25074 | Hetero | 80 | 107 | 74.8 | 0.11 | 0 | 82 | - | ADAMTS17 |
| 15 | 41149968 | Insertion | - | TC | 2 | 25074 | Hetero | 11 | 57 | 19.3 | 0.33 | 0 | 65 | - | SPINT1 |
| 15 | 48527542 | Insertion | - | A | 1 | 25074 | Hetero | 24 | 89 | 27.0 | 0.43 | 0 | 95 | - | SLC12A1 |
| 15 | 55835273 | Insertion | - | T | 1 | 25074 | Hetero | 15 | 30 | 50.0 | 0.31 | 0 | 30 | - | PYGO1 |
| 15 | 63559888 | Insertion | - | T | 1 | 25074 | Hetero | 74 | 91 | 81.3 | 0.27 | 0 | 76 | - | RAB8B |
| 15 | 78911038 | Insertion | - | A | 1 | 25074 | Hetero | 21 | 105 | 20.0 | 0.22 | 0 | 93 | - | CHRNA3 |
| 15 | 96881004 | Insertion | - | A | 1 | 25074 | Hetero | 16 | 56 | 28.6 | 0.24 | 0 | 70 | - | NR2F2 |
| 15 | 96881050 | Insertion | - | A | 1 | 25074 | Hetero | 15 | 72 | 20.8 | 0.40 | 0 | 67 | - | NR2F2 |
| 16 | 615296 | SNV | G | A | 1 | 25074 | Hetero | 33 | 81 | 40.7 | 0.30 | 0 | 37 | Yes | C16orf11 |
| 16 | 719489 | SNV | C | T | 1 | 25074 | Hetero | 37 | 74 | 50.0 | 0.17 | 0 | 39 | - | MSLN |
| 16 | 1306718 | SNV | C | G | 1 | 25074 | Hetero | 12 | 40 | 30.0 | 0.17 | 0 | 31 | - | TPSD1 |
| 16 | 1371338 | SNV | T | C | 1 | 25074 | Hetero | 102 | 206 | 49.5 | 0.41 | 0 | 139 | - | UBE2I |
| 16 | 1706784 | SNV | G | A | 1 | 25074 | Hetero | 99 | 201 | 49.3 | 0.41 | 0 | 95 | Yes | CRAMP1L |
| 16 | 1876845 | SNV | C | A | 1 | 25074 | Hetero | 80 | 156 | 51.3 | 0.36 | 0 | 63 | - | HAGH |
| 16 | 1877675 | SNV | T | G | 1 | 25074 | Hetero | 95 | 199 | 47.7 | 0.38 | 0 | 117 | Yes | FAHD1 |
| 16 | 2124249 | SNV | A | G | 1 | 25074 | Hetero | 25 | 59 | 42.4 | 0.21 | 0 | 35 | Yes | TSC2 |
| 16 | 2227385 | SNV | C | T | 1 | 25074 | Hetero | 43 | 84 | 51.2 | 0.36 | 0 | 48 | - | TRAF7 |
| 16 | 2261210 | SNV | T | C | 1 | 25074 | Hetero | 58 | 113 | 51.3 | 0.31 | 0 | 60 | - | C16orf79 |
| 16 | 2285163 | SNV | G | A | 1 | 25074 | Hetero | 119 | 229 | 52.0 | 0.48 | 0 | 106 | - | E4F1 |
| 16 | 3024201 | SNV | G | A | 1 | 25074 | Hetero | 48 | 96 | 50.0 | 0.31 | 0 | 41 | - | PKMYT1 |
| 16 | 3573319 | SNV | T | C | 1 | 25074 | Hetero | 71 | 137 | 51.8 | 0.31 | 0 | 77 | - | CLUAP1 |
| 16 | 8875575 | SNV | T | A | 1 | 25074 | Hetero | 79 | 187 | 42.2 | 0.40 | 0 | 104 | - | ABAT |
| 16 | 8877515 | SNV | T | C | 1 | 25074 | Hetero | 63 | 134 | 47.0 | 0.46 | 0 | 93 | - | ABAT |
| 16 | 12536864 | SNV | G | T | 1 | 25074 | Hetero | 30 | 68 | 44.1 | 0.41 | 0 | 44 | Yes | SNX29 |
| 16 | 20320961 | Deletion | A | - | 1 | 25074 | Hetero | 31 | 62 | 50.0 | 0.16 | 0 | 40 | - | GP2 |
| 16 | 20360338 | SNV | G | A | 1 | 25074 | Hetero | 30 | 158 | 19.0 | 0.47 | 0 | 104 | No | UMOD |
| 16 | 20361214 | Deletion | A | - | 1 | 25074 | Hetero | 30 | 86 | 34.9 | 0.10 | 0 | 49 | - | UMOD |
| 16 | 21108745 | SNV | T | G | 1 | 25074 | Hetero | 48 | 91 | 52.7 | 0.40 | 0 | 41 | Yes | DNAH3 |
| 16 | 21548095 | SNV | G | A | 1 | 25074 | Hetero | 11 | 48 | 22.9 | 0.23 | 0 | 51 | - | CTB-31N19.2 |
| 16 | 22444123 | SNV | G | A | 1 | 25074 | Hetero | 12 | 75 | 16.0 | 0.25 | 0 | 49 | - | CDR2 |
| 16 | 22444186 | SNV | A | C | 1 | 25074 | Hetero | 35 | 84 | 41.7 | 0.11 | 0 | 33 | - | CDR2 |
| 16 | 22580572 | SNV | G | T | 1 | 25074 | Hetero | 20 | 82 | 24.4 | 0.30 | 0 | 147 | - | RP11-368J21.3 |
| 16 | 23457223 | SNV | T | C | 1 | 25074 | Hetero | 84 | 153 | 54.9 | 0.43 | 0 | 112 | Yes | COG7 |
| 16 | 24950765 | SNV | C | T | 1 | 25074 | Hetero | 13 | 69 | 18.8 | 0.08 | 0 | 40 | No | ARHGAP17 |
| 16 | 26036679 | SNV | C | T | 1 | 25074 | Hetero | 111 | 235 | 47.2 | 0.45 | 0 | 157 | - | HS3ST4 |
| 16 | 28332544 | Deletion | A | - | 1 | 25074 | Hetero | 13 | 108 | 12.0 | 0.46 | 0 | 54 | - | SBK1 |
| 16 | 30035873 | SNV | G | A | 1 | 25074 | Hetero | 144 | 268 | 53.7 | 0.47 | 0 | 117 | - | C16orf92 |
| 16 | 30670496 | SNV | G | C | 1 | 25074 | Hetero | 67 | 129 | 51.9 | 0.41 | 0 | 73 | - | FBRS |
| 16 | 46657971 | SNV | G | A | 1 | 25074 | Hetero | 9 | 43 | 20.9 | 0.36 | 0 | 78 | - | RP11-46D6.1 |
| 16 | 55857480 | SNV | C | T | 1 | 25074 | Hetero | 30 | 263 | 11.4 | 0.16 | 0 | 200 | Yes | CES1 |
| 16 | 55905615 | SNV | C | T | 1 | 25074 | Hetero | 98 | 197 | 49.7 | 0.36 | 0 | 153 | No | CES5A |
| 16 | 57951272 | SNV | T | C | 1 | 25074 | Hetero | 51 | 106 | 48.1 | 0.50 | 0 | 76 | Yes | CNGB1 |
| 16 | 67218384 | SNV | G | A | 1 | 25074 | Hetero | 103 | 205 | 50.2 | 0.14 | 0 | 109 | Yes | EXOC3L1 |
| 16 | 67859772 | SNV | G | C | 1 | 25074 | Hetero | 71 | 164 | 43.3 | 0.35 | 0 | 93 | - | TSNAXIP1 |
| 16 | 67981898 | SNV | C | A | 1 | 25074 | Hetero | 116 | 219 | 53.0 | 0.21 | 0 | 94 | - | SLC12A4 |
| 16 | 70163691 | SNV | C | T | 1 | 25074 | Hetero | 68 | 270 | 25.2 | 0.48 | 0 | 102 | - | PDPR |
| 16 | 71065363 | SNV | T | C | 1 | 25074 | Hetero | 29 | 128 | 22.7 | 0.34 | 0 | 64 | - | HYDIN |
| 16 | 71100712 | SNV | G | A | 1 | 25074 | Hetero | 120 | 429 | 28.0 | 0.28 | 0 | 304 | - | HYDIN |
| 16 | 71165560 | SNV | C | T | 1 | 25074 | Hetero | 51 | 187 | 27.3 | 0.47 | 0 | 128 | - | HYDIN |
| 16 | 71166771 | SNV | G | A | 1 | 25074 | Hetero | 24 | 105 | 22.9 | 0.44 | 0 | 85 | - | HYDIN |
| 16 | 74366531 | SNV | A | C | 1 | 25074 | Hetero | 28 | 189 | 14.8 | 0.33 | 0 | 55 | - | AC009120.6 |
| 16 | 74452050 | SNV | A | G | 1 | 25074 | Hetero | 7 | 42 | 16.7 | 0.44 | 0 | 55 | No | CLEC18B |
| 16 | 75148657 | SNV | C | T | 1 | 25074 | Hetero | 111 | 230 | 48.3 | 0.44 | 0 | 117 | - | LDHD |
| 16 | 77465363 | SNV | C | A | 1 | 25074 | Hetero | 29 | 147 | 19.7 | 0.41 | 0 | 115 | No | ADAMTS18 |
| 16 | 79634233 | SNV | C | T | 1 | 25074 | Hetero | 21 | 57 | 36.8 | 0.22 | 0 | 40 | - | MAF |
| 16 | 83983889 | SNV | T | C | 1 | 25074 | Hetero | 41 | 68 | 60.3 | 0.36 | 0 | 38 | - | RP11-505K9.4 |
| 16 | 84135364 | SNV | G | A | 1 | 25074 | Hetero | 255 | 559 | 45.6 | 0.43 | 0 | 436 | Yes | MBTPS1 |
| 16 | 87448829 | SNV | G | C | 1 | 25074 | Hetero | 114 | 247 | 46.2 | 0.37 | 0 | 170 | - | ZCCHC14 |
| 16 | 87921674 | SNV | T | A | 1 | 25074 | Hetero | 152 | 333 | 45.6 | 0.39 | 0 | 170 | - | CA5A |
| 16 | 88778586 | SNV | G | A | 1 | 25074 | Hetero | 141 | 263 | 53.6 | 0.46 | 0 | 103 | Yes | CTU2 |
| 16 | 88876539 | SNV | C | T | 1 | 25074 | Hetero | 59 | 127 | 46.5 | 0.27 | 0 | 75 | No | APRT |
| 16 | 89775284 | SNV | G | T | 1 | 25074 | Hetero | 32 | 64 | 50.0 | 0.35 | 0 | 31 | Yes | C16orf7 |
| 16 | 89831363 | SNV | T | C | 1 | 25074 | Hetero | 48 | 285 | 16.8 | 0.48 | 0 | 125 | Yes | FANCA |
| 16 | 15155386 | Deletion | AT | - | 2 | 25074 | Hetero | 81 | 159 | 50.9 | 0.37 | 0 | 89 | - | PDXDC1 |
| 16 | 15680717 | Insertion | - | C | 1 | 25074 | Hetero | 19 | 80 | 23.8 | 0.46 | 0 | 47 | - | C16orf45 |
| 16 | 19548843 | Insertion | - | A | 1 | 25074 | Hetero | 34 | 52 | 65.4 | 0.16 | 0 | 56 | Insertion | CCP110 |
| 16 | 23075055 | Insertion | - | T | 1 | 25074 | Hetero | 42 | 115 | 36.5 | 0.45 | 0 | 72 | - | USP31 |
| 16 | 23079234 | Insertion | - | CA | 2 | 25074 | Hetero | 14 | 48 | 29.2 | 0.19 | 0 | 36 | - | USP31 |
| 16 | 29705974 | Insertion | - | T | 1 | 25074 | Hetero | 54 | 116 | 46.6 | 0.24 | 0 | 67 | - | AC009133.19 |
| 16 | 30565788 | Insertion | - | CA | 2 | 25074 | Hetero | 86 | 250 | 34.4 | 0.37 | 0 | 146 | - | AC002310.13 |
| 16 | 58577315 | Insertion | - | A | 1 | 25074 | Hetero | 7 | 69 | 10.1 | 0.38 | 0 | 49 | Insertion | CNOT1 |
| 16 | 69599949 | Insertion | - | C | 1 | 25074 | Hetero | 263 | 528 | 49.8 | 0.49 | 0 | 281 | - | NFAT5 |
| 16 | 70176607 | Deletion | AA | - | 2 | 25074 | Hetero | 29 | 174 | 16.7 | 0.13 | 0 | 105 | - | PDPR |
| 16 | 72120084 | Deletion | GG | - | 2 | 25074 | Hetero | 7 | 58 | 12.1 | 0.29 | 0 | 39 | - | TXNL4B |
| 16 | 77225217 | Insertion | - | G | 1 | 25074 | Hetero | 59 | 131 | 45.0 | 0.35 | 0 | 89 | - | MON1B |
| 16 | 84213541 | Insertion | - | C | 1 | 25074 | Hetero | 101 | 237 | 42.6 | 0.49 | 0 | 152 | - | TAF1C |
| 16 | 85706942 | Insertion | - | A | 1 | 25074 | Hetero | 26 | 66 | 39.4 | 0.29 | 0 | 35 | - | KIAA0182 |
| 16 | 8947197 | Insertion | - | A | 1 | 25074 | Homo | 130 | 130 | 100.0 | 0.49 | 0 | 107 | - | CARHSP1 |
| 17 | 915771 | SNV | C | T | 1 | 25074 | Homo | 243 | 267 | 91.0 | 0.31 | 0 | 179 | - | ABR |
| 17 | 1552877 | SNV | C | T | 1 | 25074 | Hetero | 23 | 184 | 12.5 | 0.40 | 0 | 65 | No | RILP |
| 17 | 1641171 | SNV | A | C | 1 | 25074 | Hetero | 13 | 82 | 15.9 | 0.23 | 0 | 58 | - | WDR81 |
| 17 | 7577559 | SNV | G | A | 1 | 25074 | Hetero | 76 | 105 | 72.4 | 0.49 | 0 | 44 | Yes | TP53 |
| 17 | 7590297 | SNV | T | C | 1 | 25074 | Hetero | 81 | 96 | 84.4 | 0.40 | 0 | 52 | - | TP53 |
| 17 | 7851523 | SNV | T | A | 1 | 25074 | Homo | 164 | 184 | 89.1 | 0.49 | 0 | 110 | Yes | CNTROB |
| 17 | 8076918 | SNV | G | C | 1 | 25074 | Hetero | 215 | 244 | 88.1 | 0.46 | 0 | 134 | - | TMEM107 |
| 17 | 8132574 | SNV | C | T | 1 | 25074 | Hetero | 21 | 131 | 16.0 | 0.42 | 0 | 61 | - | CTC1 |
| 17 | 10404875 | SNV | A | T | 1 | 25074 | Hetero | 175 | 212 | 82.5 | 0.32 | 0 | 142 | - | MYH2 |
| 17 | 10541169 | SNV | C | G | 1 | 25074 | Hetero | 67 | 87 | 77.0 | 0.42 | 0 | 63 | No | MYH3 |
| 17 | 14079163 | SNV | G | A | 1 | 25074 | Hetero | 11 | 106 | 10.4 | 0.18 | 0 | 180 | - | COX10 |
| 17 | 16594048 | SNV | A | G | 1 | 25074 | Hetero | 69 | 78 | 88.5 | 0.45 | 0 | 64 | Yes | CCDC144A |
| 17 | 17071008 | SNV | C | T | 1 | 25074 | Hetero | 12 | 70 | 17.1 | 0.23 | 0 | 48 | Yes | MPRIP |
| 17 | 30326166 | SNV | A | G | 1 | 25074 | Hetero | 88 | 98 | 89.8 | 0.49 | 0 | 77 | - | SUZ12 |
| 17 | 30349265 | SNV | A | G | 1 | 25074 | Hetero | 14 | 126 | 11.1 | 0.36 | 0 | 96 | Yes | LRRC37B |
| 17 | 39921082 | Deletion | A | - | 1 | 25074 | Hetero | 11 | 104 | 10.6 | 0.25 | 0 | 44 | - | JUP |
| 17 | 41345661 | SNV | C | T | 1 | 25074 | Hetero | 9 | 79 | 11.4 | 0.36 | 0 | 48 | - | NBR1 |
| 17 | 44103308 | SNV | A | G | 1 | 25074 | Hetero | 142 | 172 | 82.6 | 0.36 | 0 | 105 | - | MAPT |
| 17 | 44103345 | SNV | G | T | 1 | 25074 | Hetero | 111 | 136 | 81.6 | 0.40 | 0 | 91 | - | MAPT |
| 17 | 46627262 | Deletion | A | - | 1 | 25074 | Homo | 123 | 124 | 99.2 | 0.41 | 0 | 56 | - | HOXB-AS1 |
| 17 | 47038266 | SNV | G | C | 1 | 25074 | Hetero | 20 | 150 | 13.3 | 0.48 | 0 | 76 | Yes | GIP |
| 17 | 48913377 | SNV | G | A | 1 | 25074 | Homo | 227 | 252 | 90.1 | 0.33 | 0 | 144 | Yes | WFIKKN2 |
| 17 | 54588484 | SNV | G | A | 1 | 25074 | Hetero | 87 | 104 | 83.7 | 0.41 | 0 | 51 | Yes | ANKFN1 |
| 17 | 62232243 | SNV | C | G | 1 | 25074 | Hetero | 80 | 94 | 85.1 | 0.21 | 0 | 54 | Yes | TEX2 |
| 17 | 66036943 | SNV | T | G | 1 | 25074 | Hetero | 26 | 182 | 14.3 | 0.48 | 0 | 97 | - | KPNA2 |
| 17 | 72768527 | SNV | T | C | 1 | 25074 | Hetero | 21 | 74 | 28.4 | 0.22 | 0 | 48 | Yes | NAT9 |
| 17 | 72832788 | SNV | G | A | 1 | 25074 | Homo | 303 | 336 | 90.2 | 0.49 | 0 | 176 | Yes | TMEM104 |
| 17 | 74077568 | SNV | C | T | 1 | 25074 | Hetero | 31 | 284 | 10.9 | 0.22 | 0 | 139 | - | ZACN |
| 17 | 78187720 | SNV | A | G | 1 | 25074 | Homo | 257 | 257 | 100.0 | 0.28 | 0 | 157 | - | SGSH |
| 17 | 78246996 | SNV | C | T | 1 | 25074 | Hetero | 111 | 128 | 86.7 | 0.23 | 0 | 56 | - | RNF213 |
| 17 | 80332349 | SNV | C | T | 1 | 25074 | Hetero | 290 | 324 | 89.5 | 0.46 | 0 | 144 | Yes | UTS2R |
| 17 | 80438458 | SNV | C | T | 1 | 25074 | Hetero | 12 | 77 | 15.6 | 0.08 | 0 | 51 | - | NARF |
| 17 | 80615617 | SNV | G | A | 1 | 25074 | Hetero | 180 | 209 | 86.1 | 0.38 | 0 | 130 | - | RAB40B |
| 17 | 27046781 | Insertion | - | A | 1 | 25074 | Hetero | 189 | 224 | 84.4 | 0.19 | 0 | 104 | - | RPL23A |
| 17 | 27050924 | Insertion | - | AT | 2 | 25074 | Hetero | 91 | 133 | 68.4 | 0.31 | 0 | 88 | - | RPL23A |
| 17 | 33479823 | Insertion | - | C | 1 | 25074 | Homo | 60 | 66 | 90.9 | 0.19 | 0 | 38 | - | UNC45B |
| 17 | 3564711 | Insertion | - | G | 1 | 25074 | Hetero | 59 | 126 | 46.8 | 0.49 | 0 | 131 | - | CTNS |
| 17 | 37690050 | Insertion | - | A | 1 | 25074 | Hetero | 22 | 123 | 17.9 | 0.26 | 0 | 60 | - | CDK12 |
| 17 | 45567594 | Deletion | TC | - | 2 | 25074 | Homo | 176 | 176 | 100.0 | 0.41 | 0 | 116 | - | MRPL45P2 |
| 17 | 53014442 | Insertion | - | T | 1 | 25074 | Hetero | 55 | 122 | 45.1 | 0.33 | 0 | 66 | - | TOM1L1 |
| 17 | 59486437 | Insertion | - | A | 1 | 25074 | Hetero | 87 | 113 | 77.0 | 0.48 | 0 | 66 | - | TBX2 |
| 17 | 67512924 | Insertion | - | TT | 2 | 25074 | Homo | 160 | 160 | 100.0 | 0.16 | 0 | 143 | - | MAP2K6 |
| 17 | 70121696 | Deletion | TT | - | 2 | 25074 | Hetero | 7 | 54 | 13.0 | 0.38 | 0 | 38 | - | SOX9 |
| 17 | 7147350 | Insertion | - | C | 1 | 25074 | Hetero | 17 | 160 | 10.6 | 0.12 | 0 | 94 | - | CTDNEP1 |
| 17 | 73897033 | Insertion | - | C | 1 | 25074 | Hetero | 231 | 266 | 86.8 | 0.48 | 0 | 162 | - | MRPL38 |
| 17 | 76127849 | Insertion | - | G | 1 | 25074 | Hetero | 146 | 175 | 83.4 | 0.43 | 0 | 93 | - | TMC6 |
| 17 | 78187722 | Deletion | AG | - | 2 | 25074 | Homo | 257 | 257 | 100.0 | 0.29 | 0 | 155 | - | SGSH |
| 17 | 79390345 | Insertion | - | TA | 2 | 25074 | Hetero | 10 | 78 | 12.8 | 0.36 | 0 | 54 | - | BAHCC1 |
| 17 | 81050833 | Insertion | - | G | 1 | 25074 | Homo | 244 | 244 | 100.0 | 0.43 | 0 | 165 | - | METRNL |
| 18 | 8314780 | SNV | C | T | 1 | 25074 | Hetero | 17 | 116 | 14.7 | 0.48 | 0 | 145 | No | PTPRM |
| 18 | 9888143 | SNV | G | A | 1 | 25074 | Hetero | 114 | 180 | 63.3 | 0.43 | 0 | 194 | - | TXNDC2 |
| 18 | 13611175 | SNV | A | G | 1 | 25074 | Hetero | 13 | 70 | 18.6 | 0.33 | 0 | 62 | - | C18orf1 |
| 18 | 14837169 | Deletion | T | - | 1 | 25074 | Hetero | 35 | 48 | 72.9 | 0.20 | 0 | 58 | - | ANKRD30B |
| 18 | 18691280 | SNV | G | A | 1 | 25074 | Hetero | 87 | 112 | 77.7 | 0.38 | 0 | 143 | - | ROCK1 |
| 18 | 24435006 | Deletion | A | - | 1 | 25074 | Hetero | 14 | 93 | 15.1 | 0.44 | 0 | 127 | - | AQP4 |
| 18 | 29480919 | SNV | T | C | 1 | 25074 | Hetero | 58 | 70 | 82.9 | 0.32 | 0 | 53 | Yes | TRAPPC8 |
| 18 | 29709056 | SNV | G | A | 1 | 25074 | Hetero | 26 | 81 | 32.1 | 0.38 | 0 | 102 | - | RNF138 |
| 18 | 43432031 | Deletion | C | - | 1 | 25074 | Hetero | 54 | 70 | 77.1 | 0.43 | 0 | 86 | - | EPG5 |
| 18 | 47373488 | SNV | A | G | 1 | 25074 | Hetero | 6 | 52 | 11.5 | 0.25 | 0 | 58 | - | SCARNA17 |
| 18 | 48322396 | SNV | T | C | 1 | 25074 | Hetero | 55 | 67 | 82.1 | 0.47 | 0 | 66 | - | MRO |
| 18 | 48327814 | SNV | C | T | 1 | 25074 | Hetero | 42 | 52 | 80.8 | 0.40 | 0 | 73 | Yes | MRO |
| 18 | 57020418 | SNV | A | C | 1 | 25074 | Hetero | 19 | 88 | 21.6 | 0.10 | 0 | 80 | - | LMAN1 |
| 18 | 11883448 | Insertion | - | A | 1 | 25074 | Hetero | 11 | 51 | 21.6 | 0.36 | 0 | 69 | - | GNAL |
| 18 | 12014383 | Insertion | - | A | 1 | 25074 | Hetero | 11 | 72 | 15.3 | 0.45 | 0 | 74 | - | IMPA2 |
| 18 | 19020210 | Insertion | - | T | 1 | 25074 | Hetero | 66 | 81 | 81.5 | 0.29 | 0 | 79 | - | GREB1L |
| 18 | 29847462 | Insertion | - | T | 1 | 25074 | Hetero | 43 | 75 | 57.3 | 0.42 | 0 | 64 | - | FAM59A |
| 18 | 45363142 | Insertion | - | G | 1 | 25074 | Hetero | 47 | 55 | 85.5 | 0.42 | 0 | 70 | - | SMAD2 |
| 18 | 56412882 | Insertion | - | T | 1 | 25074 | Hetero | 57 | 66 | 86.4 | 0.07 | 0 | 47 | - | MALT1 |
| 18 | 911377 | Insertion | - | T | 1 | 25074 | Hetero | 92 | 116 | 79.3 | 0.40 | 0 | 108 | - | ADCYAP1 |
| 19 | 828362 | SNV | C | A | 1 | 25074 | Hetero | 87 | 144 | 60.4 | 0.27 | 0 | 80 | Yes | AZU1 |
| 19 | 1467446 | SNV | G | A | 1 | 25074 | Hetero | 40 | 68 | 58.8 | 0.25 | 0 | 46 | No | APC2 |
| 19 | 1821853 | SNV | C | T | 1 | 25074 | Hetero | 47 | 74 | 63.5 | 0.35 | 0 | 38 | - | REXO1 |
| 19 | 2251059 | SNV | C | T | 1 | 25074 | Hetero | 41 | 112 | 36.6 | 0.46 | 0 | 76 | - | AMH |
| 19 | 2643436 | SNV | C | T | 1 | 25074 | Hetero | 61 | 202 | 30.2 | 0.21 | 0 | 154 | - | GNG7 |
| 19 | 2877509 | SNV | A | T | 1 | 25074 | Hetero | 81 | 194 | 41.8 | 0.42 | 0 | 119 | Yes | ZNF556 |
| 19 | 4217074 | SNV | G | A | 1 | 25074 | Hetero | 50 | 129 | 38.8 | 0.36 | 0 | 77 | No | ANKRD24 |
| 19 | 5831000 | Deletion | C | - | 1 | 25074 | Hetero | 211 | 324 | 65.1 | 0.37 | 0 | 214 | - | FUT6 |
| 19 | 6165806 | SNV | G | C | 1 | 25074 | Hetero | 23 | 65 | 35.4 | 0.27 | 0 | 52 | - | RFX2 |
| 19 | 7542312 | SNV | G | A | 1 | 25074 | Hetero | 56 | 95 | 58.9 | 0.25 | 0 | 69 | Yes | PEX11G |
| 19 | 7570876 | SNV | C | A | 1 | 25074 | Hetero | 44 | 126 | 34.9 | 0.45 | 0 | 69 | Yes | C19orf45 |
| 19 | 7712298 | SNV | C | T | 1 | 25074 | Hetero | 19 | 94 | 20.2 | 0.50 | 0 | 77 | Yes | STXBP2 |
| 19 | 7978685 | SNV | A | T | 1 | 25074 | Hetero | 42 | 133 | 31.6 | 0.42 | 0 | 57 | - | MAP2K7 |
| 19 | 8188250 | SNV | G | A | 1 | 25074 | Homo | 64 | 70 | 91.4 | 0.20 | 0 | 57 | - | FBN3 |
| 19 | 8654264 | SNV | C | T | 1 | 25074 | Hetero | 26 | 90 | 28.9 | 0.47 | 0 | 69 | - | ADAMTS10 |
| 19 | 9068022 | SNV | G | A | 1 | 25074 | Hetero | 19 | 115 | 16.5 | 0.33 | 0 | 110 | Yes | MUC16 |
| 19 | 9225801 | SNV | T | C | 1 | 25074 | Hetero | 101 | 198 | 51.0 | 0.47 | 0 | 160 | No | OR7G1 |
| 19 | 9922385 | SNV | A | G | 1 | 25074 | Hetero | 75 | 126 | 59.5 | 0.33 | 0 | 77 | Yes | FBXL12 |
| 19 | 12016861 | SNV | G | A | 1 | 25074 | Hetero | 47 | 139 | 33.8 | 0.43 | 0 | 125 | Yes | ZNF69 |
| 19 | 15133658 | SNV | C | T | 1 | 25074 | Hetero | 65 | 166 | 39.2 | 0.23 | 0 | 116 | - | CCDC105 |
| 19 | 17660284 | SNV | A | G | 1 | 25074 | Hetero | 89 | 173 | 51.4 | 0.38 | 0 | 118 | No | FAM129C |
| 19 | 18186575 | SNV | G | C | 1 | 25074 | Hetero | 72 | 170 | 42.4 | 0.29 | 0 | 107 | No | IL12RB1 |
| 19 | 18898342 | SNV | C | T | 1 | 25074 | Hetero | 26 | 77 | 33.8 | 0.20 | 0 | 61 | Yes | COMP |
| 19 | 19016386 | SNV | G | C | 1 | 25074 | Hetero | 36 | 92 | 39.1 | 0.29 | 0 | 56 | Possible splice site disruption | COPE |
| 19 | 19231634 | SNV | A | G | 1 | 25074 | Hetero | 74 | 121 | 61.2 | 0.28 | 0 | 77 | No | TMEM161A |
| 19 | 19637102 | Deletion | C | - | 1 | 25074 | Hetero | 76 | 235 | 32.3 | 0.31 | 0 | 161 | - | NDUFA13 |
| 19 | 33465006 | SNV | G | T | 1 | 25074 | Hetero | 16 | 99 | 16.2 | 0.32 | 0 | 87 | Yes | C19orf40 |
| 19 | 35085332 | SNV | T | C | 1 | 25074 | Hetero | 75 | 94 | 79.8 | 0.33 | 0 | 80 | - | SCGB2B2 |
| 19 | 35642475 | SNV | C | T | 1 | 25074 | Hetero | 35 | 187 | 18.7 | 0.36 | 0 | 142 | - | FXYD7 |
| 19 | 36018994 | SNV | C | G | 1 | 25074 | Hetero | 203 | 272 | 74.6 | 0.49 | 0 | 229 | Yes | SBSN |
| 19 | 37642863 | SNV | C | T | 1 | 25074 | Hetero | 138 | 197 | 70.1 | 0.42 | 0 | 204 | No | ZNF585A |
| 19 | 38912854 | SNV | C | T | 1 | 25074 | Hetero | 39 | 171 | 22.8 | 0.31 | 0 | 89 | - | RASGRP4 |
| 19 | 40575181 | SNV | C | A | 1 | 25074 | Hetero | 31 | 39 | 79.5 | 0.38 | 0 | 32 | - | ZNF780A |
| 19 | 41945450 | SNV | A | G | 1 | 25074 | Hetero | 71 | 95 | 74.7 | 0.15 | 0 | 90 | - | ATP5SL |
| 19 | 42342295 | SNV | C | T | 1 | 25074 | Hetero | 45 | 77 | 58.4 | 0.35 | 0 | 58 | No | LYPD4 |
| 19 | 45781801 | SNV | A | G | 1 | 25074 | Hetero | 16 | 77 | 20.8 | 0.39 | 0 | 51 | Yes | MARK4 |
| 19 | 46112876 | SNV | G | A | 1 | 25074 | Hetero | 13 | 79 | 16.5 | 0.14 | 0 | 74 | - | EML2 |
| 19 | 48525586 | SNV | G | A | 1 | 25074 | Hetero | 11 | 97 | 11.3 | 0.17 | 0 | 88 | - | ELSPBP1 |
| 19 | 50384515 | SNV | G | A | 1 | 25074 | Hetero | 33 | 103 | 32.0 | 0.43 | 0 | 65 | - | TBC1D17 |
| 19 | 51871135 | SNV | C | T | 1 | 25074 | Hetero | 123 | 165 | 74.5 | 0.28 | 0 | 122 | - | CLDND2 |
| 19 | 54377440 | SNV | C | T | 1 | 25074 | Hetero | 60 | 240 | 25.0 | 0.41 | 0 | 208 | No | MYADM |
| 19 | 54723896 | SNV | T | C | 1 | 25074 | Homo | 52 | 54 | 96.3 | 0.34 | 0 | 163 | - | RPS9 |
| 19 | 54723898 | SNV | T | C | 1 | 25074 | Homo | 48 | 56 | 85.7 | 0.31 | 0 | 168 | - | RPS9 |
| 19 | 55113133 | SNV | G | A | 1 | 25074 | Hetero | 25 | 115 | 21.7 | 0.14 | 0 | 98 | - | LILRB1 |
| 19 | 55239223 | SNV | G | A | 1 | 25074 | Homo | 177 | 177 | 100.0 | 0.40 | 0 | 125 | Yes | KIR3DL1 |
| 19 | 55494076 | SNV | G | A | 1 | 25074 | Hetero | 14 | 91 | 15.4 | 0.29 | 0 | 74 | Yes | NLRP2 |
| 19 | 55593837 | SNV | G | T | 1 | 25074 | Hetero | 92 | 130 | 70.8 | 0.34 | 0 | 75 | Yes | EPS8L1 |
| 19 | 55872240 | SNV | G | A | 1 | 25074 | Hetero | 170 | 230 | 73.9 | 0.45 | 0 | 167 | No | FAM71E2 |
| 19 | 56052374 | SNV | G | A | 1 | 25074 | Hetero | 200 | 252 | 79.4 | 0.31 | 0 | 180 | No | SGK110 |
| 19 | 56938308 | Deletion | A | - | 1 | 25074 | Hetero | 11 | 89 | 12.4 | 0.25 | 0 | 64 | - | ZNF583 |
| 19 | 57742518 | SNV | C | T | 1 | 25074 | Hetero | 39 | 139 | 28.1 | 0.38 | 0 | 111 | - | AURKC |
| 19 | 10097443 | Insertion | - | AA | 2 | 25074 | Hetero | 38 | 116 | 32.8 | 0.47 | 0 | 89 | - | COL5A3 |
| 19 | 12190463 | Insertion | - | A | 1 | 25074 | Hetero | 23 | 65 | 35.4 | 0.44 | 0 | 58 | - | ZNF844 |
| 19 | 14070706 | Insertion | - | G | 1 | 25074 | Hetero | 17 | 167 | 10.2 | 0.24 | 0 | 191 | Possible splice site disruption | DCAF15 |
| 19 | 16756040 | Insertion | - | AG | 2 | 25074 | Hetero | 53 | 76 | 69.7 | 0.34 | 0 | 64 | - | C19orf42 |
| 19 | 17714028 | Insertion | - | AC | 2 | 25074 | Hetero | 41 | 139 | 29.5 | 0.35 | 0 | 123 | - | UNC13A |
| 19 | 34304260 | Insertion | - | T | 1 | 25074 | Hetero | 68 | 88 | 77.3 | 0.41 | 0 | 68 | - | KCTD15 |
| 19 | 41869487 | Insertion | - | A | 1 | 25074 | Hetero | 16 | 84 | 19.0 | 0.11 | 0 | 58 | - | TMEM91 |
| 19 | 46032736 | Deletion | AG | - | 2 | 25074 | Hetero | 41 | 200 | 20.5 | 0.15 | 0 | 114 | - | OPA3 |
| 19 | 46136268 | Insertion | - | T | 1 | 25074 | Hetero | 44 | 200 | 22.0 | 0.44 | 0 | 179 | - | EML2 |
| 19 | 49120252 | Insertion | - | A | 1 | 25074 | Hetero | 24 | 35 | 68.6 | 0.32 | 0 | 41 | - | RPL18 |
| 19 | 56527179 | Insertion | - | C | 1 | 25074 | Hetero | 75 | 103 | 72.8 | 0.41 | 0 | 130 | - | NLRP5 |
| 19 | 9435321 | Deletion | AA | - | 2 | 25074 | Hetero | 6 | 48 | 12.5 | 0.33 | 0 | 40 | - | ZNF559 |
| 20 | 3302900 | SNV | C | T | 1 | 25074 | Hetero | 33 | 81 | 40.7 | 0.39 | 0 | 48 | No | C20orf194 |
| 20 | 10385879 | SNV | C | T | 1 | 25074 | Hetero | 28 | 258 | 10.9 | 0.50 | 0 | 157 | - | MKKS |
| 20 | 11904723 | SNV | C | A | 1 | 25074 | Hetero | 31 | 154 | 20.1 | 0.42 | 0 | 116 | - | BTBD3 |
| 20 | 13839962 | SNV | C | T | 1 | 25074 | Hetero | 60 | 102 | 58.8 | 0.46 | 0 | 71 | No | SEL1L2 |
| 20 | 23066156 | SNV | C | T | 1 | 25074 | Hetero | 48 | 300 | 16.0 | 0.37 | 0 | 178 | Yes | CD93 |
| 20 | 25197273 | SNV | C | T | 1 | 25074 | Hetero | 80 | 163 | 49.1 | 0.43 | 0 | 82 | - | ENTPD6 |
| 20 | 25202063 | SNV | G | A | 1 | 25074 | Hetero | 72 | 150 | 48.0 | 0.38 | 0 | 91 | - | ENTPD6 |
| 20 | 26188954 | Deletion | T | - | 1 | 25074 | Hetero | 101 | 770 | 13.1 | 0.42 | 0 | 1281 | - | RP3-410C9.1 |
| 20 | 30309566 | SNV | C | T | 1 | 25074 | Hetero | 225 | 455 | 49.5 | 0.36 | 0 | 250 | No | BCL2L1 |
| 20 | 30584762 | SNV | G | T | 1 | 25074 | Hetero | 225 | 485 | 46.4 | 0.39 | 0 | 270 | No | XKR7 |
| 20 | 31805182 | SNV | C | T | 1 | 25074 | Hetero | 88 | 200 | 44.0 | 0.18 | 0 | 113 | - | BPIFA3 |
| 20 | 32000618 | SNV | C | T | 1 | 25074 | Hetero | 53 | 126 | 42.1 | 0.17 | 0 | 79 | - | SNTA1 |
| 20 | 33863756 | SNV | C | T | 1 | 25074 | Hetero | 43 | 71 | 60.6 | 0.24 | 0 | 49 | - | EDEM2 |
| 20 | 35749005 | SNV | T | G | 1 | 25074 | Hetero | 34 | 80 | 42.5 | 0.29 | 0 | 62 | Yes | C20orf132 |
| 20 | 35787298 | SNV | T | G | 1 | 25074 | Hetero | 67 | 129 | 51.9 | 0.19 | 0 | 71 | No | C20orf132 |
| 20 | 36488414 | SNV | G | T | 1 | 25074 | Hetero | 105 | 204 | 51.5 | 0.31 | 0 | 121 | Yes | CTNNBL1 |
| 20 | 39317566 | SNV | G | T | 1 | 25074 | Hetero | 47 | 84 | 56.0 | 0.13 | 0 | 86 | - | MAFB |
| 20 | 40944592 | SNV | C | T | 1 | 25074 | Hetero | 44 | 94 | 46.8 | 0.29 | 0 | 49 | Yes | PTPRT |
| 20 | 43030125 | SNV | A | C | 1 | 25074 | Hetero | 85 | 193 | 44.0 | 0.41 | 0 | 156 | Yes | HNF4A |
| 20 | 44802447 | SNV | A | G | 1 | 25074 | Hetero | 49 | 112 | 43.8 | 0.38 | 0 | 77 | - | CDH22 |
| 20 | 48894902 | SNV | T | G | 1 | 25074 | Hetero | 105 | 196 | 53.6 | 0.20 | 0 | 89 | - | RP11-290F20.3 |
| 20 | 18541699 | Insertion | - | A | 1 | 25074 | Hetero | 76 | 156 | 48.7 | 0.46 | 0 | 102 | - | SEC23B |
| 20 | 26188967 | Insertion | - | C | 1 | 25074 | Hetero | 81 | 778 | 10.4 | 0.46 | 0 | 1389 | - | RP3-410C9.1 |
| 20 | 30060720 | Insertion | - | CA | 2 | 25074 | Hetero | 18 | 71 | 25.4 | 0.35 | 0 | 38 | - | DEFB124 |
| 20 | 33057941 | Insertion | - | T | 1 | 25074 | Hetero | 82 | 221 | 37.1 | 0.25 | 0 | 126 | - | ITCH |
| 20 | 43128540 | Insertion | - | T | 1 | 25074 | Hetero | 27 | 85 | 31.8 | 0.43 | 0 | 49 | - | SERINC3 |
| 20 | 44453335 | Insertion | - | C | 1 | 25074 | Hetero | 25 | 89 | 28.1 | 0.38 | 0 | 58 | - | TNNC2 |
| 20 | 44468982 | Insertion | - | C | 1 | 25074 | Homo | 113 | 113 | 100.0 | 0.23 | 0 | 56 | - | SNX21 |
| 20 | 44512793 | Deletion | TG | - | 2 | 25074 | Hetero | 69 | 131 | 52.7 | 0.37 | 0 | 52 | - | ZSWIM1 |
| 20 | 50003737 | Insertion | - | A | 1 | 25074 | Hetero | 78 | 132 | 59.1 | 0.43 | 0 | 90 | - | NFATC2 |
| 20 | 50008006 | Insertion | - | G | 1 | 25074 | Hetero | 8 | 57 | 14.0 | 0.50 | 0 | 34 | - | NFATC2 |
| 20 | 5178081 | Insertion | - | CT | 2 | 25074 | Hetero | 60 | 104 | 57.7 | 0.40 | 0 | 57 | - | CDS2 |
| 20 | 5754003 | Insertion | - | T | 1 | 25074 | Hetero | 79 | 166 | 47.6 | 0.35 | 0 | 98 | - | C20orf196 |
| 20 | 58512721 | Insertion | - | T | 1 | 25074 | Hetero | 56 | 96 | 58.3 | 0.44 | 0 | 54 | - | FAM217B |
| 21 | 9908366 | SNV | C | T | 1 | 25074 | Hetero | 81 | 331 | 24.5 | 0.47 | 0 | 455 | - | MAFIPL |
| 21 | 9909099 | SNV | C | T | 1 | 25074 | Hetero | 25 | 115 | 21.7 | 0.21 | 0 | 127 | - | MAFIPL |
| 21 | 30717617 | Deletion | T | - | 1 | 25074 | Homo | 87 | 87 | 100.0 | 0.46 | 0 | 68 | - | BACH1 |
| 21 | 32201816 | SNV | G | A | 1 | 25074 | Hetero | 51 | 109 | 46.8 | 0.24 | 0 | 148 | - | ENSG00000184586 |
| 21 | 33296822 | SNV | A | T | 1 | 25074 | Hetero | 59 | 108 | 54.6 | 0.30 | 0 | 95 | Yes | HUNK |
| 21 | 33721550 | SNV | A | C | 1 | 25074 | Hetero | 74 | 164 | 45.1 | 0.25 | 0 | 152 | - | URB1 |
| 21 | 33739041 | SNV | A | G | 1 | 25074 | Hetero | 50 | 101 | 49.5 | 0.44 | 0 | 77 | No | URB1 |
| 21 | 36090072 | SNV | T | C | 1 | 25074 | Hetero | 52 | 102 | 51.0 | 0.45 | 0 | 77 | - | CLIC6 |
| 21 | 36162828 | SNV | A | G | 1 | 25074 | Hetero | 45 | 80 | 56.3 | 0.50 | 0 | 73 | - | RUNX1 |
| 21 | 38390879 | SNV | T | C | 1 | 25074 | Hetero | 19 | 58 | 32.8 | 0.40 | 0 | 66 | - | DSCR6 |
| 21 | 43767589 | SNV | C | T | 1 | 25074 | Hetero | 94 | 196 | 48.0 | 0.44 | 0 | 172 | - | TFF2 |
| 21 | 43838679 | SNV | C | T | 1 | 25074 | Hetero | 53 | 93 | 57.0 | 0.24 | 0 | 90 | Yes | UBASH3A |
| 21 | 44592546 | SNV | T | G | 1 | 25074 | Hetero | 106 | 198 | 53.5 | 0.42 | 0 | 168 | - | CRYAA |
| 21 | 46309265 | SNV | G | A | 1 | 25074 | Hetero | 44 | 106 | 41.5 | 0.47 | 0 | 96 | No | ITGB2 |
| 21 | 46888627 | SNV | G | A | 1 | 25074 | Hetero | 21 | 62 | 33.9 | 0.13 | 0 | 55 | Yes | COL18A1 |
| 21 | 46915528 | SNV | G | A | 1 | 25074 | Hetero | 31 | 67 | 46.3 | 0.44 | 0 | 83 | - | COL18A1 |
| 21 | 46915877 | SNV | T | G | 1 | 25074 | Hetero | 21 | 47 | 44.7 | 0.30 | 0 | 57 | - | COL18A1 |
| 21 | 46915985 | SNV | C | T | 1 | 25074 | Hetero | 8 | 75 | 10.7 | 0.50 | 0 | 87 | - | COL18A1 |
| 21 | 47333960 | SNV | G | A | 1 | 25074 | Hetero | 91 | 168 | 54.2 | 0.41 | 0 | 133 | No | PCBP3 |
| 21 | 47572798 | Deletion | G | - | 1 | 25074 | Hetero | 32 | 74 | 43.2 | 0.35 | 0 | 78 | - | FTCD |
| 21 | 47648552 | SNV | C | T | 1 | 25074 | Hetero | 81 | 175 | 46.3 | 0.38 | 0 | 142 | - | LSS |
| 21 | 47731439 | SNV | G | C | 1 | 25074 | Hetero | 30 | 52 | 57.7 | 0.12 | 0 | 48 | Yes | C21orf58 |
| 21 | 47838253 | SNV | A | C | 1 | 25074 | Hetero | 70 | 123 | 56.9 | 0.48 | 0 | 109 | - | PCNT |
| 21 | 46293698 | Insertion | - | C | 1 | 25074 | Hetero | 17 | 40 | 42.5 | 0.11 | 0 | 43 | - | PTTG1IP |
| 22 | 17581245 | SNV | T | G | 1 | 25074 | Hetero | 32 | 86 | 37.2 | 0.46 | 0 | 59 | Possible splice site disruption | IL17RA |
| 22 | 17593891 | Deletion | T | - | 1 | 25074 | Homo | 60 | 64 | 93.8 | 0.43 | 0 | 38 | - | IL17RA |
| 22 | 17599674 | SNV | A | G | 1 | 25074 | Hetero | 33 | 109 | 30.3 | 0.35 | 0 | 82 | - | CECR6 |
| 22 | 21192957 | SNV | T | C | 1 | 25074 | Hetero | 273 | 427 | 63.9 | 0.32 | 0 | 322 | No | PI4KA |
| 22 | 24445648 | SNV | C | T | 1 | 25074 | Hetero | 25 | 64 | 39.1 | 0.21 | 0 | 85 | Yes | CABIN1 |
| 22 | 25424548 | SNV | C | T | 1 | 25074 | Hetero | 33 | 102 | 32.4 | 0.43 | 0 | 80 | No | KIAA1671 |
| 22 | 25428562 | SNV | G | A | 1 | 25074 | Hetero | 28 | 48 | 58.3 | 0.24 | 0 | 50 | - | KIAA1671 |
| 22 | 32330385 | SNV | C | T | 1 | 25074 | Hetero | 55 | 87 | 63.2 | 0.42 | 0 | 64 | Yes | C22orf24 |
| 22 | 35808729 | SNV | G | A | 1 | 25074 | Hetero | 33 | 98 | 33.7 | 0.17 | 0 | 63 | - | MCM5 |
| 22 | 36545272 | Deletion | A | - | 1 | 25074 | Hetero | 39 | 98 | 39.8 | 0.37 | 0 | 66 | - | APOL3 |
| 22 | 37452285 | SNV | G | A | 1 | 25074 | Hetero | 51 | 92 | 55.4 | 0.23 | 0 | 54 | - | KCTD17 |
| 22 | 37765878 | SNV | G | C | 1 | 25074 | Hetero | 52 | 189 | 27.5 | 0.25 | 0 | 129 | - | ELFN2 |
| 22 | 37891661 | SNV | G | A | 1 | 25074 | Homo | 115 | 117 | 98.3 | 0.46 | 0 | 88 | - | CARD10 |
| 22 | 38165136 | SNV | G | A | 1 | 25074 | Hetero | 38 | 79 | 48.1 | 0.40 | 0 | 46 | Yes | TRIOBP |
| 22 | 39421774 | Deletion | C | - | 1 | 25074 | Hetero | 42 | 71 | 59.2 | 0.18 | 0 | 44 | - | APOBEC3D |
| 22 | 42522651 | SNV | G | C | 1 | 25074 | Hetero | 39 | 88 | 44.3 | 0.33 | 0 | 61 | No | C22orf32 |
| 22 | 42539452 | SNV | G | A | 1 | 25074 | Hetero | 30 | 67 | 44.8 | 0.34 | 0 | 43 | No | CYP2D7P1 |
| 22 | 42546706 | SNV | G | T | 1 | 25074 | Hetero | 27 | 39 | 69.2 | 0.10 | 0 | 32 | - | CYP2D8P1 |
| 22 | 43616574 | SNV | G | A | 1 | 25074 | Hetero | 24 | 178 | 13.5 | 0.21 | 0 | 111 | No | SCUBE1 |
| 22 | 50721691 | SNV | C | T | 1 | 25074 | Hetero | 113 | 188 | 60.1 | 0.21 | 0 | 118 | - | PLXNB2 |
| 22 | 51064480 | SNV | C | T | 1 | 25074 | Hetero | 69 | 125 | 55.2 | 0.39 | 0 | 107 | Yes | ARSA |
| 22 | 24837909 | Insertion | - | T | 1 | 25074 | Homo | 139 | 140 | 99.3 | 0.35 | 0 | 151 | - | ADORA2A |
| 22 | 29130347 | Insertion | - | T | 1 | 25074 | Hetero | 34 | 58 | 58.6 | 0.16 | 0 | 40 | - | CHEK2 |
| 22 | 29196043 | Insertion | - | T | 1 | 25074 | Hetero | 39 | 125 | 31.2 | 0.40 | 0 | 93 | - | XBP1 |
| 22 | 35948603 | Insertion | - | AA | 2 | 25074 | Hetero | 70 | 123 | 56.9 | 0.48 | 0 | 90 | - | RASD2 |
| 22 | 36677553 | Insertion | - | A | 1 | 25074 | Hetero | 31 | 77 | 40.3 | 0.42 | 0 | 60 | - | MYH9 |
| 22 | 39482241 | Insertion | - | A | 1 | 25074 | Hetero | 18 | 69 | 26.1 | 0.19 | 0 | 46 | - | APOBEC3G |
| 22 | 39916626 | Insertion | - | C | 1 | 25074 | Hetero | 51 | 83 | 61.4 | 0.45 | 0 | 60 | - | ATF4 |
| 22 | 41175909 | Insertion | - | A | 1 | 25074 | Homo | 64 | 65 | 98.5 | 0.39 | 0 | 65 | - | SLC25A17 |
| 22 | 44891541 | Insertion | - | C | 1 | 25074 | Homo | 73 | 87 | 83.9 | 0.45 | 0 | 59 | - | LDOC1L |
| 22 | 50564618 | Insertion | - | T | 1 | 25074 | Hetero | 64 | 103 | 62.1 | 0.19 | 0 | 85 | - | MOV10L1 |
| 22 | 50646859 | Insertion | - | A | 1 | 25074 | Hetero | 5 | 50 | 10.0 | 0.20 | 0 | 34 | - | RP3-402G11.5 |
| X | 219632 | SNV | C | A | 1 | 25074 | Hetero | 134 | 313 | 42.8 | 0.38 | 0 | 298 | - | PLCXD1 |
| X | 611009 | SNV | C | A | 1 | 25074 | Hetero | 96 | 164 | 58.5 | 0.43 | 0 | 179 | - | SHOX |
| X | 1522164 | Deletion | A | - | 1 | 25074 | Hetero | 35 | 68 | 51.5 | 0.46 | 0 | 70 | Deletion | ASMTL |
| X | 2404842 | SNV | G | A | 1 | 25074 | Hetero | 217 | 386 | 56.2 | 0.39 | 0 | 270 | - | DHRSX |
| X | 9905294 | SNV | T | C | 1 | 25074 | Hetero | 108 | 257 | 42.0 | 0.38 | 0 | 93 | No | SHROOM2 |
| X | 11140697 | Deletion | C | - | 1 | 25074 | Hetero | 69 | 132 | 52.3 | 0.39 | 0 | 43 | - | HCCS |
| X | 12928539 | SNV | G | T | 1 | 25074 | Hetero | 126 | 293 | 43.0 | 0.18 | 0 | 107 | Yes | TLR8 |
| X | 15833863 | SNV | G | A | 1 | 25074 | Hetero | 38 | 110 | 34.5 | 0.39 | 0 | 64 | No | ZRSR2 |
| X | 17818261 | SNV | T | C | 1 | 25074 | Hetero | 57 | 96 | 59.4 | 0.49 | 0 | 30 | - | RAI2 |
| X | 19553811 | Deletion | T | - | 1 | 25074 | Hetero | 13 | 107 | 12.1 | 0.31 | 0 | 32 | - | SH3KBP1 |
| X | 30578299 | SNV | G | T | 1 | 25074 | Hetero | 79 | 257 | 30.7 | 0.46 | 0 | 123 | Yes | CXorf21 |
| X | 42637105 | SNV | T | C | 1 | 25074 | Hetero | 21 | 105 | 20.0 | 0.43 | 0 | 86 | - | ENSG00000102055 |
| X | 45011028 | SNV | C | G | 1 | 25074 | Hetero | 94 | 114 | 82.5 | 0.35 | 0 | 76 | Yes | CXorf36 |
| X | 53566067 | Deletion | A | - | 1 | 25074 | Hetero | 85 | 110 | 77.3 | 0.38 | 0 | 56 | - | HUWE1 |
| X | 55172516 | SNV | A | C | 1 | 25074 | Hetero | 100 | 831 | 12.0 | 0.31 | 0 | 1072 | - | FAM104B |
| X | 71694534 | SNV | C | T | 1 | 25074 | Hetero | 49 | 313 | 15.7 | 0.36 | 0 | 165 | No | HDAC8 |
| X | 77084620 | SNV | A | C | 1 | 25074 | Hetero | 103 | 119 | 86.6 | 0.12 | 0 | 57 | - | MAGT1 |
| X | 77383553 | SNV | C | T | 1 | 25074 | Hetero | 16 | 63 | 25.4 | 0.29 | 0 | 30 | - | PGK1 |
| X | 95940238 | SNV | G | C | 1 | 25074 | Hetero | 11 | 78 | 14.1 | 0.36 | 0 | 45 | - | DIAPH2 |
| X | 111155903 | SNV | G | A | 1 | 25074 | Hetero | 109 | 137 | 79.6 | 0.45 | 0 | 66 | No | TRPC5 |
| X | 118759375 | SNV | T | C | 1 | 25074 | Hetero | 179 | 230 | 77.8 | 0.34 | 0 | 88 | - | SEPT6 |
| X | 123518365 | SNV | C | T | 1 | 25074 | Hetero | 79 | 103 | 76.7 | 0.50 | 0 | 52 | Yes | STAG2 |
| X | 140994260 | SNV | C | G | 1 | 25074 | Hetero | 271 | 341 | 79.5 | 0.50 | 0 | 182 | Yes | MAGEC1 |
| X | 152751427 | SNV | T | G | 1 | 25074 | Hetero | 77 | 104 | 74.0 | 0.20 | 0 | 44 | Yes | HAUS7 |
| X | 152835361 | SNV | C | T | 1 | 25074 | Hetero | 18 | 88 | 20.5 | 0.23 | 0 | 38 | - | ATP2B3 |
| X | 153050623 | SNV | G | A | 1 | 25074 | Hetero | 70 | 94 | 74.5 | 0.15 | 0 | 63 | Yes | SRPK3 |
| X | 153588248 | SNV | C | G | 1 | 25074 | Hetero | 171 | 212 | 80.7 | 0.43 | 0 | 56 | Yes | FLNA |
| X | 155003466 | SNV | G | C | 1 | 25074 | Hetero | 144 | 188 | 76.6 | 0.45 | 0 | 197 | - | SPRY3 |
| X | 100516325 | Insertion | - | TT | 2 | 25074 | Homo | 72 | 75 | 96.0 | 0.49 | 0 | 38 | - | DRP2 |
| X | 133560121 | Insertion | - | T | 1 | 25074 | Hetero | 54 | 66 | 81.8 | 0.45 | 0 | 48 | - | PHF6 |
| X | 135128103 | Insertion | - | T | 1 | 25074 | Hetero | 8 | 39 | 20.5 | 0.13 | 0 | 30 | - | SLC9A6 |
| X | 14622416 | Insertion | - | T | 1 | 25074 | Hetero | 47 | 68 | 69.1 | 0.19 | 0 | 42 | - | GLRA2 |
| X | 15337849 | Insertion | - | A | 1 | 25074 | Hetero | 56 | 126 | 44.4 | 0.30 | 0 | 50 | - | PIGA |
| X | 31200832 | Insertion | - | T | 1 | 25074 | Hetero | 32 | 74 | 43.2 | 0.16 | 0 | 44 | - | DMD |
| X | 9659767 | Insertion | - | G | 1 | 25074 | Hetero | 59 | 162 | 36.4 | 0.32 | 0 | 52 | - | TBL1X |
| Y | 21154426 | SNV | G | A | 1 | 25074 | Hetero | 16 | 39 | 41.0 | 0.42 | 0 | 62 | - | TTTY14 |
| 1 | 15755487 | SNV | C | G | 1 | 25089 | Hetero | 70 | 248 | 28.2 | 0.35 | 0 | 142 | - | EFHD2 |
| 1 | 28905170 | SNV | T | C | 1 | 25089 | Hetero | 67 | 225 | 29.8 | 0.46 | 0 | 116 | - | SNHG12 |
| 1 | 31404688 | SNV | T | C | 1 | 25089 | Hetero | 48 | 110 | 43.6 | 0.45 | 0 | 71 | - | PUM1 |
| 1 | 53542923 | SNV | G | A | 1 | 25089 | Hetero | 123 | 398 | 30.9 | 0.42 | 0 | 203 | Yes | PODN |
| 1 | 87599370 | SNV | G | A | 1 | 25089 | Hetero | 40 | 126 | 31.7 | 0.49 | 0 | 80 | No | HS2ST1 |
| 1 | 89851751 | SNV | A | T | 1 | 25089 | Hetero | 30 | 99 | 30.3 | 0.47 | 0 | 36 | - | GBP6 |
| 1 | 92181868 | SNV | T | C | 1 | 25089 | Hetero | 29 | 102 | 28.4 | 0.47 | 0 | 42 | No | TGFBR3 |
| 1 | 104297272 | SNV | C | T | 1 | 25089 | Hetero | 31 | 250 | 12.4 | 0.30 | 0 | 106 | - | AMY1C |
| 1 | 112313431 | Deletion | T | - | 1 | 25089 | Hetero | 5 | 44 | 11.4 | 0.40 | 0 | 33 | - | KCND3 |
| 1 | 153661456 | Deletion | C | - | 1 | 25089 | Hetero | 70 | 237 | 29.5 | 0.37 | 0 | 118 | Deletion | NPR1 |
| 1 | 222840503 | SNV | G | T | 1 | 25089 | Hetero | 64 | 130 | 49.2 | 0.44 | 0 | 38 | - | MIA3 |
| 1 | 226254652 | SNV | A | G | 1 | 25089 | Hetero | 24 | 76 | 31.6 | 0.43 | 0 | 30 | - | H3F3A |
| 1 | 231353571 | SNV | G | C | 1 | 25089 | Hetero | 67 | 304 | 22.0 | 0.49 | 0 | 145 | - | TRIM67 |
| 1 | 244218294 | SNV | C | T | 1 | 25089 | Hetero | 211 | 470 | 44.9 | 0.45 | 0 | 213 | No | ZNF238 |
| 1 | 20519954 | Insertion | - | T | 1 | 25089 | Hetero | 47 | 172 | 27.3 | 0.38 | 0 | 54 | - | UBXN10 |
| 1 | 32831245 | Insertion | - | A | 1 | 25089 | Hetero | 79 | 224 | 35.3 | 0.45 | 0 | 94 | - | BSDC1 |
| 2 | 1677450 | SNV | G | A | 1 | 25089 | Hetero | 91 | 262 | 34.7 | 0.25 | 0 | 139 | Yes | PXDN |
| 2 | 14777662 | SNV | A | T | 1 | 25089 | Hetero | 28 | 85 | 32.9 | 0.43 | 0 | 41 | - | FAM84A |
| 2 | 24991942 | SNV | A | G | 1 | 25089 | Hetero | 72 | 211 | 34.1 | 0.43 | 0 | 113 | - | NCOA1 |
| 2 | 27167670 | SNV | C | T | 1 | 25089 | Hetero | 67 | 191 | 35.1 | 0.36 | 0 | 97 | No | DPYSL5 |
| 2 | 64686498 | SNV | C | T | 1 | 25089 | Hetero | 21 | 90 | 23.3 | 0.36 | 0 | 38 | - | LGALSL |
| 2 | 97399353 | SNV | C | A | 1 | 25089 | Hetero | 38 | 150 | 25.3 | 0.18 | 0 | 71 | - | LMAN2L |
| 2 | 231738234 | SNV | G | A | 1 | 25089 | Hetero | 79 | 258 | 30.6 | 0.46 | 0 | 154 | Yes | ITM2C |
| 2 | 233438981 | SNV | A | G | 1 | 25089 | Hetero | 98 | 323 | 30.3 | 0.36 | 0 | 165 | Yes | EIF4E2 |
| 2 | 202029001 | Insertion | - | A | 1 | 25089 | Hetero | 10 | 97 | 10.3 | 0.29 | 0 | 40 | - | CFLAR |
| 3 | 48885266 | SNV | C | A | 1 | 25089 | Hetero | 26 | 45 | 57.8 | 0.28 | 0 | 32 | - | PRKAR2A |
| 3 | 100508282 | SNV | C | T | 1 | 25089 | Hetero | 24 | 130 | 18.5 | 0.30 | 0 | 42 | - | ABI3BP |
| 3 | 129100227 | SNV | T | C | 1 | 25089 | Hetero | 81 | 327 | 24.8 | 0.32 | 0 | 98 | - | RP11-529F4.1 |
| 3 | 172241220 | SNV | C | G | 1 | 25089 | Hetero | 191 | 421 | 45.4 | 0.48 | 0 | 171 | - | TNFSF10 |
| 3 | 196736477 | Insertion | - | G | 1 | 25089 | Hetero | 38 | 155 | 24.5 | 0.33 | 0 | 51 | - | MFI2 |
| 4 | 37357999 | SNV | G | T | 1 | 25089 | Hetero | 24 | 87 | 27.6 | 0.28 | 0 | 49 | No | KIAA1239 |
| 4 | 76895255 | SNV | T | C | 1 | 25089 | Hetero | 38 | 154 | 24.7 | 0.42 | 0 | 88 | Yes | SDAD1 |
| 4 | 183659696 | SNV | C | T | 1 | 25089 | Hetero | 30 | 98 | 30.6 | 0.27 | 0 | 54 | No | ODZ3 |
| 4 | 105389550 | Insertion | - | T | 1 | 25089 | Hetero | 9 | 82 | 11.0 | 0.33 | 0 | 52 | - | CXXC4 |
| 5 | 1880158 | SNV | G | C | 1 | 25089 | Hetero | 17 | 51 | 33.3 | 0.33 | 0 | 30 | - | IRX4 |
| 5 | 41203371 | SNV | T | A | 1 | 25089 | Hetero | 139 | 355 | 39.2 | 0.35 | 0 | 159 | - | C6 |
| 5 | 43652013 | SNV | A | G | 1 | 25089 | Hetero | 22 | 95 | 23.2 | 0.20 | 0 | 42 | - | NNT |
| 5 | 54718718 | SNV | G | T | 1 | 25089 | Hetero | 86 | 230 | 37.4 | 0.21 | 0 | 101 | Yes | SKIV2L2 |
| 5 | 140227416 | SNV | C | T | 1 | 25089 | Hetero | 42 | 90 | 46.7 | 0.40 | 0 | 50 | - | PCDHA1 |
| 5 | 140261954 | SNV | C | T | 1 | 25089 | Hetero | 50 | 111 | 45.0 | 0.14 | 0 | 77 | Yes | PCDHA1 |
| 5 | 72185591 | Insertion | - | T | 1 | 25089 | Hetero | 6 | 59 | 10.2 | 0.14 | 0 | 35 | - | TNPO1 |
| 5 | 77296411 | Insertion | - | A | 1 | 25089 | Hetero | 20 | 54 | 37.0 | 0.41 | 0 | 41 | - | AP3B1 |
| 6 | 24651196 | SNV | A | T | 1 | 25089 | Hetero | 72 | 159 | 45.3 | 0.48 | 0 | 67 | No | TDP2 |
| 6 | 39053982 | SNV | T | C | 1 | 25089 | Hetero | 16 | 116 | 13.8 | 0.29 | 0 | 60 | - | GLP1R |
| 6 | 64415945 | SNV | C | G | 1 | 25089 | Hetero | 44 | 164 | 26.8 | 0.44 | 0 | 71 | Yes | PHF3 |
| 6 | 114182252 | Deletion | TT | - | 2 | 25089 | Hetero | 9 | 85 | 10.6 | 0.11 | 0 | 38 | - | MARCKS |
| 6 | 158613375 | Deletion | AA | - | 2 | 25089 | Hetero | 12 | 104 | 11.5 | 0.25 | 0 | 33 | - | GTF2H5 |
| 7 | 2294696 | SNV | G | A | 1 | 25089 | Hetero | 60 | 170 | 35.3 | 0.48 | 0 | 92 | Yes | SNX8 |
| 7 | 11441514 | SNV | C | T | 1 | 25089 | Hetero | 45 | 152 | 29.6 | 0.22 | 0 | 84 | Yes | THSD7A |
| 7 | 12272256 | SNV | A | G | 1 | 25089 | Hetero | 38 | 110 | 34.5 | 0.48 | 0 | 45 | - | TMEM106B |
| 7 | 21784069 | SNV | C | T | 1 | 25089 | Hetero | 48 | 134 | 35.8 | 0.42 | 0 | 75 | Yes | DNAH11 |
| 7 | 33407331 | SNV | C | G | 1 | 25089 | Hetero | 17 | 63 | 27.0 | 0.20 | 0 | 33 | - | BBS9 |
| 7 | 47344459 | SNV | G | C | 1 | 25089 | Hetero | 54 | 168 | 32.1 | 0.42 | 0 | 85 | No | TNS3 |
| 7 | 47408542 | SNV | C | T | 1 | 25089 | Hetero | 88 | 287 | 30.7 | 0.40 | 0 | 150 | No | TNS3 |
| 7 | 73097322 | SNV | G | A | 1 | 25089 | Hetero | 69 | 190 | 36.3 | 0.47 | 0 | 99 | No | DNAJC30 |
| 7 | 99987714 | SNV | T | C | 1 | 25089 | Hetero | 32 | 83 | 38.6 | 0.24 | 0 | 41 | Yes | PILRA |
| 7 | 137561885 | SNV | C | A | 1 | 25089 | Hetero | 22 | 65 | 33.8 | 0.39 | 0 | 43 | - | CREB3L2 |
| 7 | 138394473 | SNV | G | A | 1 | 25089 | Hetero | 32 | 102 | 31.4 | 0.39 | 0 | 68 | No | ATP6V0A4 |
| 7 | 131812769 | Deletion | TC | - | 2 | 25089 | Hetero | 10 | 99 | 10.1 | 0.10 | 0 | 42 | - | PLXNA4 |
| 7 | 57531120 | Insertion | - | A | 1 | 25089 | Hetero | 9 | 79 | 11.4 | 0.46 | 0 | 46 | - | ZNF716 |
| 8 | 69703995 | SNV | G | A | 1 | 25089 | Hetero | 65 | 140 | 46.4 | 0.44 | 0 | 49 | - | C8orf34 |
| 8 | 95511725 | SNV | C | T | 1 | 25089 | Hetero | 12 | 89 | 13.5 | 0.31 | 0 | 33 | No | KIAA1429 |
| 8 | 113395780 | SNV | C | G | 1 | 25089 | Hetero | 37 | 149 | 24.8 | 0.22 | 0 | 64 | - | CSMD3 |
| 8 | 139890022 | SNV | C | T | 1 | 25089 | Hetero | 62 | 190 | 32.6 | 0.42 | 0 | 74 | Yes | COL22A1 |
| 8 | 145739559 | SNV | C | G | 1 | 25089 | Hetero | 75 | 295 | 25.4 | 0.44 | 0 | 94 | - | RECQL4 |
| 9 | 21970900 | SNV | C | T | 1 | 25089 | Hetero | 31 | 68 | 45.6 | 0.46 | 0 | 50 | Possible splice site disruption | CDKN2A |
| 9 | 139401755 | SNV | A | T | 1 | 25089 | Hetero | 56 | 117 | 47.9 | 0.19 | 0 | 78 | - | NOTCH1 |
| 9 | 33056972 | Deletion | AA | - | 2 | 25089 | Hetero | 4 | 37 | 10.8 | 0.40 | 0 | 33 | - | SMU1 |
| 9 | 3824933 | Insertion | - | T | 1 | 25089 | Hetero | 9 | 61 | 14.8 | 0.30 | 0 | 46 | - | GLIS3 |
| 10 | 38406627 | SNV | G | T | 1 | 25089 | Hetero | 25 | 81 | 30.9 | 0.43 | 0 | 33 | Yes | ZNF37A |
| 10 | 50953946 | SNV | A | C | 1 | 25089 | Hetero | 50 | 154 | 32.5 | 0.45 | 0 | 59 | No | OGDHL |
| 10 | 102123874 | Insertion | - | T | 1 | 25089 | Hetero | 12 | 64 | 18.8 | 0.46 | 0 | 30 | - | SCD |
| 11 | 1862255 | SNV | C | G | 1 | 25089 | Hetero | 73 | 212 | 34.4 | 0.44 | 0 | 116 | - | TNNI2 |
| 11 | 2434775 | SNV | G | A | 1 | 25089 | Hetero | 67 | 224 | 29.9 | 0.49 | 0 | 102 | Yes | TRPM5 |
| 11 | 18195312 | SNV | C | A | 1 | 25089 | Hetero | 48 | 173 | 27.7 | 0.44 | 0 | 106 | Yes | RP11-113D6.6 |
| 11 | 62591931 | SNV | C | A | 1 | 25089 | Hetero | 50 | 140 | 35.7 | 0.37 | 0 | 87 | Possible splice site disruption | STX5 |
| 11 | 66260292 | SNV | A | G | 1 | 25089 | Hetero | 85 | 286 | 29.7 | 0.39 | 0 | 151 | Yes | DPP3 |
| 11 | 68305169 | Deletion | A | - | 1 | 25089 | Hetero | 1038 | 1172 | 88.6 | 0.47 | 0 | 115 | Deletion | PPP6R3 |
| 11 | 71713148 | SNV | C | T | 1 | 25089 | Hetero | 123 | 231 | 53.2 | 0.36 | 0 | 157 | - | IL18BP |
| 11 | 111600450 | SNV | T | G | 1 | 25089 | Hetero | 38 | 86 | 44.2 | 0.39 | 0 | 45 | - | SIK2 |
| 11 | 117989698 | SNV | A | T | 1 | 25089 | Hetero | 40 | 76 | 52.6 | 0.50 | 0 | 57 | - | TMPRSS4 |
| 12 | 48436769 | Deletion | T | - | 1 | 25089 | Hetero | 13 | 101 | 12.9 | 0.40 | 0 | 31 | - | SENP1 |
| 12 | 49434325 | SNV | G | A | 1 | 25089 | Hetero | 43 | 126 | 34.1 | 0.47 | 0 | 50 | Yes | MLL2 |
| 12 | 51461698 | SNV | C | T | 1 | 25089 | Hetero | 62 | 143 | 43.4 | 0.47 | 0 | 81 | Yes | CSRNP2 |
| 12 | 122691665 | SNV | G | A | 1 | 25089 | Hetero | 95 | 256 | 37.1 | 0.42 | 0 | 121 | No | B3GNT4 |
| 12 | 133334571 | Deletion | A | - | 1 | 25089 | Hetero | 5 | 49 | 10.2 | 0.20 | 0 | 31 | - | POLE |
| 12 | 49430920 | Insertion | - | G | 1 | 25089 | Hetero | 54 | 143 | 37.8 | 0.22 | 0 | 75 | Insertion | MLL2 |
| 13 | 21353832 | SNV | C | T | 1 | 25089 | Hetero | 34 | 145 | 23.4 | 0.33 | 0 | 54 | - | XPO4 |
| 13 | 53602981 | SNV | G | A | 1 | 25089 | Hetero | 103 | 273 | 37.7 | 0.44 | 0 | 150 | Yes | OLFM4 |
| 13 | 73633169 | SNV | C | T | 1 | 25089 | Hetero | 39 | 142 | 27.5 | 0.28 | 0 | 90 | - | KLF5 |
| 13 | 95278226 | SNV | A | G | 1 | 25089 | Hetero | 25 | 86 | 29.1 | 0.48 | 0 | 58 | Yes | GPR180 |
| 14 | 29237349 | SNV | C | T | 1 | 25089 | Hetero | 55 | 163 | 33.7 | 0.37 | 0 | 66 | No | FOXG1 |
| 14 | 31642636 | SNV | G | A | 1 | 25089 | Hetero | 70 | 254 | 27.6 | 0.25 | 0 | 97 | - | HECTD1 |
| 14 | 35487983 | SNV | T | G | 1 | 25089 | Hetero | 43 | 101 | 42.6 | 0.30 | 0 | 42 | - | SRP54 |
| 14 | 55243200 | SNV | G | A | 1 | 25089 | Hetero | 65 | 220 | 29.5 | 0.49 | 0 | 128 | No | SAMD4A |
| 14 | 60978779 | SNV | A | T | 1 | 25089 | Hetero | 7 | 65 | 10.8 | 0.29 | 0 | 42 | - | SIX6 |
| 14 | 107013325 | SNV | C | A | 1 | 25089 | Hetero | 41 | 175 | 23.4 | 0.48 | 0 | 96 | - | IGHV3-49 |
| 15 | 29996533 | SNV | C | A | 1 | 25089 | Hetero | 30 | 122 | 24.6 | 0.30 | 0 | 41 | - | TJP1 |
| 15 | 40568493 | SNV | C | T | 1 | 25089 | Hetero | 50 | 186 | 26.9 | 0.38 | 0 | 83 | - | RP11-133K1.2 |
| 15 | 88404985 | SNV | G | T | 1 | 25089 | Hetero | 24 | 112 | 21.4 | 0.38 | 0 | 34 | - | NTRK3 |
| 15 | 59961108 | Insertion | - | A | 1 | 25089 | Hetero | 43 | 112 | 38.4 | 0.47 | 0 | 43 | Insertion | BNIP2 |
| 16 | 3708160 | SNV | C | G | 1 | 25089 | Hetero | 95 | 284 | 33.5 | 0.48 | 0 | 159 | Yes | DNASE1 |
| 16 | 20329562 | SNV | C | T | 1 | 25089 | Hetero | 61 | 174 | 35.1 | 0.49 | 0 | 96 | Yes | GP2 |
| 16 | 21071674 | SNV | T | C | 1 | 25089 | Hetero | 31 | 114 | 27.2 | 0.29 | 0 | 67 | Yes | DNAH3 |
| 17 | 7417246 | SNV | A | T | 1 | 25089 | Hetero | 124 | 399 | 31.1 | 0.48 | 0 | 194 | Yes | POLR2A |
| 17 | 7577610 | SNV | T | A | 1 | 25089 | Hetero | 29 | 78 | 37.2 | 0.29 | 0 | 47 | - | TP53 |
| 17 | 9792735 | SNV | C | T | 1 | 25089 | Hetero | 80 | 218 | 36.7 | 0.33 | 0 | 97 | Yes | GLP2R |
| 17 | 63554536 | SNV | C | T | 1 | 25089 | Hetero | 133 | 376 | 35.4 | 0.45 | 0 | 196 | Yes | AXIN2 |
| 17 | 73315270 | SNV | T | G | 1 | 25089 | Hetero | 33 | 103 | 32.0 | 0.45 | 0 | 47 | - | GRB2 |
| 17 | 15406124 | Deletion | CT | - | 2 | 25089 | Hetero | 7 | 70 | 10.0 | 0.29 | 0 | 34 | - | CDRT4 |
| 18 | 33059363 | SNV | C | G | 1 | 25089 | Hetero | 37 | 138 | 26.8 | 0.34 | 0 | 87 | Yes | INO80C |
| 18 | 61089599 | SNV | G | A | 1 | 25089 | Hetero | 118 | 358 | 33.0 | 0.45 | 0 | 173 | - | VPS4B |
| 18 | 33754701 | Insertion | - | T | 1 | 25089 | Hetero | 16 | 136 | 11.8 | 0.27 | 0 | 52 | - | ELP2 |
| 18 | 42644773 | Insertion | - | A | 1 | 25089 | Hetero | 51 | 81 | 63.0 | 0.29 | 0 | 34 | - | SETBP1 |
| 19 | 14037844 | SNV | A | G | 1 | 25089 | Hetero | 54 | 267 | 20.2 | 0.48 | 0 | 164 | Yes | CC2D1A |
| 19 | 18100584 | SNV | G | A | 1 | 25089 | Hetero | 33 | 77 | 42.9 | 0.47 | 0 | 46 | No | KCNN1 |
| 19 | 33122330 | SNV | G | A | 1 | 25089 | Hetero | 32 | 108 | 29.6 | 0.47 | 0 | 50 | Yes | ANKRD27 |
| 19 | 49637975 | SNV | G | A | 1 | 25089 | Hetero | 90 | 247 | 36.4 | 0.41 | 0 | 128 | - | PPFIA3 |
| 19 | 56466153 | SNV | C | A | 1 | 25089 | Hetero | 33 | 290 | 11.4 | 0.43 | 0 | 197 | Yes | NLRP8 |
| 19 | 10131866 | Insertion | - | T | 1 | 25089 | Hetero | 14 | 67 | 20.9 | 0.07 | 0 | 31 | - | RDH8 |
| 19 | 52786700 | Insertion | - | T | 1 | 25089 | Hetero | 8 | 61 | 13.1 | 0.33 | 0 | 35 | - | ZNF766 |
| 20 | 5172717 | SNV | A | T | 1 | 25089 | Hetero | 51 | 156 | 32.7 | 0.32 | 0 | 91 | - | CDS2 |
| 20 | 13251343 | SNV | C | A | 1 | 25089 | Hetero | 66 | 238 | 27.7 | 0.47 | 0 | 103 | Yes | ISM1 |
| 20 | 13280230 | SNV | A | T | 1 | 25089 | Hetero | 18 | 62 | 29.0 | 0.47 | 0 | 42 | - | ISM1 |
| 20 | 61879054 | SNV | T | A | 1 | 25089 | Hetero | 40 | 118 | 33.9 | 0.20 | 0 | 49 | Yes | NKAIN4 |
| 20 | 62195619 | SNV | C | A | 1 | 25089 | Hetero | 81 | 260 | 31.2 | 0.46 | 0 | 99 | Yes | RP4-697K14.7 |
| 20 | 20016925 | Insertion | - | G | 1 | 25089 | Hetero | 105 | 321 | 32.7 | 0.29 | 0 | 160 | Insertion | CRNKL1 |
| 21 | 43220665 | Deletion | A | - | 1 | 25089 | Hetero | 11 | 79 | 13.9 | 0.27 | 0 | 51 | - | PRDM15 |
| 21 | 47693388 | SNV | G | A | 1 | 25089 | Hetero | 47 | 138 | 34.1 | 0.47 | 0 | 76 | No | MCM3AP |
| 22 | 39130858 | SNV | G | A | 1 | 25089 | Hetero | 25 | 94 | 26.6 | 0.44 | 0 | 39 | - | GTPBP1 |
| 22 | 47570332 | SNV | C | G | 1 | 25089 | Hetero | 26 | 224 | 11.6 | 0.42 | 0 | 112 | - | TBC1D22A |
| 1 | 910821 | SNV | C | G | 1 | 25178 | Hetero | 71 | 167 | 42.5 | 0.38 | 0 | 194 | - | PLEKHN1 |
| 1 | 1277951 | SNV | C | T | 1 | 25178 | Hetero | 21 | 81 | 25.9 | 0.29 | 0 | 76 | - | DVL1 |
| 1 | 1582237 | SNV | A | G | 1 | 25178 | Hetero | 67 | 279 | 24.0 | 0.50 | 0 | 297 | - | CDK11B |
| 1 | 2938319 | SNV | G | A | 1 | 25178 | Hetero | 55 | 105 | 52.4 | 0.34 | 0 | 160 | No | ACTRT2 |
| 1 | 10518103 | SNV | G | A | 1 | 25178 | Hetero | 19 | 42 | 45.2 | 0.32 | 0 | 63 | - | DFFA |
| 1 | 11847604 | SNV | C | T | 1 | 25178 | Hetero | 47 | 98 | 48.0 | 0.37 | 0 | 100 | - | C1orf167 |
| 1 | 13802504 | SNV | G | A | 1 | 25178 | Hetero | 33 | 184 | 17.9 | 0.43 | 0 | 119 | Yes | LRRC38 |
| 1 | 15911230 | SNV | A | T | 1 | 25178 | Hetero | 77 | 164 | 47.0 | 0.37 | 0 | 180 | Yes | AGMAT |
| 1 | 16975087 | SNV | G | T | 1 | 25178 | Hetero | 50 | 320 | 15.6 | 0.37 | 0 | 456 | - | MST1P2 |
| 1 | 17594312 | SNV | C | T | 1 | 25178 | Hetero | 54 | 201 | 26.9 | 0.09 | 0 | 209 | - | PADI3 |
| 1 | 17597371 | SNV | C | A | 1 | 25178 | Hetero | 40 | 94 | 42.6 | 0.21 | 0 | 98 | - | PADI3 |
| 1 | 21076241 | SNV | G | A | 1 | 25178 | Hetero | 52 | 110 | 47.3 | 0.26 | 0 | 123 | No | HP1BP3 |
| 1 | 22313521 | SNV | G | T | 1 | 25178 | Hetero | 28 | 64 | 43.8 | 0.13 | 0 | 183 | - | CELA3B |
| 1 | 22329042 | Deletion | C | - | 1 | 25178 | Hetero | 60 | 190 | 31.6 | 0.08 | 0 | 159 | Deletion | CELA3A |
| 1 | 23382520 | SNV | T | C | 1 | 25178 | Hetero | 104 | 212 | 49.1 | 0.37 | 0 | 194 | No | KDM1A |
| 1 | 23697999 | SNV | G | A | 1 | 25178 | Hetero | 76 | 188 | 40.4 | 0.44 | 0 | 175 | No | C1orf213 |
| 1 | 23713829 | SNV | G | A | 1 | 25178 | Hetero | 35 | 55 | 63.6 | 0.41 | 0 | 67 | No | TCEA3 |
| 1 | 24128405 | SNV | T | C | 1 | 25178 | Hetero | 24 | 47 | 51.1 | 0.26 | 0 | 61 | - | HMGCL |
| 1 | 24294362 | SNV | A | T | 1 | 25178 | Hetero | 137 | 275 | 49.8 | 0.41 | 0 | 320 | - | SRSF10 |
| 1 | 25291064 | SNV | C | T | 1 | 25178 | Hetero | 69 | 281 | 24.6 | 0.36 | 0 | 270 | - | RUNX3 |
| 1 | 25570097 | SNV | T | C | 1 | 25178 | Hetero | 68 | 125 | 54.4 | 0.44 | 0 | 109 | Yes | C1orf63 |
| 1 | 25944169 | SNV | T | C | 1 | 25178 | Hetero | 116 | 245 | 47.3 | 0.48 | 0 | 234 | - | MAN1C1 |
| 1 | 26517239 | SNV | C | A | 1 | 25178 | Hetero | 52 | 99 | 52.5 | 0.41 | 0 | 95 | Yes | CATSPER4 |
| 1 | 26608805 | SNV | A | G | 1 | 25178 | Hetero | 5 | 47 | 10.6 | 0.20 | 0 | 68 | No | UBXN11 |
| 1 | 27248235 | SNV | C | T | 1 | 25178 | Hetero | 22 | 101 | 21.8 | 0.43 | 0 | 100 | - | NUDC |
| 1 | 27277325 | SNV | G | A | 1 | 25178 | Hetero | 24 | 60 | 40.0 | 0.21 | 0 | 68 | - | C1orf172 |
| 1 | 27677271 | SNV | C | T | 1 | 25178 | Hetero | 54 | 194 | 27.8 | 0.23 | 0 | 168 | - | SYTL1 |
| 1 | 32479435 | SNV | C | T | 1 | 25178 | Hetero | 123 | 215 | 57.2 | 0.46 | 0 | 174 | - | KHDRBS1 |
| 1 | 33283080 | SNV | G | T | 1 | 25178 | Hetero | 82 | 171 | 48.0 | 0.47 | 0 | 196 | - | YARS |
| 1 | 34285465 | SNV | T | C | 1 | 25178 | Hetero | 32 | 71 | 45.1 | 0.34 | 0 | 70 | - | CSMD2 |
| 1 | 36884530 | Deletion | G | - | 1 | 25178 | Hetero | 94 | 162 | 58.0 | 0.25 | 0 | 215 | - | OSCP1 |
| 1 | 38031088 | SNV | G | C | 1 | 25178 | Hetero | 42 | 91 | 46.2 | 0.43 | 0 | 103 | - | DNALI1 |
| 1 | 38230581 | SNV | C | T | 1 | 25178 | Hetero | 130 | 250 | 52.0 | 0.42 | 0 | 224 | - | EPHA10 |
| 1 | 38411815 | Deletion | C | - | 1 | 25178 | Hetero | 32 | 59 | 54.2 | 0.09 | 0 | 78 | - | INPP5B |
| 1 | 38456116 | SNV | G | A | 1 | 25178 | Hetero | 53 | 93 | 57.0 | 0.12 | 0 | 99 | - | SF3A3 |
| 1 | 42620305 | Deletion | T | - | 1 | 25178 | Hetero | 27 | 75 | 36.0 | 0.35 | 0 | 77 | - | GUCA2B |
| 1 | 42900930 | SNV | A | G | 1 | 25178 | Hetero | 54 | 110 | 49.1 | 0.27 | 0 | 108 | - | ZMYND12 |
| 1 | 43212427 | SNV | G | C | 1 | 25178 | Hetero | 120 | 264 | 45.5 | 0.31 | 0 | 242 | Yes | LEPRE1 |
| 1 | 53728290 | SNV | G | A | 1 | 25178 | Hetero | 62 | 134 | 46.3 | 0.49 | 0 | 124 | - | LRP8 |
| 1 | 54433804 | SNV | T | C | 1 | 25178 | Hetero | 82 | 133 | 61.7 | 0.17 | 0 | 154 | - | LRRC42 |
| 1 | 54707925 | SNV | G | A | 1 | 25178 | Hetero | 36 | 73 | 49.3 | 0.35 | 0 | 79 | - | SSBP3 |
| 1 | 55505604 | SNV | G | A | 1 | 25178 | Hetero | 18 | 37 | 48.6 | 0.50 | 0 | 31 | Yes | PCSK9 |
| 1 | 63876817 | SNV | A | G | 1 | 25178 | Hetero | 91 | 196 | 46.4 | 0.28 | 0 | 230 | Possible splice site disruption | ALG6 |
| 1 | 65656376 | SNV | T | C | 1 | 25178 | Hetero | 115 | 239 | 48.1 | 0.27 | 0 | 256 | - | AK4 |
| 1 | 77096238 | SNV | T | A | 1 | 25178 | Hetero | 54 | 115 | 47.0 | 0.44 | 0 | 122 | - | ST6GALNAC3 |
| 1 | 87812758 | SNV | T | C | 1 | 25178 | Hetero | 34 | 81 | 42.0 | 0.23 | 0 | 90 | - | LMO4 |
| 1 | 89732236 | SNV | G | A | 1 | 25178 | Hetero | 44 | 95 | 46.3 | 0.11 | 0 | 85 | Yes | GBP5 |
| 1 | 90182599 | SNV | A | G | 1 | 25178 | Hetero | 30 | 72 | 41.7 | 0.48 | 0 | 81 | - | LRRC8C |
| 1 | 91813039 | SNV | T | A | 1 | 25178 | Hetero | 20 | 47 | 42.6 | 0.32 | 0 | 82 | - | HFM1 |
| 1 | 94342279 | SNV | T | C | 1 | 25178 | Hetero | 146 | 287 | 50.9 | 0.43 | 0 | 308 | No | DNTTIP2 |
| 1 | 94351769 | Deletion | T | - | 1 | 25178 | Hetero | 33 | 60 | 55.0 | 0.50 | 0 | 86 | - | GCLM |
| 1 | 120535418 | SNV | A | G | 1 | 25178 | Hetero | 104 | 217 | 47.9 | 0.26 | 0 | 255 | - | NOTCH2 |
| 1 | 120536214 | SNV | C | T | 1 | 25178 | Hetero | 87 | 277 | 31.4 | 0.23 | 0 | 710 | - | NOTCH2 |
| 1 | 120612194 | SNV | C | G | 1 | 25178 | Hetero | 11 | 92 | 12.0 | 0.23 | 0 | 97 | - | NOTCH2 |
| 1 | 145076161 | SNV | C | G | 1 | 25178 | Hetero | 158 | 643 | 24.6 | 0.24 | 0 | 623 | - | PDE4DIP |
| 1 | 145282818 | SNV | G | A | 1 | 25178 | Hetero | 215 | 926 | 23.2 | 0.34 | 0 | 961 | - | NOTCH2NL |
| 1 | 145527941 | SNV | G | A | 1 | 25178 | Hetero | 46 | 102 | 45.1 | 0.31 | 0 | 106 | Yes | ITGA10 |
| 1 | 146317771 | SNV | C | A | 1 | 25178 | Hetero | 27 | 84 | 32.1 | 0.45 | 0 | 116 | - | HYDIN2 |
| 1 | 146334172 | SNV | G | A | 1 | 25178 | Hetero | 29 | 56 | 51.8 | 0.24 | 0 | 60 | - | HYDIN2 |
| 1 | 150691953 | SNV | G | A | 1 | 25178 | Hetero | 42 | 170 | 24.7 | 0.45 | 0 | 196 | Yes | HORMAD1 |
| 1 | 154140110 | SNV | T | G | 1 | 25178 | Hetero | 37 | 75 | 49.3 | 0.18 | 0 | 85 | - | TPM3 |
| 1 | 154453455 | SNV | C | G | 1 | 25178 | Hetero | 42 | 118 | 35.6 | 0.41 | 0 | 136 | - | SHE |
| 1 | 155932014 | SNV | C | G | 1 | 25178 | Hetero | 28 | 161 | 17.4 | 0.07 | 0 | 124 | - | ARHGEF2 |
| 1 | 160195517 | SNV | T | C | 1 | 25178 | Hetero | 38 | 77 | 49.4 | 0.42 | 0 | 79 | - | DCAF8 |
| 1 | 161561363 | SNV | C | T | 1 | 25178 | Hetero | 17 | 43 | 39.5 | 0.24 | 0 | 42 | - | FCGR3A |
| 1 | 161993435 | SNV | C | T | 1 | 25178 | Hetero | 81 | 168 | 48.2 | 0.41 | 0 | 172 | - | OLFML2B |
| 1 | 169526035 | SNV | G | T | 1 | 25178 | Hetero | 42 | 76 | 55.3 | 0.21 | 0 | 88 | Yes | F5 |
| 1 | 179072441 | SNV | G | A | 1 | 25178 | Hetero | 31 | 58 | 53.4 | 0.42 | 0 | 62 | - | ABL2 |
| 1 | 181057831 | SNV | G | A | 1 | 25178 | Hetero | 117 | 200 | 58.5 | 0.47 | 0 | 140 | - | IER5 |
| 1 | 183774235 | SNV | C | A | 1 | 25178 | Hetero | 60 | 126 | 47.6 | 0.33 | 0 | 138 | - | RGL1 |
| 1 | 183774254 | SNV | G | C | 1 | 25178 | Hetero | 54 | 110 | 49.1 | 0.25 | 0 | 125 | - | RGL1 |
| 1 | 183906733 | SNV | C | T | 1 | 25178 | Hetero | 38 | 75 | 50.7 | 0.39 | 0 | 87 | - | GLT25D2 |
| 1 | 185088179 | Deletion | T | - | 1 | 25178 | Hetero | 44 | 99 | 44.4 | 0.38 | 0 | 115 | - | TRMT1L |
| 1 | 186649496 | SNV | G | C | 1 | 25178 | Hetero | 40 | 84 | 47.6 | 0.47 | 0 | 91 | - | PTGS2 |
| 1 | 197071124 | SNV | T | C | 1 | 25178 | Hetero | 86 | 162 | 53.1 | 0.33 | 0 | 240 | No | ASPM |
| 1 | 197412636 | SNV | A | G | 1 | 25178 | Hetero | 14 | 32 | 43.8 | 0.43 | 0 | 42 | - | CRB1 |
| 1 | 201338978 | SNV | G | A | 1 | 25178 | Hetero | 64 | 141 | 45.4 | 0.43 | 0 | 129 | - | TNNT2 |
| 1 | 201794746 | SNV | C | T | 1 | 25178 | Hetero | 44 | 114 | 38.6 | 0.33 | 0 | 117 | - | NAV1 |
| 1 | 203277248 | SNV | G | C | 1 | 25178 | Hetero | 41 | 84 | 48.8 | 0.39 | 0 | 70 | - | BTG2 |
| 1 | 206331744 | SNV | G | A | 1 | 25178 | Hetero | 54 | 107 | 50.5 | 0.33 | 0 | 111 | - | CTSE |
| 1 | 207785158 | SNV | G | A | 1 | 25178 | Hetero | 42 | 119 | 35.3 | 0.30 | 0 | 140 | No | CR1 |
| 1 | 207930437 | SNV | G | A | 1 | 25178 | Hetero | 88 | 179 | 49.2 | 0.21 | 0 | 193 | Yes | CD46 |
| 1 | 211192501 | SNV | G | A | 1 | 25178 | Hetero | 59 | 129 | 45.7 | 0.36 | 0 | 163 | Yes | KCNH1 |
| 1 | 212965444 | SNV | T | C | 1 | 25178 | Hetero | 61 | 138 | 44.2 | 0.26 | 0 | 147 | - | TATDN3 |
| 1 | 214549727 | SNV | C | A | 1 | 25178 | Hetero | 44 | 98 | 44.9 | 0.49 | 0 | 121 | Yes | PTPN14 |
| 1 | 217781559 | Deletion | A | - | 1 | 25178 | Hetero | 11 | 31 | 35.5 | 0.25 | 0 | 43 | - | GPATCH2 |
| 1 | 220863685 | SNV | G | A | 1 | 25178 | Hetero | 29 | 60 | 48.3 | 0.39 | 0 | 70 | - | C1orf115 |
| 1 | 222791599 | SNV | A | G | 1 | 25178 | Hetero | 55 | 113 | 48.7 | 0.41 | 0 | 154 | - | MIA3 |
| 1 | 222801868 | SNV | G | A | 1 | 25178 | Hetero | 65 | 130 | 50.0 | 0.32 | 0 | 188 | Yes | MIA3 |
| 1 | 225239246 | SNV | G | A | 1 | 25178 | Hetero | 14 | 31 | 45.2 | 0.47 | 0 | 39 | Yes | DNAH14 |
| 1 | 226107965 | SNV | G | A | 1 | 25178 | Hetero | 58 | 109 | 53.2 | 0.45 | 0 | 136 | - | RP4-559A3.7 |
| 1 | 226254947 | SNV | T | C | 1 | 25178 | Hetero | 22 | 52 | 42.3 | 0.09 | 0 | 49 | - | H3F3A |
| 1 | 227506004 | SNV | A | G | 1 | 25178 | Hetero | 109 | 233 | 46.8 | 0.28 | 0 | 254 | - | CDC42BPA |
| 1 | 228481080 | SNV | C | T | 1 | 25178 | Hetero | 61 | 141 | 43.3 | 0.36 | 0 | 182 | No | OBSCN |
| 1 | 230503756 | SNV | C | T | 1 | 25178 | Hetero | 16 | 74 | 21.6 | 0.29 | 0 | 86 | Yes | PGBD5 |
| 1 | 230898527 | SNV | C | T | 1 | 25178 | Hetero | 66 | 158 | 41.8 | 0.40 | 0 | 187 | No | CAPN9 |
| 1 | 243329075 | SNV | T | C | 1 | 25178 | Hetero | 49 | 273 | 17.9 | 0.37 | 0 | 294 | No | CEP170 |
| 1 | 244724183 | SNV | G | A | 1 | 25178 | Hetero | 49 | 165 | 29.7 | 0.42 | 0 | 198 | Yes | C1orf101 |
| 1 | 246830647 | SNV | A | T | 1 | 25178 | Hetero | 11 | 72 | 15.3 | 0.42 | 0 | 79 | - | CNST |
| 1 | 246943889 | SNV | G | A | 1 | 25178 | Hetero | 109 | 249 | 43.8 | 0.44 | 0 | 290 | - | RP11-439E19.3 |
| 1 | 247486494 | SNV | G | A | 1 | 25178 | Hetero | 18 | 46 | 39.1 | 0.41 | 0 | 65 | Yes | ZNF496 |
| 1 | 248637199 | SNV | T | C | 1 | 25178 | Hetero | 11 | 56 | 19.6 | 0.09 | 0 | 457 | Yes | OR2T3 |
| 1 | 248722671 | SNV | T | A | 1 | 25178 | Homo | 94 | 102 | 92.2 | 0.19 | 0 | 155 | Yes | RP11-438F14.3 |
| 1 | 249105901 | SNV | G | A | 1 | 25178 | Hetero | 54 | 102 | 52.9 | 0.47 | 0 | 156 | - | SH3BP5L |
| 1 | 100159552 | Insertion | - | T | 1 | 25178 | Hetero | 50 | 117 | 42.7 | 0.18 | 0 | 139 | - | PALMD |
| 1 | 10132493 | Insertion | - | T | 1 | 25178 | Hetero | 27 | 83 | 32.5 | 0.17 | 0 | 92 | - | UBE4B |
| 1 | 114227223 | Insertion | - | A | 1 | 25178 | Hetero | 32 | 80 | 40.0 | 0.30 | 0 | 88 | - | MAGI3 |
| 1 | 114524265 | Insertion | - | C | 1 | 25178 | Hetero | 82 | 161 | 50.9 | 0.42 | 0 | 241 | Insertion | OLFML3 |
| 1 | 117529859 | Insertion | - | T | 1 | 25178 | Hetero | 29 | 60 | 48.3 | 0.43 | 0 | 85 | - | PTGFRN |
| 1 | 154310223 | Insertion | - | A | 1 | 25178 | Homo | 233 | 236 | 98.7 | 0.46 | 0 | 255 | - | ATP8B2 |
| 1 | 161953015 | Insertion | - | G | 1 | 25178 | Hetero | 37 | 86 | 43.0 | 0.26 | 0 | 68 | - | OLFML2B |
| 1 | 180082703 | Insertion | - | A | 1 | 25178 | Homo | 69 | 71 | 97.2 | 0.43 | 0 | 76 | - | CEP350 |
| 1 | 183191211 | Insertion | - | T | 1 | 25178 | Hetero | 42 | 108 | 38.9 | 0.39 | 0 | 93 | - | LAMC2 |
| 1 | 185069314 | Insertion | - | T | 1 | 25178 | Hetero | 43 | 84 | 51.2 | 0.11 | 0 | 106 | - | RNF2 |
| 1 | 201926386 | Insertion | - | T | 1 | 25178 | Homo | 176 | 178 | 98.9 | 0.30 | 0 | 218 | - | TIMM17A |
| 1 | 208200200 | Insertion | - | TT | 2 | 25178 | Hetero | 50 | 84 | 59.5 | 0.16 | 0 | 82 | - | PLXNA2 |
| 1 | 20945252 | Insertion | - | C | 1 | 25178 | Hetero | 56 | 128 | 43.8 | 0.21 | 0 | 144 | - | CDA |
| 1 | 236988593 | Insertion | - | T | 1 | 25178 | Homo | 69 | 69 | 100.0 | 0.16 | 0 | 87 | - | MTR |
| 1 | 24683536 | Insertion | - | CC | 2 | 25178 | Hetero | 11 | 38 | 28.9 | 0.42 | 0 | 61 | - | GRHL3 |
| 1 | 27682440 | Insertion | - | G | 1 | 25178 | Hetero | 43 | 100 | 43.0 | 0.32 | 0 | 85 | - | MAP3K6 |
| 1 | 33322690 | Insertion | - | T | 1 | 25178 | Hetero | 42 | 66 | 63.6 | 0.40 | 0 | 77 | - | S100PBP |
| 1 | 39793057 | Insertion | - | G | 1 | 25178 | Hetero | 108 | 149 | 72.5 | 0.23 | 0 | 115 | - | MACF1 |
| 1 | 47142543 | Insertion | - | A | 1 | 25178 | Hetero | 37 | 79 | 46.8 | 0.48 | 0 | 100 | - | KIAA0494 |
| 1 | 59121144 | Insertion | - | T | 1 | 25178 | Hetero | 35 | 76 | 46.1 | 0.49 | 0 | 69 | - | MYSM1 |
| 1 | 63903846 | Insertion | - | T | 1 | 25178 | Hetero | 32 | 86 | 37.2 | 0.47 | 0 | 86 | - | ALG6 |
| 1 | 75172757 | Deletion | AA | - | 2 | 25178 | Hetero | 82 | 168 | 48.8 | 0.33 | 0 | 190 | - | CRYZ |
| 1 | 792281 | Deletion | TT | - | 2 | 25178 | Hetero | 28 | 60 | 46.7 | 0.30 | 0 | 75 | - | RP11-206L10.11 |
| 1 | 85717915 | Deletion | AC | - | 2 | 25178 | Hetero | 16 | 36 | 44.4 | 0.41 | 0 | 38 | - | C1orf52 |
| 1 | 87573326 | Deletion | AT | - | 2 | 25178 | Hetero | 32 | 51 | 62.7 | 0.29 | 0 | 54 | - | HS2ST1 |
| 1 | 90472880 | Insertion | - | C | 1 | 25178 | Hetero | 39 | 68 | 57.4 | 0.18 | 0 | 62 | - | ZNF326 |
| 1 | 9163163 | Insertion | - | AA | 2 | 25178 | Hetero | 17 | 94 | 18.1 | 0.36 | 0 | 90 | - | GPR157 |
| 1 | 9429264 | Insertion | - | T | 1 | 25178 | Hetero | 31 | 85 | 36.5 | 0.44 | 0 | 77 | - | SPSB1 |
| 2 | 1793989 | Deletion | T | - | 1 | 25178 | Hetero | 30 | 52 | 57.7 | 0.42 | 0 | 87 | - | MYT1L |
| 2 | 3502293 | SNV | G | A | 1 | 25178 | Hetero | 60 | 127 | 47.2 | 0.32 | 0 | 116 | - | ADI1 |
| 2 | 8996501 | SNV | T | C | 1 | 25178 | Hetero | 33 | 84 | 39.3 | 0.41 | 0 | 76 | - | MBOAT2 |
| 2 | 9544874 | SNV | T | C | 1 | 25178 | Hetero | 40 | 70 | 57.1 | 0.45 | 0 | 86 | - | ASAP2 |
| 2 | 10198248 | SNV | G | A | 1 | 25178 | Hetero | 41 | 105 | 39.0 | 0.36 | 0 | 110 | - | CYS1 |
| 2 | 11781282 | SNV | C | G | 1 | 25178 | Hetero | 17 | 38 | 44.7 | 0.33 | 0 | 52 | - | GREB1 |
| 2 | 17962460 | SNV | C | T | 1 | 25178 | Hetero | 71 | 137 | 51.8 | 0.34 | 0 | 129 | Yes | SMC6 |
| 2 | 20101229 | SNV | C | A | 1 | 25178 | Hetero | 89 | 173 | 51.4 | 0.45 | 0 | 206 | - | TTC32 |
| 2 | 25961196 | SNV | G | C | 1 | 25178 | Hetero | 15 | 33 | 45.5 | 0.44 | 0 | 32 | - | ASXL2 |
| 2 | 26150211 | SNV | G | A | 1 | 25178 | Hetero | 47 | 132 | 35.6 | 0.47 | 0 | 129 | - | KIF3C |
| 2 | 26613955 | SNV | C | T | 1 | 25178 | Hetero | 26 | 59 | 44.1 | 0.45 | 0 | 50 | - | EPT1 |
| 2 | 27930040 | Deletion | C | - | 1 | 25178 | Hetero | 32 | 60 | 53.3 | 0.19 | 0 | 73 | - | AC074091.13 |
| 2 | 29245993 | SNV | C | T | 1 | 25178 | Hetero | 94 | 195 | 48.2 | 0.37 | 0 | 225 | Yes | FAM179A |
| 2 | 29455255 | SNV | G | A | 1 | 25178 | Hetero | 109 | 204 | 53.4 | 0.34 | 0 | 203 | No | ALK |
| 2 | 30785038 | SNV | A | G | 1 | 25178 | Hetero | 94 | 186 | 50.5 | 0.26 | 0 | 171 | Yes | LCLAT1 |
| 2 | 30865982 | SNV | T | G | 1 | 25178 | Hetero | 8 | 52 | 15.4 | 0.50 | 0 | 60 | - | LCLAT1 |
| 2 | 31620502 | SNV | A | T | 1 | 25178 | Hetero | 35 | 136 | 25.7 | 0.37 | 0 | 151 | - | XDH |
| 2 | 32249172 | SNV | G | A | 1 | 25178 | Hetero | 49 | 103 | 47.6 | 0.46 | 0 | 105 | - | DPY30 |
| 2 | 43991390 | SNV | C | T | 1 | 25178 | Hetero | 24 | 68 | 35.3 | 0.19 | 0 | 63 | No | PLEKHH2 |
| 2 | 61245046 | SNV | G | C | 1 | 25178 | Hetero | 108 | 203 | 53.2 | 0.41 | 0 | 186 | - | PUS10 |
| 2 | 64861512 | Deletion | G | - | 1 | 25178 | Hetero | 41 | 72 | 56.9 | 0.17 | 0 | 82 | - | SERTAD2 |
| 2 | 65249188 | SNV | A | G | 1 | 25178 | Hetero | 26 | 58 | 44.8 | 0.30 | 0 | 66 | - | SLC1A4 |
| 2 | 73114691 | SNV | G | C | 1 | 25178 | Hetero | 21 | 53 | 39.6 | 0.39 | 0 | 50 | Yes | SPR |
| 2 | 73448969 | SNV | C | T | 1 | 25178 | Hetero | 64 | 171 | 37.4 | 0.42 | 0 | 219 | Yes | SMYD5 |
| 2 | 73679799 | SNV | T | A | 1 | 25178 | Hetero | 189 | 371 | 50.9 | 0.37 | 0 | 415 | Yes | ALMS1 |
| 2 | 73717908 | SNV | A | G | 1 | 25178 | Hetero | 154 | 271 | 56.8 | 0.41 | 0 | 325 | Yes | ALMS1 |
| 2 | 74092353 | SNV | G | A | 1 | 25178 | Hetero | 54 | 101 | 53.5 | 0.44 | 0 | 88 | - | STAMBP |
| 2 | 75099594 | SNV | A | G | 1 | 25178 | Hetero | 111 | 258 | 43.0 | 0.36 | 0 | 278 | - | HK2 |
| 2 | 80136915 | SNV | A | C | 1 | 25178 | Hetero | 62 | 119 | 52.1 | 0.36 | 0 | 146 | Yes | CTNNA2 |
| 2 | 86297326 | SNV | T | A | 1 | 25178 | Hetero | 34 | 126 | 27.0 | 0.49 | 0 | 135 | Yes | POLR1A |
| 2 | 86678190 | SNV | G | A | 1 | 25178 | Hetero | 42 | 70 | 60.0 | 0.32 | 0 | 41 | - | KDM3A |
| 2 | 88055889 | SNV | G | A | 1 | 25178 | Hetero | 60 | 232 | 25.9 | 0.13 | 0 | 330 | - | PLGLB2 |
| 2 | 88055929 | SNV | A | C | 1 | 25178 | Hetero | 112 | 419 | 26.7 | 0.41 | 0 | 408 | - | PLGLB2 |
| 2 | 88056000 | SNV | T | C | 1 | 25178 | Hetero | 132 | 457 | 28.9 | 0.39 | 0 | 380 | - | PLGLB2 |
| 2 | 88056464 | SNV | T | C | 1 | 25178 | Hetero | 161 | 465 | 34.6 | 0.40 | 0 | 372 | - | PLGLB2 |
| 2 | 88826108 | SNV | C | A | 1 | 25178 | Hetero | 42 | 87 | 48.3 | 0.27 | 0 | 92 | - | C2orf51 |
| 2 | 91766609 | SNV | G | A | 1 | 25178 | Hetero | 98 | 741 | 13.2 | 0.27 | 0 | 915 | - | ENSG00000230964 |
| 2 | 97359338 | SNV | C | T | 1 | 25178 | Hetero | 63 | 136 | 46.3 | 0.39 | 0 | 161 | - | FER1L5 |
| 2 | 97427277 | SNV | C | A | 1 | 25178 | Hetero | 68 | 207 | 32.9 | 0.49 | 0 | 224 | Yes | CNNM4 |
| 2 | 97877478 | SNV | G | A | 1 | 25178 | Hetero | 57 | 179 | 31.8 | 0.41 | 0 | 180 | Possible splice site disruption | ANKRD36 |
| 2 | 102019108 | SNV | T | G | 1 | 25178 | Hetero | 137 | 281 | 48.8 | 0.41 | 0 | 279 | Yes | RFX8 |
| 2 | 107107488 | SNV | G | A | 1 | 25178 | Hetero | 78 | 147 | 53.1 | 0.43 | 0 | 163 | - | AC108868.4 |
| 2 | 107419796 | SNV | A | G | 1 | 25178 | Hetero | 41 | 88 | 46.6 | 0.48 | 0 | 93 | - | ST6GAL2 |
| 2 | 110260147 | SNV | C | T | 1 | 25178 | Hetero | 44 | 93 | 47.3 | 0.49 | 0 | 91 | - | SH3RF3 |
| 2 | 111923064 | SNV | G | T | 1 | 25178 | Hetero | 71 | 130 | 54.6 | 0.35 | 0 | 143 | - | BCL2L11 |
| 2 | 119739128 | SNV | T | A | 1 | 25178 | Hetero | 7 | 60 | 11.7 | 0.29 | 0 | 77 | - | MARCO |
| 2 | 121979217 | SNV | G | C | 1 | 25178 | Hetero | 53 | 104 | 51.0 | 0.42 | 0 | 106 | - | TFCP2L1 |
| 2 | 128394877 | SNV | C | T | 1 | 25178 | Hetero | 130 | 265 | 49.1 | 0.38 | 0 | 238 | - | MYO7B |
| 2 | 128463749 | SNV | A | G | 1 | 25178 | Hetero | 41 | 81 | 50.6 | 0.49 | 0 | 68 | - | WDR33 |
| 2 | 129025148 | SNV | C | T | 1 | 25178 | Hetero | 23 | 55 | 41.8 | 0.43 | 0 | 70 | - | HS6ST1 |
| 2 | 131519854 | SNV | C | T | 1 | 25178 | Hetero | 60 | 116 | 51.7 | 0.42 | 0 | 119 | Yes | FAM123C |
| 2 | 132250067 | SNV | G | A | 1 | 25178 | Hetero | 45 | 74 | 60.8 | 0.37 | 0 | 59 | - | MZT2A |
| 2 | 138000059 | SNV | G | A | 1 | 25178 | Hetero | 69 | 153 | 45.1 | 0.35 | 0 | 183 | Yes | THSD7B |
| 2 | 149544914 | SNV | T | C | 1 | 25178 | Hetero | 52 | 120 | 43.3 | 0.45 | 0 | 117 | - | EPC2 |
| 2 | 160175529 | SNV | T | C | 1 | 25178 | Hetero | 46 | 92 | 50.0 | 0.21 | 0 | 92 | - | BAZ2B |
| 2 | 165945168 | SNV | T | C | 1 | 25178 | Hetero | 27 | 59 | 45.8 | 0.42 | 0 | 67 | - | AC013463.2 |
| 2 | 170681284 | SNV | T | C | 1 | 25178 | Hetero | 80 | 166 | 48.2 | 0.48 | 0 | 188 | - | METTL5 |
| 2 | 171916563 | Deletion | A | - | 1 | 25178 | Hetero | 66 | 136 | 48.5 | 0.42 | 0 | 144 | - | TLK1 |
| 2 | 172340571 | SNV | A | C | 1 | 25178 | Hetero | 57 | 101 | 56.4 | 0.46 | 0 | 120 | - | DCAF17 |
| 2 | 175260511 | SNV | C | T | 1 | 25178 | Hetero | 108 | 219 | 49.3 | 0.29 | 0 | 291 | No | SCRN3 |
| 2 | 178479976 | SNV | A | G | 1 | 25178 | Hetero | 42 | 76 | 55.3 | 0.28 | 0 | 83 | - | AC073834.3 |
| 2 | 179665358 | SNV | C | G | 1 | 25178 | Hetero | 19 | 42 | 45.2 | 0.35 | 0 | 47 | Yes | TTN |
| 2 | 182423343 | SNV | C | T | 1 | 25178 | Hetero | 28 | 58 | 48.3 | 0.23 | 0 | 50 | Yes | CERKL |
| 2 | 182928576 | SNV | G | A | 1 | 25178 | Hetero | 45 | 84 | 53.6 | 0.32 | 0 | 112 | - | PPP1R1C |
| 2 | 187558792 | SNV | T | C | 1 | 25178 | Hetero | 53 | 108 | 49.1 | 0.43 | 0 | 116 | - | FAM171B |
| 2 | 191556283 | SNV | G | A | 1 | 25178 | Hetero | 72 | 134 | 53.7 | 0.43 | 0 | 134 | - | NAB1 |
| 2 | 191855977 | SNV | C | G | 1 | 25178 | Hetero | 25 | 59 | 42.4 | 0.38 | 0 | 73 | No | STAT1 |
| 2 | 197792325 | SNV | G | A | 1 | 25178 | Hetero | 47 | 188 | 25.0 | 0.49 | 0 | 224 | No | PGAP1 |
| 2 | 197866465 | SNV | G | A | 1 | 25178 | Hetero | 33 | 55 | 60.0 | 0.49 | 0 | 64 | - | ANKRD44 |
| 2 | 200776353 | SNV | T | C | 1 | 25178 | Hetero | 45 | 111 | 40.5 | 0.48 | 0 | 126 | No | C2orf69 |
| 2 | 200791068 | SNV | G | T | 1 | 25178 | Hetero | 36 | 83 | 43.4 | 0.44 | 0 | 108 | - | C2orf69 |
| 2 | 201845944 | SNV | A | G | 1 | 25178 | Hetero | 146 | 256 | 57.0 | 0.28 | 0 | 292 | - | FAM126B |
| 2 | 202028920 | SNV | C | A | 1 | 25178 | Hetero | 38 | 105 | 36.2 | 0.50 | 0 | 106 | - | CFLAR |
| 2 | 202487001 | Deletion | A | - | 1 | 25178 | Hetero | 36 | 67 | 53.7 | 0.50 | 0 | 61 | - | TMEM237 |
| 2 | 203379739 | SNV | C | T | 1 | 25178 | Hetero | 76 | 161 | 47.2 | 0.30 | 0 | 190 | - | BMPR2 |
| 2 | 203807653 | SNV | A | G | 1 | 25178 | Hetero | 34 | 212 | 16.0 | 0.31 | 0 | 270 | Yes | WDR12 |
| 2 | 203849780 | Deletion | A | - | 1 | 25178 | Hetero | 5 | 48 | 10.4 | 0.17 | 0 | 40 | - | WDR12 |
| 2 | 210888820 | SNV | A | G | 1 | 25178 | Hetero | 11 | 100 | 11.0 | 0.50 | 0 | 84 | No | KANSL1L |
| 2 | 212245958 | SNV | T | A | 1 | 25178 | Hetero | 53 | 102 | 52.0 | 0.45 | 0 | 139 | - | ERBB4 |
| 2 | 212247090 | SNV | G | A | 1 | 25178 | Hetero | 58 | 97 | 59.8 | 0.41 | 0 | 110 | - | ERBB4 |
| 2 | 217525646 | SNV | G | C | 1 | 25178 | Hetero | 30 | 46 | 65.2 | 0.26 | 0 | 62 | - | IGFBP2 |
| 2 | 219294201 | SNV | A | T | 1 | 25178 | Hetero | 75 | 252 | 29.8 | 0.41 | 0 | 267 | Yes | VIL1 |
| 2 | 220470900 | SNV | C | G | 1 | 25178 | Hetero | 156 | 325 | 48.0 | 0.50 | 0 | 384 | - | STK11IP |
| 2 | 220498004 | SNV | A | G | 1 | 25178 | Hetero | 135 | 328 | 41.2 | 0.38 | 0 | 322 | Yes | SLC4A3 |
| 2 | 224616622 | SNV | C | T | 1 | 25178 | Hetero | 12 | 30 | 40.0 | 0.40 | 0 | 39 | - | AP1S3 |
| 2 | 225244958 | SNV | A | G | 1 | 25178 | Hetero | 23 | 45 | 51.1 | 0.15 | 0 | 61 | - | FAM124B |
| 2 | 227773476 | Deletion | G | - | 1 | 25178 | Hetero | 30 | 50 | 60.0 | 0.23 | 0 | 55 | - | RHBDD1 |
| 2 | 228004969 | SNV | A | G | 1 | 25178 | Hetero | 78 | 181 | 43.1 | 0.14 | 0 | 248 | - | COL4A4 |
| 2 | 228552096 | SNV | C | T | 1 | 25178 | Hetero | 91 | 207 | 44.0 | 0.32 | 0 | 236 | - | SLC19A3 |
| 2 | 239241566 | SNV | T | C | 1 | 25178 | Hetero | 51 | 97 | 52.6 | 0.34 | 0 | 111 | - | TRAF3IP1 |
| 2 | 241626317 | SNV | A | G | 1 | 25178 | Hetero | 28 | 47 | 59.6 | 0.48 | 0 | 68 | - | AC011298.2 |
| 2 | 241709127 | SNV | G | T | 1 | 25178 | Hetero | 38 | 80 | 47.5 | 0.49 | 0 | 88 | - | KIF1A |
| 2 | 242433866 | SNV | T | A | 1 | 25178 | Hetero | 26 | 243 | 10.7 | 0.22 | 0 | 255 | - | FARP2 |
| 2 | 100006904 | Insertion | - | A | 1 | 25178 | Hetero | 28 | 107 | 26.2 | 0.45 | 0 | 142 | - | EIF5B |
| 2 | 105474220 | Insertion | - | A | 1 | 25178 | Hetero | 43 | 69 | 62.3 | 0.34 | 0 | 69 | - | POU3F3 |
| 2 | 14779227 | Insertion | - | AC | 2 | 25178 | Hetero | 13 | 40 | 32.5 | 0.23 | 0 | 49 | - | FAM84A |
| 2 | 170606655 | Deletion | TG | - | 2 | 25178 | Hetero | 47 | 114 | 41.2 | 0.16 | 0 | 123 | - | KLHL23 |
| 2 | 175617482 | Insertion | - | TT | 2 | 25178 | Hetero | 11 | 75 | 14.7 | 0.27 | 0 | 55 | - | AC018890.6 |
| 2 | 200135086 | Insertion | - | T | 1 | 25178 | Hetero | 23 | 56 | 41.1 | 0.39 | 0 | 50 | - | SATB2 |
| 2 | 224903353 | Insertion | - | T | 1 | 25178 | Hetero | 53 | 184 | 28.8 | 0.44 | 0 | 213 | - | SERPINE2 |
| 2 | 225630257 | Insertion | - | A | 1 | 25178 | Hetero | 43 | 87 | 49.4 | 0.45 | 0 | 87 | - | DOCK10 |
| 2 | 232458074 | Insertion | - | C | 1 | 25178 | Hetero | 117 | 286 | 40.9 | 0.48 | 0 | 306 | Insertion | C2orf57 |
| 2 | 38527397 | Insertion | - | CT | 2 | 25178 | Hetero | 32 | 114 | 28.1 | 0.50 | 0 | 100 | - | ATL2 |
| 2 | 54888872 | Insertion | - | AT | 2 | 25178 | Hetero | 33 | 84 | 39.3 | 0.45 | 0 | 97 | - | SPTBN1 |
| 2 | 75120159 | Insertion | - | TT | 2 | 25178 | Hetero | 16 | 64 | 25.0 | 0.28 | 0 | 70 | - | HK2 |
| 2 | 76975666 | Insertion | - | T | 1 | 25178 | Hetero | 17 | 33 | 51.5 | 0.26 | 0 | 47 | - | LRRTM4 |
| 2 | 88485968 | Insertion | - | C | 1 | 25178 | Hetero | 90 | 151 | 59.6 | 0.40 | 0 | 131 | - | THNSL2 |
| 2 | 9629280 | Insertion | - | T | 1 | 25178 | Hetero | 17 | 42 | 40.5 | 0.12 | 0 | 57 | - | ADAM17 |
| 2 | 9629943 | Insertion | - | A | 1 | 25178 | Hetero | 28 | 71 | 39.4 | 0.25 | 0 | 66 | - | ADAM17 |
| 2 | 96690478 | Insertion | - | C | 1 | 25178 | Hetero | 54 | 119 | 45.4 | 0.38 | 0 | 114 | - | GPAT2 |
| 2 | 97499144 | Insertion | - | C | 1 | 25178 | Hetero | 58 | 112 | 51.8 | 0.46 | 0 | 124 | - | CNNM3 |
| 3 | 4403357 | SNV | A | C | 1 | 25178 | Homo | 64 | 64 | 100.0 | 0.45 | 0 | 107 | - | SUMF1 |
| 3 | 7678029 | SNV | G | A | 1 | 25178 | Hetero | 70 | 90 | 77.8 | 0.25 | 0 | 228 | - | GRM7 |
| 3 | 9426359 | Deletion | A | - | 1 | 25178 | Hetero | 50 | 219 | 22.8 | 0.34 | 0 | 348 | Deletion | THUMPD3 |
| 3 | 9785336 | SNV | C | T | 1 | 25178 | Hetero | 60 | 196 | 30.6 | 0.36 | 0 | 257 | No | BRPF1 |
| 3 | 9798902 | SNV | T | A | 1 | 25178 | Hetero | 208 | 294 | 70.7 | 0.35 | 0 | 431 | Yes | OGG1 |
| 3 | 9982563 | SNV | G | A | 1 | 25178 | Hetero | 42 | 55 | 76.4 | 0.30 | 0 | 71 | Yes | CRELD1 |
| 3 | 10367084 | SNV | G | A | 1 | 25178 | Hetero | 45 | 56 | 80.4 | 0.25 | 0 | 95 | - | ATP2B2 |
| 3 | 12776241 | SNV | G | A | 1 | 25178 | Hetero | 19 | 71 | 26.8 | 0.41 | 0 | 89 | - | TMEM40 |
| 3 | 13612633 | SNV | C | G | 1 | 25178 | Hetero | 19 | 87 | 21.8 | 0.43 | 0 | 151 | Yes | FBLN2 |
| 3 | 14239518 | Deletion | T | - | 1 | 25178 | Hetero | 21 | 63 | 33.3 | 0.45 | 0 | 96 | - | LSM3 |
| 3 | 14240726 | SNV | G | C | 1 | 25178 | Hetero | 7 | 37 | 18.9 | 0.44 | 0 | 85 | - | LSM3 |
| 3 | 14724048 | SNV | G | T | 1 | 25178 | Hetero | 106 | 135 | 78.5 | 0.23 | 0 | 225 | - | C3orf20 |
| 3 | 17131792 | SNV | A | G | 1 | 25178 | Hetero | 44 | 59 | 74.6 | 0.40 | 0 | 87 | - | PLCL2 |
| 3 | 43097979 | SNV | G | A | 1 | 25178 | Hetero | 66 | 88 | 75.0 | 0.49 | 0 | 106 | - | FAM198A |
| 3 | 44285548 | SNV | A | C | 1 | 25178 | Hetero | 41 | 138 | 29.7 | 0.37 | 0 | 254 | Yes | C3orf77 |
| 3 | 44943360 | SNV | C | T | 1 | 25178 | Hetero | 88 | 121 | 72.7 | 0.46 | 0 | 138 | Yes | TGM4 |
| 3 | 45786343 | SNV | T | C | 1 | 25178 | Hetero | 22 | 34 | 64.7 | 0.48 | 0 | 55 | - | SACM1L |
| 3 | 45807203 | SNV | C | T | 1 | 25178 | Hetero | 64 | 109 | 58.7 | 0.43 | 0 | 161 | Yes | SLC6A20 |
| 3 | 48605076 | Deletion | A | - | 1 | 25178 | Hetero | 142 | 190 | 74.7 | 0.49 | 0 | 226 | - | COL7A1 |
| 3 | 52802623 | Deletion | A | - | 1 | 25178 | Hetero | 17 | 51 | 33.3 | 0.24 | 0 | 63 | - | NEK4 |
| 3 | 52847559 | SNV | G | A | 1 | 25178 | Hetero | 22 | 97 | 22.7 | 0.29 | 0 | 117 | - | ITIH4 |
| 3 | 52868717 | Deletion | C | - | 1 | 25178 | Hetero | 124 | 169 | 73.4 | 0.30 | 0 | 251 | - | MUSTN1 |
| 3 | 61547944 | SNV | C | A | 1 | 25178 | Hetero | 99 | 148 | 66.9 | 0.48 | 0 | 232 | - | PTPRG |
| 3 | 72425147 | SNV | T | A | 1 | 25178 | Hetero | 28 | 73 | 38.4 | 0.48 | 0 | 115 | - | RYBP |
| 3 | 72957347 | SNV | G | T | 1 | 25178 | Hetero | 13 | 52 | 25.0 | 0.38 | 0 | 111 | - | GXYLT2 |
| 3 | 75714382 | SNV | G | T | 1 | 25178 | Hetero | 75 | 407 | 18.4 | 0.38 | 0 | 615 | - | FRG2C |
| 3 | 75715355 | SNV | G | A | 1 | 25178 | Hetero | 209 | 1070 | 19.5 | 0.49 | 0 | 1188 | - | FRG2C |
| 3 | 75715450 | SNV | G | T | 1 | 25178 | Hetero | 125 | 619 | 20.2 | 0.16 | 0 | 653 | - | FRG2C |
| 3 | 75715822 | SNV | A | G | 1 | 25178 | Hetero | 14 | 110 | 12.7 | 0.36 | 0 | 134 | - | FRG2C |
| 3 | 75781152 | SNV | A | G | 1 | 25178 | Hetero | 28 | 179 | 15.6 | 0.07 | 0 | 208 | - | ZNF717 |
| 3 | 75781300 | SNV | T | C | 1 | 25178 | Hetero | 38 | 193 | 19.7 | 0.29 | 0 | 191 | - | ZNF717 |
| 3 | 75785009 | SNV | G | A | 1 | 25178 | Hetero | 23 | 91 | 25.3 | 0.19 | 0 | 93 | - | ZNF717 |
| 3 | 75785367 | SNV | A | G | 1 | 25178 | Hetero | 29 | 129 | 22.5 | 0.47 | 0 | 180 | - | ZNF717 |
| 3 | 75786337 | SNV | G | T | 1 | 25178 | Hetero | 35 | 268 | 13.1 | 0.28 | 0 | 333 | Yes | ZNF717 |
| 3 | 75786687 | SNV | A | T | 1 | 25178 | Hetero | 30 | 189 | 15.9 | 0.22 | 0 | 236 | Yes | ZNF717 |
| 3 | 75786921 | SNV | G | A | 1 | 25178 | Hetero | 22 | 192 | 11.5 | 0.48 | 0 | 249 | Yes | ZNF717 |
| 3 | 75787174 | SNV | G | T | 1 | 25178 | Hetero | 40 | 330 | 12.1 | 0.23 | 0 | 515 | Yes | ZNF717 |
| 3 | 75787476 | SNV | C | T | 1 | 25178 | Hetero | 64 | 219 | 29.2 | 0.36 | 0 | 253 | Yes | ZNF717 |
| 3 | 75788411 | SNV | G | C | 1 | 25178 | Hetero | 27 | 163 | 16.6 | 0.32 | 0 | 254 | Yes | ZNF717 |
| 3 | 86117274 | SNV | C | A | 1 | 25178 | Hetero | 13 | 66 | 19.7 | 0.50 | 0 | 91 | - | CADM2 |
| 3 | 89531070 | SNV | A | T | 1 | 25178 | Hetero | 17 | 48 | 35.4 | 0.42 | 0 | 70 | - | EPHA3 |
| 3 | 97669754 | Deletion | A | - | 1 | 25178 | Hetero | 143 | 226 | 63.3 | 0.31 | 0 | 176 | - | MINA |
| 3 | 101488794 | SNV | C | A | 1 | 25178 | Hetero | 29 | 82 | 35.4 | 0.20 | 0 | 71 | - | CEP97 |
| 3 | 101541695 | SNV | G | T | 1 | 25178 | Hetero | 13 | 46 | 28.3 | 0.38 | 0 | 53 | - | NXPE3 |
| 3 | 107768545 | SNV | T | C | 1 | 25178 | Hetero | 16 | 45 | 35.6 | 0.43 | 0 | 50 | - | CD47 |
| 3 | 111369610 | SNV | A | G | 1 | 25178 | Hetero | 45 | 106 | 42.5 | 0.42 | 0 | 68 | - | CD96 |
| 3 | 112256682 | SNV | C | A | 1 | 25178 | Hetero | 13 | 48 | 27.1 | 0.21 | 0 | 41 | Yes | ATG3 |
| 3 | 112301913 | SNV | A | G | 1 | 25178 | Hetero | 29 | 88 | 33.0 | 0.49 | 0 | 66 | - | SLC35A5 |
| 3 | 112641999 | SNV | C | T | 1 | 25178 | Hetero | 26 | 61 | 42.6 | 0.50 | 0 | 41 | - | CD200R1 |
| 3 | 114018555 | SNV | T | C | 1 | 25178 | Hetero | 75 | 110 | 68.2 | 0.22 | 0 | 85 | - | TIGIT |
| 3 | 119396230 | SNV | G | A | 1 | 25178 | Hetero | 101 | 184 | 54.9 | 0.40 | 0 | 115 | - | COX17 |
| 3 | 120067936 | SNV | G | A | 1 | 25178 | Hetero | 89 | 137 | 65.0 | 0.36 | 0 | 97 | Yes | LRRC58 |
| 3 | 121346550 | Deletion | T | - | 1 | 25178 | Hetero | 60 | 88 | 68.2 | 0.44 | 0 | 69 | - | FBXO40 |
| 3 | 122144392 | SNV | G | A | 1 | 25178 | Hetero | 32 | 114 | 28.1 | 0.35 | 0 | 98 | - | KPNA1 |
| 3 | 123166747 | SNV | A | G | 1 | 25178 | Hetero | 202 | 342 | 59.1 | 0.36 | 0 | 239 | Yes | ADCY5 |
| 3 | 130673909 | SNV | G | A | 1 | 25178 | Hetero | 14 | 85 | 16.5 | 0.20 | 0 | 45 | Yes | ATP2C1 |
| 3 | 132948094 | SNV | T | G | 1 | 25178 | Hetero | 133 | 367 | 36.2 | 0.25 | 0 | 313 | - | TMEM108 |
| 3 | 137807367 | SNV | T | C | 1 | 25178 | Hetero | 21 | 41 | 51.2 | 0.23 | 0 | 55 | - | DZIP1L |
| 3 | 138763024 | SNV | C | A | 1 | 25178 | Hetero | 173 | 463 | 37.4 | 0.43 | 0 | 340 | Yes | MRPS22 |
| 3 | 139279241 | SNV | C | T | 1 | 25178 | Hetero | 49 | 94 | 52.1 | 0.48 | 0 | 79 | - | RP11-319G6.1 |
| 3 | 148916356 | SNV | G | A | 1 | 25178 | Hetero | 82 | 137 | 59.9 | 0.32 | 0 | 105 | Yes | CP |
| 3 | 150883661 | SNV | G | A | 1 | 25178 | Hetero | 164 | 262 | 62.6 | 0.44 | 0 | 213 | No | MED12L |
| 3 | 157920941 | SNV | G | A | 1 | 25178 | Hetero | 75 | 204 | 36.8 | 0.47 | 0 | 122 | Yes | RSRC1 |
| 3 | 164906524 | SNV | T | A | 1 | 25178 | Hetero | 69 | 195 | 35.4 | 0.49 | 0 | 155 | Yes | SLITRK3 |
| 3 | 172349356 | SNV | G | A | 1 | 25178 | Hetero | 26 | 130 | 20.0 | 0.33 | 0 | 93 | - | NCEH1 |
| 3 | 179119015 | SNV | T | G | 1 | 25178 | Hetero | 102 | 575 | 17.7 | 0.39 | 0 | 518 | No | GNB4 |
| 3 | 187089128 | SNV | A | G | 1 | 25178 | Hetero | 46 | 88 | 52.3 | 0.30 | 0 | 75 | No | RTP4 |
| 3 | 195447737 | SNV | C | A | 1 | 25178 | Hetero | 11 | 95 | 11.6 | 0.31 | 0 | 62 | - | MUC20 |
| 3 | 195593882 | SNV | C | T | 1 | 25178 | Hetero | 127 | 192 | 66.1 | 0.31 | 0 | 136 | No | TNK2 |
| 3 | 196729470 | SNV | C | T | 1 | 25178 | Hetero | 55 | 85 | 64.7 | 0.29 | 0 | 56 | - | MFI2 |
| 3 | 101485482 | Insertion | - | AT | 2 | 25178 | Hetero | 10 | 75 | 13.3 | 0.45 | 0 | 49 | - | CEP97 |
| 3 | 101579643 | Deletion | TA | - | 2 | 25178 | Hetero | 36 | 73 | 49.3 | 0.43 | 0 | 38 | - | NFKBIZ |
| 3 | 10318230 | Insertion | - | T | 1 | 25178 | Hetero | 13 | 52 | 25.0 | 0.20 | 0 | 74 | - | TATDN2 |
| 3 | 111636014 | Insertion | - | A | 1 | 25178 | Hetero | 49 | 136 | 36.0 | 0.30 | 0 | 97 | - | PHLDB2 |
| 3 | 111828384 | Insertion | - | T | 1 | 25178 | Hetero | 140 | 236 | 59.3 | 0.44 | 0 | 165 | - | C3orf52 |
| 3 | 113005743 | Insertion | - | T | 1 | 25178 | Homo | 117 | 129 | 90.7 | 0.39 | 0 | 133 | - | BOC |
| 3 | 124393851 | Deletion | AT | - | 2 | 25178 | Hetero | 4 | 38 | 10.5 | 0.40 | 0 | 41 | - | KALRN |
| 3 | 125248303 | Insertion | - | T | 1 | 25178 | Hetero | 54 | 98 | 55.1 | 0.42 | 0 | 81 | - | OSBPL11 |
| 3 | 156255029 | Insertion | - | A | 1 | 25178 | Hetero | 8 | 59 | 13.6 | 0.30 | 0 | 55 | - | KCNAB1 |
| 3 | 159976432 | Insertion | - | A | 1 | 25178 | Hetero | 36 | 59 | 61.0 | 0.19 | 0 | 86 | - | IFT80 |
| 3 | 160789928 | Insertion | - | T | 1 | 25178 | Hetero | 22 | 77 | 28.6 | 0.36 | 0 | 73 | - | PPM1L |
| 3 | 169713412 | Insertion | - | T | 1 | 25178 | Hetero | 46 | 82 | 56.1 | 0.49 | 0 | 59 | - | SEC62 |
| 3 | 180694863 | Insertion | - | T | 1 | 25178 | Hetero | 4 | 32 | 12.5 | 0.50 | 0 | 32 | - | FXR1 |
| 3 | 42591051 | Insertion | - | AG | 2 | 25178 | Hetero | 93 | 134 | 69.4 | 0.42 | 0 | 212 | - | SEC22C |
| 3 | 47284574 | Insertion | - | A | 1 | 25178 | Hetero | 11 | 35 | 31.4 | 0.38 | 0 | 59 | Insertion | RP11-447D11.2 |
| 3 | 57545169 | Insertion | - | C | 1 | 25178 | Hetero | 53 | 87 | 60.9 | 0.48 | 0 | 110 | - | PDE12 |
| 3 | 69024687 | Insertion | - | TT | 2 | 25178 | Hetero | 13 | 53 | 24.5 | 0.36 | 0 | 69 | - | EOGT |
| 3 | 70014965 | Insertion | - | AA | 2 | 25178 | Hetero | 53 | 70 | 75.7 | 0.33 | 0 | 99 | - | MITF |
| 3 | 97367230 | Insertion | - | A | 1 | 25178 | Homo | 477 | 479 | 99.6 | 0.31 | 0 | 412 | - | EPHA6 |
| 4 | 68185 | Deletion | A | - | 1 | 25178 | Hetero | 50 | 418 | 12.0 | 0.33 | 0 | 380 | - | ZNF595 |
| 4 | 887699 | SNV | C | T | 1 | 25178 | Hetero | 33 | 198 | 16.7 | 0.36 | 0 | 215 | No | GAK |
| 4 | 1065843 | SNV | A | C | 1 | 25178 | Hetero | 12 | 73 | 16.4 | 0.33 | 0 | 89 | - | RNF212 |
| 4 | 1087313 | SNV | T | C | 1 | 25178 | Hetero | 65 | 124 | 52.4 | 0.19 | 0 | 190 | Yes | RNF212 |
| 4 | 1244799 | SNV | G | A | 1 | 25178 | Hetero | 37 | 210 | 17.6 | 0.33 | 0 | 242 | - | CTBP1-AS1 |
| 4 | 1946960 | SNV | G | A | 1 | 25178 | Hetero | 10 | 70 | 14.3 | 0.46 | 0 | 69 | - | WHSC1 |
| 4 | 2271427 | SNV | C | T | 1 | 25178 | Hetero | 10 | 82 | 12.2 | 0.30 | 0 | 100 | - | ZFYVE28 |
| 4 | 3134406 | SNV | T | C | 1 | 25178 | Hetero | 121 | 152 | 79.6 | 0.47 | 0 | 166 | Yes | HTT |
| 4 | 3184070 | SNV | C | T | 1 | 25178 | Hetero | 21 | 119 | 17.6 | 0.45 | 0 | 125 | - | HTT |
| 4 | 3213865 | SNV | G | A | 1 | 25178 | Hetero | 9 | 55 | 16.4 | 0.20 | 0 | 63 | No | HTT |
| 4 | 3235036 | SNV | G | A | 1 | 25178 | Hetero | 56 | 315 | 17.8 | 0.47 | 0 | 368 | No | HTT |
| 4 | 3244896 | SNV | G | T | 1 | 25178 | Hetero | 38 | 207 | 18.4 | 0.29 | 0 | 203 | - | HTT |
| 4 | 3265633 | SNV | G | A | 1 | 25178 | Hetero | 38 | 196 | 19.4 | 0.49 | 0 | 240 | Yes | MSANTD1 |
| 4 | 3319025 | SNV | G | A | 1 | 25178 | Hetero | 79 | 386 | 20.5 | 0.43 | 0 | 436 | No | RGS12 |
| 4 | 3319580 | SNV | C | T | 1 | 25178 | Hetero | 50 | 242 | 20.7 | 0.41 | 0 | 232 | No | RGS12 |
| 4 | 3429844 | SNV | C | T | 1 | 25178 | Hetero | 44 | 195 | 22.6 | 0.39 | 0 | 201 | Yes | RGS12 |
| 4 | 3432508 | SNV | G | A | 1 | 25178 | Hetero | 20 | 136 | 14.7 | 0.32 | 0 | 157 | Yes | RGS12 |
| 4 | 3449758 | SNV | T | C | 1 | 25178 | Hetero | 14 | 72 | 19.4 | 0.43 | 0 | 65 | No | HGFAC |
| 4 | 3495119 | SNV | C | A | 1 | 25178 | Hetero | 27 | 141 | 19.1 | 0.50 | 0 | 122 | Yes | DOK7 |
| 4 | 3495473 | SNV | A | C | 1 | 25178 | Hetero | 31 | 161 | 19.3 | 0.28 | 0 | 191 | - | DOK7 |
| 4 | 3509215 | SNV | T | C | 1 | 25178 | Hetero | 12 | 74 | 16.2 | 0.33 | 0 | 74 | - | LRPAP1 |
| 4 | 3509599 | SNV | G | C | 1 | 25178 | Hetero | 18 | 112 | 16.1 | 0.11 | 0 | 111 | - | LRPAP1 |
| 4 | 3520638 | SNV | G | T | 1 | 25178 | Hetero | 10 | 62 | 16.1 | 0.23 | 0 | 59 | Yes | LRPAP1 |
| 4 | 3590892 | SNV | A | G | 1 | 25178 | Hetero | 80 | 165 | 48.5 | 0.28 | 0 | 175 | Yes | RP3-368B9.1 |
| 4 | 6606997 | SNV | C | T | 1 | 25178 | Hetero | 18 | 99 | 18.2 | 0.25 | 0 | 125 | No | MAN2B2 |
| 4 | 6619574 | SNV | G | T | 1 | 25178 | Hetero | 4 | 31 | 12.9 | 0.40 | 0 | 40 | - | MAN2B2 |
| 4 | 8453741 | SNV | G | A | 1 | 25178 | Hetero | 27 | 125 | 21.6 | 0.33 | 0 | 137 | Yes | TRMT44 |
| 4 | 10441551 | SNV | C | T | 1 | 25178 | Hetero | 8 | 44 | 18.2 | 0.25 | 0 | 39 | - | ZNF518B |
| 4 | 10488255 | SNV | C | G | 1 | 25178 | Hetero | 39 | 54 | 72.2 | 0.41 | 0 | 37 | - | CLNK |
| 4 | 10542162 | SNV | A | G | 1 | 25178 | Hetero | 134 | 164 | 81.7 | 0.35 | 0 | 169 | No | CLNK |
| 4 | 13621686 | SNV | C | G | 1 | 25178 | Hetero | 17 | 78 | 21.8 | 0.36 | 0 | 67 | No | BOD1L1 |
| 4 | 17488614 | SNV | G | A | 1 | 25178 | Hetero | 16 | 65 | 24.6 | 0.38 | 0 | 99 | - | QDPR |
| 4 | 17847182 | Deletion | A | - | 1 | 25178 | Hetero | 21 | 96 | 21.9 | 0.17 | 0 | 88 | - | LCORL |
| 4 | 23803901 | SNV | C | T | 1 | 25178 | Hetero | 186 | 308 | 60.4 | 0.48 | 0 | 387 | Yes | PPARGC1A |
| 4 | 25316917 | SNV | A | G | 1 | 25178 | Hetero | 24 | 132 | 18.2 | 0.18 | 0 | 150 | - | ZCCHC4 |
| 4 | 25673284 | SNV | C | T | 1 | 25178 | Hetero | 19 | 106 | 17.9 | 0.33 | 0 | 145 | Yes | SLC34A2 |
| 4 | 25779422 | SNV | A | G | 1 | 25178 | Hetero | 143 | 186 | 76.9 | 0.42 | 0 | 169 | Yes | SEL1L3 |
| 4 | 25929957 | SNV | G | A | 1 | 25178 | Hetero | 33 | 169 | 19.5 | 0.25 | 0 | 187 | No | C4orf52 |
| 4 | 26426368 | SNV | A | G | 1 | 25178 | Hetero | 31 | 62 | 50.0 | 0.39 | 0 | 58 | - | RBPJ |
| 4 | 26640353 | SNV | A | C | 1 | 25178 | Hetero | 49 | 58 | 84.5 | 0.22 | 0 | 63 | - | TBC1D19 |
| 4 | 38800362 | SNV | T | C | 1 | 25178 | Hetero | 69 | 83 | 83.1 | 0.14 | 0 | 90 | Yes | TLR1 |
| 4 | 39449062 | SNV | T | C | 1 | 25178 | Hetero | 19 | 89 | 21.3 | 0.15 | 0 | 85 | Yes | KLB |
| 4 | 41956492 | SNV | A | G | 1 | 25178 | Hetero | 11 | 83 | 13.3 | 0.25 | 0 | 86 | - | TMEM33 |
| 4 | 42411483 | SNV | C | G | 1 | 25178 | Hetero | 43 | 50 | 86.0 | 0.47 | 0 | 66 | - | ATP8A1 |
| 4 | 57194514 | SNV | A | G | 1 | 25178 | Hetero | 61 | 293 | 20.8 | 0.42 | 0 | 318 | - | KIAA1211 |
| 4 | 57344779 | SNV | A | G | 1 | 25178 | Hetero | 40 | 47 | 85.1 | 0.14 | 0 | 54 | No | SRP72 |
| 4 | 68484520 | SNV | C | A | 1 | 25178 | Hetero | 105 | 126 | 83.3 | 0.46 | 0 | 97 | - | UBA6 |
| 4 | 69362690 | SNV | A | G | 1 | 25178 | Hetero | 86 | 102 | 84.3 | 0.23 | 0 | 121 | - | TMPRSS11E |
| 4 | 69363119 | SNV | A | G | 1 | 25178 | Hetero | 117 | 141 | 83.0 | 0.46 | 0 | 146 | - | TMPRSS11E |
| 4 | 71200982 | SNV | A | C | 1 | 25178 | Hetero | 79 | 108 | 73.1 | 0.40 | 0 | 182 | Yes | CABS1 |
| 4 | 71348449 | SNV | A | G | 1 | 25178 | Hetero | 59 | 72 | 81.9 | 0.29 | 0 | 66 | - | MUC7 |
| 4 | 76882438 | SNV | G | A | 1 | 25178 | Hetero | 62 | 81 | 76.5 | 0.47 | 0 | 75 | Yes | SDAD1 |
| 4 | 76956039 | SNV | G | A | 1 | 25178 | Hetero | 29 | 171 | 17.0 | 0.41 | 0 | 183 | - | CXCL11 |
| 4 | 77272886 | SNV | G | T | 1 | 25178 | Hetero | 7 | 34 | 20.6 | 0.33 | 0 | 41 | Yes | CCDC158 |
| 4 | 77817701 | SNV | C | T | 1 | 25178 | Hetero | 45 | 282 | 16.0 | 0.46 | 0 | 347 | No | SOWAHB |
| 4 | 78804474 | SNV | A | G | 1 | 25178 | Hetero | 27 | 38 | 71.1 | 0.23 | 0 | 53 | No | MRPL1 |
| 4 | 80824527 | SNV | A | T | 1 | 25178 | Hetero | 23 | 31 | 74.2 | 0.25 | 0 | 53 | - | ANTXR2 |
| 4 | 82010858 | SNV | T | C | 1 | 25178 | Hetero | 268 | 321 | 83.5 | 0.50 | 0 | 348 | - | PRKG2 |
| 4 | 82377288 | SNV | A | G | 1 | 25178 | Hetero | 13 | 94 | 13.8 | 0.46 | 0 | 121 | - | RASGEF1B |
| 4 | 82377648 | SNV | C | T | 1 | 25178 | Hetero | 221 | 257 | 86.0 | 0.40 | 0 | 254 | Yes | RASGEF1B |
| 4 | 85781796 | SNV | A | G | 1 | 25178 | Hetero | 31 | 139 | 22.3 | 0.31 | 0 | 165 | - | WDFY3 |
| 4 | 89300206 | SNV | C | T | 1 | 25178 | Hetero | 24 | 154 | 15.6 | 0.43 | 0 | 165 | Yes | HERC6 |
| 4 | 91321311 | SNV | C | G | 1 | 25178 | Hetero | 80 | 120 | 66.7 | 0.47 | 0 | 144 | - | FAM190A |
| 4 | 95539278 | SNV | C | T | 1 | 25178 | Hetero | 100 | 123 | 81.3 | 0.28 | 0 | 121 | No | PDLIM5 |
| 4 | 99802301 | Deletion | A | - | 1 | 25178 | Hetero | 5 | 47 | 10.6 | 0.33 | 0 | 41 | - | EIF4E |
| 4 | 101108117 | SNV | C | T | 1 | 25178 | Homo | 47 | 50 | 94.0 | 0.43 | 0 | 54 | - | DDIT4L |
| 4 | 106158541 | SNV | G | A | 1 | 25178 | Hetero | 22 | 41 | 53.7 | 0.41 | 0 | 61 | Yes | TET2 |
| 4 | 111543987 | SNV | G | T | 1 | 25178 | Hetero | 104 | 126 | 82.5 | 0.46 | 0 | 143 | - | PITX2 |
| 4 | 114269496 | SNV | C | T | 1 | 25178 | Hetero | 83 | 114 | 72.8 | 0.41 | 0 | 159 | - | ANK2 |
| 4 | 114303866 | SNV | C | T | 1 | 25178 | Hetero | 14 | 70 | 20.0 | 0.37 | 0 | 105 | - | ANK2 |
| 4 | 114458432 | SNV | G | A | 1 | 25178 | Hetero | 5 | 46 | 10.9 | 0.43 | 0 | 48 | - | CAMK2D |
| 4 | 119476395 | SNV | T | G | 1 | 25178 | Hetero | 184 | 239 | 77.0 | 0.26 | 0 | 314 | - | CEP170P1 |
| 4 | 119979129 | Deletion | A | - | 1 | 25178 | Hetero | 268 | 338 | 79.3 | 0.28 | 0 | 362 | - | SYNPO2 |
| 4 | 120982202 | SNV | G | A | 1 | 25178 | Homo | 46 | 50 | 92.0 | 0.33 | 0 | 36 | - | MAD2L1 |
| 4 | 128565127 | SNV | G | A | 1 | 25178 | Hetero | 173 | 216 | 80.1 | 0.37 | 0 | 233 | Yes | INTU |
| 4 | 134071430 | SNV | T | G | 1 | 25178 | Hetero | 107 | 131 | 81.7 | 0.41 | 0 | 185 | Yes | PCDH10 |
| 4 | 149002017 | Deletion | T | - | 1 | 25178 | Homo | 122 | 124 | 98.4 | 0.16 | 0 | 114 | - | NR3C2 |
| 4 | 153539899 | SNV | T | C | 1 | 25178 | Hetero | 89 | 115 | 77.4 | 0.45 | 0 | 82 | - | TMEM154 |
| 4 | 153899995 | SNV | T | C | 1 | 25178 | Hetero | 97 | 118 | 82.2 | 0.47 | 0 | 85 | - | FHDC1 |
| 4 | 156137893 | SNV | A | C | 1 | 25178 | Hetero | 12 | 77 | 15.6 | 0.31 | 0 | 94 | - | NPY2R |
| 4 | 159175398 | SNV | A | G | 1 | 25178 | Hetero | 22 | 108 | 20.4 | 0.42 | 0 | 111 | - | TMEM144 |
| 4 | 164448333 | SNV | C | T | 1 | 25178 | Hetero | 19 | 85 | 22.4 | 0.42 | 0 | 105 | - | MARCH1 |
| 4 | 164450083 | SNV | C | T | 1 | 25178 | Hetero | 36 | 193 | 18.7 | 0.49 | 0 | 239 | No | MARCH1 |
| 4 | 169142919 | SNV | G | A | 1 | 25178 | Hetero | 23 | 176 | 13.1 | 0.38 | 0 | 151 | No | DDX60 |
| 4 | 170926938 | SNV | C | T | 1 | 25178 | Hetero | 145 | 180 | 80.6 | 0.48 | 0 | 215 | Yes | MFAP3L |
| 4 | 172735851 | SNV | G | A | 1 | 25178 | Hetero | 73 | 90 | 81.1 | 0.20 | 0 | 99 | No | GALNTL6 |
| 4 | 173930379 | SNV | C | T | 1 | 25178 | Hetero | 21 | 114 | 18.4 | 0.37 | 0 | 116 | No | GALNTL6 |
| 4 | 175254364 | SNV | T | C | 1 | 25178 | Hetero | 22 | 89 | 24.7 | 0.38 | 0 | 91 | - | CEP44 |
| 4 | 184367749 | SNV | A | G | 1 | 25178 | Hetero | 297 | 356 | 83.4 | 0.42 | 0 | 393 | No | CDKN2AIP |
| 4 | 184626187 | SNV | G | A | 1 | 25178 | Hetero | 139 | 170 | 81.8 | 0.46 | 0 | 136 | Yes | TRAPPC11 |
| 4 | 186082941 | SNV | G | T | 1 | 25178 | Hetero | 66 | 84 | 78.6 | 0.32 | 0 | 96 | - | KIAA1430 |
| 4 | 187541209 | SNV | T | A | 1 | 25178 | Hetero | 28 | 180 | 15.6 | 0.37 | 0 | 209 | Yes | FAT1 |
| 4 | 189020256 | SNV | C | T | 1 | 25178 | Hetero | 84 | 103 | 81.6 | 0.27 | 0 | 99 | Yes | TRIML2 |
| 4 | 10441912 | Insertion | - | T | 1 | 25178 | Hetero | 11 | 85 | 12.9 | 0.42 | 0 | 102 | - | ZNF518B |
| 4 | 105391339 | Insertion | - | C | 1 | 25178 | Homo | 38 | 45 | 84.4 | 0.34 | 0 | 49 | - | CXXC4 |
| 4 | 114117474 | Insertion | - | T | 1 | 25178 | Hetero | 26 | 130 | 20.0 | 0.23 | 0 | 138 | - | ANK2 |
| 4 | 119256617 | Insertion | - | T | 1 | 25178 | Hetero | 26 | 207 | 12.6 | 0.21 | 0 | 207 | - | PRSS12 |
| 4 | 119979267 | Deletion | AC | - | 2 | 25178 | Hetero | 56 | 68 | 82.4 | 0.44 | 0 | 85 | - | SYNPO2 |
| 4 | 128812230 | Insertion | - | T | 1 | 25178 | Hetero | 20 | 52 | 38.5 | 0.27 | 0 | 47 | - | PLK4 |
| 4 | 153247290 | Deletion | GA | - | 2 | 25178 | Hetero | 152 | 253 | 60.1 | 0.49 | 0 | 305 | Deletion | FBXW7 |
| 4 | 175558532 | Insertion | - | CC | 2 | 25178 | Hetero | 33 | 170 | 19.4 | 0.42 | 0 | 210 | - | GLRA3 |
| 4 | 175562145 | Insertion | - | TA | 2 | 25178 | Hetero | 30 | 46 | 65.2 | 0.31 | 0 | 66 | - | GLRA3 |
| 4 | 3495798 | Insertion | - | G | 1 | 25178 | Hetero | 46 | 223 | 20.6 | 0.49 | 0 | 239 | - | DOK7 |
| 4 | 71658113 | Insertion | - | AC | 2 | 25178 | Hetero | 72 | 94 | 76.6 | 0.42 | 0 | 70 | - | RUFY3 |
| 4 | 761102 | Insertion | - | T | 1 | 25178 | Hetero | 15 | 93 | 16.1 | 0.39 | 0 | 95 | - | PCGF3 |
| 4 | 762263 | Deletion | TT | - | 2 | 25178 | Homo | 92 | 92 | 100.0 | 0.32 | 0 | 84 | - | PCGF3 |
| 4 | 88059180 | Insertion | - | TT | 2 | 25178 | Hetero | 11 | 86 | 12.8 | 0.45 | 0 | 110 | - | AFF1 |
| 4 | 88084360 | Insertion | - | AG | 2 | 25178 | Hetero | 18 | 151 | 11.9 | 0.29 | 0 | 128 | - | KLHL8 |
| 4 | 89911840 | Insertion | - | T | 1 | 25178 | Homo | 54 | 54 | 100.0 | 0.35 | 0 | 50 | - | FAM13A |
| 4 | 96169363 | Insertion | - | T | 1 | 25178 | Hetero | 75 | 95 | 78.9 | 0.35 | 0 | 102 | - | UNC5C |
| 5 | 437494 | SNV | G | A | 1 | 25178 | Hetero | 75 | 136 | 55.1 | 0.43 | 0 | 120 | - | AHRR |
| 5 | 442658 | SNV | C | T | 1 | 25178 | Hetero | 94 | 182 | 51.6 | 0.27 | 0 | 220 | Yes | C5orf55 |
| 5 | 839962 | SNV | G | A | 1 | 25178 | Hetero | 32 | 78 | 41.0 | 0.34 | 0 | 180 | - | ZDHHC11 |
| 5 | 848742 | SNV | C | T | 1 | 25178 | Hetero | 45 | 116 | 38.8 | 0.41 | 0 | 170 | Yes | ZDHHC11 |
| 5 | 1037670 | SNV | A | G | 1 | 25178 | Hetero | 87 | 133 | 65.4 | 0.35 | 0 | 121 | Yes | NKD2 |
| 5 | 2751790 | SNV | G | A | 1 | 25178 | Hetero | 88 | 151 | 58.3 | 0.47 | 0 | 129 | - | IRX2 |
| 5 | 7829433 | SNV | A | G | 1 | 25178 | Hetero | 36 | 107 | 33.6 | 0.45 | 0 | 115 | - | ADCY2 |
| 5 | 9039351 | SNV | C | G | 1 | 25178 | Hetero | 23 | 140 | 16.4 | 0.44 | 0 | 205 | - | CTD-2215L10.1 |
| 5 | 10461381 | SNV | T | C | 1 | 25178 | Hetero | 198 | 322 | 61.5 | 0.43 | 0 | 337 | Yes | ROPN1L |
| 5 | 34818977 | Deletion | T | - | 1 | 25178 | Hetero | 57 | 88 | 64.8 | 0.37 | 0 | 58 | - | RAI14 |
| 5 | 34913675 | SNV | T | C | 1 | 25178 | Hetero | 11 | 85 | 12.9 | 0.43 | 0 | 53 | Yes | RAD1 |
| 5 | 36143531 | SNV | A | C | 1 | 25178 | Hetero | 84 | 246 | 34.1 | 0.20 | 0 | 233 | - | LMBRD2 |
| 5 | 36249108 | SNV | G | A | 1 | 25178 | Hetero | 34 | 50 | 68.0 | 0.39 | 0 | 53 | - | RANBP3L |
| 5 | 37169027 | SNV | T | C | 1 | 25178 | Hetero | 118 | 340 | 34.7 | 0.45 | 0 | 335 | Yes | C5orf42 |
| 5 | 37179596 | SNV | A | G | 1 | 25178 | Hetero | 17 | 56 | 30.4 | 0.06 | 0 | 41 | - | C5orf42 |
| 5 | 41310388 | SNV | A | T | 1 | 25178 | Hetero | 35 | 85 | 41.2 | 0.42 | 0 | 71 | - | PLCXD3 |
| 5 | 58999615 | SNV | C | G | 1 | 25178 | Hetero | 44 | 82 | 53.7 | 0.22 | 0 | 159 | - | PDE4D |
| 5 | 67595319 | SNV | C | T | 1 | 25178 | Hetero | 22 | 72 | 30.6 | 0.41 | 0 | 84 | - | PIK3R1 |
| 5 | 68578803 | SNV | C | G | 1 | 25178 | Hetero | 47 | 100 | 47.0 | 0.25 | 0 | 112 | Yes | CCDC125 |
| 5 | 72385177 | SNV | T | A | 1 | 25178 | Hetero | 63 | 104 | 60.6 | 0.43 | 0 | 90 | - | FCHO2 |
| 5 | 73931719 | SNV | A | G | 1 | 25178 | Hetero | 27 | 118 | 22.9 | 0.47 | 0 | 130 | Yes | ENC1 |
| 5 | 74806943 | SNV | G | A | 1 | 25178 | Hetero | 45 | 95 | 47.4 | 0.34 | 0 | 118 | No | COL4A3BP |
| 5 | 74988273 | SNV | A | C | 1 | 25178 | Hetero | 33 | 131 | 25.2 | 0.45 | 0 | 180 | Yes | POC5 |
| 5 | 76115145 | SNV | C | T | 1 | 25178 | Hetero | 26 | 54 | 48.1 | 0.19 | 0 | 69 | - | F2RL1 |
| 5 | 77782100 | SNV | G | A | 1 | 25178 | Hetero | 41 | 82 | 50.0 | 0.50 | 0 | 115 | - | LHFPL2 |
| 5 | 77782699 | SNV | A | G | 1 | 25178 | Hetero | 21 | 42 | 50.0 | 0.43 | 0 | 63 | - | LHFPL2 |
| 5 | 82937382 | SNV | C | T | 1 | 25178 | Hetero | 46 | 209 | 22.0 | 0.35 | 0 | 316 | Yes | HAPLN1 |
| 5 | 94042147 | SNV | T | G | 1 | 25178 | Hetero | 36 | 86 | 41.9 | 0.45 | 0 | 122 | - | ANKRD32 |
| 5 | 95727879 | Deletion | T | - | 1 | 25178 | Hetero | 63 | 77 | 81.8 | 0.42 | 0 | 59 | - | PCSK1 |
| 5 | 108714583 | SNV | A | G | 1 | 25178 | Hetero | 117 | 250 | 46.8 | 0.43 | 0 | 343 | Yes | PJA2 |
| 5 | 112824284 | SNV | T | C | 1 | 25178 | Hetero | 74 | 154 | 48.1 | 0.47 | 0 | 210 | - | MCC |
| 5 | 112824448 | SNV | C | T | 1 | 25178 | Hetero | 79 | 142 | 55.6 | 0.41 | 0 | 186 | - | MCC |
| 5 | 117896670 | Deletion | T | - | 1 | 25178 | Hetero | 24 | 48 | 50.0 | 0.35 | 0 | 47 | - | CTD-2281M20.1 |
| 5 | 122515985 | SNV | C | T | 1 | 25178 | Hetero | 19 | 109 | 17.4 | 0.48 | 0 | 160 | No | PRDM6 |
| 5 | 126205564 | SNV | A | C | 1 | 25178 | Hetero | 29 | 61 | 47.5 | 0.45 | 0 | 81 | - | MARCH3 |
| 5 | 134294883 | SNV | T | C | 1 | 25178 | Hetero | 51 | 85 | 60.0 | 0.11 | 0 | 85 | - | PCBD2 |
| 5 | 137684604 | SNV | T | G | 1 | 25178 | Hetero | 24 | 70 | 34.3 | 0.42 | 0 | 59 | - | FAM53C |
| 5 | 138667182 | SNV | A | G | 1 | 25178 | Hetero | 30 | 48 | 62.5 | 0.30 | 0 | 55 | - | MATR3 |
| 5 | 139912380 | SNV | C | T | 1 | 25178 | Hetero | 11 | 52 | 21.2 | 0.47 | 0 | 48 | - | ANKHD1 |
| 5 | 140201442 | SNV | G | T | 1 | 25178 | Hetero | 161 | 294 | 54.8 | 0.21 | 0 | 254 | Yes | PCDHA1 |
| 5 | 140553273 | SNV | A | T | 1 | 25178 | Hetero | 103 | 299 | 34.4 | 0.41 | 0 | 313 | Yes | PCDHB7 |
| 5 | 141324705 | SNV | A | G | 1 | 25178 | Hetero | 63 | 179 | 35.2 | 0.43 | 0 | 198 | - | PCDH12 |
| 5 | 141692497 | SNV | T | A | 1 | 25178 | Hetero | 49 | 107 | 45.8 | 0.44 | 0 | 133 | - | SPRY4 |
| 5 | 142273847 | SNV | A | G | 1 | 25178 | Hetero | 60 | 277 | 21.7 | 0.48 | 0 | 329 | No | ARHGAP26 |
| 5 | 145317740 | SNV | G | C | 1 | 25178 | Hetero | 60 | 112 | 53.6 | 0.47 | 0 | 151 | No | SH3RF2 |
| 5 | 146771948 | SNV | C | T | 1 | 25178 | Hetero | 24 | 52 | 46.2 | 0.30 | 0 | 76 | - | DPYSL3 |
| 5 | 149219550 | SNV | C | T | 1 | 25178 | Hetero | 91 | 175 | 52.0 | 0.35 | 0 | 216 | - | PPARGC1B |
| 5 | 149231423 | SNV | G | A | 1 | 25178 | Hetero | 22 | 56 | 39.3 | 0.48 | 0 | 94 | - | PPARGC1B |
| 5 | 149569900 | SNV | C | T | 1 | 25178 | Hetero | 15 | 52 | 28.8 | 0.40 | 0 | 73 | Yes | SLC6A7 |
| 5 | 150029146 | SNV | C | T | 1 | 25178 | Hetero | 58 | 115 | 50.4 | 0.36 | 0 | 171 | Yes | SYNPO |
| 5 | 150444591 | SNV | G | A | 1 | 25178 | Hetero | 53 | 116 | 45.7 | 0.44 | 0 | 117 | No | TNIP1 |
| 5 | 151191135 | SNV | T | C | 1 | 25178 | Hetero | 44 | 86 | 51.2 | 0.49 | 0 | 105 | - | G3BP1 |
| 5 | 153191420 | Deletion | G | - | 1 | 25178 | Hetero | 59 | 109 | 54.1 | 0.39 | 0 | 162 | - | GRIA1 |
| 5 | 154135719 | SNV | G | T | 1 | 25178 | Hetero | 51 | 104 | 49.0 | 0.43 | 0 | 118 | No | LARP1 |
| 5 | 171636675 | SNV | A | T | 1 | 25178 | Hetero | 42 | 104 | 40.4 | 0.46 | 0 | 125 | - | UBTD2 |
| 5 | 172113745 | SNV | G | A | 1 | 25178 | Hetero | 140 | 256 | 54.7 | 0.49 | 0 | 299 | No | NEURL1B |
| 5 | 175387011 | SNV | G | A | 1 | 25178 | Hetero | 27 | 189 | 14.3 | 0.38 | 0 | 489 | No | THOC3 |
| 5 | 176918142 | SNV | G | A | 1 | 25178 | Hetero | 37 | 87 | 42.5 | 0.36 | 0 | 75 | Yes | PDLIM7 |
| 5 | 177059586 | SNV | G | A | 1 | 25178 | Hetero | 35 | 94 | 37.2 | 0.42 | 0 | 162 | - | RP11-1277A3.2 |
| 5 | 177163623 | Deletion | A | - | 1 | 25178 | Hetero | 33 | 44 | 75.0 | 0.25 | 0 | 127 | - | FAM153A |
| 5 | 177309736 | Deletion | T | - | 1 | 25178 | Hetero | 41 | 121 | 33.9 | 0.22 | 0 | 382 | - | RP11-423H2.1 |
| 5 | 177310792 | SNV | C | T | 1 | 25178 | Hetero | 39 | 135 | 28.9 | 0.38 | 0 | 390 | - | RP11-423H2.1 |
| 5 | 177549358 | Deletion | G | - | 1 | 25178 | Hetero | 28 | 70 | 40.0 | 0.45 | 0 | 93 | - | N4BP3 |
| 5 | 180480933 | Deletion | A | - | 1 | 25178 | Hetero | 118 | 287 | 41.1 | 0.47 | 0 | 369 | - | BTNL9 |
| 5 | 106714203 | Insertion | - | T | 1 | 25178 | Hetero | 34 | 87 | 39.1 | 0.30 | 0 | 117 | - | EFNA5 |
| 5 | 108673121 | Insertion | - | A | 1 | 25178 | Hetero | 16 | 41 | 39.0 | 0.06 | 0 | 67 | - | PJA2 |
| 5 | 112212524 | Insertion | - | AA | 2 | 25178 | Hetero | 29 | 85 | 34.1 | 0.30 | 0 | 147 | - | SRP19 |
| 5 | 112353002 | Deletion | TG | - | 2 | 25178 | Hetero | 33 | 60 | 55.0 | 0.44 | 0 | 63 | - | DCP2 |
| 5 | 122680580 | Insertion | - | A | 1 | 25178 | Homo | 34 | 34 | 100.0 | 0.34 | 0 | 43 | - | CEP120 |
| 5 | 137513369 | Insertion | - | AA | 2 | 25178 | Hetero | 21 | 136 | 15.4 | 0.17 | 0 | 108 | - | BRD8 |
| 5 | 140940449 | Insertion | - | T | 1 | 25178 | Hetero | 24 | 69 | 34.8 | 0.46 | 0 | 71 | - | DIAPH1 |
| 5 | 147504305 | Insertion | - | T | 1 | 25178 | Homo | 34 | 34 | 100.0 | 0.09 | 0 | 54 | - | SPINK5 |
| 5 | 149283196 | Insertion | - | A | 1 | 25178 | Hetero | 47 | 91 | 51.6 | 0.23 | 0 | 128 | - | PDE6A |
| 5 | 36608727 | Insertion | - | A | 1 | 25178 | Hetero | 47 | 151 | 31.1 | 0.27 | 0 | 127 | - | SLC1A3 |
| 5 | 5319489 | Insertion | - | A | 1 | 25178 | Hetero | 33 | 82 | 40.2 | 0.41 | 0 | 91 | - | ADAMTS16 |
| 5 | 664205 | Insertion | - | G | 1 | 25178 | Hetero | 50 | 122 | 41.0 | 0.37 | 0 | 159 | - | CEP72 |
| 5 | 74072339 | Insertion | - | A | 1 | 25178 | Hetero | 18 | 30 | 60.0 | 0.38 | 0 | 43 | - | NSA2 |
| 5 | 76760657 | Insertion | - | A | 1 | 25178 | Hetero | 58 | 85 | 68.2 | 0.29 | 0 | 144 | - | WDR41 |
| 6 | 2838893 | SNV | C | A | 1 | 25178 | Hetero | 25 | 43 | 58.1 | 0.17 | 0 | 33 | Yes | SERPINB1 |
| 6 | 5085859 | SNV | C | T | 1 | 25178 | Hetero | 43 | 96 | 44.8 | 0.39 | 0 | 129 | Yes | PPP1R3G |
| 6 | 7268884 | SNV | A | G | 1 | 25178 | Hetero | 90 | 221 | 40.7 | 0.47 | 0 | 247 | - | SSR1 |
| 6 | 9795601 | SNV | A | G | 1 | 25178 | Hetero | 6 | 42 | 14.3 | 0.17 | 0 | 43 | - | OFCC1 |
| 6 | 9795607 | SNV | A | G | 1 | 25178 | Hetero | 6 | 50 | 12.0 | 0.17 | 0 | 47 | - | OFCC1 |
| 6 | 10415487 | Deletion | C | - | 1 | 25178 | Hetero | 41 | 88 | 46.6 | 0.37 | 0 | 100 | - | TFAP2A |
| 6 | 13711758 | SNV | C | A | 1 | 25178 | Hetero | 75 | 156 | 48.1 | 0.44 | 0 | 182 | - | RANBP9 |
| 6 | 17809209 | SNV | A | G | 1 | 25178 | Hetero | 12 | 43 | 27.9 | 0.27 | 0 | 70 | - | KIF13A |
| 6 | 24495271 | SNV | G | A | 1 | 25178 | Hetero | 13 | 49 | 26.5 | 0.20 | 0 | 67 | Yes | GPLD1 |
| 6 | 26217245 | SNV | G | C | 1 | 25178 | Hetero | 89 | 358 | 24.9 | 0.50 | 0 | 424 | Yes | HIST1H2AE |
| 6 | 28093339 | SNV | T | A | 1 | 25178 | Hetero | 125 | 245 | 51.0 | 0.45 | 0 | 286 | Yes | ZSCAN16 |
| 6 | 32790100 | Deletion | A | - | 1 | 25178 | Hetero | 39 | 97 | 40.2 | 0.05 | 0 | 114 | - | TAP2 |
| 6 | 36355505 | SNV | T | A | 1 | 25178 | Hetero | 65 | 167 | 38.9 | 0.43 | 0 | 183 | - | ETV7 |
| 6 | 36695505 | SNV | C | T | 1 | 25178 | Hetero | 45 | 68 | 66.2 | 0.32 | 0 | 78 | - | PI16 |
| 6 | 42131049 | SNV | G | A | 1 | 25178 | Hetero | 44 | 97 | 45.4 | 0.11 | 0 | 127 | - | GUCA1A |
| 6 | 42237513 | SNV | C | T | 1 | 25178 | Hetero | 23 | 58 | 39.7 | 0.44 | 0 | 39 | - | TRERF1 |
| 6 | 42650807 | SNV | G | A | 1 | 25178 | Hetero | 10 | 43 | 23.3 | 0.25 | 0 | 45 | Yes | UBR2 |
| 6 | 43172156 | SNV | G | C | 1 | 25178 | Hetero | 72 | 158 | 45.6 | 0.38 | 0 | 224 | Yes | CUL9 |
| 6 | 43323109 | SNV | C | T | 1 | 25178 | Hetero | 108 | 225 | 48.0 | 0.48 | 0 | 249 | Yes | ZNF318 |
| 6 | 43473883 | SNV | C | T | 1 | 25178 | Hetero | 32 | 57 | 56.1 | 0.37 | 0 | 46 | - | TJAP1 |
| 6 | 43640274 | SNV | A | T | 1 | 25178 | Hetero | 38 | 79 | 48.1 | 0.48 | 0 | 78 | - | RSPH9 |
| 6 | 43738960 | SNV | G | A | 1 | 25178 | Hetero | 97 | 182 | 53.3 | 0.36 | 0 | 196 | Yes | VEGFA |
| 6 | 44140758 | SNV | T | A | 1 | 25178 | Hetero | 38 | 123 | 30.9 | 0.20 | 0 | 138 | Yes | CAPN11 |
| 6 | 45515641 | SNV | T | G | 1 | 25178 | Homo | 74 | 76 | 97.4 | 0.47 | 0 | 100 | - | RUNX2 |
| 6 | 49399240 | Deletion | T | - | 1 | 25178 | Hetero | 54 | 105 | 51.4 | 0.49 | 0 | 116 | - | MUT |
| 6 | 52318959 | SNV | A | G | 1 | 25178 | Hetero | 9 | 51 | 17.6 | 0.27 | 0 | 74 | Yes | EFHC1 |
| 6 | 52367473 | SNV | C | T | 1 | 25178 | Hetero | 76 | 163 | 46.6 | 0.41 | 0 | 196 | - | EFHC1 |
| 6 | 70993262 | SNV | C | A | 1 | 25178 | Hetero | 26 | 47 | 55.3 | 0.29 | 0 | 75 | - | COL9A1 |
| 6 | 75899494 | SNV | G | T | 1 | 25178 | Hetero | 92 | 227 | 40.5 | 0.32 | 0 | 332 | No | COL12A1 |
| 6 | 91296724 | SNV | G | T | 1 | 25178 | Hetero | 27 | 54 | 50.0 | 0.42 | 0 | 54 | - | MAP3K7 |
| 6 | 102074485 | SNV | G | A | 1 | 25178 | Hetero | 16 | 73 | 21.9 | 0.35 | 0 | 116 | Yes | GRIK2 |
| 6 | 107100334 | SNV | C | T | 1 | 25178 | Hetero | 105 | 200 | 52.5 | 0.23 | 0 | 293 | - | QRSL1 |
| 6 | 108192122 | SNV | C | G | 1 | 25178 | Hetero | 23 | 66 | 34.8 | 0.32 | 0 | 87 | - | SEC63 |
| 6 | 118638091 | SNV | C | T | 1 | 25178 | Hetero | 24 | 50 | 48.0 | 0.44 | 0 | 72 | - | SLC35F1 |
| 6 | 122753600 | Deletion | G | - | 1 | 25178 | Hetero | 27 | 56 | 48.2 | 0.50 | 0 | 75 | - | HSF2 |
| 6 | 125409476 | Deletion | A | - | 1 | 25178 | Hetero | 64 | 122 | 52.5 | 0.47 | 0 | 136 | - | RNF217 |
| 6 | 125614176 | SNV | T | C | 1 | 25178 | Hetero | 21 | 47 | 44.7 | 0.25 | 0 | 48 | - | HDDC2 |
| 6 | 129898179 | Deletion | A | - | 1 | 25178 | Hetero | 40 | 99 | 40.4 | 0.48 | 0 | 88 | - | RP11-73O6.4 |
| 6 | 136871627 | SNV | C | T | 1 | 25178 | Hetero | 37 | 80 | 46.3 | 0.17 | 0 | 65 | - | MAP7 |
| 6 | 138663346 | SNV | C | T | 1 | 25178 | Hetero | 23 | 45 | 51.1 | 0.25 | 0 | 39 | - | KIAA1244 |
| 6 | 146056499 | SNV | C | G | 1 | 25178 | Hetero | 24 | 46 | 52.2 | 0.08 | 0 | 37 | Yes | EPM2A |
| 6 | 147036664 | SNV | C | A | 1 | 25178 | Hetero | 25 | 56 | 44.6 | 0.29 | 0 | 58 | - | ADGB |
| 6 | 151685529 | Deletion | A | - | 1 | 25178 | Hetero | 23 | 60 | 38.3 | 0.38 | 0 | 67 | - | ZBTB2 |
| 6 | 152422515 | Deletion | A | - | 1 | 25178 | Hetero | 24 | 53 | 45.3 | 0.37 | 0 | 59 | - | ESR1 |
| 6 | 154744033 | SNV | C | T | 1 | 25178 | Hetero | 25 | 56 | 44.6 | 0.38 | 0 | 67 | - | CNKSR3 |
| 6 | 155154577 | SNV | C | T | 1 | 25178 | Hetero | 83 | 192 | 43.2 | 0.47 | 0 | 186 | - | SCAF8 |
| 6 | 158928366 | SNV | C | T | 1 | 25178 | Hetero | 41 | 87 | 47.1 | 0.44 | 0 | 102 | - | TULP4 |
| 6 | 158930337 | Deletion | T | - | 1 | 25178 | Hetero | 28 | 44 | 63.6 | 0.23 | 0 | 48 | - | TULP4 |
| 6 | 160969756 | SNV | T | A | 1 | 25178 | Hetero | 18 | 41 | 43.9 | 0.14 | 0 | 61 | - | LPA |
| 6 | 163995998 | SNV | A | C | 1 | 25178 | Hetero | 17 | 69 | 24.6 | 0.48 | 0 | 90 | - | QKI |
| 6 | 167587534 | SNV | C | T | 1 | 25178 | Hetero | 77 | 310 | 24.8 | 0.40 | 0 | 314 | - | TCP10L2 |
| 6 | 167592274 | SNV | A | C | 1 | 25178 | Homo | 76 | 79 | 96.2 | 0.36 | 0 | 128 | - | TCP10L2 |
| 6 | 167754936 | SNV | G | A | 1 | 25178 | Hetero | 149 | 271 | 55.0 | 0.38 | 0 | 309 | No | TTLL2 |
| 6 | 167786424 | SNV | G | T | 1 | 25178 | Hetero | 26 | 83 | 31.3 | 0.46 | 0 | 431 | - | TCP10 |
| 6 | 167786574 | SNV | A | T | 1 | 25178 | Hetero | 134 | 426 | 31.5 | 0.36 | 0 | 702 | - | TCP10 |
| 6 | 167788895 | SNV | G | A | 1 | 25178 | Hetero | 38 | 91 | 41.8 | 0.37 | 0 | 219 | - | TCP10 |
| 6 | 10397893 | Insertion | - | A | 1 | 25178 | Hetero | 32 | 55 | 58.2 | 0.46 | 0 | 53 | - | TFAP2A |
| 6 | 110931624 | Insertion | - | A | 1 | 25178 | Hetero | 17 | 31 | 54.8 | 0.32 | 0 | 50 | - | CDK19 |
| 6 | 116841107 | Insertion | - | G | 1 | 25178 | Homo | 86 | 86 | 100.0 | 0.42 | 0 | 98 | - | BET3L |
| 6 | 116845340 | Insertion | - | AA | 2 | 25178 | Homo | 122 | 122 | 100.0 | 0.49 | 0 | 165 | - | BET3L |
| 6 | 122749156 | Insertion | - | TC | 2 | 25178 | Hetero | 23 | 49 | 46.9 | 0.48 | 0 | 67 | - | HSF2 |
| 6 | 150719822 | Insertion | - | T | 1 | 25178 | Hetero | 21 | 42 | 50.0 | 0.41 | 0 | 49 | - | IYD |
| 6 | 160101978 | Insertion | - | A | 1 | 25178 | Hetero | 15 | 36 | 41.7 | 0.12 | 0 | 37 | - | SOD2 |
| 6 | 26385170 | Insertion | - | T | 1 | 25178 | Hetero | 66 | 137 | 48.2 | 0.13 | 0 | 157 | - | BTN2A2 |
| 6 | 27370925 | Insertion | - | A | 1 | 25178 | Hetero | 32 | 74 | 43.2 | 0.30 | 0 | 78 | - | ZNF391 |
| 6 | 31106500 | Insertion | - | C | 1 | 25178 | Hetero | 206 | 453 | 45.5 | 0.47 | 0 | 504 | Insertion | PSORS1C1 |
| 6 | 31380161 | Insertion | - | CT | 2 | 25178 | Hetero | 34 | 99 | 34.3 | 0.46 | 0 | 88 | Insertion | MICA |
| 6 | 34555666 | Insertion | - | C | 1 | 25178 | Hetero | 55 | 116 | 47.4 | 0.47 | 0 | 126 | - | C6orf106 |
| 6 | 38122149 | Insertion | - | G | 1 | 25178 | Hetero | 30 | 90 | 33.3 | 0.30 | 0 | 114 | - | ZFAND3 |
| 6 | 49398333 | Insertion | - | T | 1 | 25178 | Hetero | 19 | 41 | 46.3 | 0.35 | 0 | 52 | - | MUT |
| 6 | 49415351 | Insertion | - | A | 1 | 25178 | Hetero | 7 | 32 | 21.9 | 0.38 | 0 | 53 | - | MUT |
| 6 | 52129012 | Insertion | - | A | 1 | 25178 | Hetero | 34 | 92 | 37.0 | 0.49 | 0 | 110 | - | MCM3 |
| 6 | 53362447 | Insertion | - | A | 1 | 25178 | Hetero | 40 | 93 | 43.0 | 0.39 | 0 | 112 | - | GCLC |
| 6 | 62390245 | Insertion | - | T | 1 | 25178 | Hetero | 24 | 53 | 45.3 | 0.43 | 0 | 81 | - | KHDRBS2 |
| 6 | 87971652 | Insertion | - | A | 1 | 25178 | Hetero | 13 | 39 | 33.3 | 0.15 | 0 | 49 | - | ZNF292 |
| 7 | 588884 | Deletion | G | - | 1 | 25178 | Hetero | 61 | 117 | 52.1 | 0.33 | 0 | 114 | - | PRKAR1B |
| 7 | 926833 | SNV | G | A | 1 | 25178 | Hetero | 59 | 156 | 37.8 | 0.27 | 0 | 166 | No | SUN1 |
| 7 | 1589969 | SNV | T | C | 1 | 25178 | Hetero | 228 | 384 | 59.4 | 0.46 | 0 | 385 | Yes | TMEM184A |
| 7 | 1976463 | SNV | C | T | 1 | 25178 | Hetero | 78 | 143 | 54.5 | 0.48 | 0 | 170 | Yes | MAD1L1 |
| 7 | 3205657 | SNV | C | T | 1 | 25178 | Hetero | 39 | 69 | 56.5 | 0.17 | 0 | 94 | - | AC091801.1 |
| 7 | 4830068 | SNV | G | A | 1 | 25178 | Hetero | 113 | 254 | 44.5 | 0.50 | 0 | 246 | - | AP5Z1 |
| 7 | 5354648 | SNV | G | A | 1 | 25178 | Hetero | 20 | 49 | 40.8 | 0.24 | 0 | 60 | Yes | TNRC18 |
| 7 | 6202225 | SNV | A | G | 1 | 25178 | Hetero | 107 | 175 | 61.1 | 0.27 | 0 | 197 | - | CYTH3 |
| 7 | 14775824 | SNV | A | C | 1 | 25178 | Hetero | 31 | 55 | 56.4 | 0.06 | 0 | 82 | - | DGKB |
| 7 | 17379774 | SNV | G | T | 1 | 25178 | Hetero | 68 | 145 | 46.9 | 0.40 | 0 | 175 | Yes | AHR |
| 7 | 20180360 | Deletion | A | - | 1 | 25178 | Hetero | 20 | 46 | 43.5 | 0.45 | 0 | 42 | - | MACC1 |
| 7 | 22349762 | Deletion | A | - | 1 | 25178 | Hetero | 51 | 156 | 32.7 | 0.19 | 0 | 121 | - | RAPGEF5 |
| 7 | 27282862 | SNV | T | C | 1 | 25178 | Hetero | 57 | 134 | 42.5 | 0.38 | 0 | 130 | No | EVX1 |
| 7 | 27871845 | SNV | G | T | 1 | 25178 | Hetero | 31 | 46 | 67.4 | 0.34 | 0 | 32 | - | TAX1BP1 |
| 7 | 29605839 | SNV | T | C | 1 | 25178 | Hetero | 28 | 82 | 34.1 | 0.40 | 0 | 113 | - | PRR15 |
| 7 | 32626084 | SNV | C | A | 1 | 25178 | Hetero | 5 | 32 | 15.6 | 0.40 | 0 | 78 | - | AVL9 |
| 7 | 35840809 | SNV | G | C | 1 | 25178 | Hetero | 15 | 30 | 50.0 | 0.44 | 0 | 40 | - | SEPT7 |
| 7 | 39892622 | SNV | C | T | 1 | 25178 | Hetero | 90 | 215 | 41.9 | 0.47 | 0 | 336 | - | AC072061.2 |
| 7 | 44026251 | SNV | T | C | 1 | 25178 | Hetero | 25 | 163 | 15.3 | 0.11 | 0 | 106 | - | POLR2J4 |
| 7 | 44887481 | SNV | G | A | 1 | 25178 | Hetero | 215 | 355 | 60.6 | 0.49 | 0 | 348 | - | H2AFV |
| 7 | 47882595 | SNV | T | C | 1 | 25178 | Hetero | 37 | 77 | 48.1 | 0.26 | 0 | 83 | Yes | HUS1 |
| 7 | 57533233 | SNV | A | T | 1 | 25178 | Hetero | 15 | 35 | 42.9 | 0.32 | 0 | 42 | - | ZNF716 |
| 7 | 64312976 | SNV | C | G | 1 | 25178 | Hetero | 57 | 164 | 34.8 | 0.48 | 0 | 201 | - | ENSG00000182722 |
| 7 | 65425712 | Deletion | T | - | 1 | 25178 | Hetero | 20 | 40 | 50.0 | 0.39 | 0 | 44 | - | GUSB |
| 7 | 75034350 | SNV | T | C | 1 | 25178 | Hetero | 95 | 189 | 50.3 | 0.38 | 0 | 202 | Yes | TRIM73 |
| 7 | 75166709 | SNV | T | C | 1 | 25178 | Hetero | 53 | 87 | 60.9 | 0.46 | 0 | 112 | - | HIP1 |
| 7 | 86507291 | SNV | T | C | 1 | 25178 | Hetero | 41 | 111 | 36.9 | 0.48 | 0 | 133 | - | KIAA1324L |
| 7 | 89783856 | SNV | G | A | 1 | 25178 | Hetero | 19 | 37 | 51.4 | 0.41 | 0 | 42 | - | STEAP2-AS1 |
| 7 | 93055883 | SNV | G | A | 1 | 25178 | Hetero | 59 | 162 | 36.4 | 0.38 | 0 | 147 | Yes | CALCR |
| 7 | 93625680 | SNV | A | T | 1 | 25178 | Hetero | 18 | 53 | 34.0 | 0.15 | 0 | 58 | - | BET1 |
| 7 | 94927443 | SNV | G | C | 1 | 25178 | Hetero | 41 | 83 | 49.4 | 0.48 | 0 | 110 | - | PON1 |
| 7 | 99705142 | SNV | G | A | 1 | 25178 | Hetero | 50 | 124 | 40.3 | 0.27 | 0 | 102 | No | AP4M1 |
| 7 | 100488301 | SNV | C | A | 1 | 25178 | Hetero | 61 | 115 | 53.0 | 0.49 | 0 | 114 | - | ACHE |
| 7 | 101893581 | SNV | A | C | 1 | 25178 | Hetero | 153 | 260 | 58.8 | 0.32 | 0 | 226 | - | CUX1 |
| 7 | 102180562 | SNV | A | C | 1 | 25178 | Homo | 48 | 48 | 100.0 | 0.15 | 0 | 115 | - | RASA4B |
| 7 | 102279670 | SNV | A | C | 1 | 25178 | Hetero | 17 | 168 | 10.1 | 0.50 | 0 | 150 | - | POLR2J2 |
| 7 | 105636790 | SNV | C | A | 1 | 25178 | Hetero | 36 | 95 | 37.9 | 0.13 | 0 | 117 | Yes | CDHR3 |
| 7 | 106513198 | SNV | G | A | 1 | 25178 | Hetero | 178 | 335 | 53.1 | 0.49 | 0 | 368 | Yes | PIK3CG |
| 7 | 107626733 | SNV | C | T | 1 | 25178 | Hetero | 121 | 314 | 38.5 | 0.46 | 0 | 297 | Yes | LAMB1 |
| 7 | 112724835 | SNV | C | G | 1 | 25178 | Hetero | 47 | 100 | 47.0 | 0.37 | 0 | 125 | - | GPR85 |
| 7 | 113518995 | SNV | C | T | 1 | 25178 | Hetero | 144 | 357 | 40.3 | 0.42 | 0 | 452 | Yes | PPP1R3A |
| 7 | 115575938 | Deletion | C | - | 1 | 25178 | Hetero | 42 | 102 | 41.2 | 0.47 | 0 | 95 | - | TFEC |
| 7 | 123101564 | SNV | G | A | 1 | 25178 | Hetero | 93 | 180 | 51.7 | 0.37 | 0 | 202 | No | IQUB |
| 7 | 123600027 | SNV | C | T | 1 | 25178 | Hetero | 35 | 173 | 20.2 | 0.28 | 0 | 213 | - | SPAM1 |
| 7 | 131814536 | SNV | C | A | 1 | 25178 | Hetero | 69 | 119 | 58.0 | 0.38 | 0 | 113 | - | PLXNA4 |
| 7 | 142247193 | SNV | C | A | 1 | 25178 | Hetero | 115 | 159 | 72.3 | 0.38 | 0 | 169 | Yes | TRBV7-3 |
| 7 | 149990476 | SNV | A | G | 1 | 25178 | Hetero | 68 | 196 | 34.7 | 0.45 | 0 | 212 | No | ACTR3C |
| 7 | 154794594 | SNV | C | T | 1 | 25178 | Hetero | 13 | 90 | 14.4 | 0.08 | 0 | 88 | Yes | PAXIP1 |
| 7 | 154877218 | SNV | G | A | 1 | 25178 | Hetero | 53 | 81 | 65.4 | 0.44 | 0 | 100 | - | HTR5A |
| 7 | 155256505 | SNV | C | T | 1 | 25178 | Hetero | 71 | 113 | 62.8 | 0.24 | 0 | 145 | - | EN2 |
| 7 | 155404022 | SNV | C | T | 1 | 25178 | Hetero | 85 | 208 | 40.9 | 0.44 | 0 | 222 | Yes | AC009403.2 |
| 7 | 158555832 | SNV | G | T | 1 | 25178 | Hetero | 31 | 76 | 40.8 | 0.29 | 0 | 68 | Yes | ESYT2 |
| 7 | 101923315 | Insertion | - | C | 1 | 25178 | Homo | 150 | 150 | 100.0 | 0.48 | 0 | 193 | - | CUX1 |
| 7 | 105208020 | Insertion | - | AA | 2 | 25178 | Hetero | 41 | 66 | 62.1 | 0.28 | 0 | 85 | - | RINT1 |
| 7 | 116199666 | Insertion | - | G | 1 | 25178 | Hetero | 13 | 80 | 16.3 | 0.21 | 0 | 83 | - | CAV1 |
| 7 | 123177881 | Deletion | AA | - | 2 | 25178 | Hetero | 23 | 58 | 39.7 | 0.48 | 0 | 80 | - | NDUFA5 |
| 7 | 129414762 | Deletion | CT | - | 2 | 25178 | Hetero | 37 | 67 | 55.2 | 0.39 | 0 | 66 | - | ENSG00000207691 |
| 7 | 130827598 | Insertion | - | A | 1 | 25178 | Hetero | 94 | 166 | 56.6 | 0.41 | 0 | 193 | - | MKLN1 |
| 7 | 149561754 | Insertion | - | TT | 2 | 25178 | Hetero | 49 | 121 | 40.5 | 0.49 | 0 | 132 | - | ZNF862 |
| 7 | 17920861 | Deletion | AC | - | 2 | 25178 | Hetero | 4 | 31 | 12.9 | 0.25 | 0 | 36 | - | SNX13 |
| 7 | 22980929 | Insertion | - | AC | 2 | 25178 | Hetero | 24 | 45 | 53.3 | 0.25 | 0 | 46 | - | FAM126A |
| 7 | 29544337 | Insertion | - | TC | 2 | 25178 | Hetero | 80 | 136 | 58.8 | 0.44 | 0 | 119 | - | CHN2 |
| 7 | 30199756 | Insertion | - | TG | 2 | 25178 | Hetero | 126 | 262 | 48.1 | 0.45 | 0 | 303 | - | C7orf41 |
| 7 | 41728916 | Insertion | - | G | 1 | 25178 | Hetero | 32 | 95 | 33.7 | 0.38 | 0 | 109 | - | INHBA |
| 7 | 44152524 | Insertion | - | G | 1 | 25178 | Hetero | 133 | 212 | 62.7 | 0.46 | 0 | 235 | - | AEBP1 |
| 7 | 6866101 | Insertion | - | T | 1 | 25178 | Hetero | 52 | 202 | 25.7 | 0.10 | 0 | 202 | - | CCZ1B |
| 7 | 96649884 | Insertion | - | G | 1 | 25178 | Hetero | 76 | 121 | 62.8 | 0.37 | 0 | 117 | - | DLX5 |
| 8 | 1732555 | SNV | C | G | 1 | 25178 | Hetero | 46 | 98 | 46.9 | 0.34 | 0 | 81 | - | CLN8 |
| 8 | 3046601 | SNV | T | C | 1 | 25178 | Hetero | 40 | 87 | 46.0 | 0.20 | 0 | 90 | - | CSMD1 |
| 8 | 3474299 | SNV | A | G | 1 | 25178 | Hetero | 142 | 319 | 44.5 | 0.37 | 0 | 355 | No | CSMD1 |
| 8 | 7398538 | SNV | C | G | 1 | 25178 | Hetero | 73 | 292 | 25.0 | 0.48 | 0 | 187 | No | RP11-1118M6.1 |
| 8 | 11659998 | SNV | A | T | 1 | 25178 | Hetero | 176 | 312 | 56.4 | 0.43 | 0 | 229 | - | FDFT1 |
| 8 | 18388243 | SNV | G | A | 1 | 25178 | Hetero | 51 | 106 | 48.1 | 0.47 | 0 | 107 | - | PSD3 |
| 8 | 19677057 | Deletion | A | - | 1 | 25178 | Hetero | 17 | 37 | 45.9 | 0.11 | 0 | 35 | - | INTS10 |
| 8 | 21966886 | SNV | C | T | 1 | 25178 | Hetero | 28 | 63 | 44.4 | 0.46 | 0 | 57 | Yes | NUDT18 |
| 8 | 22412029 | SNV | C | G | 1 | 25178 | Hetero | 58 | 93 | 62.4 | 0.40 | 0 | 74 | Yes | SORBS3 |
| 8 | 23287739 | SNV | G | A | 1 | 25178 | Hetero | 58 | 116 | 50.0 | 0.39 | 0 | 108 | - | ENTPD4 |
| 8 | 28925523 | SNV | G | C | 1 | 25178 | Hetero | 45 | 100 | 45.0 | 0.47 | 0 | 114 | - | CTD-2647L4.5 |
| 8 | 30494493 | SNV | C | T | 1 | 25178 | Hetero | 16 | 30 | 53.3 | 0.26 | 0 | 50 | - | GTF2E2 |
| 8 | 55543161 | Deletion | T | - | 1 | 25178 | Hetero | 13 | 100 | 13.0 | 0.36 | 0 | 94 | - | RP1 |
| 8 | 56923298 | SNV | C | A | 1 | 25178 | Hetero | 49 | 122 | 40.2 | 0.47 | 0 | 93 | - | LYN |
| 8 | 61778294 | SNV | C | T | 1 | 25178 | Hetero | 48 | 269 | 17.8 | 0.29 | 0 | 336 | No | CHD7 |
| 8 | 62577474 | SNV | C | T | 1 | 25178 | Hetero | 29 | 74 | 39.2 | 0.49 | 0 | 66 | - | ASPH |
| 8 | 66753974 | SNV | C | A | 1 | 25178 | Hetero | 36 | 58 | 62.1 | 0.11 | 0 | 69 | - | PDE7A |
| 8 | 67476071 | SNV | T | C | 1 | 25178 | Hetero | 48 | 78 | 61.5 | 0.50 | 0 | 63 | - | MYBL1 |
| 8 | 68423909 | SNV | A | G | 1 | 25178 | Hetero | 141 | 257 | 54.9 | 0.33 | 0 | 211 | - | CPA6 |
| 8 | 81083948 | SNV | C | A | 1 | 25178 | Hetero | 38 | 90 | 42.2 | 0.29 | 0 | 60 | - | TPD52 |
| 8 | 86386497 | SNV | T | A | 1 | 25178 | Hetero | 158 | 245 | 64.5 | 0.16 | 0 | 270 | - | CA2 |
| 8 | 87623762 | SNV | G | A | 1 | 25178 | Hetero | 31 | 63 | 49.2 | 0.23 | 0 | 53 | - | CNGB3 |
| 8 | 98864933 | SNV | G | T | 1 | 25178 | Hetero | 24 | 48 | 50.0 | 0.41 | 0 | 41 | - | LAPTM4B |
| 8 | 99204772 | Deletion | A | - | 1 | 25178 | Hetero | 82 | 151 | 54.3 | 0.44 | 0 | 127 | - | NIPAL2 |
| 8 | 101092515 | SNV | T | C | 1 | 25178 | Hetero | 137 | 252 | 54.4 | 0.29 | 0 | 222 | No | RGS22 |
| 8 | 101931677 | SNV | C | T | 1 | 25178 | Hetero | 138 | 356 | 38.8 | 0.38 | 0 | 296 | - | YWHAZ |
| 8 | 104426910 | SNV | T | C | 1 | 25178 | Hetero | 54 | 87 | 62.1 | 0.23 | 0 | 95 | - | SLC25A32 |
| 8 | 113301669 | SNV | G | A | 1 | 25178 | Hetero | 55 | 119 | 46.2 | 0.45 | 0 | 105 | Yes | CSMD3 |
| 8 | 116421510 | Deletion | C | - | 1 | 25178 | Hetero | 19 | 65 | 29.2 | 0.10 | 0 | 52 | - | TRPS1 |
| 8 | 116423835 | SNV | A | T | 1 | 25178 | Hetero | 60 | 116 | 51.7 | 0.50 | 0 | 123 | - | TRPS1 |
| 8 | 118540923 | SNV | T | C | 1 | 25178 | Hetero | 67 | 172 | 39.0 | 0.36 | 0 | 172 | Yes | MED30 |
| 8 | 118552375 | Deletion | T | - | 1 | 25178 | Hetero | 16 | 37 | 43.2 | 0.31 | 0 | 37 | - | MED30 |
| 8 | 125990735 | SNV | C | T | 1 | 25178 | Hetero | 13 | 54 | 24.1 | 0.38 | 0 | 40 | - | ZNF572 |
| 8 | 126012177 | SNV | A | G | 1 | 25178 | Hetero | 120 | 290 | 41.4 | 0.38 | 0 | 197 | - | SQLE |
| 8 | 127568611 | SNV | G | C | 1 | 25178 | Hetero | 44 | 100 | 44.0 | 0.30 | 0 | 103 | - | FAM84B |
| 8 | 133015021 | SNV | T | A | 1 | 25178 | Hetero | 123 | 217 | 56.7 | 0.16 | 0 | 202 | - | EFR3A |
| 8 | 133090149 | SNV | A | G | 1 | 25178 | Hetero | 119 | 253 | 47.0 | 0.47 | 0 | 225 | Yes | RP11-240B13.2 |
| 8 | 133859076 | Deletion | A | - | 1 | 25178 | Homo | 59 | 59 | 100.0 | 0.42 | 0 | 68 | - | PHF20L1 |
| 8 | 133860996 | SNV | C | G | 1 | 25178 | Homo | 115 | 116 | 99.1 | 0.48 | 0 | 117 | - | PHF20L1 |
| 8 | 139164635 | SNV | C | T | 1 | 25178 | Hetero | 86 | 321 | 26.8 | 0.39 | 0 | 342 | Yes | FAM135B |
| 8 | 141526197 | Deletion | T | - | 1 | 25178 | Hetero | 67 | 107 | 62.6 | 0.22 | 0 | 112 | - | CHRAC1 |
| 8 | 142367315 | SNV | C | T | 1 | 25178 | Hetero | 61 | 235 | 26.0 | 0.39 | 0 | 182 | Yes | GPR20 |
| 8 | 144695918 | SNV | C | T | 1 | 25178 | Hetero | 85 | 183 | 46.4 | 0.41 | 0 | 104 | - | TSTA3 |
| 8 | 144946621 | SNV | G | A | 1 | 25178 | Hetero | 109 | 185 | 58.9 | 0.43 | 0 | 103 | No | EPPK1 |
| 8 | 145006929 | SNV | C | T | 1 | 25178 | Hetero | 175 | 310 | 56.5 | 0.47 | 0 | 202 | - | PLEC |
| 8 | 145159036 | SNV | A | G | 1 | 25178 | Hetero | 132 | 270 | 48.9 | 0.35 | 0 | 205 | - | SHARPIN |
| 8 | 145166868 | SNV | G | T | 1 | 25178 | Hetero | 118 | 260 | 45.4 | 0.43 | 0 | 181 | Yes | KIAA1875 |
| 8 | 145582844 | SNV | C | T | 1 | 25178 | Hetero | 69 | 174 | 39.7 | 0.22 | 0 | 146 | - | SLC52A2 |
| 8 | 145726503 | SNV | C | T | 1 | 25178 | Hetero | 127 | 315 | 40.3 | 0.34 | 0 | 238 | No | PPP1R16A |
| 8 | 146017337 | SNV | G | C | 1 | 25178 | Hetero | 139 | 344 | 40.4 | 0.38 | 0 | 267 | - | RPL8 |
| 8 | 146231387 | SNV | C | T | 1 | 25178 | Hetero | 25 | 52 | 48.1 | 0.33 | 0 | 57 | - | ENSG00000255559 |
| 8 | 110542343 | Insertion | - | A | 1 | 25178 | Homo | 130 | 132 | 98.5 | 0.48 | 0 | 126 | - | PKHD1L1 |
| 8 | 120790378 | Insertion | - | A | 1 | 25178 | Hetero | 36 | 55 | 65.5 | 0.48 | 0 | 50 | - | TAF2 |
| 8 | 124513603 | Insertion | - | AA | 2 | 25178 | Hetero | 31 | 104 | 29.8 | 0.23 | 0 | 73 | - | FBXO32 |
| 8 | 126051155 | Insertion | - | A | 1 | 25178 | Hetero | 33 | 97 | 34.0 | 0.24 | 0 | 74 | Insertion | KIAA0196 |
| 8 | 12879694 | Insertion | - | T | 1 | 25178 | Hetero | 48 | 80 | 60.0 | 0.12 | 0 | 84 | - | KIAA1456 |
| 8 | 15623975 | Deletion | TG | - | 2 | 25178 | Hetero | 6 | 49 | 12.2 | 0.43 | 0 | 47 | - | TUSC3 |
| 8 | 20002739 | Insertion | - | G | 1 | 25178 | Homo | 38 | 38 | 100.0 | 0.42 | 0 | 33 | - | SLC18A1 |
| 8 | 20036018 | Insertion | - | AA | 2 | 25178 | Hetero | 15 | 62 | 24.2 | 0.29 | 0 | 53 | - | SLC18A1 |
| 8 | 24324831 | Insertion | - | T | 1 | 25178 | Hetero | 40 | 107 | 37.4 | 0.43 | 0 | 100 | - | ADAM7 |
| 8 | 27593440 | Insertion | - | T | 1 | 25178 | Hetero | 29 | 68 | 42.6 | 0.41 | 0 | 73 | - | CCDC25 |
| 8 | 29965268 | Insertion | - | T | 1 | 25178 | Homo | 118 | 119 | 99.2 | 0.37 | 0 | 76 | - | LEPROTL1 |
| 8 | 57212734 | Insertion | - | TT | 2 | 25178 | Hetero | 61 | 119 | 51.3 | 0.31 | 0 | 110 | - | SDR16C5 |
| 8 | 59494754 | Insertion | - | TG | 2 | 25178 | Hetero | 83 | 293 | 28.3 | 0.45 | 0 | 334 | - | SDCBP |
| 8 | 89046466 | Insertion | - | A | 1 | 25178 | Hetero | 9 | 71 | 12.7 | 0.40 | 0 | 86 | - | MMP16 |
| 8 | 89081734 | Insertion | - | A | 1 | 25178 | Hetero | 60 | 168 | 35.7 | 0.39 | 0 | 161 | - | MMP16 |
| 8 | 97243524 | Insertion | - | T | 1 | 25178 | Hetero | 211 | 376 | 56.1 | 0.43 | 0 | 354 | Insertion | UQCRB |
| 9 | 5163849 | Deletion | A | - | 1 | 25178 | Hetero | 28 | 33 | 84.8 | 0.47 | 0 | 72 | - | RP11-39K24.10 |
| 9 | 5470465 | SNV | A | G | 1 | 25178 | Hetero | 17 | 36 | 47.2 | 0.42 | 0 | 35 | - | CD274 |
| 9 | 15745515 | SNV | C | T | 1 | 25178 | Hetero | 22 | 72 | 30.6 | 0.13 | 0 | 111 | Yes | CCDC171 |
| 9 | 17502536 | SNV | G | A | 1 | 25178 | Hetero | 14 | 46 | 30.4 | 0.18 | 0 | 72 | - | CNTLN |
| 9 | 18770634 | SNV | G | A | 1 | 25178 | Hetero | 17 | 40 | 42.5 | 0.45 | 0 | 45 | Yes | ADAMTSL1 |
| 9 | 19527988 | SNV | A | T | 1 | 25178 | Hetero | 13 | 70 | 18.6 | 0.31 | 0 | 140 | - | SLC24A2 |
| 9 | 33112632 | SNV | A | C | 1 | 25178 | Hetero | 13 | 63 | 20.6 | 0.50 | 0 | 77 | - | B4GALT1 |
| 9 | 33533472 | SNV | C | T | 1 | 25178 | Hetero | 18 | 77 | 23.4 | 0.25 | 0 | 183 | No | ANKRD18B |
| 9 | 34254038 | SNV | C | G | 1 | 25178 | Hetero | 23 | 70 | 32.9 | 0.44 | 0 | 120 | - | KIF24 |
| 9 | 35077291 | SNV | C | T | 1 | 25178 | Hetero | 18 | 75 | 24.0 | 0.32 | 0 | 99 | Yes | FANCG |
| 9 | 85958290 | SNV | C | T | 1 | 25178 | Hetero | 26 | 110 | 23.6 | 0.45 | 0 | 146 | - | FRMD3 |
| 9 | 87285407 | SNV | C | A | 1 | 25178 | Hetero | 19 | 97 | 19.6 | 0.45 | 0 | 135 | - | NTRK2 |
| 9 | 94488902 | SNV | G | A | 1 | 25178 | Hetero | 14 | 47 | 29.8 | 0.29 | 0 | 53 | Yes | ROR2 |
| 9 | 96713776 | SNV | A | C | 1 | 25178 | Hetero | 24 | 53 | 45.3 | 0.44 | 0 | 85 | - | BARX1 |
| 9 | 109774309 | SNV | G | A | 1 | 25178 | Hetero | 51 | 73 | 69.9 | 0.31 | 0 | 96 | - | ZNF462 |
| 9 | 115927415 | SNV | A | G | 1 | 25178 | Hetero | 21 | 89 | 23.6 | 0.32 | 0 | 162 | - | FKBP15 |
| 9 | 119163075 | SNV | A | G | 1 | 25178 | Hetero | 20 | 76 | 26.3 | 0.40 | 0 | 118 | - | PAPPA |
| 9 | 123752088 | SNV | T | C | 1 | 25178 | Hetero | 37 | 48 | 77.1 | 0.14 | 0 | 79 | - | C5 |
| 9 | 123837257 | SNV | C | T | 1 | 25178 | Hetero | 26 | 111 | 23.4 | 0.33 | 0 | 179 | - | CNTRL |
| 9 | 125315712 | SNV | C | T | 1 | 25178 | Hetero | 78 | 196 | 39.8 | 0.43 | 0 | 345 | No | OR1N2 |
| 9 | 125330378 | SNV | C | T | 1 | 25178 | Hetero | 45 | 62 | 72.6 | 0.43 | 0 | 97 | Yes | OR1L8 |
| 9 | 127217354 | SNV | C | T | 1 | 25178 | Hetero | 20 | 76 | 26.3 | 0.36 | 0 | 111 | Yes | GPR144 |
| 9 | 131023043 | SNV | C | T | 1 | 25178 | Hetero | 25 | 32 | 78.1 | 0.48 | 0 | 67 | Yes | GOLGA2 |
| 9 | 131755816 | SNV | G | A | 1 | 25178 | Hetero | 26 | 101 | 25.7 | 0.50 | 0 | 148 | Yes | NUP188 |
| 9 | 135779907 | SNV | T | C | 1 | 25178 | Hetero | 68 | 79 | 86.1 | 0.17 | 0 | 101 | - | TSC1 |
| 9 | 135987362 | SNV | T | C | 1 | 25178 | Hetero | 74 | 104 | 71.2 | 0.47 | 0 | 129 | - | RALGDS |
| 9 | 136333096 | SNV | A | G | 1 | 25178 | Hetero | 55 | 70 | 78.6 | 0.24 | 0 | 70 | Yes | CACFD1 |
| 9 | 136578297 | SNV | T | C | 1 | 25178 | Hetero | 53 | 74 | 71.6 | 0.29 | 0 | 133 | - | SARDH |
| 9 | 137736169 | SNV | A | G | 1 | 25178 | Hetero | 70 | 93 | 75.3 | 0.37 | 0 | 174 | - | COL5A1 |
| 9 | 137806334 | SNV | A | G | 1 | 25178 | Hetero | 79 | 116 | 68.1 | 0.33 | 0 | 177 | - | FCN1 |
| 9 | 139694152 | SNV | C | G | 1 | 25178 | Hetero | 11 | 53 | 20.8 | 0.17 | 0 | 70 | - | TMEM141 |
| 9 | 139981610 | SNV | G | A | 1 | 25178 | Hetero | 29 | 86 | 33.7 | 0.32 | 0 | 135 | No | MAN1B1 |
| 9 | 140686471 | SNV | C | T | 1 | 25178 | Hetero | 26 | 36 | 72.2 | 0.31 | 0 | 49 | - | EHMT1 |
| 9 | 140686488 | SNV | A | C | 1 | 25178 | Hetero | 29 | 38 | 76.3 | 0.43 | 0 | 55 | - | EHMT1 |
| 9 | 107556794 | Deletion | AA | - | 2 | 25178 | Hetero | 8 | 73 | 11.0 | 0.13 | 0 | 115 | - | ABCA1 |
| 9 | 111903890 | Insertion | - | A | 1 | 25178 | Hetero | 36 | 50 | 72.0 | 0.24 | 0 | 105 | - | FRRS1L |
| 9 | 114306629 | Deletion | TG | - | 2 | 25178 | Hetero | 34 | 43 | 79.1 | 0.14 | 0 | 47 | - | ZNF483 |
| 9 | 117782836 | Insertion | - | A | 1 | 25178 | Homo | 81 | 83 | 97.6 | 0.32 | 0 | 108 | - | TNC |
| 9 | 123618559 | Insertion | - | G | 1 | 25178 | Hetero | 24 | 103 | 23.3 | 0.50 | 0 | 115 | - | PHF19 |
| 9 | 132481491 | Insertion | - | C | 1 | 25178 | Hetero | 14 | 68 | 20.6 | 0.35 | 0 | 91 | - | PRRX2 |
| 9 | 135469149 | Insertion | - | G | 1 | 25178 | Homo | 136 | 136 | 100.0 | 0.47 | 0 | 198 | - | DDX31 |
| 9 | 135569038 | Deletion | AA | - | 2 | 25178 | Homo | 59 | 61 | 96.7 | 0.42 | 0 | 82 | - | GTF3C4 |
| 9 | 15510019 | Insertion | - | G | 1 | 25178 | Hetero | 28 | 39 | 71.8 | 0.16 | 0 | 79 | - | PSIP1 |
| 9 | 35854484 | Insertion | - | G | 1 | 25178 | Hetero | 18 | 76 | 23.7 | 0.42 | 0 | 104 | - | TMEM8B |
| 9 | 91608049 | Insertion | - | A | 1 | 25178 | Homo | 50 | 50 | 100.0 | 0.41 | 0 | 90 | - | C9orf47 |
| 9 | 97223213 | Insertion | - | T | 1 | 25178 | Hetero | 33 | 50 | 66.0 | 0.37 | 0 | 61 | - | HIATL1 |
| 10 | 875472 | SNV | G | A | 1 | 25178 | Hetero | 24 | 73 | 32.9 | 0.39 | 0 | 77 | No | LARP4B |
| 10 | 1066769 | SNV | T | C | 1 | 25178 | Hetero | 19 | 53 | 35.8 | 0.24 | 0 | 39 | Yes | IDI2 |
| 10 | 5805327 | SNV | T | C | 1 | 25178 | Hetero | 13 | 42 | 31.0 | 0.21 | 0 | 44 | - | FAM208B |
| 10 | 11367781 | Deletion | T | - | 1 | 25178 | Hetero | 77 | 172 | 44.8 | 0.24 | 0 | 229 | - | CELF2 |
| 10 | 22675777 | SNV | G | A | 1 | 25178 | Hetero | 46 | 81 | 56.8 | 0.25 | 0 | 100 | No | SPAG6 |
| 10 | 22706500 | SNV | G | C | 1 | 25178 | Hetero | 41 | 110 | 37.3 | 0.40 | 0 | 102 | - | SPAG6 |
| 10 | 24918828 | Deletion | T | - | 1 | 25178 | Hetero | 31 | 65 | 47.7 | 0.39 | 0 | 60 | - | ARHGAP21 |
| 10 | 35859199 | SNV | T | C | 1 | 25178 | Hetero | 27 | 61 | 44.3 | 0.31 | 0 | 53 | - | CCNY |
| 10 | 38299709 | SNV | G | C | 1 | 25178 | Hetero | 64 | 146 | 43.8 | 0.41 | 0 | 178 | Yes | ZNF33A |
| 10 | 38645343 | SNV | C | T | 1 | 25178 | Hetero | 29 | 208 | 13.9 | 0.49 | 0 | 292 | - | HSD17B7P2 |
| 10 | 48373072 | SNV | C | A | 1 | 25178 | Hetero | 91 | 185 | 49.2 | 0.49 | 0 | 209 | - | ZNF488 |
| 10 | 52596001 | SNV | G | T | 1 | 25178 | Hetero | 65 | 162 | 40.1 | 0.49 | 0 | 186 | Yes | A1CF |
| 10 | 64966638 | SNV | A | G | 1 | 25178 | Hetero | 35 | 68 | 51.5 | 0.36 | 0 | 81 | No | JMJD1C |
| 10 | 65354598 | SNV | G | T | 1 | 25178 | Hetero | 52 | 198 | 26.3 | 0.45 | 0 | 212 | Yes | REEP3 |
| 10 | 71873770 | SNV | T | C | 1 | 25178 | Hetero | 79 | 128 | 61.7 | 0.47 | 0 | 148 | - | AIFM2 |
| 10 | 73571114 | SNV | T | C | 1 | 25178 | Hetero | 86 | 183 | 47.0 | 0.46 | 0 | 181 | No | CDH23 |
| 10 | 74990293 | SNV | T | G | 1 | 25178 | Hetero | 79 | 161 | 49.1 | 0.37 | 0 | 151 | - | FAM149B1 |
| 10 | 75549047 | SNV | A | G | 1 | 25178 | Hetero | 65 | 133 | 48.9 | 0.41 | 0 | 137 | - | KIAA0913 |
| 10 | 87361636 | SNV | G | A | 1 | 25178 | Hetero | 72 | 145 | 49.7 | 0.42 | 0 | 160 | - | GRID1 |
| 10 | 88024541 | SNV | A | C | 1 | 25178 | Hetero | 71 | 140 | 50.7 | 0.27 | 0 | 143 | - | GRID1 |
| 10 | 88232494 | SNV | G | T | 1 | 25178 | Hetero | 31 | 54 | 57.4 | 0.17 | 0 | 63 | Yes | WAPAL |
| 10 | 90305196 | SNV | A | G | 1 | 25178 | Hetero | 51 | 96 | 53.1 | 0.49 | 0 | 99 | - | RNLS |
| 10 | 95278715 | SNV | C | T | 1 | 25178 | Hetero | 113 | 245 | 46.1 | 0.47 | 0 | 293 | Yes | CEP55 |
| 10 | 96066392 | SNV | A | G | 1 | 25178 | Hetero | 49 | 104 | 47.1 | 0.12 | 0 | 126 | Yes | PLCE1 |
| 10 | 99118339 | SNV | T | G | 1 | 25178 | Hetero | 42 | 102 | 41.2 | 0.38 | 0 | 101 | Yes | RRP12 |
| 10 | 102723542 | SNV | G | T | 1 | 25178 | Hetero | 24 | 48 | 50.0 | 0.31 | 0 | 37 | - | FAM178A |
| 10 | 102825186 | SNV | C | T | 1 | 25178 | Hetero | 115 | 246 | 46.7 | 0.32 | 0 | 228 | - | KAZALD1 |
| 10 | 115334172 | Deletion | C | - | 1 | 25178 | Hetero | 48 | 101 | 47.5 | 0.21 | 0 | 79 | - | HABP2 |
| 10 | 115885734 | SNV | T | C | 1 | 25178 | Hetero | 140 | 282 | 49.6 | 0.42 | 0 | 301 | Yes | C10orf118 |
| 10 | 115964548 | SNV | C | T | 1 | 25178 | Hetero | 48 | 102 | 47.1 | 0.42 | 0 | 103 | Yes | TDRD1 |
| 10 | 116331204 | SNV | C | G | 1 | 25178 | Hetero | 49 | 119 | 41.2 | 0.19 | 0 | 124 | - | ABLIM1 |
| 10 | 129903282 | SNV | A | G | 1 | 25178 | Hetero | 119 | 269 | 44.2 | 0.37 | 0 | 273 | No | MKI67 |
| 10 | 131905206 | SNV | T | C | 1 | 25178 | Hetero | 13 | 104 | 12.5 | 0.29 | 0 | 131 | - | RP11-500G10.1 |
| 10 | 135438888 | SNV | C | T | 1 | 25178 | Hetero | 70 | 191 | 36.6 | 0.28 | 0 | 117 | No | FRG2B |
| 10 | 112328650 | Insertion | - | T | 1 | 25178 | Hetero | 18 | 48 | 37.5 | 0.26 | 0 | 54 | - | SMC3 |
| 10 | 118443282 | Deletion | GA | - | 2 | 25178 | Hetero | 59 | 99 | 59.6 | 0.47 | 0 | 107 | Deletion | HSPA12A |
| 10 | 129250569 | Insertion | - | AC | 2 | 25178 | Hetero | 17 | 56 | 30.4 | 0.45 | 0 | 69 | - | DOCK1 |
| 10 | 131634900 | Insertion | - | A | 1 | 25178 | Hetero | 24 | 46 | 52.2 | 0.39 | 0 | 62 | - | EBF3 |
| 10 | 16737156 | Insertion | - | G | 1 | 25178 | Hetero | 7 | 53 | 13.2 | 0.14 | 0 | 112 | - | RSU1 |
| 10 | 58117546 | Insertion | - | C | 1 | 25178 | Hetero | 43 | 87 | 49.4 | 0.34 | 0 | 78 | - | ZWINT |
| 10 | 63855143 | Insertion | - | G | 1 | 25178 | Hetero | 28 | 75 | 37.3 | 0.40 | 0 | 75 | - | ARID5B |
| 10 | 75883134 | Insertion | - | TT | 2 | 25178 | Hetero | 65 | 112 | 58.0 | 0.49 | 0 | 114 | - | AP3M1 |
| 11 | 207412 | SNV | C | A | 1 | 25178 | Hetero | 5 | 36 | 13.9 | 0.40 | 0 | 39 | - | BET1L |
| 11 | 2152379 | SNV | T | C | 1 | 25178 | Hetero | 121 | 222 | 54.5 | 0.49 | 0 | 248 | - | IGF2 |
| 11 | 2337268 | SNV | A | C | 1 | 25178 | Hetero | 41 | 78 | 52.6 | 0.43 | 0 | 72 | - | TSPAN32 |
| 11 | 2426173 | SNV | G | A | 1 | 25178 | Hetero | 26 | 55 | 47.3 | 0.37 | 0 | 77 | No | TRPM5 |
| 11 | 5529318 | SNV | T | C | 1 | 25178 | Hetero | 107 | 170 | 62.9 | 0.49 | 0 | 211 | Yes | HBG2 |
| 11 | 5905801 | SNV | G | C | 1 | 25178 | Hetero | 64 | 158 | 40.5 | 0.47 | 0 | 245 | Yes | TRIM5 |
| 11 | 7614534 | SNV | A | G | 1 | 25178 | Hetero | 40 | 78 | 51.3 | 0.11 | 0 | 83 | - | PPFIBP2 |
| 11 | 8788297 | SNV | G | A | 1 | 25178 | Hetero | 46 | 82 | 56.1 | 0.39 | 0 | 61 | - | ST5 |
| 11 | 9773245 | SNV | C | T | 1 | 25178 | Hetero | 5 | 44 | 11.4 | 0.20 | 0 | 57 | - | SWAP70 |
| 11 | 12965782 | SNV | G | A | 1 | 25178 | Hetero | 33 | 58 | 56.9 | 0.39 | 0 | 78 | - | TEAD1 |
| 11 | 14992600 | SNV | A | G | 1 | 25178 | Hetero | 46 | 133 | 34.6 | 0.18 | 0 | 152 | - | CALCB |
| 11 | 17373579 | SNV | G | T | 1 | 25178 | Hetero | 38 | 86 | 44.2 | 0.07 | 0 | 111 | No | NCR3LG1 |
| 11 | 17523020 | SNV | C | T | 1 | 25178 | Hetero | 22 | 109 | 20.2 | 0.17 | 0 | 117 | - | USH1C |
| 11 | 18124712 | SNV | G | A | 1 | 25178 | Hetero | 44 | 220 | 20.0 | 0.28 | 0 | 241 | - | SAAL1 |
| 11 | 22303068 | Deletion | A | - | 1 | 25178 | Hetero | 21 | 74 | 28.4 | 0.32 | 0 | 105 | - | ANO5 |
| 11 | 26563574 | SNV | C | A | 1 | 25178 | Hetero | 20 | 34 | 58.8 | 0.30 | 0 | 40 | No | ANO3 |
| 11 | 30406491 | SNV | T | A | 1 | 25178 | Hetero | 57 | 113 | 50.4 | 0.45 | 0 | 130 | - | MPPED2 |
| 11 | 32955294 | SNV | T | A | 1 | 25178 | Hetero | 74 | 173 | 42.8 | 0.42 | 0 | 249 | No | QSER1 |
| 11 | 34514948 | SNV | G | A | 1 | 25178 | Hetero | 86 | 169 | 50.9 | 0.37 | 0 | 189 | - | ELF5 |
| 11 | 35641844 | SNV | G | C | 1 | 25178 | Hetero | 77 | 155 | 49.7 | 0.33 | 0 | 165 | - | FJX1 |
| 11 | 35642248 | Deletion | A | - | 1 | 25178 | Hetero | 15 | 49 | 30.6 | 0.27 | 0 | 57 | - | FJX1 |
| 11 | 36486602 | SNV | T | G | 1 | 25178 | Hetero | 28 | 54 | 51.9 | 0.43 | 0 | 79 | - | PRR5L |
| 11 | 36597282 | SNV | A | G | 1 | 25178 | Hetero | 76 | 217 | 35.0 | 0.45 | 0 | 257 | Yes | RAG1 |
| 11 | 46694322 | SNV | A | G | 1 | 25178 | Hetero | 89 | 166 | 53.6 | 0.40 | 0 | 226 | - | ATG13 |
| 11 | 47008906 | SNV | C | T | 1 | 25178 | Hetero | 49 | 88 | 55.7 | 0.19 | 0 | 101 | - | C11orf49 |
| 11 | 47744635 | SNV | C | T | 1 | 25178 | Hetero | 25 | 40 | 62.5 | 0.23 | 0 | 47 | Yes | FNBP4 |
| 11 | 55872973 | SNV | G | A | 1 | 25178 | Hetero | 103 | 189 | 54.5 | 0.36 | 0 | 357 | Yes | OR8H2 |
| 11 | 57144337 | SNV | C | G | 1 | 25178 | Hetero | 73 | 126 | 57.9 | 0.33 | 0 | 146 | Yes | PRG3 |
| 11 | 57235091 | SNV | C | T | 1 | 25178 | Hetero | 133 | 203 | 65.5 | 0.38 | 0 | 243 | Yes | RTN4RL2 |
| 11 | 57996372 | SNV | G | T | 1 | 25178 | Hetero | 136 | 266 | 51.1 | 0.22 | 0 | 359 | - | OR10Q1 |
| 11 | 61568764 | SNV | G | A | 1 | 25178 | Hetero | 9 | 66 | 13.6 | 0.30 | 0 | 74 | - | FADS2 |
| 11 | 62594639 | SNV | A | G | 1 | 25178 | Hetero | 60 | 100 | 60.0 | 0.24 | 0 | 100 | No | STX5 |
| 11 | 64088232 | SNV | G | C | 1 | 25178 | Hetero | 91 | 165 | 55.2 | 0.46 | 0 | 189 | Yes | PRDX5 |
| 11 | 64138105 | SNV | C | T | 1 | 25178 | Hetero | 7 | 48 | 14.6 | 0.14 | 0 | 58 | No | RPS6KA4 |
| 11 | 64374288 | SNV | A | G | 1 | 25178 | Hetero | 58 | 102 | 56.9 | 0.23 | 0 | 95 | - | NRXN2 |
| 11 | 64726855 | SNV | T | C | 1 | 25178 | Hetero | 153 | 281 | 54.4 | 0.47 | 0 | 327 | Yes | C11orf85 |
| 11 | 64815152 | SNV | T | C | 1 | 25178 | Hetero | 47 | 134 | 35.1 | 0.43 | 0 | 114 | Yes | NAALADL1 |
| 11 | 64981718 | SNV | G | C | 1 | 25178 | Hetero | 41 | 110 | 37.3 | 0.46 | 0 | 123 | - | SLC22A20 |
| 11 | 65359139 | SNV | C | T | 1 | 25178 | Hetero | 59 | 130 | 45.4 | 0.36 | 0 | 135 | - | EHBP1L1 |
| 11 | 65413704 | SNV | C | T | 1 | 25178 | Hetero | 101 | 284 | 35.6 | 0.41 | 0 | 331 | Yes | SIPA1 |
| 11 | 65650118 | SNV | G | A | 1 | 25178 | Hetero | 74 | 174 | 42.5 | 0.35 | 0 | 174 | Yes | CTSW |
| 11 | 65659590 | SNV | G | T | 1 | 25178 | Hetero | 42 | 96 | 43.8 | 0.44 | 0 | 112 | - | FOSL1 |
| 11 | 65811215 | SNV | C | T | 1 | 25178 | Hetero | 29 | 57 | 50.9 | 0.16 | 0 | 65 | - | GAL3ST3 |
| 11 | 66313661 | SNV | C | A | 1 | 25178 | Hetero | 112 | 204 | 54.9 | 0.33 | 0 | 172 | - | ZDHHC24 |
| 11 | 66456697 | SNV | T | G | 1 | 25178 | Hetero | 47 | 118 | 39.8 | 0.32 | 0 | 114 | - | SPTBN2 |
| 11 | 66807577 | SNV | A | T | 1 | 25178 | Hetero | 38 | 96 | 39.6 | 0.10 | 0 | 118 | Yes | SYT12 |
| 11 | 67399419 | SNV | T | C | 1 | 25178 | Hetero | 27 | 51 | 52.9 | 0.35 | 0 | 57 | - | TBX10 |
| 11 | 75152415 | SNV | C | T | 1 | 25178 | Hetero | 57 | 100 | 57.0 | 0.15 | 0 | 116 | - | GDPD5 |
| 11 | 76118174 | SNV | C | T | 1 | 25178 | Hetero | 35 | 77 | 45.5 | 0.40 | 0 | 87 | - | RP11-111M22.2 |
| 11 | 76263828 | SNV | T | A | 1 | 25178 | Hetero | 44 | 68 | 64.7 | 0.46 | 0 | 77 | - | C11orf30 |
| 11 | 78367362 | SNV | A | G | 1 | 25178 | Hetero | 52 | 130 | 40.0 | 0.40 | 0 | 159 | - | ODZ4 |
| 11 | 86661943 | SNV | T | C | 1 | 25178 | Hetero | 46 | 80 | 57.5 | 0.30 | 0 | 75 | - | PRSS23 |
| 11 | 93463219 | SNV | T | C | 1 | 25178 | Hetero | 16 | 89 | 18.0 | 0.47 | 0 | 107 | Yes | KIAA1731 |
| 11 | 93522669 | SNV | T | C | 1 | 25178 | Hetero | 29 | 63 | 46.0 | 0.48 | 0 | 79 | - | MED17 |
| 11 | 93523473 | SNV | A | T | 1 | 25178 | Hetero | 56 | 125 | 44.8 | 0.31 | 0 | 138 | Yes | MED17 |
| 11 | 94599159 | SNV | C | T | 1 | 25178 | Hetero | 45 | 94 | 47.9 | 0.38 | 0 | 115 | No | AMOTL1 |
[truncated: 136,338 more chars]
