# Supplementary material for: Genomic characterization of early-stage esophageal squamous cell carcinoma in a Japanese population
Source: Oncotarget. 2019 Jun 25;10(41):4139–48. doi: 10.18632/oncotarget.27014 (PMC6609253; doi:10.18632/oncotarget.27014)
Supplement: Supplementary file 3 [file oncotarget-10-4139-s003.docx]

| **Supplementary Table 6: Somatic mutations in 26 cancerous tissues in early-stage Esophageal squamous cell carcinoma using target sequecncing** | | | | | | | | | | |  |  |  |
| --- | --- | --- | --- | --- | --- | --- | --- | --- | --- | --- | --- | --- | --- |
| SampleID | Gene | Type | Zygosity | Chr. | Position | Ref | Alt | MAF | Count | Coverage | Function | Codon | AA |
| 8372 | NOTCH2 | SNP | HET | 1 | 120478094 | C | A | 0.308 | 20 | 65 | MISSENSE | gGt/gTt | G1219V |
| 8372 | TTN | SNP | HOM | 2 | 179449478 | G | T | 0.205 | 9 | 44 | NONSENSE | taC/taA | Y12565* |
| 8372 | FAM135B | SNP | HOM | 8 | 139164848 | G | C | 0.131 | 49 | 374 | MISSENSE | Caa/Gaa | Q624E |
| 8372 | NOTCH1 | SNP | HOM | 9 | 139399229 | C | T | 0.219 | 33 | 151 | NONSENSE | tgG/tgA | W1638* |
| 8372 | TP53 | SNP | HET | 17 | 7578478 | G | C | 0.511 | 95 | 186 | MISSENSE | cCc/cGc | P112R |
| 8399 | FLG | SNP | HOM | 1 | 152281054 | G | A | 0.137 | 43 | 314 | MISSENSE | aCc/aTc | T2103I |
| 8399 | PKHD1L1 | SNP | HOM | 8 | 110447419 | A | G | 0.224 | 19 | 85 | MISSENSE | gAa/gGa | E1114G |
| 8399 | NOTCH1 | SNP | HET | 9 | 139410528 | C | T | 0.605 | 69 | 114 | MISSENSE | tGc/tAc | C525Y |
| 8399 | TDRD1 | SNP | HOM | 10 | 115987005 | C | A | 0.114 | 4 | 35 | MISSENSE | aCa/aAa | T1117K |
| 8399 | FAT3 | SNP | HET | 11 | 92533843 | C | A | 0.489 | 85 | 174 | MISSENSE | aCa/aAa | T2555K |
| 8399 | KMT2A | SNP | HOM | 11 | 118360870 | G | T | 0.125 | 4 | 32 | MISSENSE | aaG/aaT | K1534N |
| 8399 | KMT2A | SNP | HOM | 11 | 118375053 | A | G | 0.153 | 62 | 404 | MISSENSE | Agt/Ggt | S2813G |
| 8399 | MUC19 | SNP | HOM | 12 | 40907569 | A | G | 0.154 | 46 | 298 | MISSENSE | aAc/aGc | N6635S |
| 8399 | MUC19 | SNP | HOM | 12 | 40907571 | A | G | 0.153 | 46 | 301 | MISSENSE | Att/Gtt | I6636V |
| 8399 | TP53 | Deletion | HET | 17 | 7578240 | CACACGCAA | C | 0.632 | 108 | 171 |  |  |  |
| 8399 | TP53 | SNP | HET | 17 | 7578249 | A | T | 0.633 | 107 | 169 | MISSENSE | aaT/aaA | N161K |
| 8399 | MUC16 | SNP | HET | 19 | 9077530 | G | T | 0.36 | 64 | 178 | MISSENSE | Cag/Aag | Q3306K |
| 8420 | FSCB | SNP | HOM | 14 | 44974966 | G | A | 0.118 | 4 | 34 | MISSENSE | Cca/Tca | P409S |
| 8420 | TP53 | SNP | HET | 17 | 7578394 | T | C | 0.51 | 107 | 210 | MISSENSE | cAt/cGt | H140R |
| 8420 | ZNF750 | Deletion | HET | 17 | 80789802 | CGG | C | 0.483 | 161 | 333 |  |  |  |
| 8496 | PCDHGA6 | SNP | HOM | 5 | 140754849 | A | T | 0.133 | 10 | 75 | MISSENSE | aAt/aTt | N400I |
| 8496 | MUC19 | SNP | HOM | 12 | 40879080 | C | T | 0.145 | 36 | 248 | MISSENSE | gCa/gTa | A4109V |
| 8496 | MUC19 | SNP | HOM | 12 | 40879082 | A | T | 0.144 | 36 | 250 | NONSENSE | Aga/Tga | R4110* |
| 8496 | TP53 | SNP | HOM | 17 | 7578235 | T | C | 0.151 | 58 | 383 | MISSENSE | tAt/tGt | Y166C |
| 8496 | TP53 | SNP | HOM | 17 | 7579414 | C | T | 0.228 | 46 | 202 | NONSENSE | tgG/tgA | W52* |
| 8540 | THSD7B | SNP | HOM | 2 | 138373833 | T | A | 0.145 | 34 | 234 | MISSENSE | gTg/gAg | V1171E |
| 8540 | PCDHA8 | SNP | HET | 5 | 140221071 | G | T | 0.254 | 16 | 63 | MISSENSE | gaG/gaT | E55D |
| 8540 | FAT2 | SNP | HET | 5 | 150922296 | C | G | 0.272 | 76 | 279 | MISSENSE | Gag/Cag | E2798Q |
| 8540 | FAT2 | SNP | HOM | 5 | 150924951 | C | T | 0.214 | 18 | 84 | MISSENSE | Gat/Aat | D1913N |
| 8540 | FAT2 | SNP | HOM | 5 | 150930323 | C | T | 0.206 | 82 | 398 | MISSENSE | cGa/cAa | R1469Q |
| 8540 | FAT2 | SNP | HET | 5 | 150943034 | G | C | 0.328 | 20 | 61 | MISSENSE | ttC/ttG | F1142L |
| 8540 | TP53 | SNP | HET | 17 | 7577157 | T | C | 0.5 | 62 | 124 |  |  |  |
| 8540 | ZNF750 | Deletion | HET | 17 | 80789587 | TGTGTAA | T | 0.53 | 131 | 247 |  | ttttacaca/tta | FYT246L |
| 8540 | MUC16 | SNP | HOM | 19 | 9065173 | G | T | 0.105 | 4 | 38 | MISSENSE | Ctt/Att | L7425I |
| 8972 | NFE2L2 | SNP | HOM | 2 | 178095584 | G | C | 0.125 | 4 | 32 | MISSENSE | Ctg/Gtg | L483V |
| 8972 | ZNF750 | Insertion | HET | 17 | 80789615 | A | AT | 0.647 | 313 | 484 |  |  |  |
| 26710 | DHX9 | SNP | HOM | 1 | 182828195 | C | G | 0.133 | 22 | 165 | MISSENSE | agC/agG | S361R |
| 26710 | MIA3 | SNP | HOM | 1 | 222838725 | C | T | 0.108 | 18 | 167 | MISSENSE | Ctc/Ttc | L1830F |
| 26710 | MIA3 | SNP | HOM | 1 | 222838734 | C | T | 0.121 | 18 | 149 | MISSENSE | Cgg/Tgg | R1833W |
| 26710 | PCDHGB3 | SNP | HET | 5 | 140749995 | G | A | 0.257 | 29 | 113 | MISSENSE | Ggg/Agg | G12R |
| 26710 | ZAN | SNP | HOM | 7 | 100374121 | C | T | 0.213 | 20 | 94 | MISSENSE | gCg/gTg | A2139V |
| 26710 | CLUH | SNP | HET | 17 | 2594021 | A | T | 0.4 | 104 | 260 | MISSENSE | cTc/cAc | L1266H |
| 26710 | TP53 | SNP | HET | 17 | 7577586 | A | C | 0.341 | 248 | 728 | MISSENSE | aTc/aGc | I100S |
| 26710 | MUC16 | SNP | HOM | 19 | 9046582 | C | A | 0.137 | 10 | 73 | MISSENSE | gaG/gaT | E11683D |
| 27363 | SLC6A6 | SNP | HOM | 3 | 14508059 | G | A | 0.125 | 3 | 24 | MISSENSE | atG/atA | M256I |
| 27363 | CACNA1B | SNP | HOM | 9 | 140972570 | G | A | 0.143 | 4 | 28 | MISSENSE | Gcc/Acc | A1652T |
| 27363 | TP53 | SNP | HET | 17 | 7578457 | C | A | 0.284 | 44 | 155 | MISSENSE | cGc/cTc | R119L |
| 27487 | AKR7L | SNP | HOM | 1 | 19600376 | T | G | 0.139 | 5 | 36 | MISSENSE | Atg/Ctg | M65L |
| 27487 | FLG | SNP | HOM | 1 | 152280857 | T | C | 0.125 | 4 | 32 | MISSENSE | Agc/Ggc | S2169G |
| 27487 | MUC19 | SNP | HOM | 12 | 40879080 | C | T | 0.244 | 40 | 164 | MISSENSE | gCa/gTa | A4109V |
| 27487 | MUC19 | SNP | HOM | 12 | 40879082 | A | T | 0.244 | 40 | 164 | NONSENSE | Aga/Tga | R4110* |
| 27487 | NBEA | Deletion | HET | 13 | 35516972 | GCCGGGC | G | 0.375 | 33 | 88 |  | ccgggc/- | PG6- |
| 27487 | PRSS21 | SNP | HOM | 16 | 2871599 | C | T | 0.121 | 4 | 33 | MISSENSE | cCg/cTg | P299L |
| 27512 | FAT1 | SNP | HOM | 4 | 187541577 | C | T | 0.189 | 14 | 74 | MISSENSE | Gag/Aag | E2055K |
| 27512 | FAT1 | SNP | HOM | 4 | 187541586 | C | T | 0.241 | 14 | 58 | MISSENSE | Gaa/Aaa | E2052K |
| 27512 | FAT1 | SNP | HOM | 4 | 187541598 | C | G | 0.237 | 14 | 59 | MISSENSE | Gat/Cat | D2048H |
| 27512 | NOTCH1 | SNP | HET | 9 | 139412344 | C | A | 0.5 | 57 | 114 | MISSENSE | gGc/gTc | G434V |
| 27512 | MGA | SNP | HOM | 15 | 42041327 | G | A | 0.121 | 4 | 33 | MISSENSE | cGg/cAg | R1632Q |
| 27512 | TP53 | SNP | HET | 17 | 7578469 | C | A | 0.488 | 125 | 256 | MISSENSE | gGc/gTc | G115V |
| 27554 | OBSCN | SNP | HOM | 1 | 228562419 | G | T | 0.216 | 105 | 485 | SILENT | ggG/ggT | G7543 |
| 27554 | CIR1 | SNP | HET | 2 | 175215419 | G | C | 0.294 | 83 | 282 | MISSENSE | Cta/Gta | L216V |
| 27554 | CSMD3 | SNP | HET | 8 | 113358394 | G | T | 0.392 | 76 | 194 | MISSENSE | cCt/cAt | P2021H |
| 27554 | FAT3 | SNP | HET | 11 | 92577185 | C | A | 0.267 | 16 | 60 | MISSENSE | gCc/gAc | A3551D |
| 27554 | CNTNAP4 | SNP | HOM | 16 | 76569532 | A | C | 0.218 | 88 | 404 | MISSENSE | cAg/cCg | Q876P |
| 27554 | TP53 | Insertion | HET | 17 | 7578394 | T | TGGTGGGGGCA | 0.677 | 304 | 449 |  |  |  |
| 27554 | MUC16 | SNP | HOM | 19 | 9067215 | C | G | 0.226 | 30 | 133 | MISSENSE | gGc/gCc | G6744A |
| 27567 | LRP1B | SNP | HOM | 2 | 141122270 | G | T | 0.163 | 26 | 160 | MISSENSE | gaC/gaA | D3697E |
| 27567 | FAT3 | SNP | HOM | 11 | 92531403 | C | A | 0.125 | 4 | 32 | MISSENSE | Cag/Aag | Q1742K |
| 27567 | MUC19 | SNP | HET | 12 | 40879854 | G | T | 0.489 | 46 | 94 | MISSENSE | aGc/aTc | S4367I |
| 27567 | MGA | SNP | HET | 15 | 42059296 | C | G | 0.283 | 77 | 272 | MISSENSE | Cag/Gag | Q2797E |
| 27581 | CSPG4 | SNP | HOM | 15 | 75981407 | G | A | 0.105 | 4 | 38 | MISSENSE | Cgc/Tgc | R667C |
| 27581 | NOTCH3 | SNP | HOM | 19 | 15296299 | C | T | 0.105 | 4 | 38 | MISSENSE | Ggg/Agg | G715R |
| 27581 | SLC35E1 | SNP | HOM | 19 | 16664627 | C | A | 0.133 | 4 | 30 | NONSENSE | Gag/Tag | E366* |
| 27581 | TP53 | SNP | HET | 17 | 7577120 | C | T | 0.701 | 75 | 107 | MISSENSE | cGt/cAt | R114H |
| 27584 | TTN | SNP | HOM | 2 | 179430997 | G | A | 0.15 | 6 | 40 | MISSENSE | aCg/aTg | T17556M |
| 27584 | TP53 | SNP | HOM | 17 | 7578190 | T | G | 0.146 | 25 | 171 | MISSENSE | tAt/tCt | Y181S |
| 27589 | TTN | SNP | HOM | 2 | 179639752 | T | A | 0.121 | 4 | 33 | MISSENSE | cAc/cTc | H2183L |
| 27596 | PRG4 | SNP | HOM | 1 | 186277277 | G | A | 0.166 | 112 | 673 | MISSENSE | gGg/gAg | G675E |
| 27596 | FAT2 | Deletion | HOM | 5 | 150911335 | CA | C | 0.177 | 44 | 249 |  |  |  |
| 27596 | CSPG4 | SNP | HOM | 15 | 75982072 | G | A | 0.127 | 8 | 63 | MISSENSE | gCc/gTc | A445V |
| 27596 | CSPG4 | SNP | HOM | 15 | 75982085 | C | T | 0.127 | 8 | 63 | MISSENSE | Gag/Aag | E441K |
| 27596 | MUC16 | SNP | HOM | 19 | 9018463 | T | C | 0.138 | 18 | 130 | MISSENSE | Acc/Gcc | T12571A |
| 27596 | MUC16 | SNP | HOM | 19 | 9018508 | T | G | 0.155 | 20 | 129 | MISSENSE | Aag/Cag | K12556Q |
| 27599 | TP53 | SNP | HOM | 17 | 7577081 | T | C | 0.192 | 28 | 146 | MISSENSE | gAa/gGa | E127G |
| 27599 | TP53 | SNP | HOM | 17 | 7577508 | T | A | 0.188 | 88 | 467 | MISSENSE | gAa/gTa | E126V |
| 27599 | MUC16 | SNP | HOM | 19 | 9064483 | G | T | 0.114 | 4 | 35 | MISSENSE | Cag/Aag | Q7655K |
| 28383 | NOTCH1 | Deletion | HET | 9 | 139417503 | CGTT | C | 0.477 | 84 | 176 |  | aac/- | N180- |
| 28383 | TP53 | SNP | HOM | 17 | 7578526 | C | T | 0.182 | 14 | 77 | MISSENSE | tGc/tAc | C135Y |
| 28383 | TP53 | SNP | HET | 17 | 7579328 | T | C | 0.284 | 56 | 197 | MISSENSE | aAg/aGg | K120R |
| 28383 | NOTCH3 | SNP | HET | 19 | 15298786 | A | T | 0.382 | 13 | 34 | NONSENSE | tgT/tgA | C504* |
| 28463 | CSMD3 | SNP | HOM | 8 | 113871484 | C | A | 0.207 | 24 | 116 | MISSENSE | Ggc/Tgc | G445C |
| 28463 | MUC19 | SNP | HOM | 12 | 40879080 | C | T | 0.143 | 10 | 70 | MISSENSE | gCa/gTa | A4109V |
| 28463 | MUC19 | SNP | HOM | 12 | 40879082 | A | T | 0.145 | 10 | 69 | NONSENSE | Aga/Tga | R4110* |
| 28463 | ERBB2 | SNP | HOM | 17 | 37866679 | G | A | 0.215 | 72 | 335 | MISSENSE | atG/atA | M252I |
| 28524 | TENM3 | SNP | HOM | 4 | 183635384 | G | T | 0.223 | 54 | 242 | MISSENSE | gGa/gTa | G789V |
| 28524 | FAT1 | SNP | HOM | 4 | 187525001 | G | C | 0.109 | 14 | 128 | NONSENSE | tCa/tGa | S3560* |
| 28524 | FAT1 | Deletion | HET | 4 | 187630701 | CAA | C | 0.322 | 55 | 171 |  |  |  |
| 28524 | CUL9 | SNP | HOM | 6 | 43155826 | C | A | 0.111 | 4 | 36 | MISSENSE | Ctg/Atg | L653M |
| 28524 | NOTCH1 | SNP | HOM | 9 | 139412303 | G | A | 0.792 | 431 | 544 | NONSENSE | Cga/Tga | R448* |
| 28524 | FSCB | SNP | HET | 14 | 44975695 | C | A | 0.686 | 24 | 35 | MISSENSE | Gtt/Ttt | V166F |
| 28524 | CSPG4 | SNP | HOM | 15 | 75982085 | C | T | 0.123 | 14 | 114 | MISSENSE | Gag/Aag | E441K |
| 28643 | NFE2L2 | SNP | HOM | 2 | 178095914 | T | C | 0.18 | 51 | 283 | MISSENSE | Atc/Gtc | I373V |
| 28643 | PCDHB5 | SNP | HOM | 5 | 140517289 | C | T | 0.196 | 48 | 245 | MISSENSE | tCa/tTa | S758L |
| 28643 | ZFHX4 | SNP | HET | 8 | 77765990 | G | T | 0.358 | 57 | 159 | MISSENSE | cGt/cTt | R2278L |
| 28643 | MUC19 | SNP | HET | 12 | 40879080 | C | T | 0.267 | 48 | 180 | MISSENSE | gCa/gTa | A4109V |
| 28643 | MUC19 | SNP | HET | 12 | 40879082 | A | T | 0.267 | 48 | 180 | NONSENSE | Aga/Tga | R4110* |
| 28643 | TP53 | Insertion | HET | 17 | 7577514 | G | GT | 0.684 | 288 | 421 |  |  |  |
| 28643 | ZNF750 | SNP | HOM | 17 | 80790197 | T | G | 0.8 | 60 | 75 | MISSENSE | aAg/aCg | K45T |
| 28773 | FLG | SNP | HOM | 1 | 152280857 | T | C | 0.104 | 5 | 48 | MISSENSE | Agc/Ggc | S2169G |
| 28773 | PRG4 | SNP | HOM | 1 | 186277277 | G | A | 0.198 | 86 | 434 | MISSENSE | gGg/gAg | G675E |
| 28773 | PCDHGA5 | SNP | HET | 5 | 140744550 | G | A | 0.539 | 76 | 141 | MISSENSE | gGa/gAa | G218E |
| 28773 | CUL9 | SNP | HOM | 6 | 43170869 | G | T | 0.125 | 4 | 32 | MISSENSE | aGc/aTc | S1259I |
| 28773 | ZNF750 | SNP | HET | 17 | 80790256 | A | T | 0.653 | 246 | 377 | NONSENSE | taT/taA | Y25* |
| 28909 | AKR7L | SNP | HET | 1 | 19595137 | C | T | 0.556 | 35 | 63 | MISSENSE | Gcg/Acg | A255T |
| 28909 | FLG | SNP | HOM | 1 | 152280857 | T | C | 0.13 | 3 | 23 | MISSENSE | Agc/Ggc | S2169G |
| 28909 | PRG4 | SNP | HOM | 1 | 186277277 | G | A | 0.123 | 87 | 705 | MISSENSE | gGg/gAg | G675E |
| 28909 | PCDHA8 | SNP | HET | 5 | 140221012 | C | T | 0.275 | 22 | 80 | MISSENSE | Ccc/Tcc | P36S |
| 28909 | PCLO | SNP | HET | 7 | 82579519 | C | A | 0.424 | 97 | 229 | MISSENSE | aGg/aTg | R3462M |
| 28909 | ZAN | SNP | HOM | 7 | 100392906 | C | T | 0.103 | 26 | 252 | MISSENSE | Cgg/Tgg | R2727W |
| 28909 | ZFHX4 | SNP | HOM | 8 | 77690485 | C | G | 0.224 | 104 | 465 | MISSENSE | tgC/tgG | C1045W |
| 28909 | ZFHX4 | SNP | HET | 8 | 77766682 | C | A | 0.294 | 65 | 221 | MISSENSE | Cag/Aag | Q2509K |
| 28909 | NOTCH1 | SNP | HET | 9 | 139396317 | G | A | 0.451 | 105 | 233 | MISSENSE | Cgg/Tgg | R1841W |
| 28909 | TP53 | SNP | HET | 17 | 7579311 | C | T | 0.469 | 130 | 277 |  |  |  |
| 29019_SM | DHX9 | SNP | HOM | 1 | 182850535 | G | T | 0.165 | 15 | 91 | MISSENSE | Gta/Tta | V1025L |
| 29019_SM | TTN | SNP | HOM | 2 | 179418103 | C | T | 0.18 | 16 | 89 | MISSENSE | Gat/Aat | D20777N |
| 29019_SM | TTN | SNP | HOM | 2 | 179659753 | C | T | 0.188 | 35 | 186 | MISSENSE | Ggt/Agt | G381S |
| 29019_SM | BAP1 | SNP | HET | 3 | 52443861 | G | C | 0.384 | 38 | 99 | MISSENSE | Cca/Gca | P12A |
| 29019_SM | NOTCH1 | SNP | HET | 9 | 139399276 | C | A | 0.282 | 67 | 238 | NONSENSE | Gag/Tag | E1623* |
| 29019_SM | TP53 | SNP | HOM | 17 | 7578272 | G | A | 0.24 | 12 | 50 | MISSENSE | Cat/Tat | H154Y |
| 29019_T | TET2 | SNP | HOM | 4 | 106164920 | G | T | 0.121 | 4 | 33 | MISSENSE | tGt/tTt | p.C1263Y |
| 29019_T | ADAM29 | SNP | HOM | 4 | 175898483 | G | C | 0.125 | 4 | 32 | MISSENSE | Gag/Cag | p.E603* |
| 29019_T | PCDHA10 | SNP | HOM | 5 | 140236620 | G | T | 0.129 | 4 | 31 | MISSENSE | atG/atT | p.M329I |
| 29019_T | LRP5 | SNP | HOM | 11 | 68177402 | G | C | 0.131 | 84 | 643 | MISSENSE | atG/atC | p.M704I |
| 29019_T | TP53 | SNP | HET | 17 | 7578235 | T | C | 0.482 | 192 | 398 | MISSENSE | tAt/tGt | p.Y205S |
| 29019_T | MUC16 | SNP | HOM | 19 | 9059971 | C | T | 0.191 | 42 | 220 | MISSENSE | Gtt/Att |  |
| 29829 | PCDHGA6 | SNP | HET | 5 | 140755326 | C | T | 0.36 | 68 | 189 | MISSENSE | gCg/gTg | A559V |
| 29829 | PCLO | SNP | HET | 7 | 82764524 | G | T | 0.277 | 43 | 155 | MISSENSE | tCc/tAc | S781Y |
| 29829 | MUC19 | SNP | HOM | 12 | 40879080 | C | T | 0.124 | 50 | 404 | MISSENSE | gCa/gTa | A4109V |
| 29829 | MUC19 | SNP | HOM | 12 | 40879082 | A | T | 0.123 | 50 | 406 | NONSENSE | Aga/Tga | R4110* |
| 29829 | MUC19 | SNP | HOM | 12 | 40930771 | C | A | 0.107 | 33 | 308 | MISSENSE | ttC/ttA | F7395L |
| 29829 | TP53 | SNP | HET | 17 | 7577545 | T | C | 0.282 | 175 | 621 | MISSENSE | Atg/Gtg | M114V |
| 29845 | NOTCH1 | SNP | HOM | 9 | 139395192 | G | A | 0.125 | 18 | 144 | NONSENSE | Cag/Tag | Q1916* |
| 29845 | TP53 | SNP | HOM | 17 | 7578235 | T | C | 0.203 | 93 | 459 | MISSENSE | tAt/tGt | Y166C |
| 29850 | TENM3 | SNP | HOM | 4 | 183522124 | C | T | 0.24 | 31 | 129 | MISSENSE | Cct/Tct | P187S |
| 29850 | PCDHA8 | SNP | HOM | 5 | 140221012 | C | T | 0.118 | 4 | 34 | MISSENSE | Ccc/Tcc | P36S |
| 29850 | PCLO | SNP | HOM | 7 | 82580054 | C | T | 0.127 | 38 | 299 | MISSENSE | Gag/Aag | E3284K |
| 29850 | CCDC171 | SNP | HOM | 9 | 15779015 | T | G | 0.133 | 8 | 60 | MISSENSE | cTg/cGg | L983R |
| 29850 | CCDC171 | SNP | HOM | 9 | 15779020 | G | A | 0.167 | 10 | 60 | MISSENSE | Gct/Act | A985T |
| 29850 | NOTCH1 | SNP | HET | 9 | 139411782 | G | T | 0.339 | 39 | 115 | NONSENSE | tgC/tgA | C499* |
| 29850 | TP53 | Deletion | HET | 17 | 7577089 | GCGC | G | 0.388 | 50 | 129 |  | cggcgc/cgc | RR123R |
| 29850 | HKR1 | SNP | HOM | 19 | 37854415 | G | C | 0.106 | 12 | 113 | MISSENSE | aGg/aCg | R573T |
| 29850 | HKR1 | SNP | HOM | 19 | 37854420 | C | T | 0.108 | 18 | 166 | MISSENSE | Cac/Tac | H575Y |
| 29855 | PCDHA8 | SNP | HOM | 5 | 140221071 | G | T | 0.243 | 9 | 37 | MISSENSE | gaG/gaT | E55D |
| 29855 | TP53 | SNP | HET | 17 | 7577570 | C | T | 0.574 | 240 | 418 | MISSENSE | atG/atA | M105I |
| 29855 | APOBEC3B | SNP | HOM | 22 | 39381835 | T | C | 0.161 | 28 | 174 | MISSENSE | Tac/Cac | Y65H |
| Non-synonymous somatic mutations in 26 early-stage esophageal squamous cell carcinomas (ESCCs) identified using target pane mounted candidate ESCC driver genes. | | | | | | | | | | | | | |
| Chr.; Chromosome, Ref; Refference allele, Alt; mutatant allele, MAF; mutant allele freguency, Coverage is fileter read depth using molecular barcode | | | | | | | | | | | | | |
